# Selective hydroboration of unsaturated bonds by an easily accessible heterotopic cobalt catalyst

**DOI:** 10.1038/s41467-021-24117-5

**Published:** 2021-06-21

**Authors:** Chuhan Li, Shuo Song, Yuling Li, Chang Xu, Qiquan Luo, Yinlong Guo, Xiaoming Wang

**Affiliations:** 1 State Key Laboratory of Organometallic Chemistry, Center for Excellence in Molecular Synthesis, Shanghai Institute of Organic Chemistry, Chinese Academy of Sciences, Shanghai, China; 2 Department of Chemistry, Anhui University, Hefei, Anhui China; 3 Institutes of Physical Science and Information Technology, Anhui University, Hefei, Anhui China; 4 School of Chemistry and Materials Science, Hangzhou Institute for Advanced Study, University of Chinese Academy of Sciences, Hangzhou, China

**Keywords:** Homogeneous catalysis, Synthetic chemistry methodology

## Abstract

Homogeneous earth-abundant metal catalysis based on well-defined molecular complexes has achieved great advance in synthetic methodologies. However, sophisticated ligand, hazardous activator and multistep synthesis starting from base metal salts are generally required for the generation of active molecular catalysts, which may hinder their broad application in large scale organic synthesis. Therefore, the development of metal cluster catalysts formed in situ from simple earth-abundant metal salts is of importance for the practical utilization of base metal resource, yet it is still in its infancy. Herein, a mixture of catalytic amounts of cobalt (II) iodide and potassium tert-butoxide is discovered to be highly active for selective hydroboration of vinylarenes and dihydroboration of nitriles, affording a good yield of diversified hydroboration products that without isolation can readily undergo further one pot transformations. It should be highlighted that the alkoxide-pinacolborane combination acts as an efficient activation strategy to activate cobalt (II) iodide for the generation of metastable heterotopic cobalt catalysts in situ, which is proposed to be catalytically active species.

## Abstract (web summary)

Homogeneous earth-abundant metal catalysis based on well-defined metal complexes is of interest for organic synthesis, but typically employs expensive catalysts, air sensitive or synthetically challenging chemicals. Here, the authors report an efficient and regio-selective catalytic system for hydroboration of vinylarenes and organic nitriles with HBPin, using commercially available CoI_2_ and KO^*t*^Bu under ligand-free conditions.

## Introduction

**Fig. 1:**
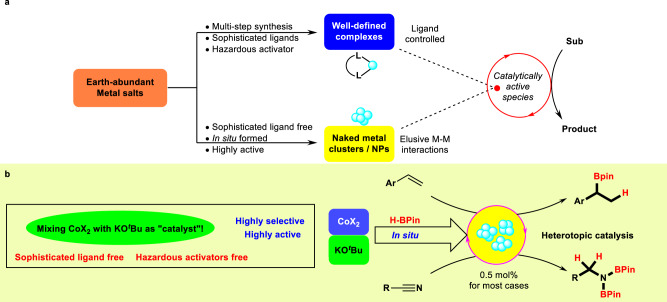
Earth-abundant metal catalysis in organic synthesis. **a** Homogenerous earth-abundant metal catalysis in synthetic methodology. **b** Simple CoX_2_ activation by KO^*t*^Bu in hydroboration reactions.

Homogeneous earth-abundant metal catalysis is one of the keys to the sustainable future in organic synthesis benefited from the advantages of cheap, earth-abundant, and less toxic base metals^1–5^. Their well-defined metal complexes have achieved great advances as homogeneous catalysts in recent years (Fig. 1a). For example, cobalt is abundant, inexpensive and a variety of their salts are commercially available^1–5^. In the past decade, several well-defined alkene hydroboration catalysts with sophisticated ligands have been developed based on cobalt complexes^6–10^. Nevertheless, these reactions typically employed catalysts bearing sophisticated ligands, which can be expensive, air sensitive, or difficult to be synthesized. In addition, current methods for the activation of the pre-catalysts to lower oxidation-state catalytic species relied heavily on the use of various hazardous reducing reagents, such as main group organometallics or hydrides^11,12^. All of these hinder their broad application in large-scale organic synthesis. Complementary to such molecularly defined systems, catalysis by metallic clusters has become a quite recent field of research which attracted the attention from the chemical community^13–19^. It has appeared that metal clusters which are formed in situ from simple metal salts may fill the gap between the single metal atom with sophisticated ligands and the metal nanoparticles (NPs) (Fig. 1a)^20–29^. They can also invoke distinct catalytic properties compared to conventional NPs. Taking cobalt again as an example, some ill-defined or nanoparticulate Co catalysts, prepared by in situ reduction of a cobalt salt with a reductant, have been reported to exhibit good hydrogenation activities^30–38^. Despite this enormous potential in catalysis, the development of metal cluster catalysts based on earth-abundant metals is still in its infancy^20–29^. From a practical perspective, the development of ligand-free heterotopic cobalt catalysts for synthetically useful alkene hydroboration reaction with HBPin, using readily available cobalt salts would be highly desirable^39–46^.

Herein, we disclose a user-friendly catalytic protocol using the mixture of CoI_2_ and KO^*t*^Bu for highly active and selective Markovnikov hydroboration of vinylarenes and double hydroboration of nitriles, without using any costly ligand/activator. It should be noted that Markovnikov hydroboration reactions are rarely approached with base-metal catalysts^47–58^. KO^*t*^Bu is proposed to act as a masked reducing agent, by reacting with HBPin to form an ate-type complex that can then serve as a reductive pre-catalyst activator^11^. Preliminary mechanistic studies suggest that the Co(II) salt is most likely to be reduced in situ to some low-valent Co species, which undergo aggregation to form heterotopic Co catalysts responsible for the catalysis (Fig. 1b). Notably, using other strong reductants such as NaBHEt_3_ or Grignard reagents lead to poor results, demonstrating the formation of the heterotopic species is largely influenced by the reductants and the alkoxide-pinacolborane combination plays a key role in the success of the present catalysis. The as-synthesized hydroboration products can serve as valuable synthons in further synthetic manipulations in a one-pot transformation, demonstrating the practicality and utility of the present methodologies.

## Results

### Reaction development

**Fig. 2:**
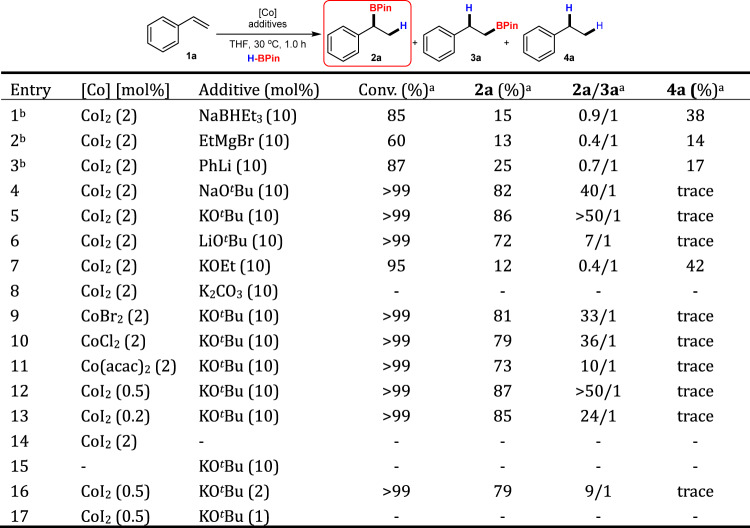
Optimization of reaction conditions for Cocatalyzed hydroboration of styrene. Reaction conditions: HBPin (0.48 mmol) was added in a portion into a mixture of the cobalt salt, the additive and **1a** (0.4 mmol) in anhydrous THF (2 mL), and the resulting mixture was stirred under N_2_ at 30 °C for 1.0 h. ^a^Determined by GC analysis of the reaction mixture. ^b^The additive was added in the end.

The study was commenced by hydroboration of styrene **1a** with HBPin, using commercially available CoI_2_ as the pre-catalyst in combination with substoichiometric amount of a reductant. The use of NaBHEt_3_, EtMgBr, or PhLi as the activator in the reaction only gave the desired hydroboration products in low yields with poor regioselectivities (Fig. 2, entries 1–3). Hydrogenated product **4a** (ethylbenzene) was also detected in these reaction mixtures, which is consistent with the good hydrogenation activities of the generated nanoparticles^30–38^. In 2017, Thomas et al. reported that a boron ‘ate’ reductive species can be formed in situ by the reaction between alkoxide and HBPin and activate high-oxidation-state cobalt complexes immediately as hydride donors^11,59^. Inspired from this pioneering work, a catalytic amount of NaO^*t*^Bu (10 mol%) was tested in the reaction, leading to a boosting in the yield of hydroboration product **2a** to 82% with excellent Markovnikov regioselectivity (40/1), highlighting the importance of the activators in the catalytic activity (Fig. 2, entry 4 vs 1–3). Changing NaO^*t*^Bu to KO^*t*^Bu slightly improved the results (Fig. 2, entry 5), whereas use of LiO^*t*^Bu resulted in a decrease in the regioselectivity (Fig. 2, entry 6). The addition of crown ethers to the reactions turned out to be deleterious for the catalysis (for details, see the Supplementary Information). These results suggested that the cations of the alkoxide salts might be involved in the in situ formed boron ‘ate’ reductive species and active cobalt species, which in turn can affect the catalytic performance. On the other hand, the use of KOEt or K_2_CO_3_ gave very poor results (Fig. 2, entries 7 and 8). Other cheap Co(II) salts, such as CoBr_2_, CoCl_2_, and Co(acac)_2_ also worked very well as catalyst precursors, suggesting that this activation mechanism should be less irrelevant to the anions of the cobalt salts (Fig. 2, entries 9–11). To our delight, reducing the loading of CoI_2_ to 0.5 mol% still afforded complete conversion of **1a** in 1.0 h, giving the product **2a** in 87% yield with >50/1 Markovnikov regioselectivity (Fig. 2, entry 12). Further diminishing the catalyst loading to 0.2 mol% was also successful, and the product **2a** was still obtained in high yield (85%) with a slightly lower regioselectivity (24/1, Fig. 2, entry 13 vs 12). Control experiments showed that no reaction of **1a** occurred in the absence of a Co precursor or KO^*t*^Bu, thus attesting the essential roles of both Co and the base for the catalysis (Fig. 2, entries 14, 15). When only 2 mol% KO^*t*^Bu was used, the regioselectivity was decreased to 9/1, whereas the reaction did not proceed at all with only 1.0 mol% KO^*t*^Bu, which was only two times the molar quantity of pre-catalyst (Fig. 2, entries 16, 17). These results seem to be consistent with the significant role of a large excess of reductant formed in situ in the present catalysis.

### Substrate scope and synthetic applications

**Fig. 3:**
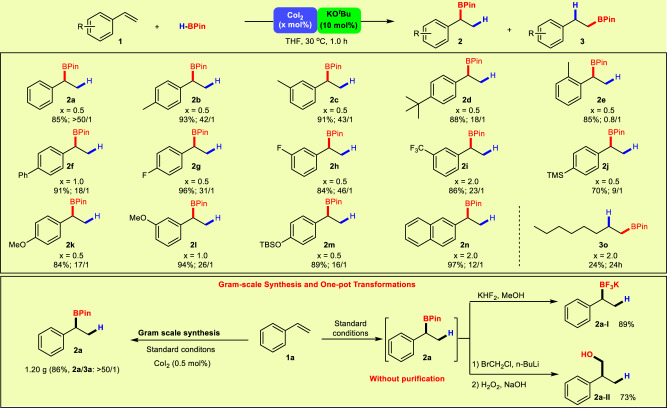
CoI_2_/KO^*t*^Bu catalyzed Markovnikov hydroboration of vinylarenes. Reaction conditions: HBPin (1.2 mmol) was added to a mixture of CoI_2_ (0.5–2.0 mol%), KO^*t*^Bu (0.1 mmol) and **1** (1.0 mmol) in 5 mL anhydrous THF, and the mixture was stirred at 30 °C for 1.0 h under N_2_. The yields are for isolated **2** and **3**. The ratio of **2**/**3** was determined by ^1^H-NMR of the crude product.

With the optimized reaction conditions in hand, the substrate scope of the Markovnikov hydroboration was investigated (Fig. 3). It is noteworthy that in most cases, the reaction consumed only 0.5 mol% of CoI_2_ and provided the targeted products in high yields with excellent regioselectivities. Styrene derivatives bearing electron-donating groups such as methyl and tert-butyl groups underwent successful hydroboration in excellent yields with high regioselectivities (**2b**–**2d**, 88–93% yields, 18/1–43/1). Styrene with *o*-methyl group on phenyl ring gave a poor regioselectivity, probably due to the unfavorable steric hinderance (**2e**). The reaction of 4-phenylstyrene using 1.0 mol% of CoI_2_ gave the secondary boronic ester **2f** in good yield with high regioselectivity (91%, 18/1). In addition, electron-withdrawing substituents including fluoro- and trifluoromethyl- were also well-tolerated, giving the corresponding secondary boronic esters **2g**–**2i** in good to excellent yields and selectivities (84–96%, 23/1–46/1). The reactions also worked efficiently for styrenes bearing trialkylsilyl- or ether substituents, giving good to excellent yields and selectivities of the branched boronic esters **2j**–**2m** (70–94%, 9/1–26/1). The reaction of 2-vinylnaphthalene afforded the branched boronic ester **2n** in good yield with high regioselectivity (97%, 12/1). However, the reactions for styrene derivatives bearing a formyl or acetoxy group only resulted in intractable mixtures. Apart from the aromatic alkenes, 1-octene can also be hydroborated using 2.0 mol% CoI_2_, giving the *anti*-Markovnikov product **3o** in only 24% isolated yield, indicating the relatively poor catalytic performance of the present system towards aliphatic olefins. Gram-scale synthesis using reaction of **1a** under 0.5 mol% CoI_2_ loading also proceeded smoothly, affording the hydroboration product **2a** in 86% yield (1.20 g) with excellent Markovnikov regioselectivity (>50/1). Treatment of the in situ generated alkylboronate **2a** with aqueous KHF_2_ afforded the corresponding trifluoroborate **2a-I** in 89% yield, which is a valuable synthetic intermediate for Suzuki cross-couplings. In addition, the reaction of the hydroboration mixture with BrCH_2_Cl and ^*n*^BuLi, followed by the oxidation, gave the product **2a-II** in 73% yield. In these cases, the reactions are performed directly on the hydroboration mixture without isolation and purification of the alkylboronate intermediate, demonstrating the utility of the present methodology.

Encouraged by the results obtained in the CoI_2_/KO^*t*^Bu mediated hydroboration of styrene derivatives, we extended this ligand-free cobalt catalytic system further into dihydroboration of nitriles^60–66^. To our delight, using only 0.5 mol% CoI_2_ and 10 mol% KO^*t*^Bu, the dihydroboration of benzonitrile **5a** worked smoothly in Et_2_O at 30 °C, giving the corresponding ammonium salt in 98% yield after the treatment with HCl (for details, see the Supplementary Information). In the absence of the cobalt salt, the reaction did not give any of the desired product. It should be noted that Findlater group and von Wangelin group developed double hydroboration of nitriles using NaBHEt_3_ and lithium bis(trimethylsilyl)amide as catalyst, respectively^67,68^.

**Fig. 4:**
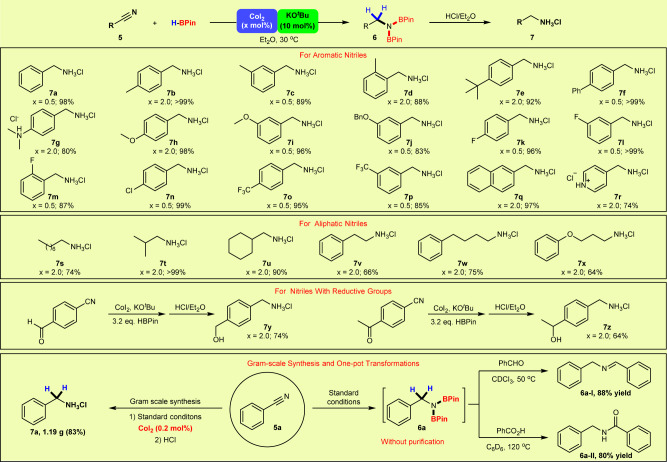
CoI_2_/KO^*t*^Bu catalyzed dihydroboration of nitriles. Reaction conditions: HBPin (2.2 mmol) was added to a mixture of CoI_2_ (0.5–2.0 mol%), KO^*t*^Bu (0.1 mmol, 10 mol%) and **5** (1.0 mmol) in anhydrous Et_2_O (2 mL), and the reaction mixture was stirred under N_2_ at 30 °C for 4.0–24.0 h. Isolated yield of the corresponding ammonium salt is shown.

The scope of nitriles for the dihydroboration was next explored and the ammonium salts were generally obtained in good to excellent yields (Fig. 4). The reactions of nitriles with an electron-donating group on the phenyl ring (Me, ^*t*^Bu-, Ph-, Me_2_N-, MeO-, and BnO-) afforded the corresponding products **7a**–**7j** in 80–>99% yields. Notably, in the case of *o*-tolunitrile, the methyl group did not adversely affect the catalytic reaction, affording the product **7d** in 88% yield. In addition, substrates with electron-withdrawing functional groups on the backbone (F-, Cl-, CF_3_-) were also well-tolerated, and yields of 85–>99% for **7k**–**7p** were obtained smoothly. The reaction of 2-naphthonitrile delivered the product **7q** in 97% yield. For substrate **5r** bearing a pyridyl ring, the reaction still furnished an excellent yield of **7r** (74%), attesting the notable tolerance of the catalyst to a coordinating group on the substrate. Gratifyingly, the reactions of aliphatic nitriles also proceeded smoothly, leading to the formation of the corresponding products **7s**–**7x** in good to excellent yields (64%–>99%). The hydroboration of nitrile substrates bearing another reducible functional group (formyl or acetyl) was further investigated, and both the cyano and carbonyl groups were reduced under standard conditions, giving a moderate yield of the doubly reduced product **7y** (74%) and **7z** (64%), respectively. Therefore, aromatic, heterocyclic as well as aliphatic nitriles are well compatible with this ligand-free Co catalyzed dihydroboration, showing the robustness of the protocol. A 10 mmol scale reaction of **5a** was successfully carried out in the presence of 0.2 mol% CoI_2_, and the product **7a** was isolated in 83% yield (1.19 g), demonstrating the potential practicability of this method. In addition, several one-pot transformations starting from benzonitrile were further investigated to show the synthetic utility. PhCH_2_N(BPin)_2_, produced in situ in the present hydroboration of benzonitrile with HBPin, was subjected to the reaction with benzaldehyde, affording *N*-benzylidenebenzylamine **6a-I** in 88% yield for this one-pot transformation^66^. Furthermore, a relay process of hydroboration of benzonitrile followed by the reaction with benzoic acid at 120 °C, allowed for the facile synthesis of amide **6a-II** in 80% yield without the need for an exogenous coupling reagent^65^.

### Mechanistic studies

**Fig. 5:**
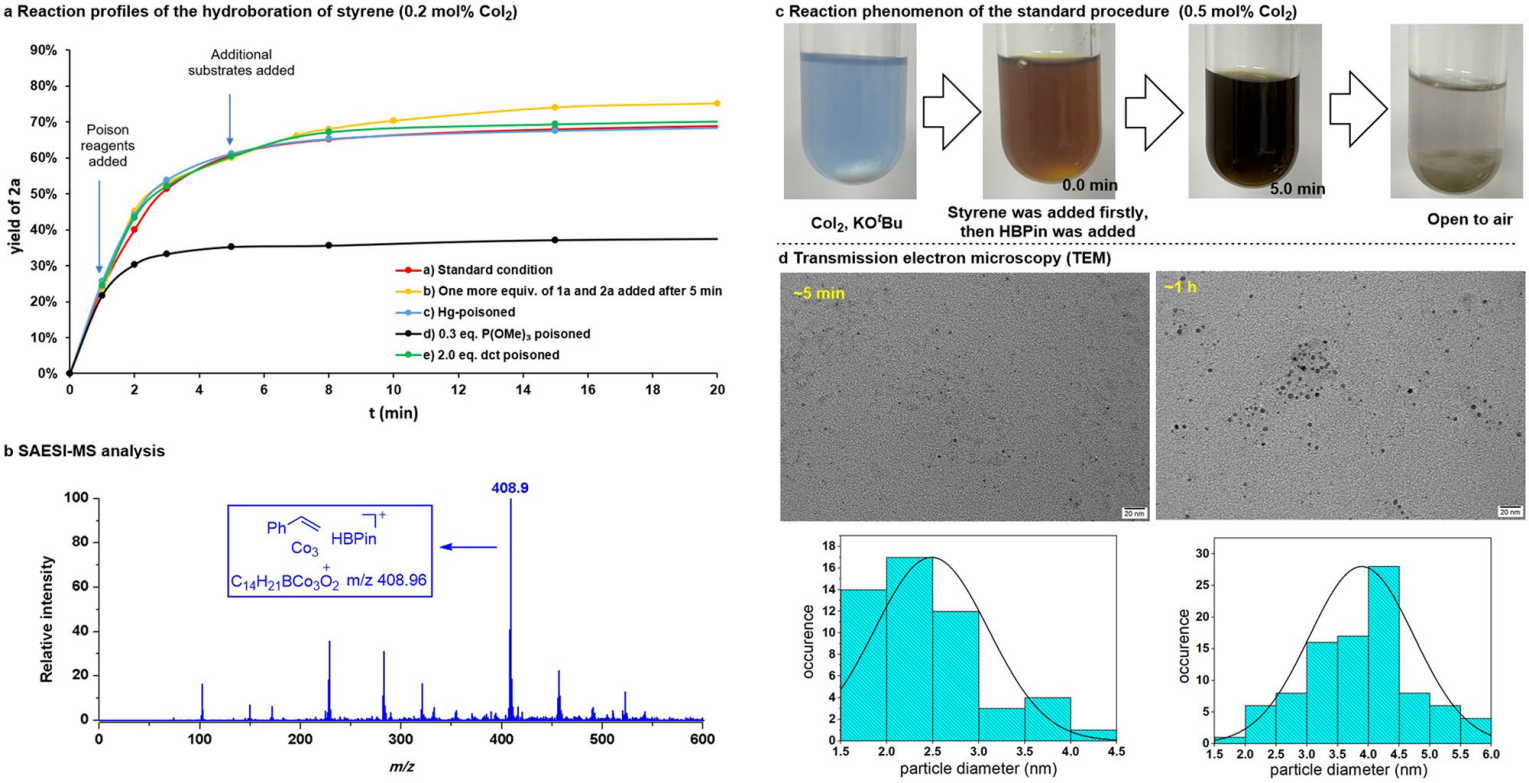
Data for preliminary mechanistic studies. **a** Reaction profiles for hydroboration of styrene with HBPin performed under different conditions. **b** SAESI-MS spectrum of the sample taken at 0.5 min under the standard reaction conditions. **c** Visual changes in color and turbidity of the mixture during the reaction course. **d** TEM images for the samples which were prepared at 5.0 min and 1.0 h.

Mechanistic studies were conducted to understand the above hydroboration reactions, specifically on the identification of the catalytically active species. Kinetic profiles for the hydroboration reaction of styrene with 0.2 mol% CoI_2_ exhibits no obvious induction period, and the yield of **2a** reached 61% in 5.0 min, showing a high reactivity during the first 5.0 min (Fig. 5a, curve a). However, the reaction rate was slowed down obviously after that point. Addition of one more equivalent of **1a** and HBPin each to the reaction system at 5.0 min resulted in only a slight increase in the yield of the product **2a**, suggesting that most of the in situ generated active catalyst deactivated in the first few minutes (Fig. 5a, curve b). Kinetic poisoning studies were further performed to ascertain the topicity of the operating catalyst species^69,70^. The addition of excess Hg to the reaction system under standard conditions at 1.0 min had almost no effect on the reaction rate (Fig. 5a, curve c). On the other hand, addition of substoichiometric trimethylphosphite [P(OMe)_3_, 0.3 equiv per Co atom] to the reaction system under standard conditions at 1.0 min led to sharp inhibition of catalytic turnover (Fig. 5a, curve d). The selective homogeneous catalyst poison dibenzo[a,e]cyclo-octatetraene (dct, 2.0 equiv per Co) showed no effect of product formation (Fig. 5a, curve e). Based on the results of these control experiments, we postulate a heterotopic mechanism that involves initial reduction of CoI_2_ by in situ generated reductant, and rapid aggregation of the resulting low-valent cobalt species to heterotopic Co catalysts as the catalytically active species^13–19^.

Solvent-assisted electrospray ionization (SAESI) mass spectrum of the reaction mixture for hydroboration of **1a**, collected after 0.5 min on the initiation of the reaction, shows an intense signal at *m*/*z* = 408.9 (Fig. 5b, calcd for C_14_H_21_BCo_3_O_2_^+^: 408.96), which matches the *m*/*z* of [HBpin-Co_3_-styrene + H]^+^ (for details, see the Supplementary Information). Although the exact nature of the assembly is not clear at current stage, it is tempting to suggest the peak might be a snapshot of the reacting system, and that the multimetallic species may be the genuine catalyst in the catalytic cycle^20–29^. However, some efforts to investigate UV/Vis measurements of the formed active species in the reaction mixture failed, which might be due to the high activity, sensitivity, and spontaneous aggregation to larger particles. Co(II)(alkoxide)_2_ was prepared from the reaction of CoI_2_ and KO^*t*^Bu in THF, and the isolated salt did not show good catalytic activity in the hydroboration of styrene, suggesting that bis-tert-butoxide cobalt salt itself is unlikely to be the catalytically active species^11^. The mix of CoI_2_ (0.5 mol%) and KO^*t*^Bu (10.0 mol%) in THF led to a nattier blue suspension (Fig. 5c). When styrene **1a** was added to the mixture, the mixture remained unchanged. In contrast, the further addition of HBPin led to a brown solution immediately, indicating that HBPin was involved in the activation of the cobalt salt and some very active cobalt species formed quickly according to the reaction profile^11,59^. After 5.0 min, the color turned to dark black, suggesting that the cobalt species may aggregate to larger inactive particles. When the reaction system was exposed to air for 30 min, the black color faded and some particles precipitated, supporting that the in situ formed cobalt species in the reaction mixture are air sensitive. Transmission electron microscopy (TEM) showed that the sample which was prepared at 5.0 min after the reaction beginning contained cobalt nanoparticles with an average particle size of 2.5 nm (Fig. 5d). It should be noted that smaller particles are hard to be identified. The average particle size was further increased to 3.9 nm at 1.0 h, supporting that the gradual aggregation of the cobalt species to Co particles with time, which is consistent with the reaction profile and phenomenon. Although the exact underlying reasons are still not clear at the present stage, it is tempting to propose that some metal–metal interactions in the heterotopic Co catalysts might account for the highly catalytic activities^20–29^.

## Discussion

In this work, we develop a highly efficient and regio-selective catalytic system for hydroboration of vinylarenes and organic nitriles with HBPin, using commercially available CoI_2_ and KO^*t*^Bu under ligand-free conditions. The alkoxide-pinacolborane combination plays a key role to activate CoI_2_ to the formation of active heterotopic Co catalysts in the hydroboration of olefins. The practical feature of this ligand-free earth-abundant metal catalysis and mechanistic understanding of this discovery may extend beyond hydroboration itself and provide a useful model for the development of earth-abundant metal catalysis in organic methodologies.

## Methods

### General procedure for styrene derivatives hydroboration

In a nitrogen-filled glovebox, CoI_2_ (0.5–2 mol%), KO^*t*^Bu (11.2 mg, 0.1 mmol, 10 mol%), anhydrous THF (5 mL), and olefin **1** (1.0 mmol) were added to a 10 mL vial equipped with a magnetic stir bar. HBPin (153.6 mg, 174 µL, 1.2 mmol) was then added to the stirring mixture. The reaction mixture was stirred vigorously at 30 °C for 1.0 h and then was quenched by exposing the solution to air. The resulting solution was concentrated in vacuum and the residue was purified by chromatography on silica gel eluting with petroleum ether/Et_2_O to give the product. The regioselectivity was determined by ^1^H-NMR of the crude product.

### General procedure for nitrile hydroboration

In a nitrogen-filled glovebox, CoI_2_ (0.5–2 mol%), KO^*t*^Bu (11.2 mg, 0.1 mmol, 10.0 mol%), anhydrous Et_2_O (2 mL, 0.5 M), and nitrile **5** (1 mmol) were added to a 10 mL vial equipped with a magnetic stir bar. HBPin (281.6 mg, 319 µL, 2.2 mmol, 2.2 eq.) was then added to the stirring mixture. The reaction mixture was stirred vigorously at 30 °C for 4 h and then was filtered through a pad of celite. The residue was washed with Et_2_O until no product remained on the celite. HCl (0.6 M in Et_2_O, 4 mL, 2.4 mmol) was added to the filtrate, affording amines as hydrochloride salts. The resulting suspension was stirred for 1 h and then filtered through a pad of celite. The residue on the celite was washed with MeOH to redissolve the product. After that, volatiles were removed by a rotate evaporator. The product was purified by forming a slurry from the mixture solution of CH_2_Cl_2_ and ethyl acetate as a solid.

### Reporting summary

Further information on research design is available in the Nature Research Reporting Summary linked to this article.

## Supplementary information

Supplementary Information

Reporting Summary

## Data Availability

The data supporting the findings of this study are available within the article and its Supplementary Information file. Any further relevant data are available from the authors on request.
