# Supplementary material for: Selective hydroboration of unsaturated bonds by an easily accessible heterotopic cobalt catalyst
Source: Nat Commun. 2021 Jun 21;12:3813. doi: 10.1038/s41467-021-24117-5 (PMC8217234; doi:10.1038/s41467-021-24117-5)
Supplement: Supplementary file 1 — Supplementary Information [file 41467_2021_24117_MOESM1_ESM.pdf]

Supplementary Information

Selective Hydroboration of Unsaturated Bonds by An Easily Accessible Heterotopic Cobalt Catalyst

Chuhan Li[1], Shuo Song[1], Yuling Li[1], Chang Xu[2], Qiquan Luo[3], Yinlong Guo[1*], Xiaoming Wang[1,4*]

[1] State Key Laboratory of Organometallic Chemistry, Center for Excellence in Molecular Synthesis, Shanghai Institute of Organic Chemistry, Chinese Academy of Sciences, 345 Lingling Road, Shanghai 200032 (China)
[2] Department of Chemistry, Anhui University, Hefei, Anhui 230601 (China)
[3] Institutes of Physical Science and Information Technology, Anhui University, Hefei, Anhui 230601 (China)
[4] School of Chemistry and Materials Science, Hangzhou Institute for Advanced Study, University of Chinese Academy of Sciences, 1 Sub-lane Xiangshan, Hangzhou 310024 (China)

*Correspondence author. Email: ylguo@sioc.ac.cn; xiaoming@sioc.ac.cn

Table of Content

1. General information ... 3

2. Optimization of reaction conditions ... 4

3. Procedure for hydroboration of alkenes ... 7

4. Procedure for hydroboration of nitriles ... 12

5. Transformations from styrene by a one-pot process ... 18

6. Transformations from benzonitrile by a one-pot process ... 19

7. Mechanism studies ... 20

7.1 ICP-MS Analyses for Trace Metal Contaminants ... 20

7.2 Kinetic & poisoning studies ... 21

7.3 TEM Analysis ... 23

7.4 SAESI-MS Analysis ... 25

7.5 Radical-trapping experiments ... 26

7.6 The effect of the addition of crown ethers ... 27

7.7 Other borane sources used in this cobalt catalytic system ... 27

7.8 Co(II)(alkoxide)$_2$ salt as the potential pre-catalyst ... 28

7.9 ^{11}B NMR of the reaction mixture ... 29

8. NMR Spectra ... 30

9. Supplementary References ... 96

1. General information

The reactions were performed in oven-dried glassware under a nitrogen atmosphere, unless otherwise noted. Dry solvents were bought and stored over molecular sieves in a nitrogen atmosphere. Tetrahydrofuran (THF) was distilled from sodium/benzophenone prior to use. The following chemicals were purchased, stored in a glovebox (N_2) and used as received: CoI_2 (>95%, >99.5% Co, Strem), KO^{t}Bu (99.99% trace metals basis, Aldrich), Pinacolborane (97%, TCI). All alkenes, except for **1f**, **1j**, **1m** and **1n** with high boiling points, were vacuum distilled from CaH_2 prior to use. All nitirles, except for **5e-j**, **5k**, **5n**, **5o, 5q, 5r** and **5x-z** with high boiling points, were vacuum distilled from CaH_2 prior to use. Nitrile **5k** was synthesized according to the literature.[1] All other reagents and solvents mentioned in this text were purchased from commercial sources and used as received.

The analytical thin layer chromatography (TLC) was performed on HSGF/UV254 plates. The flash chromatography was performed on Huanghai silica gel (200-300 mesh) by standard techniques eluting with solvents as indicated.

On a Varian Mercury (400 MHz), Bruker (400 MHz) or Agilent (400 MHz or 600 MHz), the ^{1}H, ^{11}B, ^{13}C, ^{19}F and ^{29}Si-NMR spectra were recorded in solvents as indicated. Chemical shifts (δ) are given in ppm relative to TMS. The residual solvent signals were used as references and the chemical shifts converted to the TMS scale ($CDCl_3$: δH = 7.26 ppm, δC = 77.16 ppm).

GC analysis was performed on Shimadzu GC-2014 gas chromatograph. High resolution mass spectrometer (HR-MS) were carried out on JMS-T100LP AccuTOF LC-plus 4G by the Analytical Laboratory of Shanghai Institute of Organic Chemistry (CAS).

Elemental analyses were performed on a Elementar vario EL *III* CHN instrument and Leeman High Dispersion ICP by the Analytical Laboratory of Shanghai Institute of Organic Chemistry (CAS).

2. Optimization of reaction conditions

Supplementary Table 1. Hydroboration of styrene with different solvents

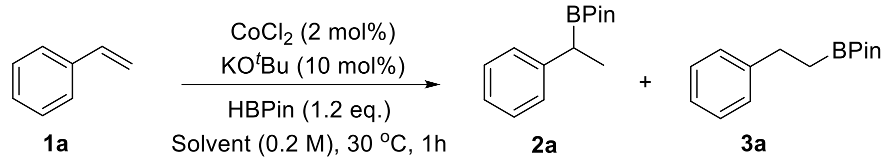

Entry	Solvent	Conv. (%)^{b}	Yield (**2a**+**3a**) (%)^{b}	**2a/3a**^{b}
1	THF	>99	83	>50/1
2	Et_2O	>99	63	0.4/1
3	Toluene	5	trace	-
4	Benzene	n.r.	n.r.	-
5	Hexane	n.r.	n.r.	-
6	Cyclohexane	n.r.	n.r.	-
7	Dioxane	95	38	1.8/1

^{a}Reaction conditions: HBPin (0.48 mmol) was added to a mixture of $CoCl_2$ (2 mol%), KO^{t}Bu (10 mol%) and styrene (0.4 mmol) in solvents (2 mL) and stirred at 30 °C for 1.0 h. ^{b}Determined by GC.

Supplementary Table 2. Hydroboration of styrene with different pre-catalysts

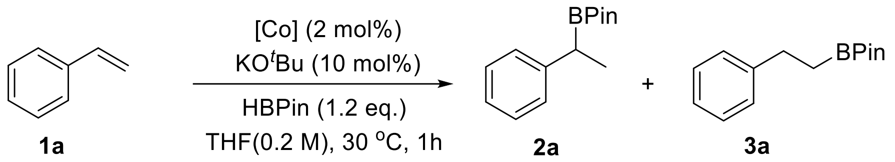

Entry	pre-catalysts	Conv. (%)^{b}	Yield (**2a**+**3a**) (%)^{b}	**2a/3a**^{b}
1	CoI_2	>99	92	48.7/1
2	$CoBr_2$	>99	84	33.0/1
3	$CoCl_2$	>99	81	35.6/1
4	CoF_2	6	6	9.5/1
5	$Co(OAc)_2$	98	78	9.6/1
6	$Co(acac)_2$	>99	81	10.0/1
7	$Co(acac)_3$	>99	87	34.8/1
8	$Co(NH_3)_6Cl_3$	90	76	13.1/1
9	Co_2CO_3	16	3	10.1/1
10	CoF_3	23	12	19.1/1

^{a}Reaction conditions: HBPin (0.48 mmol) was added to a mixture of pre-catalysts (2 mol%), KO^{t}Bu (10 mol%) and styrene (0.4 mmol) in THF (2 mL) and stirred at 30 °C for 1.0 h. ^{b}Determined by GC.

Supplementary Table 3. Hydroboration of styrene with different additives

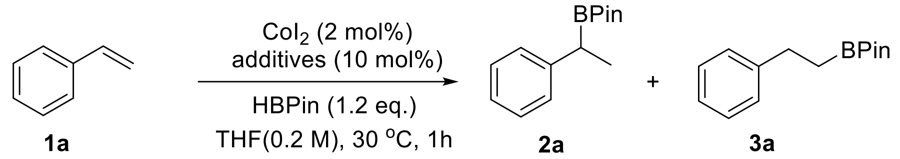

Entry	Additive	Yield (**2a**+**3a**) (%)[b]	**2a/3a**[b]
1	-	n.r.	-
2	KO^{t}Bu	87	>50/1
3	NaO^{t}Bu	84	39.2/1
4	LiO^{t}Bu	82	7.1/1
5	KOH	4	5.0/1
6	KOAc	n.r.	-
7	KHMDS	80	8.9/1
8	KOEt	45	0.4/1
9	CsF	n.r.	-
10	Cs_2CO_3	n.r.	-
11	K_2CO_3	n.r.	-
12	Na_2CO_3	n.r.	-
13	Li_2CO_3	n.r.	-
14	$NaHBEt_3$	5	5.3/1
15	PhLi	n.r.	-
16	EtMgBr	n.r.	-
17	$KHBEt_3$[c]	3	1.5/1
18	$NaHBEt_3$[c]	30	0.9/1
19	$LiHBEt_3$[c]	26	1.1/1
20	MeLi[c]	11	0.6/1
21	PhLi[c]	61	0.7/1
22	EtMgBr[c]	47	0.4/1

[a]Reaction conditions: HBPin (0.48 mmol) was added to a mixture of CoI_2 (2 mol%), additives (10 mol%) and styrene (0.4 mmol) in THF (2 mL) and stirred at 30 °C for 1.0 h. [b]Determined by GC. [c]Additives were added at last.

Supplementary Table 4. Hydroboration of styrene with different amount of catalyst

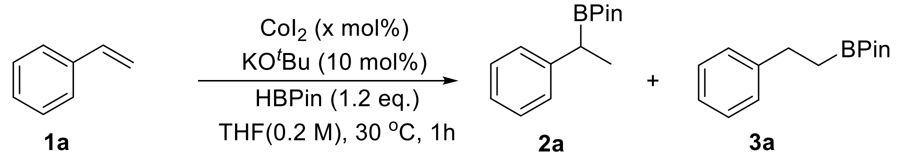

Entry	CoI_2 (mol%)	Conv. (%)[b]	Yield (**2a**+**3a**) (%)[b]	**2a/3a**[b]
1	2.0	>99	85	46.7/1
2	1.0	>99	91	>50/1

3	0.5	>99	88	>50/1
4	0.2	>99	89	24.2/1
5	0.1	88	74	16.4/1

[a]Reaction conditions: HBPin (0.48 mmol) was added to a mixture of CoI_2 (x mol%), KO^tBu (10 mol%) and styrene (0.4 mmol) in THF (2 mL) and stirred at 30 °C for 1.0 h. [b]Determined by GC.

Supplementary Table 5. Hydroboration of styrene with different amount of additive

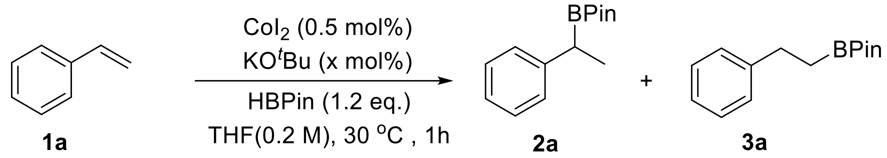

Entry	KO^tBu (mol%)	Yield (**2a**+**3a**) (%)[b]	**2a/3a**[b]
1	20	86	46.0/1
2	15	88	>50/1
3	10	88	>50/1
4	8	90	>50/1
5	6	90	>50/1
6	4	88	>50/1
7	2	88	9.2/1
8	1	n.r.	-

[a]Reaction conditions: HBPin (0.48 mmol) was added to a mixture of CoI_2 (0.5 mol%), KO^tBu (x mol%) and styrene (0.4 mmol) in THF (2 mL) and stirred at 30 °C for 1.0 h. [b]Determined by GC.

Supplementary Table 6. Hydroboration of styrene in different amount of solvent

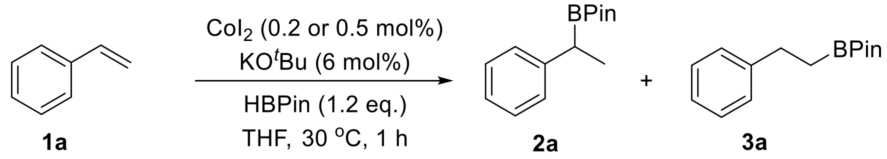

Entry	CoI_2 (mol%)	*c* (**1a**) (mol/L)	Conv. (%)[b]	Yield (**2a**+**3a**) (%)[b]	**2a/3a**[b]
1	0.2	1	>99	82	9.6/1
2	0.2	0.5	>99	88	18.5/1
3	0.2	0.2	97	81	15.0/1
4	0.2	0.1	95	77	16.3/1
5	0.2	0.05	82	61	10.6/1
6	0.2	neat	60	35	5.2/1
7	0.5	1	>99	86	14.8/1
8	0.5	0.5	>99	87	>50/1
9	0.5	0.2	>99	88	>50/1
10	0.5	0.1	>99	87	48.6/1

[a]Reaction conditions: HBPin (0.48 mmol) was added to a mixture of CoI_2 (0.2 or 0.5 mol%), KO^tBu (6 mol%) and styrene (0.4 mmol) in THF (x mL) and stirred at 30 °C for 1.0 h. [b]Determined by GC.

3. Procedure for hydroboration of alkenes

Representative procedure for alkene hydroboration. In a nitrogen filled glovebox, CoI_2 (x mol%), KO^tBu (11.2 mg, 0.1 mmol, 10 mol%), THF (5 mL) and olefin **1** (1.0 mmol) were added to a 10 mL vial equipped with a magnetic stir bar. HBPin (153.6 mg, 174 μL, 1.2 mmol) was then added to the mixture. The reaction mixture was stirred vigorously at 30 °C for 1.0 h and then was quenched by exposing the solution to air. The resulting solution was concentrated in vacuum and the residue was purified by chromatography on silica gel eluting with petroleum ether/Et_2O to give the product. The regioselectivity was determined by ^{1}H-NMR of the crude product.

2-(1-Phenylethyl)-4,4,5,5-tetramethyl-1,3,2-dioxaborolane (2a)[2]

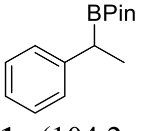

1a (104.2 mg, 1.0 mmol) and CoI_2 (1.56 mg, 0.005 mmol, 0.5 mol%) were used to give **2a** as a colorless oil (197.9 mg, 85%) with the regioselectivity of >50/1 (B/L).
^{1}H NMR (400 MHz, $CDCl_3$) δ 7.32 – 7.21 (m, 4H), 7.19 – 7.12 (m, 1H), 2.47 (q, *J* = 7.4 Hz, 1H), 1.37 (d, *J* = 7.5 Hz, 3H), 1.23 (d, *J* = 5.4 Hz, 12H) ppm.
^{13}C NMR (101 MHz, $CDCl_3$) δ 145.0, 128.4, 127.9, 125.2, 83.3, 24.70, 24.66, 17.1 ppm.
^{11}B NMR (128 MHz, $CDCl_3$) δ 33.67 ppm.

2-(1-(4-Methylphenyl)ethyl)-4,4,5,5-tetramethyl-1,3,2-dioxaborolane (2b)[3]

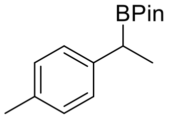

1b (118.2 mg, 1.0 mmol) and CoI_2 (1.56 mg, 0.005 mmol, 0.5 mol%) were used to give **2b** as a colorless oil (228.1 mg, 93%) with the regioselectivity of 42/1 (B/L).
^{1}H NMR (400 MHz, $CDCl_3$) δ 7.20 – 7.03 (m, 4H), 2.43 (q, *J* = 7.5 Hz, 1H), 2.34 (s, 3H), 1.35 (d, *J* = 7.5 Hz, 3H), 1.24 (d, *J* = 5.1 Hz, 12H) ppm.
^{13}C NMR (101 MHz, $CDCl_3$) δ 142.0, 134.4, 129.1, 127.7, 83.3, 24.71, 24.68, 21.1, 17.4 ppm.
^{11}B NMR (128 MHz, $CDCl_3$) δ 33.70 ppm.

2-(1-(3-Methylphenyl)ethyl)-4,4,5,5-tetramethyl-1,3,2-dioxaborolane (2c)[2]

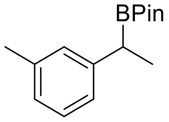

1c (118.2 mg, 1.0 mmol) and CoI_2 (1.56 mg, 0.005 mmol, 0.5 mol%) were used to give **2c** as a colorless oil (224.0 mg, 91%) with the regioselectivity of 43/1 (B/L).
^{1}H NMR (400 MHz, $CDCl_3$) δ 7.25 – 7.17 (m, 1H), 7.13 – 7.06 (m, 2H), 7.04 – 6.97 (m, 1H), 2.47 (q, *J* = 7.5 Hz, 1H), 2.38 (s, 3H), 1.40 (d, *J* = 7.4 Hz, 3H), 1.27 (d, *J* = 5.5 Hz, 12H) ppm.
^{13}C NMR (101 MHz, $CDCl_3$) δ 145.0, 137.8, 128.7, 128.3, 126.0, 124.9, 83.3, 24.72, 24.69, 21.6, 17.3 ppm.
^{11}B NMR (128 MHz, $CDCl_3$) δ 33.65 ppm.

2-(1-(4-tert-Butylphenyl)ethyl)-4,4,5,5-tetramethyl-1,3,2-dioxaborolane (2d)[2]

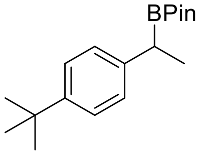

1d (160.3 mg, 1.0 mmol) and CoI_2 (1.56 mg, 0.005 mmol, 0.5 mol%) were used to give **2d** as a colorless oil (252.1 mg, 88%) with the regioselectivity of 18/1 (B/L).
^{1}H NMR (400 MHz, $CDCl_3$) δ 7.33 (d, *J* = 8.4 Hz, 2H), 7.20 (d, *J* = 8.3 Hz, 2H), 2.46 (q, *J* = 7.5 Hz, 1H), 1.41 – 1.31 (m, 12H), 1.26 (d, *J* = 5.0 Hz, 12H) ppm.
^{13}C NMR (101 MHz, $CDCl_3$) δ 147.6, 141.7, 127.4, 125.2, 83.2, 34.3, 31.5, 24.7, 24.7, 17.3 ppm.
^{11}B NMR (128 MHz, $CDCl_3$) δ 33.46 ppm.

2-(1-(2-Methylphenyl)ethyl)-4,4,5,5-tetramethyl-1,3,2-dioxaborolane (2e) and 2-(4-methylphenethyl)-4,4,5,5-tetramethyl-1,3,2-dioxaborolane (3e)[3]

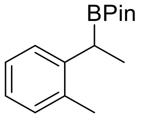

1e (118.2 mg, 1.0 mmol) and CoI_2 (1.56 mg, 0.005 mmol, 0.5 mol%) were used to give the mixture of **2e** and **3e** as a colorless oil (210.4 mg, 85%) with the regioselectivity of 0.8:1 (B/L).
For 2e:
^{1}H NMR (400 MHz, $CDCl_3$) δ 7.34 – 7.05 (m, 4H), 2.65 (q, *J* = 7.5 Hz, 1H) 2.38 (s, 3H), 1.39 (d, *J* = 7.5 Hz, 3H), 1.32 – 1.25 (m, 12H) ppm.
^{13}C NMR (101 MHz, $CDCl_3$) δ 143.5, 135.7, 130.1, 127.2, 126.2, 125.1, 83.3, 24.8, 24.7, 20.0, 16.5 ppm.
^{11}B NMR (128 MHz, $CDCl_3$) δ 33.91 ppm.
For 3e:
^{1}H NMR (400 MHz, $CDCl_3$) δ 7.34 – 7.05 (m, 4H), 2.79 (t, *J* = 8.2 Hz, 2H), 2.38 (s, 3H), 1.32 – 1.25 (m, 12H), 1.17 (t, *J* = 8.2 Hz, 2H) ppm.
^{13}C NMR (101 MHz, $CDCl_3$) δ 142.6, 135.8, 130.1, 128.2, 126.0, 125.7, 83.2, 27.3, 24.9, 19.4 ppm.
^{11}B NMR (128 MHz, $CDCl_3$) δ 33.91 ppm.

2-(1-(1,1'-Biphenyl)-4-ylethyl)-4,4,5,5-tetramethyl-1,3,2-dioxaborolane (2f)[2]

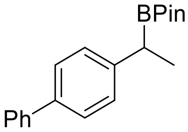

1f (180.3 mg, 1.0 mmol) and CoI_2 (3.13 mg, 0.01 mmol, 1.0 mol%) were used to give **2f** as a white solid (279.2 mg, 91%) with the regioselectivity of 18:1 (B/L). **Mp** 70-72 °C
^{1}H NMR (400 MHz, $CDCl_3$) δ 7.65 – 7.57 (m, 2H), 7.56 – 7.50 (m, 2H), 7.48 – 7.40 (m, 2H), 7.37 – 7.29 (m, 3H), 2.52 (q, *J* = 7.5 Hz, 1H), 1.40 (d, *J* = 7.5 Hz, 3H), 1.25 (d, *J* = 4.8 Hz, 12H) ppm.
^{13}C NMR (101 MHz, $CDCl_3$) δ 144.2, 141.2, 137.9, 128.6, 128.2, 127.0, 126.9, 126.8, 83.4, 24.64, 24.61, 17.1 ppm.
^{11}B NMR (128 MHz, $CDCl_3$) δ 33.61 ppm.

2-(1-(4-Fluorophenyl)ethyl)-4,4,5,5-tetramethyl-1,3,2-dioxaborolane (2g)[2]

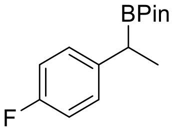

1g (122.1 mg, 1.0 mmol) and CoI_2 (1.56 mg, 0.005 mmol, 0.5 mol%) were used to give **2g** as a colorless oil (239.0 mg, 96%) with the regioselectivity of 31:1 (B/L).
^{1}H NMR (400 MHz, $CDCl_3$) δ 7.22 – 7.14 (m, 2H), 7.00 – 6.91 (m, 2H), 2.43 (q, *J* = 7.5 Hz, 1H), 1.33 (d, *J* = 7.6 Hz, 3H), 1.22 (d, *J* = 4.8 Hz, 12H) ppm.
^{13}C NMR (101 MHz, $CDCl_3$) δ 162.0, 159.6, 140.50, 140.47, 129.0, 128.9, 115.0, 114.8, 83.3, 24.6, 24.5, 17.2 ppm.
^{19}F NMR (376 MHz, $CDCl_3$) δ -118.96 ppm.
^{11}B NMR (128 MHz, $CDCl_3$) δ 33.62 ppm.

2-(1-(3-Fluorophenyl)ethyl)-4,4,5,5-tetramethyl-1,3,2-dioxaborolane (2h)[2]

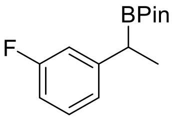

1h (122.1 mg, 1.0 mmol) and CoI_2 (1.56 mg, 0.005 mmol, 0.5 mol%) were used to give **2h** as a colorless oil (210.0 mg, 84%) with the regioselectivity of 46:1 (B/L).
^{1}H NMR (400 MHz, $CDCl_3$) δ 7.28 – 7.16 (m, 1H), 7.06 – 6.91 (m, 2H), 6.88 – 6.79 (m, 1H), 2.47 (q, *J* = 7.1 Hz, 1H), 1.35 (d, *J* = 7.5 Hz, 3H), 1.23 (d, *J* = 4.5 Hz, 12H) ppm.
^{13}C NMR (101 MHz, $CDCl_3$) δ 164.2, 161.7, 147.7, 147.6, 129.54, 129.46, 123.44, 123.41, 114.6, 114.4, 112.0, 111.8, 83.4, 24.8, 24.6, 24.5, 16.7 ppm.
^{19}F NMR (376 MHz, $CDCl_3$) δ -113.97 ppm.
^{11}B NMR (128 MHz, $CDCl_3$) δ 33.59 ppm.

2-(1-(3-Trifluromethylphenyl)ethyl)-4,4,5,5-tetramethyl-1,3,2-dioxaborolane (2i)[4]

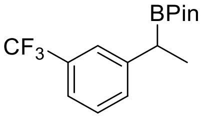

1i (172.2 mg, 1.0 mmol) and CoI_2 (6.26 mg, 0.02 mmol, 2.0 mol%) were used to give **2i** as a colorless oil (258.8mg, 86%) with the regioselectivity of 23/1 (B/L).
^{1}H NMR (400 MHz, $CDCl_3$) δ 7.52 – 7.46 (m, 1H), 7.44 – 7.33 (m, 3H), 2.51 (q, *J* = 7.5 Hz, 1H), 1.36 (d, *J* = 7.5 Hz, 3H), 1.22 (d, *J* = 4.0 Hz, 12H) ppm.
^{13}C {^{19}F} NMR (151 MHz, $CDCl_3$) δ 146.1, 131.3, 130.6, 128.7, 124.6, 124.5, 122.0, 83.6, 24.6, 24.6, 16.9 ppm.
^{19}F NMR (376 MHz, $CDCl_3$) δ -62.58 ppm.
^{11}B NMR (128 MHz, $CDCl_3$) δ 33.55 ppm.

2-(1-(4-Trimethylsilylphenyl)ethyl)-4,4,5,5-tetramethyl-1,3,2-dioxaborolane (2j)[5]

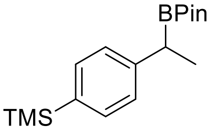

1j (176.3 mg, 1.0 mmol) and CoI_2 (1.56 mg, 0.005 mmol, 0.5 mol%) were used to give **2j** as a colorless oil (211.5 mg, 70%) with the regioselectivity of 9/1 (B/L).
^{1}H NMR (400 MHz, $CDCl_3$) δ 7.47 (d, *J* = 8.1 Hz, 2H), 7.26 (d, *J* = 7.9 Hz, 2H), 2.47 (q, *J* = 7.5 Hz, 1H), 1.38 (d, *J* = 7.5 Hz, 3H), 1.26 (d, *J* = 4.6 Hz, 12H), 0.29 (s, 9H) ppm.
^{13}C NMR (101 MHz, $CDCl_3$) δ 145.6, 136.2, 133.4, 127.3, 83.3, 24.7, 24.6, 17.1, -1.0 ppm.
^{11}B NMR (128 MHz, $CDCl_3$) δ 33.65 ppm.
^{29}Si NMR (119 MHz, $CDCl_3$) δ -4.65 ppm.

2-(1-(4-Methoxylphenyl)ethyl)-4,4,5,5-tetramethyl-1,3,2-dioxaborolane (2k)[2]

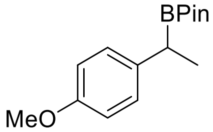

1k (134.2 mg, 1.0 mmol) and CoI_2 (1.56 mg, 0.005 mmol, 0.5 mol%) were used to give **2k** as a colorless oil (219.6 mg, 84%) with the regioselectivity of 17/1 (B/L).
^{1}H NMR (400 MHz, $CDCl_3$) δ 7.15 (d, *J* = 8.6 Hz, 2H), 6.83 (d, *J* = 8.6 Hz, 2H), 3.78 (s, 3H), 2.39 (q, *J* = 7.5 Hz, 1H), 1.32 (d, *J* = 7.5 Hz, 3H), 1.22 (d, *J* = 5.1 Hz, 12H) ppm.
^{13}C NMR (101 MHz, $CDCl_3$) δ 157.3, 137.1, 128.7, 113.8, 83.3, 55.2, 24.7, 24.7, 17.4 ppm.
^{11}B NMR (128 MHz, $CDCl_3$) δ 33.63 ppm.

2-(1-(3-Methoxylphenyl)ethyl)-4,4,5,5-tetramethyl-1,3,2-dioxaborolane (2l)[5]

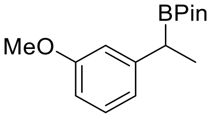

1l (134.2 mg, 1.0 mmol) and CoI_2 (3.13 mg, 0.01 mmol, 1.0 mol%) were used to give **2l** as a colorless oil (246.4 mg, 94%) with the regioselectivity of 26/1 (B/L).
^{1}H NMR (400 MHz, $CDCl_3$) δ 7.22 – 7.14 (m, 1H), 6.86 – 6.77 (m, 2H), 6.73 – 6.65 (m, 1H), 3.79 (s, 3H), 2.43 (q, J = 7.5 Hz, 1H), 1.33 (d, J = 7.5 Hz, 3H), 1.22 (d, J = 5.1 Hz, 12H) ppm.
^{13}C NMR (101 MHz, $CDCl_3$) δ 159.6, 146.6, 129.2, 120.3, 113.5, 110.5, 83.3, 55.0, 24.60, 24.58, 17.0 ppm.
^{11}B NMR (128 MHz, $CDCl_3$) δ 33.43 ppm.

tert-butyldimethyl(4-(1-(4,4,5,5-tetramethyl-1,3,2-dioxaborolan-2-yl)ethyl)phenoxy)silane (2m)[4]

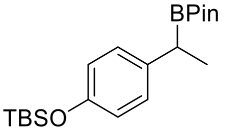

1m (234.4 mg, 1.0 mmol) and CoI_2 (1.56 mg, 0.005 mmol, 0.5 mol%) were used to give **2m** as a colorless oil (321.7 mg, 89%) with the regioselectivity of 16/1 (B/L).
^{1}H NMR (400 MHz, $CDCl_3$) δ 7.07 (d, J = 8.6 Hz, 2H), 6.74 (d, J = 8.5 Hz, 2H), 2.37 (q, J = 7.5 Hz, 1H), 1.30 (d, J = 7.6 Hz, 3H), 1.20 (d, J = 5.8 Hz, 12H), 0.98 (s, 9H), 0.19 (s, 6H) ppm.
^{13}C NMR (101 MHz, $CDCl_3$) δ 153.0, 137.4, 128.5, 119.8, 83.1, 25.7, 24.6, 24.5, 18.2, 17.1, -4.4 ppm.
^{11}B NMR (128 MHz, $CDCl_3$) δ 33.49 ppm.
^{29}Si NMR (119 MHz, $CDCl_3$) δ 19.99 ppm.

2-(1-(naphthalene-2-yl))-4,4,5,5-tetramethyl-1,3,2-dioxaborolane (2n)[3]

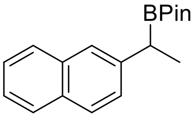

1n (154.2 mg, 1.0 mmol) and CoI_2 (6.26 mg, 0.02 mmol, 2.0 mol%) were used to give **2n** as a white solid (273.2 mg, 97%) with the regioselectivity of 12:1 (B/L). **Mp** 62-63 °C
^{1}H NMR (400 MHz, $CDCl_3$) δ 7.85 – 7.75 (m, 3H), 7.71 – 7.66 (m, 1H), 7.51 – 7.37 (m, 3H), 2.66 (q, J = 7.5 Hz, 1H), 1.48 (d, J = 7.4 Hz, 3H), 1.24 (d, J = 6.2 Hz, 12H) ppm.
^{13}C NMR (101 MHz, $CDCl_3$) δ 142.7, 134.0, 131.8, 127.8, 127.60, 127.55, 127.3, 125.7, 125.4, 124.9, 83.5, 24.7, 24.7, 16.9 ppm.
^{11}B NMR (128 MHz, $CDCl_3$) δ 33.56 ppm.

4,4,5,5-tetramethyl-2-octyl-[1,3,2]dioxaborolane (3o)[5]

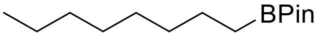

1o (112.2 mg, 1.0 mmol) and CoI_2 (6.26 mg, 0.02 mmol, 2.0 mol%) were used to give **3o** as a colorless oil (57.6 mg, 24%).
^{1}H NMR (400 MHz, $CDCl_3$) δ 1.39 (m, 2H), 1.24 (m, 22H), 0.87 (m, 3H), 0.76 (t, J = 7.8 Hz, 2H) ppm.
^{13}C NMR (101 MHz, $CDCl_3$) δ 82.9, 32.6, 32.0, 29.5, 29.4, 24.9, 24.1, 22.8, 14.2 ppm.
^{11}B NMR (128 MHz, $CDCl_3$) δ 34.17 ppm.

Unsuccessful substrates

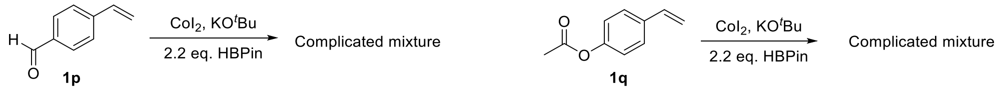

In a nitrogen filled glovebox, CoI_2 (6.26 mg, 0.02 mmol, 2.0 mol%), KO^{t}Bu (11.2 mg, 0.1 mmol, 10 mol%), THF (5 mL) and olefin **1m** or **1q** (1.0 mmol) were added to a 10 mL vial equipped with a magnetic stir bar. HBPin (281.6 mg, 319 μL, 2.2 mmol) was then added to the mixture. The reaction mixture was stirred at 30 ºC for 1.0 h and then was quenched by exposing the solution to air. The crude ^{1}H-NMR showed that the products were complicated mixtures.

4. Procedure for hydroboration of nitriles

General Procedure A (GP A) for nitrile hydroboration. In a nitrogen filled glovebox, CoI_2 (1.56 mg, 0.005 mmol, 0.5 mol%), KO^{t}Bu (11.2 mg, 0.1 mmol, 10.0 mol%), Et_2O (2 mL, 0.5 M) and nitrile **5** (1 mmol) were added to a 10 mL vial equipped with a magnetic stir bar. HBPin (281.6 mg, 319 μL, 2.2 mmol, 2.2 eq.) was then added to the mixture. The reaction mixture was stirred vigorously at 30 °C for 4 h and then was filtered through a pad of celite. The residue was washed with Et_2O until no product remained on the celite. HCl (0.6M in Et_2O, 4 mL, 2.4 mmol) was added to the filtrate, affording amines as hydrochloride salts. The resulting suspension was stirred for 1 h and then filtered through a pad of celite. The residue on the celite was washed with MeOH to redissolve the product. After that, volatiles were removed by a rotate evaporator. The product was purified by forming a slurry from the mixture solution of CH_2Cl_2 and ethyl acetate as a solid.

General Procedure B (GP B) for nitrile hydroboration. In a nitrogen filled glovebox, CoI_2 (6.26 mg, 0.02 mmol, 2.0 mol%), KO^{t}Bu (11.2 mg, 0.1 mmol, 10.0 mol%), Et_2O (2 mL, 0.5 M) and nitrile **5** (1 mmol) were added to a 10 mL vial equipped with a magnetic stir bar. HBPin (281.6 mg, 319 μL, 2.2 mmol, 2.2 eq.) was then added to the mixture. The reaction mixture was stirred vigorously at 30 °C for 24 h and then was filtered through a pad of celite. The residue was washed with Et_2O until no product remained on the celite. HCl (0.6M in Et_2O, 4 mL, 2.4 mmol) was added to the filtrate, affording the amines as hydrochloride salts. The resulting suspension was stirred for 1 h and then filtered through a pad of celite. The residue on the celite was washed with MeOH to redissolve the product. After that, volatiles were removed by a rotate evaporator. The product was purified by forming a slurry from the mixture solution of CH_2Cl_2 and ethyl acetate as a solid.

General Procedure C (GP C) for nitrile hydroboration. In a nitrogen filled glovebox, CoI_2 (6.26 mg, 0.02 mmol, 2 mol%), KO^{t}Bu (11.2 mg, 0.1 mmol, 10.0 mol%), Et_2O (2 mL, 0.5 M) and nitrile **5** (1 mmol) were added to a 10 mL vial equipped with a magnetic stir bar. HBPin (281.6 mg, 319 μL, 2.2 mmol) was then added to the mixture. The reaction mixture was stirred vigorously at 30 °C for 24 h, diluted with 5 mL toluene, and then was filtered through a pad of celite. The residue was washed with Et_2O until no product remained on the celite. HCl (0.6M in Et_2O, 4 mL, 2.4 mmol) was added to the resulting solution, affording the amines as hydrochloride salts. The resulting suspension was stirred for 1 h and then filtered through a pad of celite. The residue on the celite was washed with MeOH to redissolve the product. After that, volatiles were removed by a rotate evaporator. The crude product was purified by forming a slurry from the mixture solution of CH_2Cl_2 and ethyl acetate.Trace cobalt salts were removed by the recrystallization from appropriate solvents listed below.

Benzylamine hydrochloride (7a)[6]

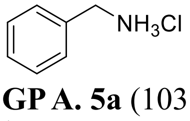

GP A. 5a (103.1 mg, 1.0 mmol) was used to give **7a** as a white solid (141.4 mg, 98%). **Mp** 238-239 °C
^{1}H NMR (400 MHz, DMSO-d_6) δ 8.38 (brs, 3H), 7.63 – 7.17 (m, 5H), 4.02 (s, 2H) ppm.
^{13}C NMR (101 MHz, DMSO-d_6) δ 134.6, 129.4, 128.9, 128.8, 42.5 ppm.

***p*-Tolylmethanamine hydrochloride (7b)**[6]

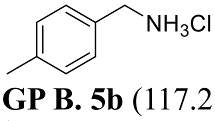

GP B. 5b (117.2 mg, 1.0 mmol) was used to give **7b** as a white solid (160.4 mg, >99%). **Mp** 220-221 °C
^{1}H NMR (400 MHz, DMSO-d_6) δ 8.25 (brs, 3H), 7.33 (d, J = 5.6 Hz, 2H), 7.20 (d, J = 5.1 Hz, 2H), 3.95 (s, 2H), 2.29 (s, 3H) ppm.
^{13}C NMR (101 MHz, DMSO-d_6) δ 138.1, 131.5, 129.4, 129.4, 42.3, 21.2 ppm.

***m*-Tolylmethanamine hydrochloride (7c)**[6]

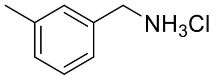

GP A. 12 h of reaction time. **5c** (117.2 mg, 1.0 mmol) was used to give **7c** as a white solid (140.8 mg, 89%). **Mp** 215-216 °C**^{1}H NMR** (400 MHz, DMSO-*d*$_6$) δ 8.48 (brs, 3H), 7.35 – 7.24 (m, 3H), 7.23 – 7.15 (m, 1H), 3.96 (s, 2H), 2.31 (s, 3H) ppm.
^{13}C NMR (101 MHz, DMSO-*d*$_6$) δ 138.1, 134.5, 130.0, 129.3, 128.9, 126.4, 42.5, 21.4 ppm.

o-Tolylmethanamine hydrochloride (7d)[6]

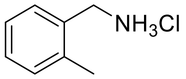

GP B. 5d (117.2 mg, 1.0 mmol) was used to give **7d** as a white solid (140.0 mg, 88%). **Mp** 224-225 °C
^{1}H NMR (400 MHz, DMSO-*d*$_6$) δ 8.29 (brs, 3H), 7.42 – 7.33 (m, 1H), 7.30 – 7.18 (m, 3H), 3.99 (s, 2H), 2.33 (s, 3H) ppm.
^{13}C NMR (101 MHz, DMSO-*d*$_6$) δ 137.1, 132.8, 130.7, 129.7, 128.8, 126.4, 39.8, 19.3 ppm.

4-(tert-Butyl)-benzylamine hydrochloride (7e)[7]

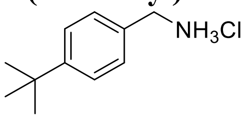

GP B. 5e (159.2 mg, 1.0 mmol) was used to give **7e** as a white solid (185.3 mg, 92%). **Mp** 258-259 °C
^{1}H NMR (400 MHz, DMSO-*d*$_6$) δ 8.30 (brs, 3H), 7.50 – 7.33 (m, 4H), 3.98 (s, 2H), 1.28 (s, 9H) ppm.
^{13}C NMR (101 MHz, DMSO-*d*$_6$) δ 151.3, 131.6, 129.2, 125.7, 42.2, 34.8, 31.5 ppm.

[1,1'-Biphenyl]-4-ylmethanamine hydrochloride (7f)[7]

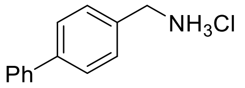

In a nitrogen filled glovebox, CoI_2 (1.56 mg, 0.005 mmol, 0.5 mol%), KO^{t}Bu (11.2 mg, 0.1 mmol), Et_2O (2 mL, 0.5 M) and nitrile **5f** (179.2 mg, 1.0 mmol) were added to a 10 mL vial equipped with a magnetic stir bar. HBPin (281.6 mg, 319 µL, 2.2 mmol) was then added to the mixture. The reaction mixture was stirred at 30 °C for 4 h, dissolved in DCM/Et_2O, and then was filtered through a pad of Celite. The residue was washed with Et_2O until no product remained on the celite. HCl solution (0.6M in Et_2O, 4 mL, 2.4 mmol) was added to the resulting solution, affording the amines as hydrochloride salts. The resulting suspension was stirred for 1 h and then filtered through a pad of celite. The residue was redissolved in MeOH and volatiles were removed by a rotate evaporator. The product was purified by forming a slurry from the mixture solution of CH_2Cl_2 and ethyl acetate.
White solid; (221.2 mg, >99%). **Mp** 278-279 °C
^{1}H NMR (400 MHz, DMSO-*d*$_6$) δ 8.41 (brs, 3H), 7.74 – 7.63 (m, 4H), 7.59 – 7.53 (m, 2H), 7.50 – 7.42 (m, 2H), 7.41 – 7.33 (m, 1H), 4.04 (s, 2H) ppm.
^{13}C NMR (101 MHz, DMSO-*d*$_6$) δ 140.6, 140.0, 133.8, 130.1, 129.4, 128.1, 127.2, 127.1, 42.2 ppm.

4-(Dimethylamino)-benzylamine monohydrochloride (7g)[8]

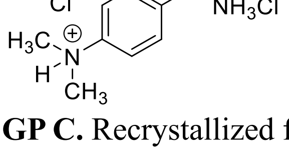

GP C. Recrystallized from MeOH/DCM. **5g** (146.2 mg, 1.0 mmol) was used to give **7g** as a yellow solid (177.6 mg, 80 %). **Mp** 195-196 °C
^{1}H NMR (400 MHz, CD_3OD) δ7.79 (d, *J* = 8.8 Hz, 2H), 7.72 (d, *J* = 8.7 Hz, 2H), 4.22 (s, 2H), 3.31 (s, 6H) ppm.
^{13}C NMR (101 MHz, CD_3OD) δ 142.0, 134.1, 129.8, 119.8, 44.4, 40.7 ppm.

4-Methoxy-benzylamine hydrochloride (7h)[6]

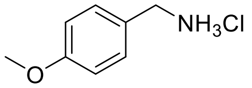

GP B. 5h (133.2 mg, 1.0 mmol) was used to give **7h** as a white solid (170.4 mg, 98%). **Mp** 232-233 ℃
^{1}H NMR (400 MHz, DMSO-d_6) δ 8.44 (brs, 3H), 7.43 (d, J = 8.8 Hz, 2H), 6.96 (d, J = 7.0 Hz, 2H), 3.92 (s, 2H), 3.75 (s, 3H) ppm.
^{13}C NMR (101 MHz, DMSO-d_6) δ 159.7, 131.0, 126.4, 114.3, 55.6, 42.0 ppm.

3-Methoxy-benzylamine hydrochloride (7i)[7]

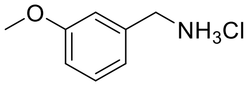

GP A. 5i (133.2 mg, 1.0 mmol) was used to give **7i** as a white solid (167.1 mg, 96%). **Mp** 160-161 °C
^{1}H NMR (400 MHz, DMSO-d_6) δ 8.27 (brs, 3H), 7.39 – 7.26 (m, 1H), 7.13 – 7.07 (m, 1H), 7.06 – 6.99 (m, 1H), 6.99 – 6.92 (m, 1H), 4.00 (s, 2H), 3.78 (s, 3H) ppm.
^{13}C NMR (101 MHz, DMSO-d_6) δ 159.7, 136.0, 130.1, 121.4, 114.9, 114.4, 55.6, 42.5 ppm.

3-Benzyloxy-benzylamine hydrochloride (7j)

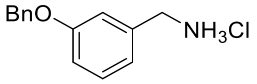

GP A. 5j (209.2 mg, 1.0 mmol) was used to give **7j** as a white solid (207.1 mg, 83%). **Mp** 159-160 °C.
^{1}H NMR (400 MHz, DMSO-d_6) δ 8.64 (brs, 3H), 7.52 – 7.21 (m, 7H), 7.10 – 7.04 (m, 1H), 7.03 – 6.96 (m, 1H), 5.11 (s, 2H), 3.97 (s, 2H) ppm.
^{13}C NMR (101 MHz, DMSO-d_6) δ 158.8, 137.3, 136.1, 130.1, 128.9, 128.3, 128.2, 121.6, 116.0, 115.0, 69.7, 42.5 ppm.
IR (neat) 3331, 3092, 3033, 2893, 1599, 1496, 1381, 732.
HRMS (ESI) m/z: $[M – Cl]^+$ calcd for $C_{14}H_{16}NO$ 214.1232; found: 214.1226.

4-Fluoro-benzylamine hydrochloride (7k)[6]

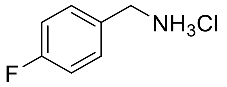

GP A. 5k (121.1 mg, 1.0 mmol) was used to give **7k** as a white solid (156.4 mg, 96%). **Mp** 251-252 °C
^{1}H NMR (400 MHz, DMSO-d_6) δ 8.27 (brs, 3H), 7.61 – 7.45 (m, 2H), 7.33 – 7.19 (m, 2H), 4.02 (s, 2H) ppm.
^{13}C {^{19}F} NMR (101 MHz, DMSO-d$_6$) δ 162.6, 131.9, 131.0, 115.8, 41.9 ppm.
^{19}F NMR (376 MHz, DMSO-d_6) δ -112.96 ppm.

3-Fluoro-benzylamine hydrochloride (7l)[9]

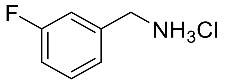

GP A. 5l (121.1 mg, 1.0 mmol) was used to give **7l** as a white solid (163.4 mg, >99%). **Mp** 270-271 °C
^{1}H NMR (400 MHz, D_2O) δ 7.56 – 7.34 (m, 1H), 7.34 – 6.99 (m, 3H), 4.16 (s, 2H) ppm.
^{13}C NMR (151 MHz, D_2O) δ 162.4, 160.8, 133.82, 133.77, 130.1, 130.0, 123.67, 123.65, 115.1, 115.0, 114.7, 114.6, 41.6 ppm.
^{19}F NMR (376 MHz, D_2O) δ-110.33 (q, J = 8.4 Hz) ppm.

2-Fluoro-benzylamine hydrochloride (7m)[9]

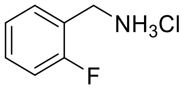

GP A. 5m (121.1 mg, 1.0 mmol) was used to give **7m** as a white solid (141.5 mg, 87%). **Mp** 167-168 ºC
^{1}H NMR (400 MHz, D_2O) δ 7.54 – 7.37 (m, 2H), 7.32 – 7.14 (m, 2H), 4.23 (s, 2H) ppm.
^{13}C NMR (151 MHz, D_2O) δ 160.7, 159.1, 130.8, 130.7, 130.3, 130.2, 124.0, 123.9, 118.6, 118.5, 114.8, 114.7, 36.21, 36.19 ppm.
^{19}F NMR (376 MHz, D_2O) δ -115.74 (q, J = 7.5, 7.1 Hz) ppm.

4-Chloro-benzylamine hydrochloride (7n)[7]

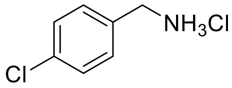

GP A. 5n (137.6 mg, 1.0 mmol) was used to give **7n** as a white solid (176.4 mg, 99%). **Mp** 236-237 ºC
^{1}H NMR (400 MHz, DMSO-d_6) δ 8.28 (brs, 3H), 7.60 – 7.41 (m, 4H), 4.03 (s, 2H) ppm.
^{13}C NMR (101 MHz, DMSO-d_6) δ 133.6, 133.5, 131.5, 128.9, 41.8 ppm.

4-Trifluoromethyl-benzylamine hydrochloride (7o)[6]

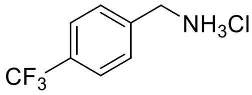

GP A. 5o (171.1 mg, 1.0 mmol) was used to give **7o** as a white solid (203.0 mg, 95%). **Mp** 240-241 ºC
^{1}H NMR (400 MHz, DMSO-d_6) δ 8.64 (brs, 3H), 7.87 – 7.62 (m, 4H), 4.12 (s, 2H) ppm.
^{13}C {^{19}F} NMR (101 MHz, DMSO-d_6) δ 139.4, 130.4, 129.4, 125.8, 124.7, 42.1 ppm.
^{19}F NMR (376 MHz, DMSO-d_6) δ -60.32 ppm.

3-Trifluoromethyl-benzylamine hydrochloride (7p)[10]

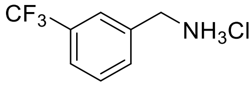

GP A. 5p (171.1 mg, 1.0 mmol) was used to give **7p** as a white solid (181.1 mg, 85%). **Mp** 165-166 ºC
^{1}H NMR (400 MHz, DMSO-d_6) δ 8.50 (brs, 3H), 7.96 – 7.88 (m, 1H), 7.85 – 7.78 (m, 1H), 7.78 – 7.72 (m, 1H), 7.70 – 7.61 (m, 1H), 4.14 (s, 2H) ppm.
^{13}C (^{19}F) NMR (101 MHz, DMSO-d_6) δ 136.1, 133.8, 130.1, 129.7, 126.4, 125.6, 124.6, 42.1 ppm.
^{19}F NMR (376 MHz, DMSO-d_6) δ -60.38 ppm.

Naphthalen-2-ylmethanamine hydrochloride (7q)[11]

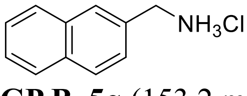

GP B. 5q (153.2 mg, 1.0 mmol) was used to give **7q** as a white solid (188.6 mg, 97%). **Mp** 265-266 ºC
^{1}H NMR (400 MHz, DMSO-d_6) δ 8.38 (brs, 3H), 8.03 – 7.82 (m, 4H), 7.67 – 7.45 (m, 3H), 4.18 (s, 2H) ppm.
^{13}C NMR (101 MHz, DMSO-d_6) δ 133.0, 132.2, 128.6, 128.4, 128.2, 128.1, 127.1 (2C), 127.0, 126.9, 42.7 ppm.

pyridin-4-ylmethanamine dihydrochloride (7r)[12]

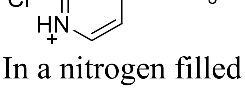

In a nitrogen filled glovebox, CoI_2 (6.26 mg, 0.02 mmol, 2 mol%), KO^{t}Bu (11.2 mg, 0.1 mmol), Et_2O (2 mL, 0.5 M) and nitrile **5r** (104.1 mg, 1.0 mmol) were added to a 10 mL vial equipped with a magnetic stir bar. HBPin (281.6 mg, 319 μL, 2.2 mmol) was then added to the mixture. The reaction mixture was stirred at 30 ºC for 24 h, dissolved in DCM/Et_2O, and then was filtered through a pad of Celite. The residue was washed with Et_2O until no product remained on the celite. HCl (0.6M in Et_2O, 4 mL, 2.4

mmol) was added to the resulting solution, affording the amines as hydrochloride salts. The resulting suspension was stirred for 1 h and then filtered. The product was purified by forming a slurry from CH_2Cl_2, following by the recrystallization from MeOH.
Yellow solid; (133.4 mg, 74%). **Mp** 238-239 °C.
^{1}H NMR (400 MHz, D_2O) δ 8.86 (d, *J* = 6.0 Hz, 2H), 8.12 (d, *J* = 6.0 Hz, 2H), 4.57 (s, 2H) ppm.
^{13}C NMR (101 MHz, D_2O) δ 153.2, 141.7, 126.2, 41.6 ppm.
IR (neat) 3052, 2971, 1638, 1594, 1503, 1392, 1198, 776.

n-Octylamine hydrochloride (7s)[6]

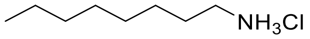

GP C. Recrystallized from DCM/EA. **5s** (125.2 mg, 1.0 mmol) was used to give **7s** as a white solid (124.1 mg, 74%). **Mp** 203-204 °C
^{1}H NMR (400 MHz, DMSO-*d*$_6$) δ 8.02 (brs, 3H), 2.71 (t, *J* = 7.7 Hz, 2H), 1.53 (p, *J* = 7.3 Hz, 2H), 1.36 – 1.11 (m, 10H), 0.84 (t, *J* = 6.5 Hz, 3H) ppm.
^{13}C NMR (101 MHz, DMSO-*d*$_6$) δ 39.1, 31.6, 29.0, 28.9, 27.3, 26.3, 22.5, 14.4 ppm.

isobutylamine hydrochloride (7t)[6]

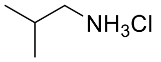

GP B. 5t (69.1 mg, 1.0 mmol) was used to give **7t** as a white solid (110.4 mg, >99%). **Mp** 160-161 °C
^{1}H NMR (400 MHz, DMSO-*d*$_6$) δ 8.05 (brs, 3H), 2.61 (d, *J* = 7.0 Hz, 2H), 1.88 (hept, *J* = 13.5, 6.8 Hz, 1H), 0.92 (d, *J* = 6.7 Hz, 6H) ppm.
^{13}C NMR (101 MHz, DMSO-*d*$_6$) δ 46.0, 26.7, 20.3 ppm.

Cyclohexanmethylamine hydrochloride (7u)[11]

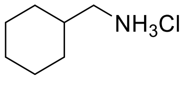

GP B. 5u (109.2 mg, 1.0 mmol) was used to give **7u** as a white solid (136.0 mg, 90%). **Mp** 261-262 °C
^{1}H NMR (400 MHz, DMSO-*d*$_6$) δ 7.99 (brs, 3H), 2.59 (d, J = 6.9 Hz, 2H), 1.78 – 1.44 (m, 6H), 1.25 – 1.01 (m, 3H), 0.96 – 0.80 (m, 2H) ppm.
^{13}C NMR (101 MHz, DMSO-*d*$_6$) δ 44.8, 35.8, 30.3, 26.1, 25.5 ppm.

2-Phenylethylamine hydrochloride (7v)[6]

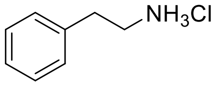

GP C. Recrystallized from DCM/EA. **5v** (117.2 mg, 1.0 mmol) was used to give **7v** as a white solid (105.1 mg, 66%). **Mp** 215-216 °C
^{1}H NMR (400 MHz, DMSO-*d*$_6$) δ 7.98 (brs, 3H), 7.35 – 7.28 (m, 2H), 7.28 – 7.20 (m, 3H), 3.07 – 2.94 (m, 2H), 2.90 – 2.81 (m, 2H) ppm.
^{13}C NMR (101 MHz, DMSO-*d*$_6$) δ 137.9, 129.1, 129.0, 127.1, 40.3, 33.3 ppm.

4-Phenylbutylamine hydrochloride (7w)[13]

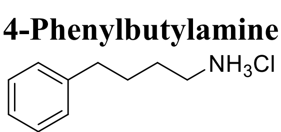

GP C. Recrystallized from DCM/EA. **5w** (145.2 mg, 1.0 mmol) was used to give **7w** as a white solid (140.6 mg, 75%). **Mp** 157-158 °C
^{1}H NMR (400 MHz, D_2O) δ 7.39 – 7.31 (m, 2H), 7.31 – 7.16 (m, 3H), 2.96 (t, *J* = 6.9 Hz, 2H), 2.65 (t, *J* = 6.6 Hz, 2H), 1.78 – 1.50 (m, 4H) ppm.
^{13}C NMR (101 MHz, D_2O) δ 142.2, 128.5, 128.5, 126.0, 39.2, 34.2, 27.6, 26.1 ppm.

3-phenoxypropylamine hydrochloride (7x)[14]

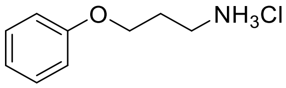

GP C. Recrystallized from DCM/EA. **5x** (147.2 mg, 1.0 mmol) was used to give **7x** as a white solid (120.7 mg, 64%). **Mp** 165-166 °C

^{1}H NMR (400 MHz, CD_3OD) δ 7.39 – 7.13 (m, 2H), 7.02 – 6.82 (m, 3H), 4.11 (t, J = 5.5 Hz, 2H), 3.16 (t, J = 7.2 Hz, 2H), 2.15 (p, J = 6.4 Hz, 2H) ppm.

^{13}C NMR (151 MHz, CD_3OD) δ 158.5, 129.1, 120.7, 114.1, 64.7, 37.3, 27.0 ppm.

[4-(amino-methyl)phenyl]methanol hydrochloride (7y)[6]

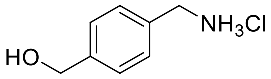

In a nitrogen filled glovebox, CoI_2 (6.26 mg, 0.02 mmol, 2.0 mol%), KO^{t}Bu (11.2 mg, 0.1 mmol), Et_2O (2 mL, 0.5 M) and nitrile **5y** (131.1 mg, 1.0 mmol) were added to a 10 mL vial equipped with a magnetic stir bar. HBPin (409.6 mg, 464 µL, 3.2 mmol) was then added to the mixture dropwise. The reaction mixture was stirred at 30 °C for 24 h, dissolved in CH_2Cl_2/Et_2O, and then was filtered through a pad of Celite. The residue was washed with Et_2O and CH_2Cl_2 until no product remained on the celite. HCl solution (0.6M in Et_2O, 4 mL, 2.4 mmol) was added to the resulting solution, affording the amines as hydrochloride salts. The resulting suspension was stirred for 1 h and then filtered through a pad of celite. The residue was redissolved in MeOH and volatiles were removed by a rotate evaporator. The product was purified by forming a slurry from the mixture solution (1 mL MeOH, 10 mL CH_2Cl_2 and 20 mL ethyl acetate) as a solid.

White solid; (128.6 mg, 74%). **Mp** 157-158 °C

^{1}H NMR (400 MHz, DMSO-d_6) δ 8.40 (brs, 3H), 7.43 (d, J = 7.8 Hz, 2H), 7.34 (d, J = 7.8 Hz, 2H), 5.28 (t, J = 5.7 Hz, 1H), 4.50 (d, J = 5.5 Hz, 2H), 3.98 (s, 2H) ppm.

^{13}C NMR (101 MHz, DMSO-d_6) δ 143.3, 132.7, 129.2, 126.9, 62.9, 42.3 ppm.

1-(4-(aminomethyl)phenyl)ethan-1-ol hydrochloride (7z)[15]

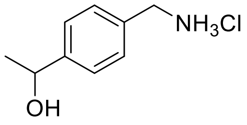

In a nitrogen filled glovebox, CoI_2 (6.26 mg, 0.02 mmol, 2.0 mol%), KO^{t}Bu (11.2 mg, 0.1 mmol), Et_2O (2 mL, 0.5 M) and nitrile **5z** (145.2 mg, 1.0 mmol) were added to a 10 mL vial equipped with a magnetic stir bar. HBPin (409.6 mg, 464 µL, 3.2 mmol) was then added to the mixture dropwise. The reaction mixture was stirred at 30 °C for 24 h, dissolved in CH_2Cl_2/Et_2O, and then was filtered through a pad of Celite. The residue was washed with Et_2O and CH_2Cl_2 until no product remained on the celite. HCl solution (0.6M in Et_2O, 4 mL, 2.4 mmol) was added to the resulting solution, affording the amines as hydrochloride salts. The resulting suspension was stirred for 1 h and then filtered through a pad of celite. The residue was redissolved in MeOH and volatiles were removed by a rotate evaporator. The product was purified by forming a slurry from the mixture solution (1 mL MeOH, 10 mL CH_2Cl_2 and 20 mL ethyl acetate) as a solid.

White solid; (120.1 mg, 64%). **Mp** 149-150 °C

^{1}H NMR (400 MHz, D_2O) δ 7.47 – 7.37 (m, 4H), 4.89 (q, J = 6.6 Hz, 1H), 4.13 (s, 2H), 1.42 (d, J = 6.6 Hz, 3H) ppm.

^{13}C NMR (101 MHz, D_2O) δ 145.8, 131.6, 129.0, 126.2, 69.3, 42.6, 23.4 ppm.

5. Transformations from styrene by a one-pot process

The synthesis of potassium (1-phenylethyl)trifluoroborate (2a-I)[16]

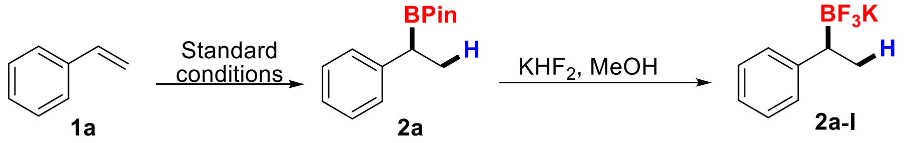

In a nitrogen filled glovebox, CoI_2 (1.56 mg, 0.5 mol%), KO^{t}Bu (11.2mg, 0.1 mmol), THF (5 mL, 0.2 M) and styrene **1a** (104 mg, 1.0 mmol) were added to a 20 mL vial equipped with a magnetic stir bar. HBPin (153.6 mg, 174 µL, 1.2 mmol) was then added to the mixture. The reaction mixture was stirred at 30 ºC for 1 h and then was quenched by exposing the solution to air. The resulting solution was concentrated in vacuum and the residue was dissolved in MeOH (5 mL). Then, KHF_2 solution (351.5 mg, 4.5 mmol, in 1 mL water) was added dropwise and the mixture was stirred at room temperature for 30 min. The solvent was then removed under vacuum and the solid residue was triturated with dry acetone (5 mL). The mixture was filtered through a pad of Celite and the residue were washed with additional acetone (3×2 mL). The combined solution was concentrated in vacuo to give white solid. The solid was washed with ether (3×5 mL) and petroleum ether (3×5 mL) and dried under vacuum, affording the desired product **2a-I** as a white solid (189.7 mg, 89 %). **Mp** 188-189 ºC

^{1}H NMR (400 MHz, DMSO-d_6) δ 7.11 – 6.95 (m, 4H), 6.92 – 6.81 (m, 1H), 1.58 (brs, 1H), 1.03 (d, J = 7.3 Hz, 3H) ppm.

^{13}C NMR (101 MHz, DMSO-d_6) δ 152.7, 127.9, 127.2, 122.6, 17.7 ppm.

^{19}F NMR (376 MHz, DMSO-d_6) δ -147.11 ppm.

The synthesis of 2-phenyl-1-propanol (2a-II)[17]

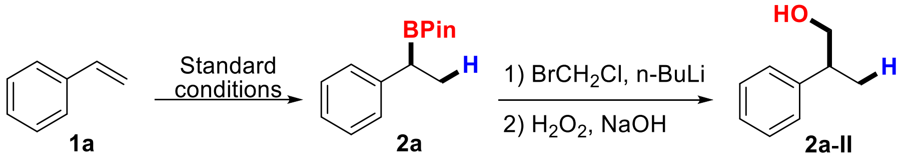

In a nitrogen filled glovebox, CoI_2 (1.56 mg, 0.5 mol%), KO^{t}Bu (11.2mg, 0.1 mmol), THF (5 mL, 0.2 M) and styrene **1a** (104 mg, 1.0 mmol) were added to a 25 mL Schlenk tube equipped with a magnetic stir bar. HBPin (153.6 mg, 174 µL, 1.2 mmol) was then added to the mixture. The reaction mixture was stirred at 30 ºC for 1 h and then $BrCH_2Cl$ (154.8 mg, 1.2 mmol) was added. After cooling to -78 °C, n-BuLi (2.4 M in hexane, 0.50 mL, 1.2 mmol) was added dropwise over 10 min to the flask with vigorous stirring. The reaction mixture was then allowed to warm gradually to room temperature overnight. Then, methanol (2.0 mL), NaOH (1 M in water, 4.0 mL) and H_2O_2 (30% w/v in water, 0.63 mL, 5 mmol) were added, and the reaction mixture was stirred at room temperature for 3h. The organic layer was separated, and the water layer was extracted by ethyl acetate (3×10 mL). The combined organic layer was washed with brine, dried with $MgSO_4$ and concentrated under vacuum. The crude product was purified by column chromatography on silica (PE:Et_2O = 5:1), affording the desired product **2a-II** as yellow oil (99.7 mg, 73 %).

^{1}H NMR (400 MHz, $CDCl_3$) δ 7.40 – 7.17 (m, 5H), 3.67 (d, J = 6.8 Hz, 2H), 2.93 (h, J = 6.7 Hz, 1H), 1.74 (s, 1H), 1.27 (d, J = 6.9 Hz, 3H) ppm.

^{13}C NMR (101 MHz, $CDCl_3$) δ 143.9, 128.7, 127.6, 126.8, 68.8, 42.6, 17.8 ppm.

6. Transformations from benzonitrile by a one-pot process

The synthesis of *N*-benzylidenebenzylamine (6a-I)[18]

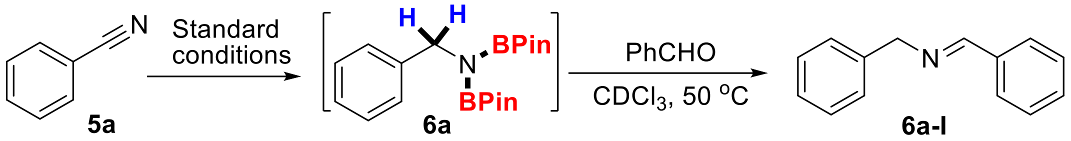

In a nitrogen filled glovebox, CoI_2 (0.78 mg, 0.0025 mmol, 0.5 mol%), KO^{t}Bu (5.6 mg, 0.05 mmol), and benzonitrile **5a** (51.6 mg, 51 μL, 0.5 mmol) were added to a 10 mL sealed tube equipped with a magnetic stir bar. HBPin (140.8 mg, 160 μL, 1.1 mmol) was then added, and the reaction mixture was stirred at 30 °C for 4 h. Then, $CDCl_3$ (2 mL) and benzaldehyde (58.3 mg, 56 μL, 0.55 mmol) was added and the reaction mixture was heated for 5 hours at 50 °C. The yield of product (89%) was determined by ^{1}H-NMR with 1,3,5-trimethoxybenzene (28.6 mg, 0.17 mmol) as internal.

^{1}H NMR (400 MHz, $CDCl_3$) δ 8.42 – 8.37 (m, 1H), 7.83 – 7.75 (m, 2H), 7.46 – 7.37 (m, 3H), 7.37 – 7.30 (m, 4H), 7.29 – 7.26 (m, 1H), 4.83 (s, 2H) ppm.

The synthesis of N-benzylbenzamide (6a-II)[19]

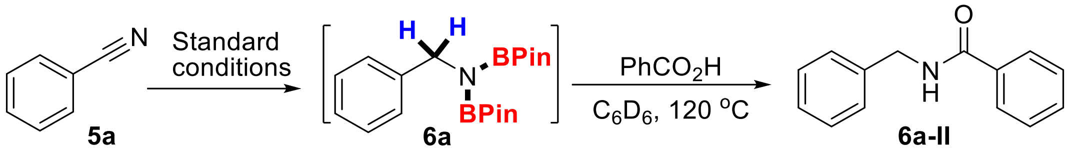

In a nitrogen filled glovebox, CoI_2 (0.78 mg, 0.0025 mmol, 0.5 mol%), KO^{t}Bu (5.6 mg, 0.05 mmol), and benzonitrile **5a** (51.6 mg, 51 μL, 0.5 mmol) were added to a 10 mL sealed tube equipped with a magnetic stir bar. HBPin (140.8 mg, 160 μL, 1.1 mmol) was then added, and the reaction mixture was stirred at 30 °C for 4 h. Then, C_6D_6 (2 mL) and benzoic acid (73.2 mg, 0.6 mmol) was added and the reaction mixture was heated for 24 hours at 120 °C. Volatiles were removed under vacuum, and the residue was purified by column chromatography (PE : EA = 8:1, with 1% TEA) to obtain a white solid (84.3 mg, 80%). **Mp** 99-100 °C

^{1}H NMR (400 MHz, $CDCl_3$) δ 7.99 (d, *J* = 7.2 Hz, 2H), 7.45 – 7.40 (m, 3H), 7.39 – 7.27 (m, 5H), 7.01 (brs, 1H), 4.71 (d, *J* = 5.9 Hz, 2H) ppm.

^{13}C NMR (101 MHz, $CDCl_3$) δ 167.5, 138.3, 134.5, 131.6, 128.9, 128.7, 128.0, 127.7, 127.1, 44.2 ppm.

7. Mechanism studies

7.1 ICP-MS Analyses for Trace Metal Contaminants

ICP-MS (Inductively Coupled Plasma Mass Spectroscopy) analysis of a typical reaction:

Nitric acid (1 mL, Conc.) was carefully added dropwise to a mixture of CoI_2 (2 mol%), styrene (1 mmol), HBPin (0.5 mmol) and KO^{t}Bu (10 mol%). The mixture was diluted with water (ultrapure) to a total volume of 50 mL.

Analysis of the resulting solution was performed using an Thermo iCAP Q ICP-MS calibrated against a multi-element standard solution.

Supplementary Table 7. Concentration of elements determined by ICP-MS

Entry	Concentration (ppb)	Relative to Co (Element/Co)
Al	168.81	5.38×10^{-2}
Cr	0.10	3.28×10^{-5}
Mn	0.36	1.14×10^{-4}
Fe	11.31	3.61×10^{-3}
Co	3135.45	1.00
Ni	0.29	9.17×10^{-5}
Cu	0.53	1.69×10^{-4}
Ru	0.01<	4.22×10^{-8}
Rh	0.17	5.43×10^{-5}
Pd	0.15	4.84×10^{-5}
Ag	0.75	2.40×10^{-4}
Os	0.01<	1.02×10^{-7}
Ir	0.01	4.75×10^{-6}
Pt	0.99	3.17×10^{-4}
Au	0.34	1.09×10^{-4}
Hg	1.98	6.32×10^{-4}

7.2 Kinetic & poisoning studies

7.2.1 One more equivalent of substrates added to test the activity of the catalyst

Standard condition for kinetic studies: In a nitrogen filled glovebox, CoI_2 (1.25 mg, 0.004 mmol, 0.2 mol%), KO^{t}Bu (11.2 mg, 0.1 mmol, 5 mol%), THF (15 mL) and styrene **1a** (208 mg, 0.23 mL, 2.0 mmol) were added to a 20 mL vial equipped with a magnetic stir bar. Mesitylene (200 μL) was added as the internal. HBPin (307.2 mg, 0.35 mL, 2.4 mmol) was then added, and the reaction mixture was stirred at 30 ºC.

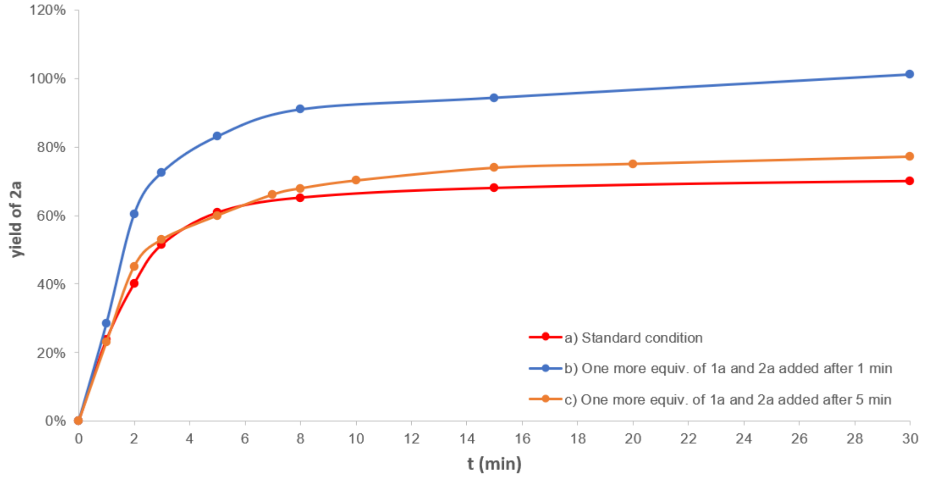

Supplementary Figure 1. Additional substrates for testing the activity of the catalyst. One more equivalent of substrates (2.0 mmol **1a**, 2.4 mmol HBPin) were added at 1.0 min or 5.0 min. The yield of boronic ester **2a** was calculated by GC according to the initial amount of **1a**.

7.2.2 Poisoning experiments

General reaction condition for poisoning experiments: In a nitrogen filled glovebox, CoI_2 (1.25 mg, 0.004 mmol, 0.2 mol%), KO^{t}Bu (11.2 mg, 0.1 mmol, 5 mol%), THF (15 mL) and styrene **1a** (208 mg, 0.23 mL, 2 mmol) were added to a 20 mL vial equipped with a magnetic stir bar. Mesitylene (200 μL) was added as the internal. HBPin (307.2 mg, 0.35 mL, 2.4 mmol) was then added, and the reaction mixture was stirred at 30 ºC. After 1.0 min, poisoning reagent was added.

Mercury (Hg)

Excess Hg was added at 1.0 min after the reaction beginning.

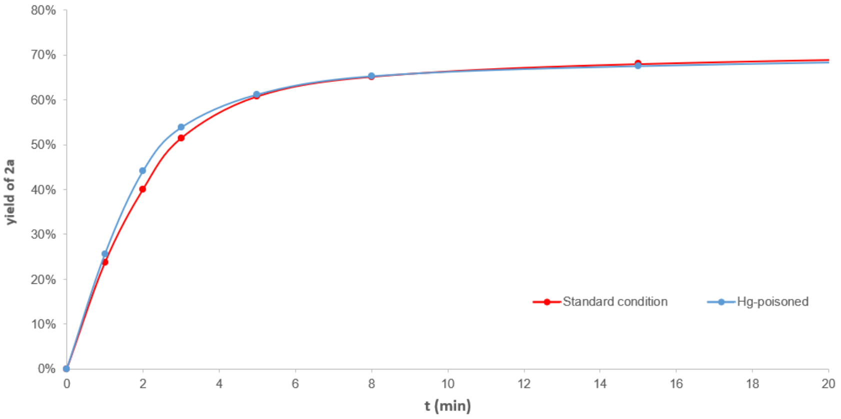

Supplementary Figure 2. Poisoning studies with mercury. The yield of boronic esters was determined by GC (mesitylene as internal).

Trimethylphosphite

A defined volume of a standardized solution of $P(OMe)_3$ in THF [$P(OMe)_3$, 0.2, 0.3 or 0.5 equiv per Co atom] was added to the reaction solution with a Hamilton syringe at 1.0 minute after the reaction beginning.

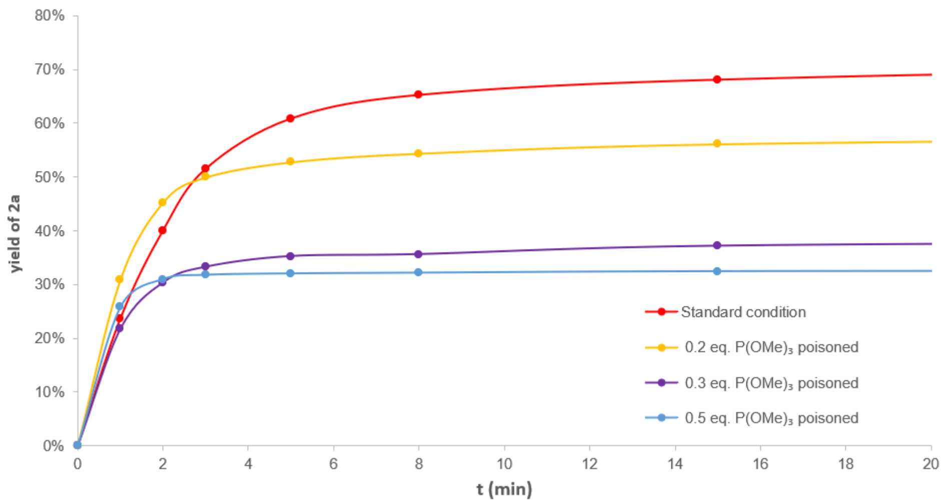

Supplementary Figure 3. Poisoning studies with $P(OMe)_3$. The yield of boronic ester was determined by GC (mesitylene as internal).

Dibenzo[a,e]cyclooctatetraene (dct)

A defined volume of a standardized solution of dct in THF was added to the reaction solution with a Hamilton syringe at 1.0 minute after the reaction beginning.

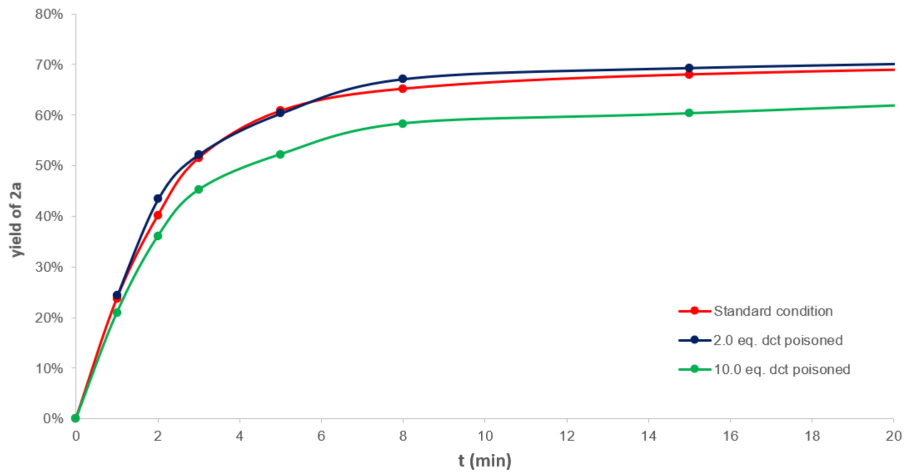

Supplementary Figure 4. Poisoning studies with dct. The yield of boronic ester was determined by GC (mesitylene as internal).

7.3 TEM Analysis

Two samples were analyzed by TEM (JEOL 2100 LAB6 operating at 200 kV coupled with an energy-dispersive X-ray spectroscopy detector SDD X-Oxford TSR).

The first sample was prepared from the reaction mixture collected at 2 minutes after the reaction beginning under the standard conditions. After being filtered through a 0.22 μm membrane, drops of the filtrate were deposited onto holey carbon grids. All these operations took about three minutes.

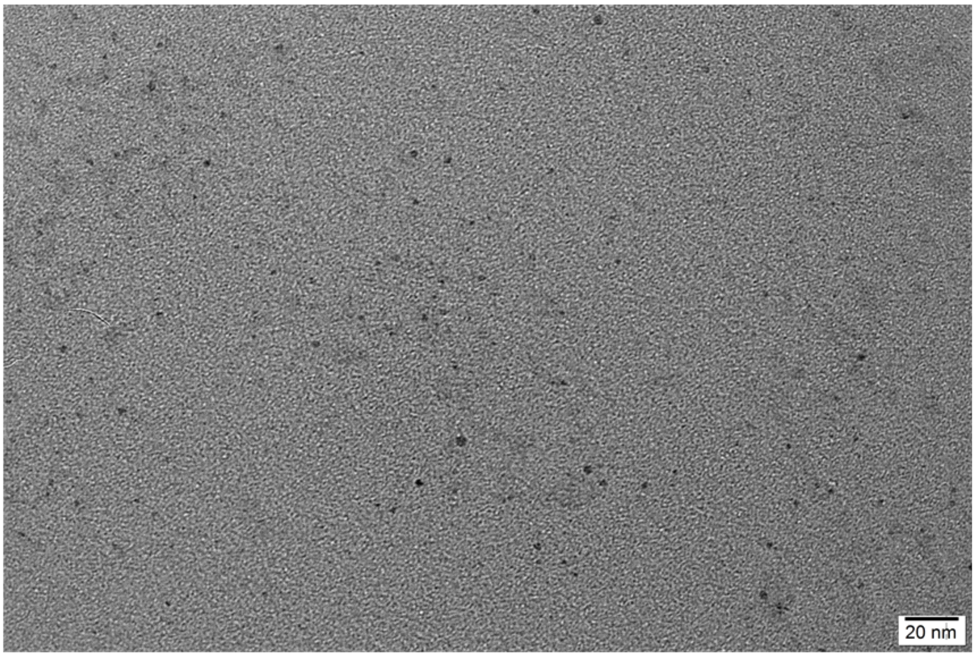

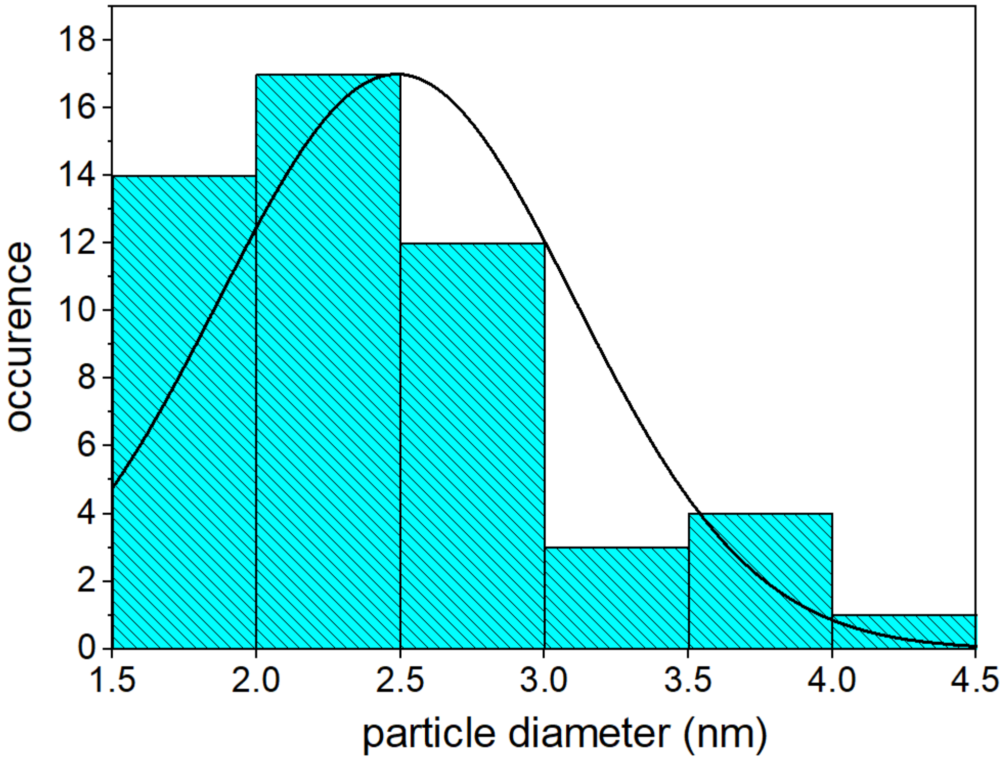

Supplementary Figure 5. The TEM image (above) and the particle size distribution (below) of the first sample.

The second sample was prepared from the reaction mixture collected at 60 minutes after the reaction beginning under the standard conditions. After being filtered through a 0.22 μm membrane, drops of the filtrate were deposited onto holey carbon grids.

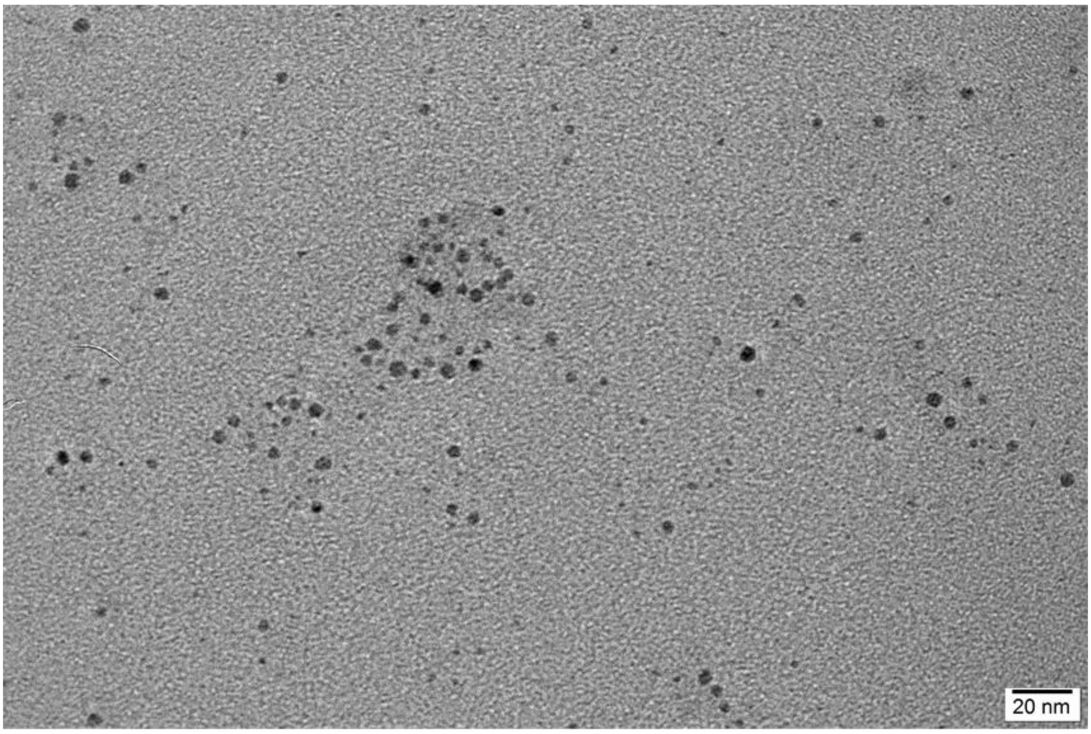

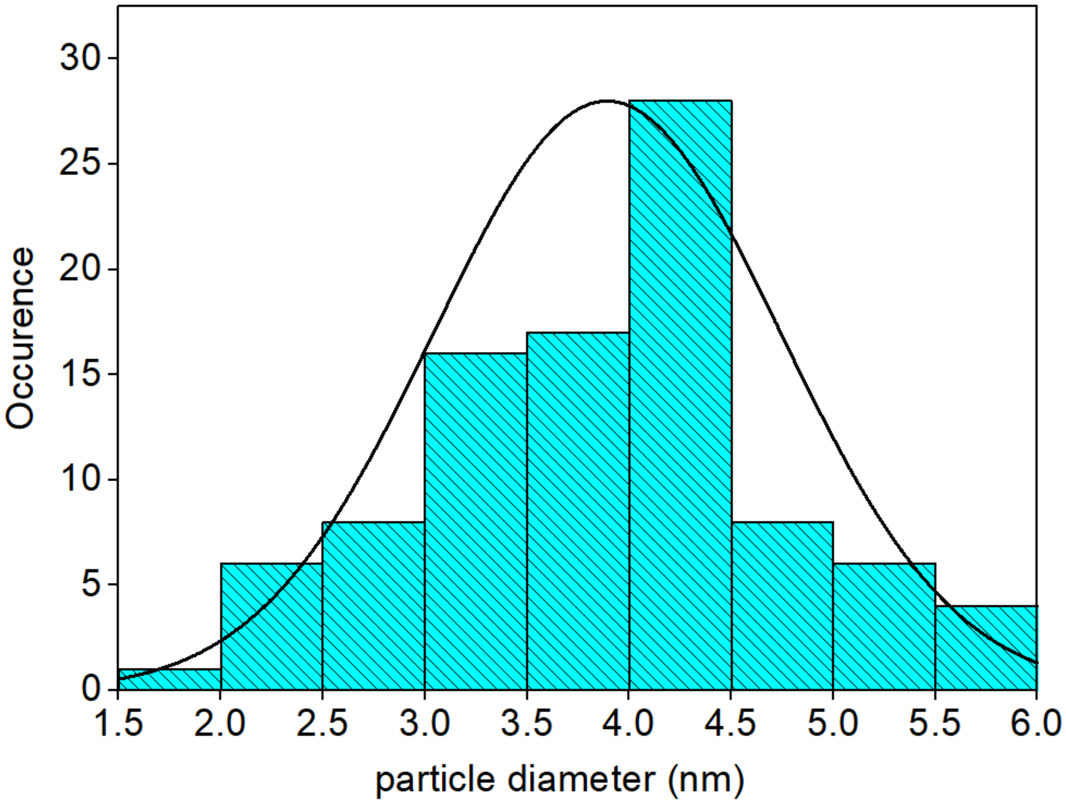

Supplementary Figure 6. The TEM image (above) and the particle size distribution (below) of the second sample.

7.4 SAESI-MS Analysis

SAESI-MS spectra were recorded on a Thermo TSQ Quantum Access triple-quadrupole mass spectrometer (Thermo Fisher Scientific, Waltham, MA) equipped with a home-made SAESI ion source in positive mode. The basic SAESI conditions were: vacuum, 2.8×10^{-6} torr; spray voltage, 4000 V; capillary temperature, 275˚C; sheath gas pressure of two sprayers, 3 arb. units; the collision energy of CID, 20 eV. Data acquisition and analysis were done with the Xcalibur (version 2.0, Thermo Fisher Scientific) software package. In solvent-assisted electrospray ionization mass spectrometric experiment, the angle (α) between the two sprayers is 45° and the distance (b) between the tip of sprayers and the inlet to the mass is 6 mm. The chemical solutions were injected by a 500-μL air-tight syringe with a speed at 5 μL/min to SAESI-MS. The assisted solvent of methanol was injected by another 500-μL air-tight syringe with a speed at 5 μL/min.

Under Ar, CoI_2 (1.25mg, 0.004mmol, 0.2 mol%), KO^{t}Bu (11.2 mg, 0.1 mmol, 5 mol%), THF (10 mL) and styrene (208 mg, 2 mmol) were added to a 25 mL Schlenk tube equipped with a magnetic stir bar. HBPin (307 mg, 2.4 mmol) was then added to the mixture. The reaction mixture was determined by SAESI-MS immediately.

a)

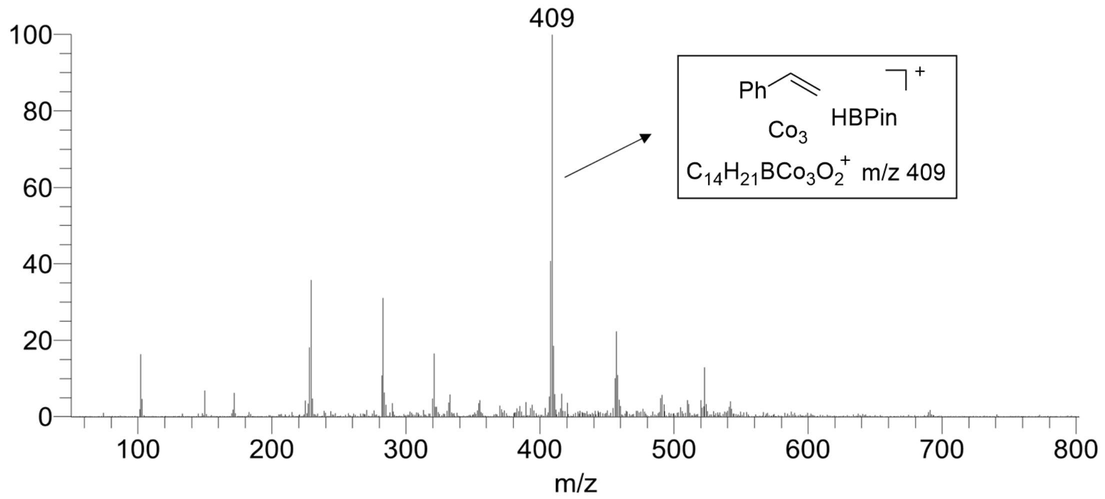

b)

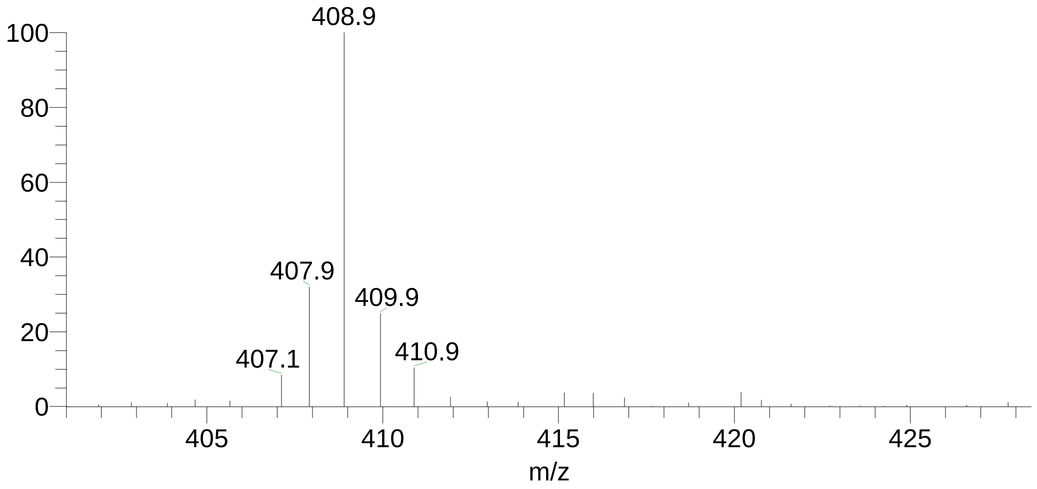

c)

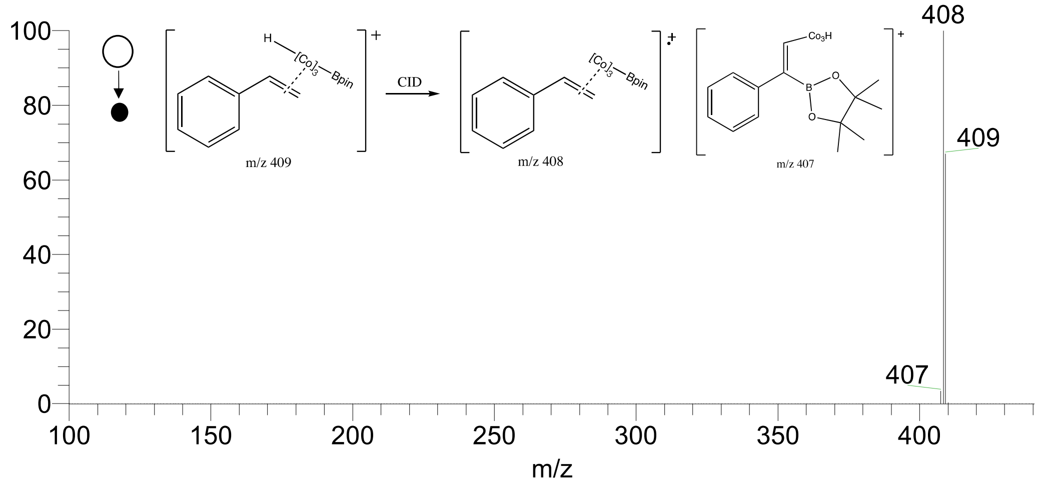

Supplementary Figure 7. SAESI-MS spectra. a) SAESI-MS spectrum of the reaction solution. b) Expanded SAESI-MS spectrum, showing the major signal from m/z 400 to 430. c) SAESI-MS/MS spectrum of complex ion at *m/z* 409.

7.5 Radical-trapping experiments

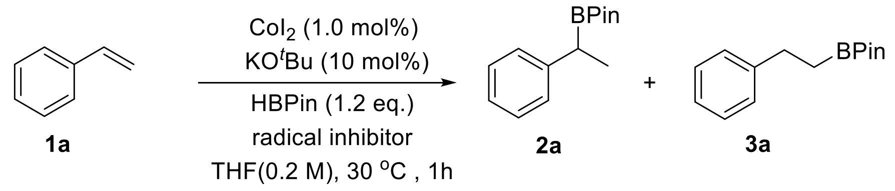

Supplementary Table 7. The result of radical-trapping experiments

Entry	radical inhibitors	Yield **2a** (%)
1	-	90
2	0.5 eq. BHT	30
3	0.5 eq. TEMPO	36
4	1.5 eq. 1,1-diphenylethene	76

General-procedure for radical trapping experiments. In a nitrogen filled glovebox, CoI_2 (1.25mg, 0.004 mmol, 1.0 mol%), KO^{t}Bu (4.5 mg, 0.04 mmol, 10 mol%), radical inhibitors, THF (2 mL) and styrene (0.4 mmol) were added to a 10 mL vial equipped with a magnetic stir bar. HBPin (61.4 mg, 70 µL, 0.48 mmol) was then added to the mixture. The reaction mixture was stirred at 30 °C for 1.0 h and then was quenched by exposing the solution to air. The yield of compound **2a** was determined by GC analysis.

7.6 The effect of the addition of crown ethers

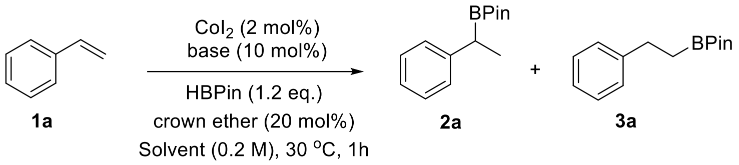

Supplementary Table 8. The result of hydroboration with or without crown ethers

Entry	base	crown ether	Yield (**2a**+**3a**) (%)[b]	**2a**/**3a**[b]
1	KO^{t}Bu	-	92	53.3/1
2	KO^{t}Bu	18-crown-6	87	17.3/1
3	NaO^{t}Bu	-	88	42.8/1
4	NaO^{t}Bu	15-crown-5	86	12.5/1
5	LiO^{t}Bu	-	86	7.3/1
6	LiO^{t}Bu	12-crown-4	17	5.2/1

General-procedure for hydroboration with or without crown ethers: In a nitrogen filled glovebox, CoI_2 (2.5 mg, 0.008 mmol, 2.0 mol%), base (0.04 mmol, 10 mol%), crown ethers (with or without, 0.08 mmol, 20 mol%), THF (2 mL) and styrene (0.4 mmol) were added to a 10 mL vial equipped with a magnetic stir bar. The mixture was stirred for 5 min and HBPin (61.4 mg, 70 µL, 0.48 mmol) was then added to the mixture. The reaction mixture was stirred at 30 °C for 1.0 h and then was quenched by exposing the solution to air. The yield of compound **2a** and the ratio of **2a**/**3a** was determined by GC analysis.

7.7 Other borane sources used in this cobalt catalytic system

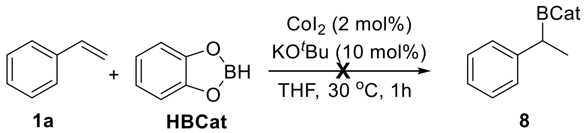

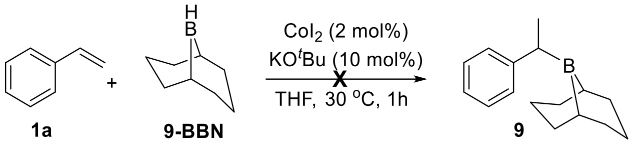

In a nitrogen filled glovebox, CoI_2 (6.26 mg, 0.02 mmol, 2.0 mol%), KO^{t}Bu (11.2 mg, 0.1 mmol, 10 mol%), THF (5 mL) and styrene (104.2 mg, 1.0 mmol) were added to a 10 mL vial equipped with a magnetic stir bar. Catechol borane (HBCat, 143.9 mg, 128 µL, 1.2 mmol) or 9-borabicyclo[3.3.1]nonane (9-BBN, 0.5 M in THF, 2.4 mL, 1.2 mmol) was then added to the mixture. The reaction mixture was stirred at 30 °C for 1.0 h and then was quenched by exposing the solution to air. The reaction mixture was determined by GC-MS and the conversion of substrate **1a** was determined by GC analysis. No hydroboration product was found with <5% conversion of substrate **1a**.

7.8 Co(II)(alkoxide)$_2$ salt as the potential pre-catalyst

The preparation of Co(II)(alkoxide)$_2$ salt[20]: In a nitrogen filled glovebox, an oven-dried 25 mL screw-cap vial equipped with a magnetic stir bar was charged with CoI_2 (312.7 mg, 1.0 mmol), KO^{t}Bu (224.4 mg, 2.0 mmol) and 4 mL of dry THF. Resulting mixture was stirred at 60˚C for 24 hours. After cooling down to room temperature, in the glove box, purple solid was filtered off and washed 3 times with small portions of hexane. After drying under reduced pressure, 418.2 mg of precatalyst Co(O^{t}Bu)$_2$•nKI was obtained. Co(O^{t}Bu)$_2$•nKI is **air sensitive** and should be stored under N_2.
CHN elemental analysis: found: C, 18.27; H, 3.44; N, not found.
The observed C:H ratio (5.311) and the absence of N suggest that the organic part of the precatalyst should be O^{t}Bu (calculated C:H ratio is 5.296).
ICP-OES elemental analysis: found: K, 13.78; Co, 9.97.
The K : Co ratio is approximately 2.08.

The application of Co(II)(alkoxide)$_2$ salts: In a nitrogen filled glovebox, Co(O^{t}Bu)$_2$•nKI (5.0 mg, approximately 0.01 mmol), THF (5 mL) and styrene (104.2 mg, 1.0 mmol) were added to a 10 mL vial equipped with a magnetic stir bar. HBPin (153.6 mg, 174 µL, 1.2 mmol) was then added to the mixture. The reaction mixture was stirred at 30 ºC for 1.0 h and then was quenched by exposing the solution to air. The yield of compound **2a** was determined by GC analysis.

However, this coablt alkoxide salt did not show a good catalytic activity in the hydroboration of styrene in the absence of KO^{t}Bu, resulting in only 6 % yield of the product **2a** with 2:1 B/L selectivity, suggesting that bis-tert-butoxide cobalt salt itself is not the catalytically active species. This result is also consistent with the significant role of a large excess of reductant formed i*n situ* from KO^{t}Bu and HBPin in the present catalysis.

7.9 ^{11}B NMR of the reaction mixture

In a nitrogen filled glovebox, CoI_2 (6.26 mg, 0.02 mmol, 2.0 mol%), KO^{t}Bu (11.2 mg, 0.1 mmol, 10 mol%), THF (5 mL) and styrene **1a** (104.2 mg, 1.0 mmol) were added to a 10 mL vial equipped with a magnetic stir bar. HBPin (153.6 mg, 174 µL, 1.2 mmol) was then added to the mixture and the reaction mixture was stirred at 30 °C for 1.0 h. The ^{11}B NMR of the resulting solution was collected and the signal of diboraxane was observed, suggesting that diboraxane existed in the reaction mixture, which might be formed from the reaction of HBPin with ineluctable H_2O.[21,22] It should be noted that trace amount of diboraxane was also detected in the reagent HBPin which was used in the reaction by ^{11}B NMR.

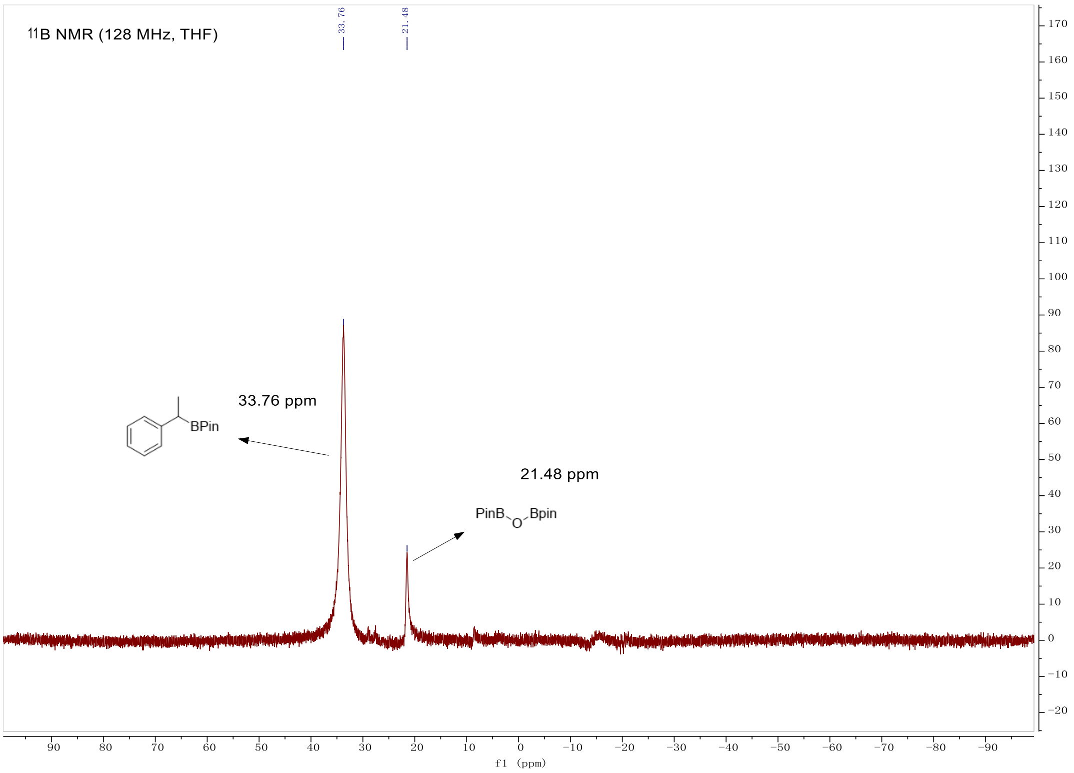

Supplementary Figure 8. ^{11}B NMR of the reaction mixture.

8. NMR Spectra

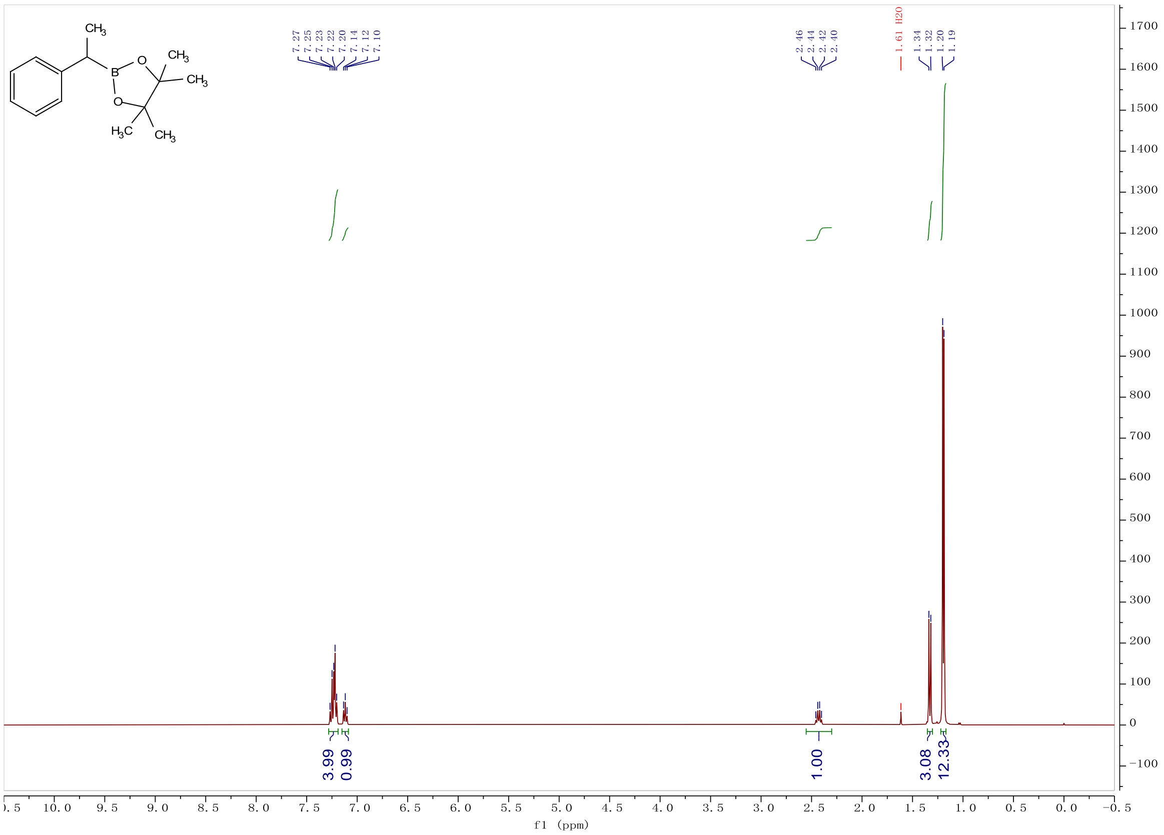

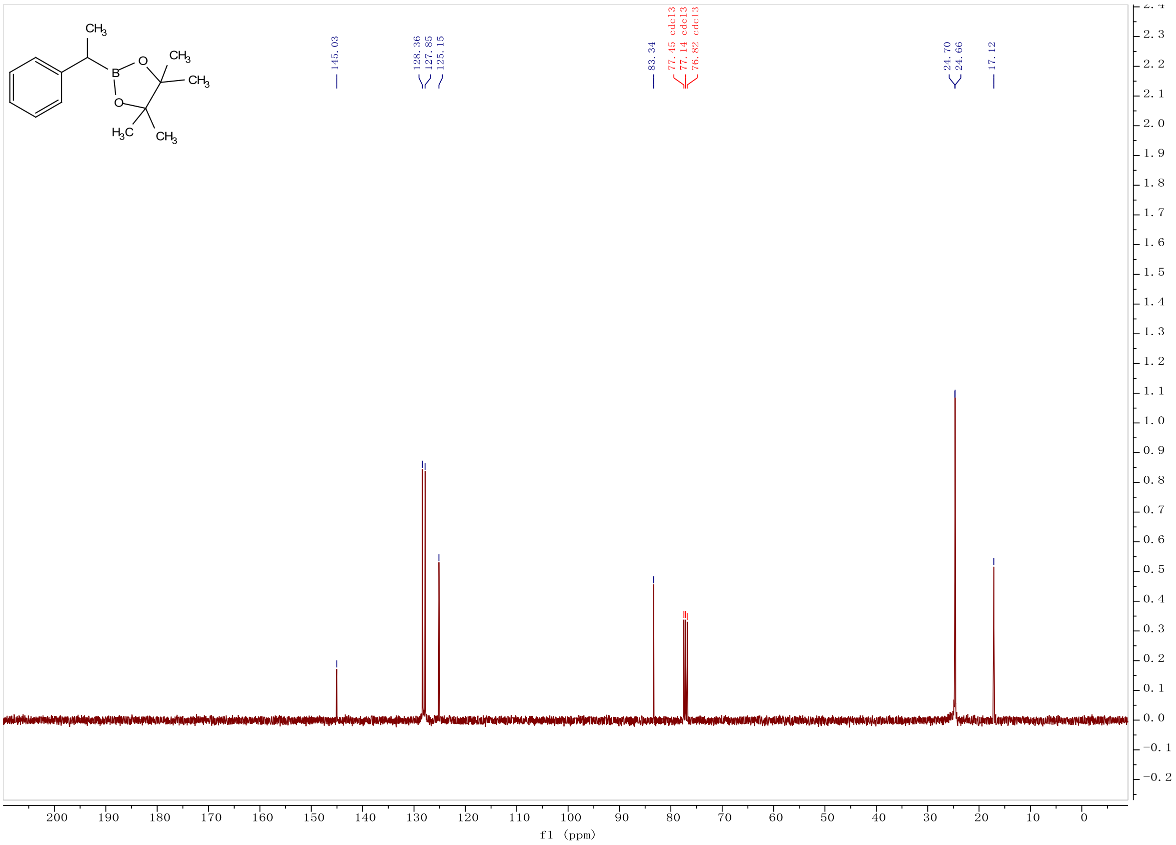

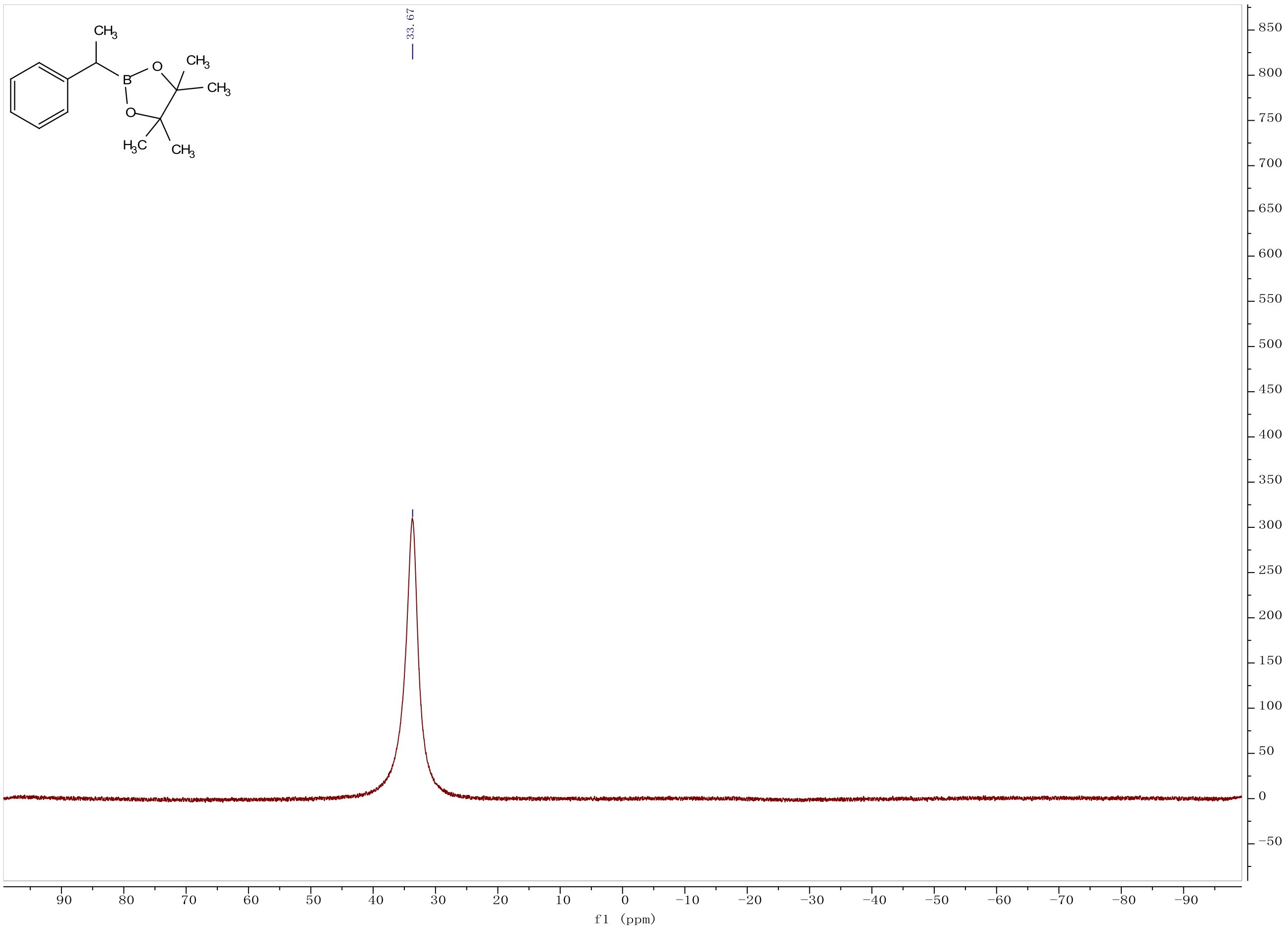

Supplementary Figure 9. ^{1}**H,** ^{13}**C,** ^{11}**B NMR** of compound **2a**.

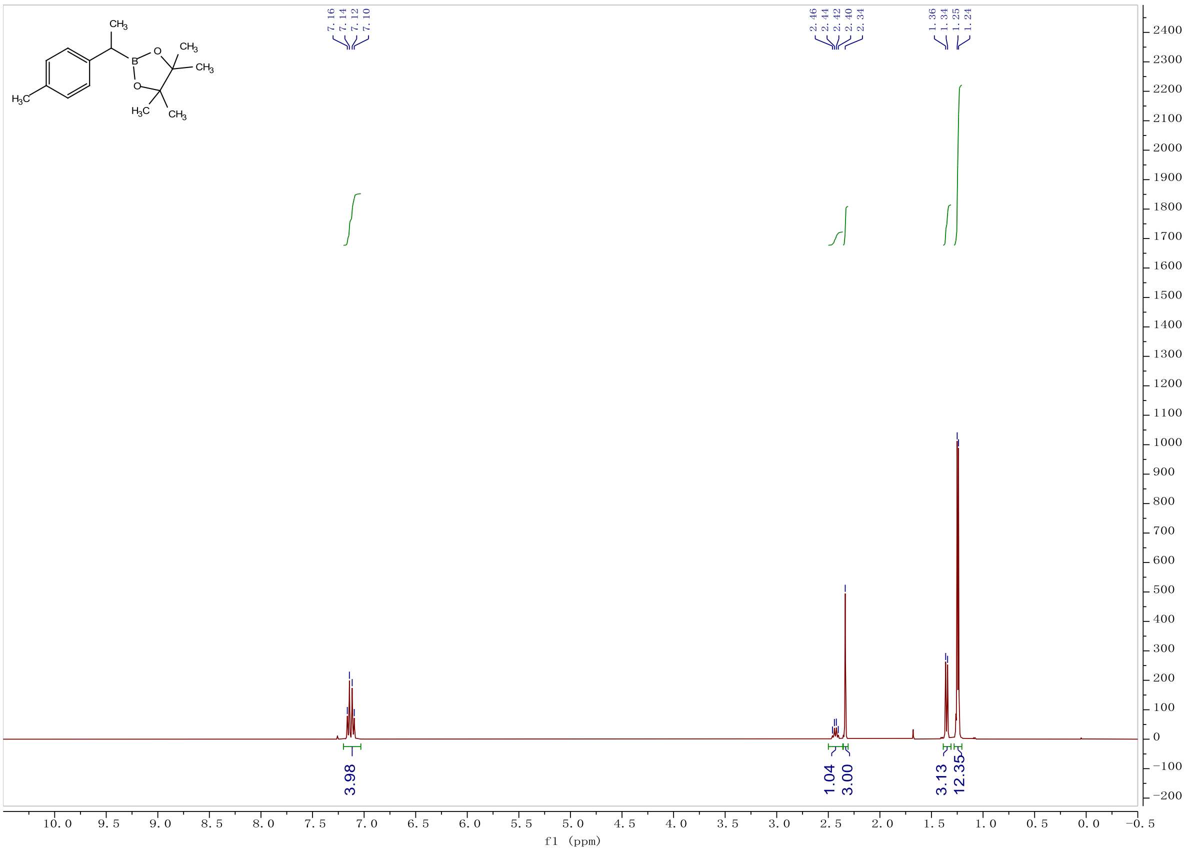
7.16
7.14
7.12
7.10
2.46
2.44
2.42
2.40
2.34
1.36
1.34
1.25
1.24
3.98
1.04
3.00
3.13
12.35
f1 (ppm)

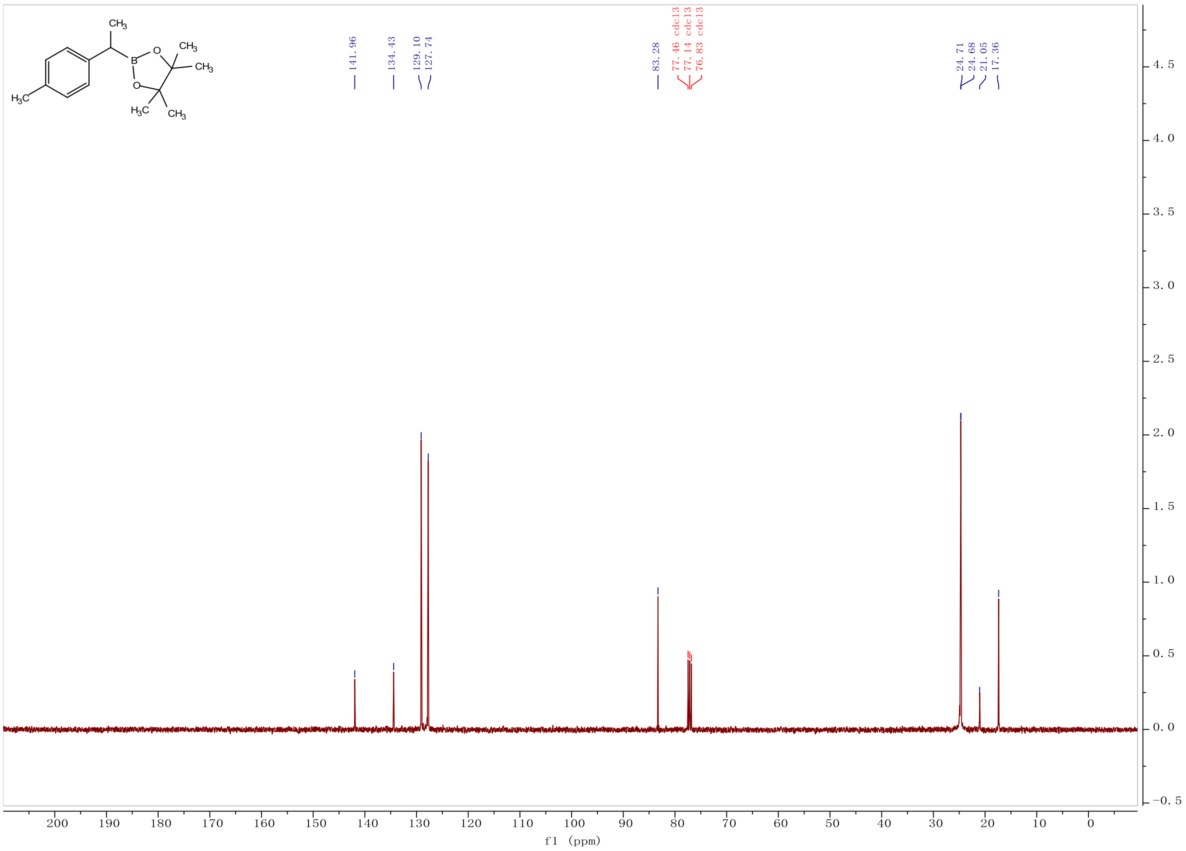
141.96
134.43
129.10
127.74
83.28
77.46 cdcl3
77.14 cdcl3
76.83 cdcl3
24.71
24.68
21.05
17.36
f1 (ppm)

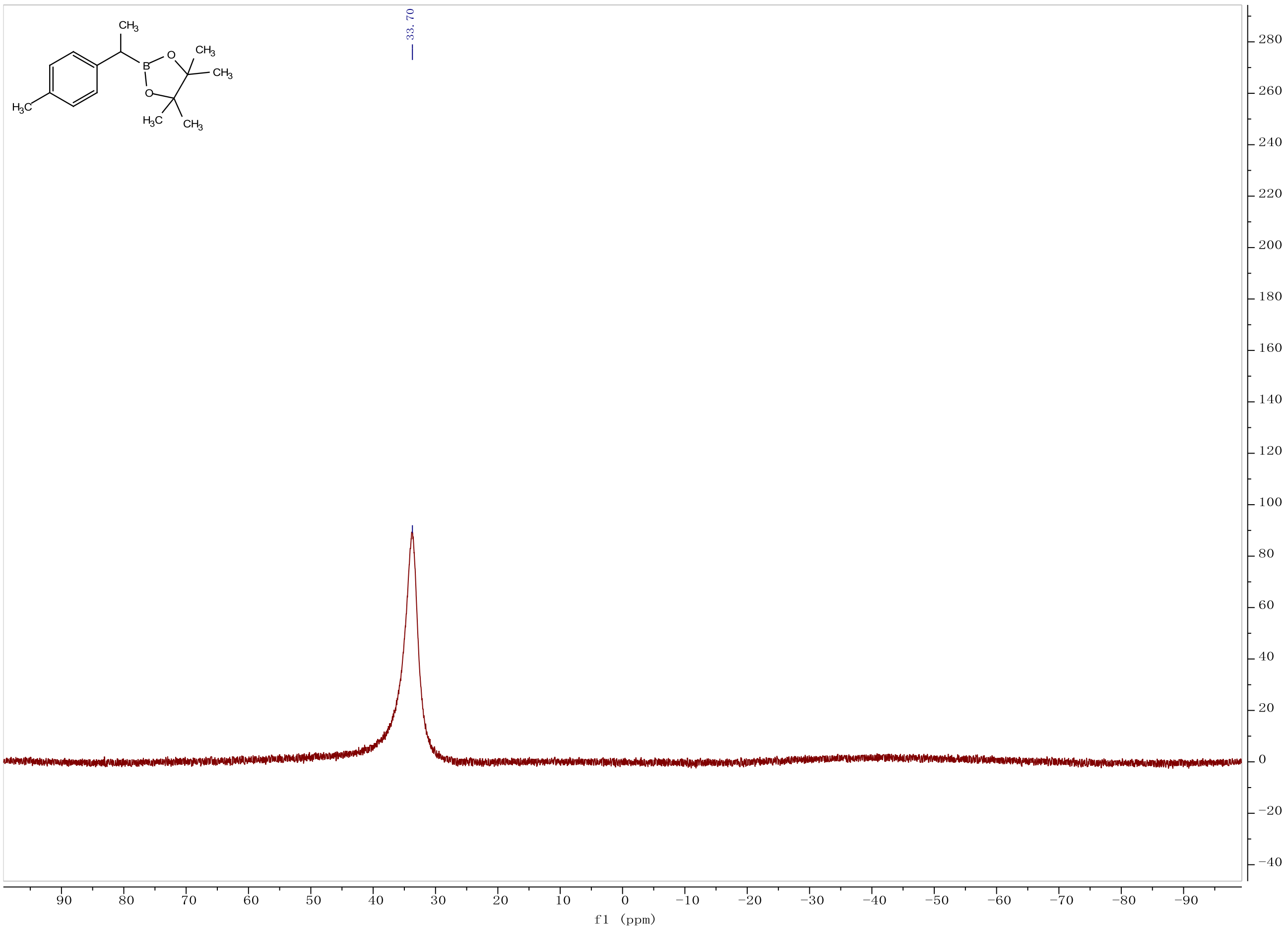

Supplementary Figure 10. ^{1}**H**, ^{13}**C,** ^{11}**B NMR** of compound **2b**.

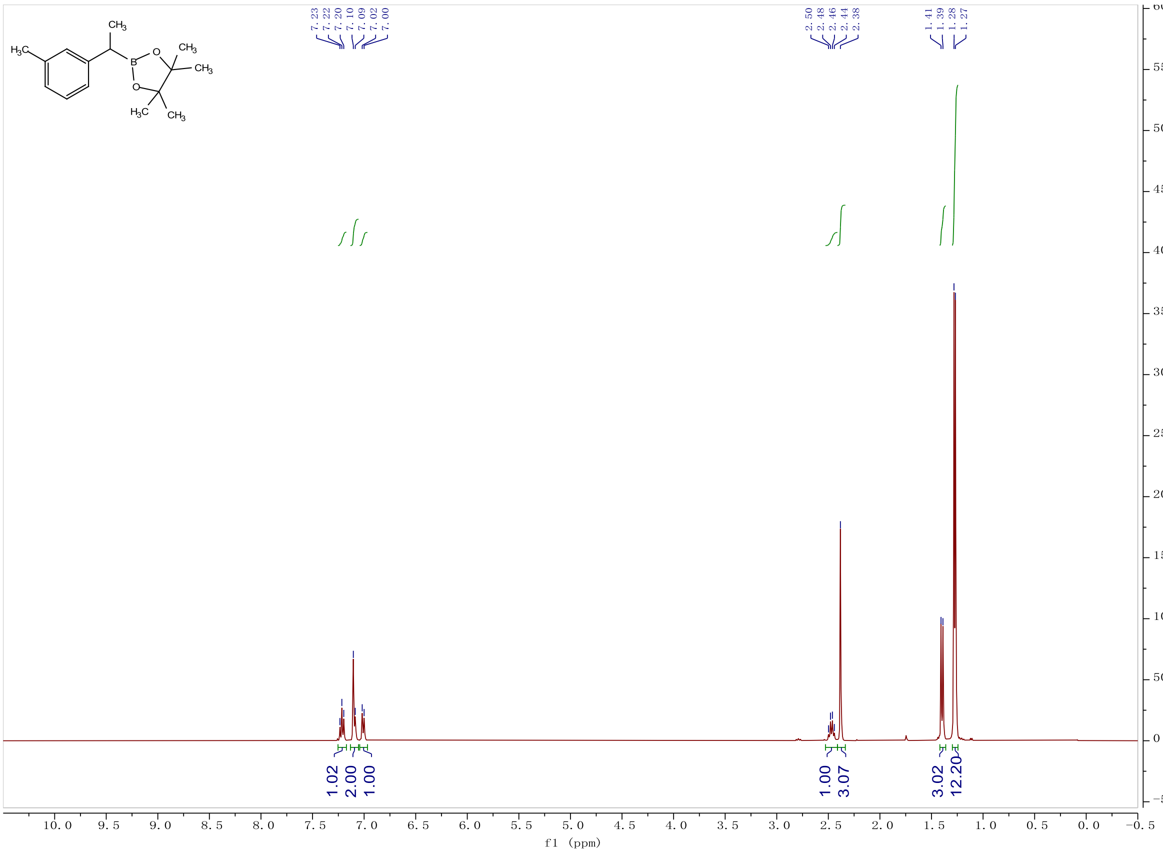
CH3
H3C
B
O
O
CH3
CH3
H3C
CH3
7.23
7.22
7.20
7.10
7.09
7.02
7.00
2.50
2.48
2.46
2.44
2.38
1.41
1.39
1.28
1.27
1.02
2.00
1.00
1.00
3.07
3.02
12.20
f1 (ppm)

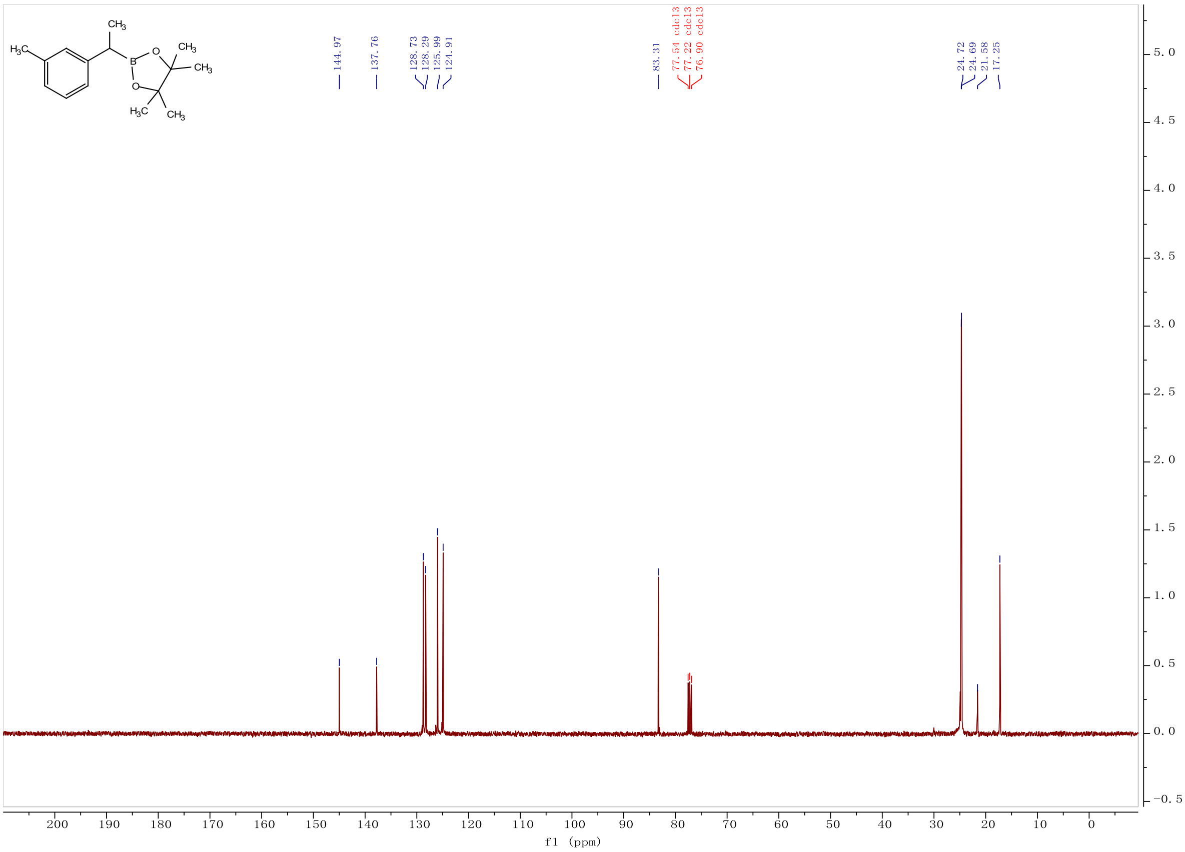
CH3
H3C
B
O
O
CH3
CH3
H3C
CH3
144.97
137.76
128.73
128.29
125.99
124.91
83.31
77.54 cdcl3
77.22 cdcl3
76.90 cdcl3
24.72
24.69
21.58
17.25
f1 (ppm)

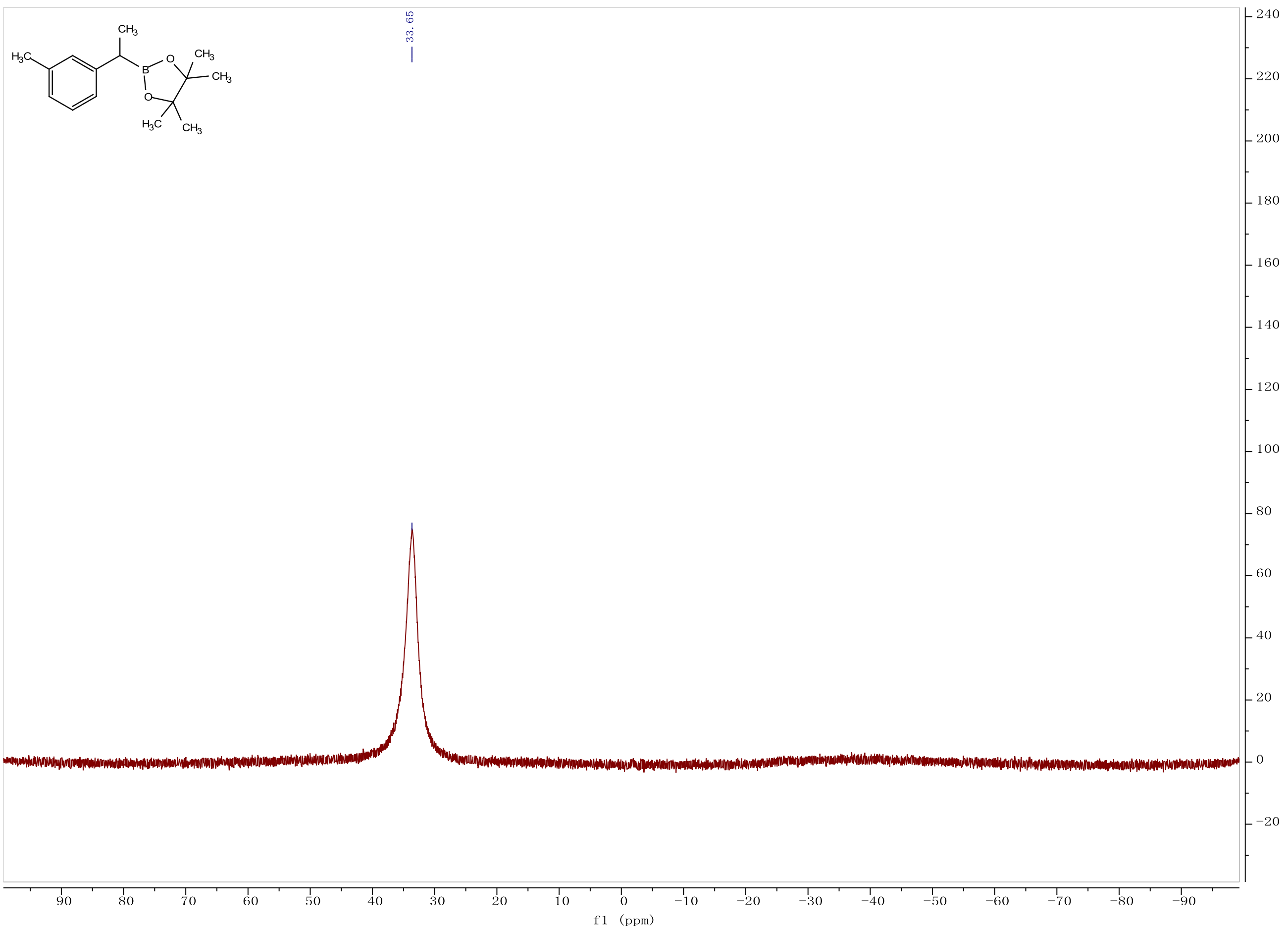

Supplementary Figure 11. ^{1}**H**, ^{13}**C,** ^{11}**B NMR** of compound **2c**.

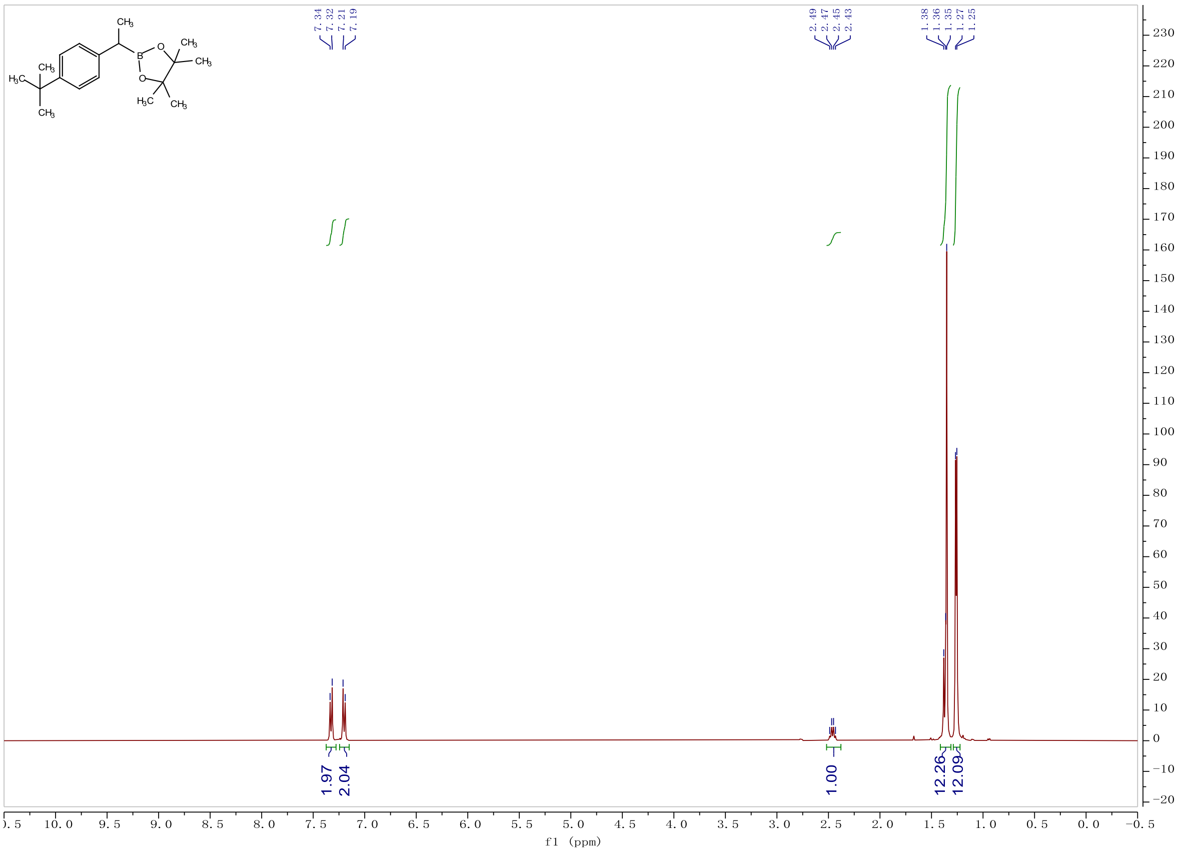
7.34
7.32
7.21
7.19
2.49
2.47
2.45
2.43
1.38
1.36
1.35
1.27
1.25
1.97
2.04
1.00
12.26
12.09
f1 (ppm)

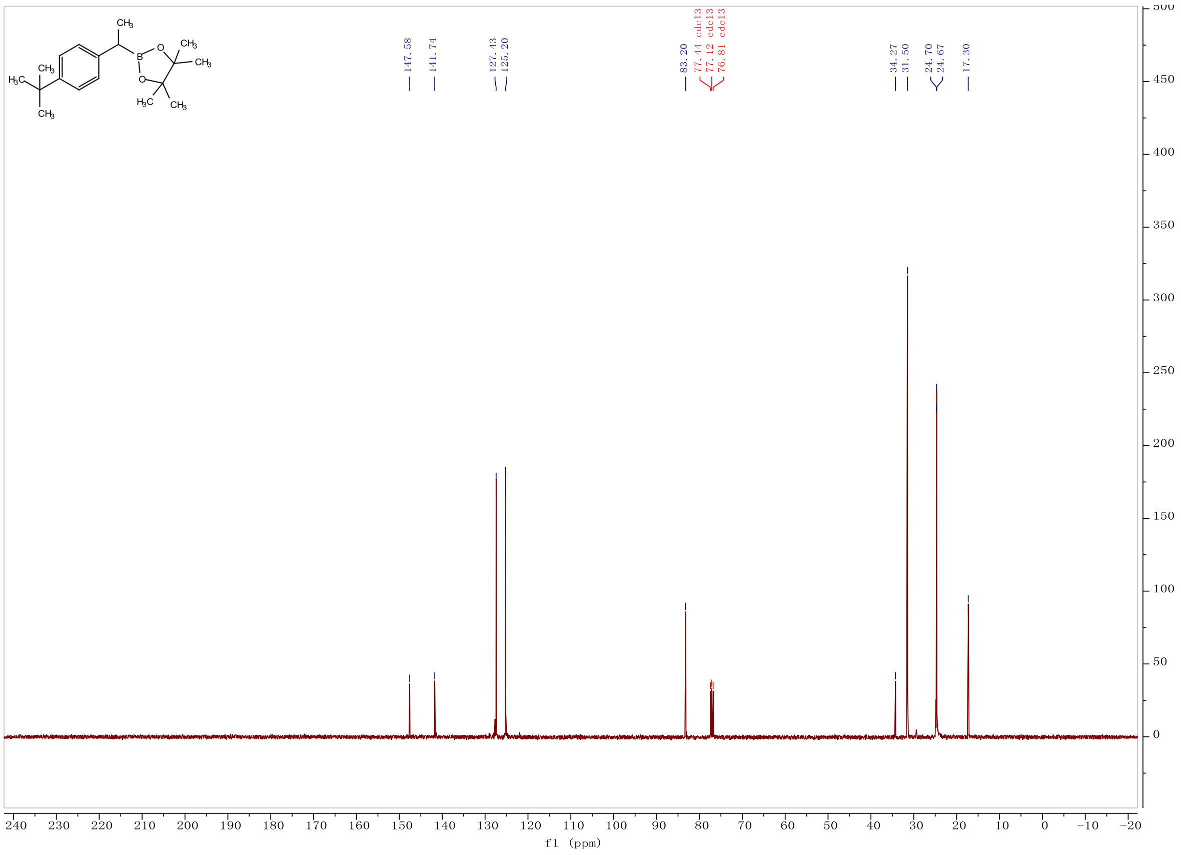
147.58
141.74
127.43
125.20
83.20
77.44 cdcl3
77.12 cdcl3
76.81 cdcl3
34.27
31.50
24.70
24.67
17.30
f1 (ppm)

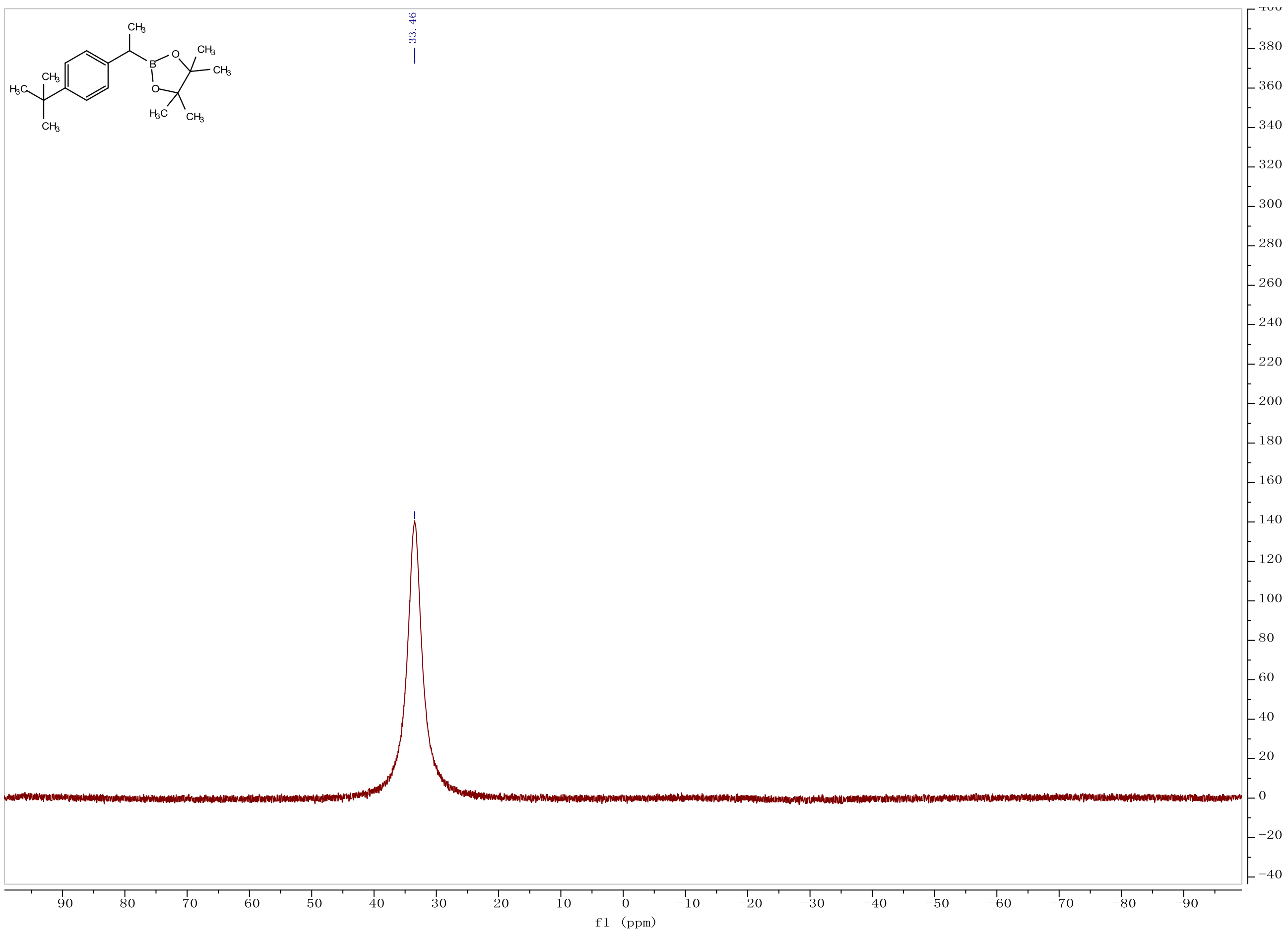

Supplementary Figure 12. ^{1}**H**, ^{13}**C,** ^{11}**B NMR** of compound **2d**.

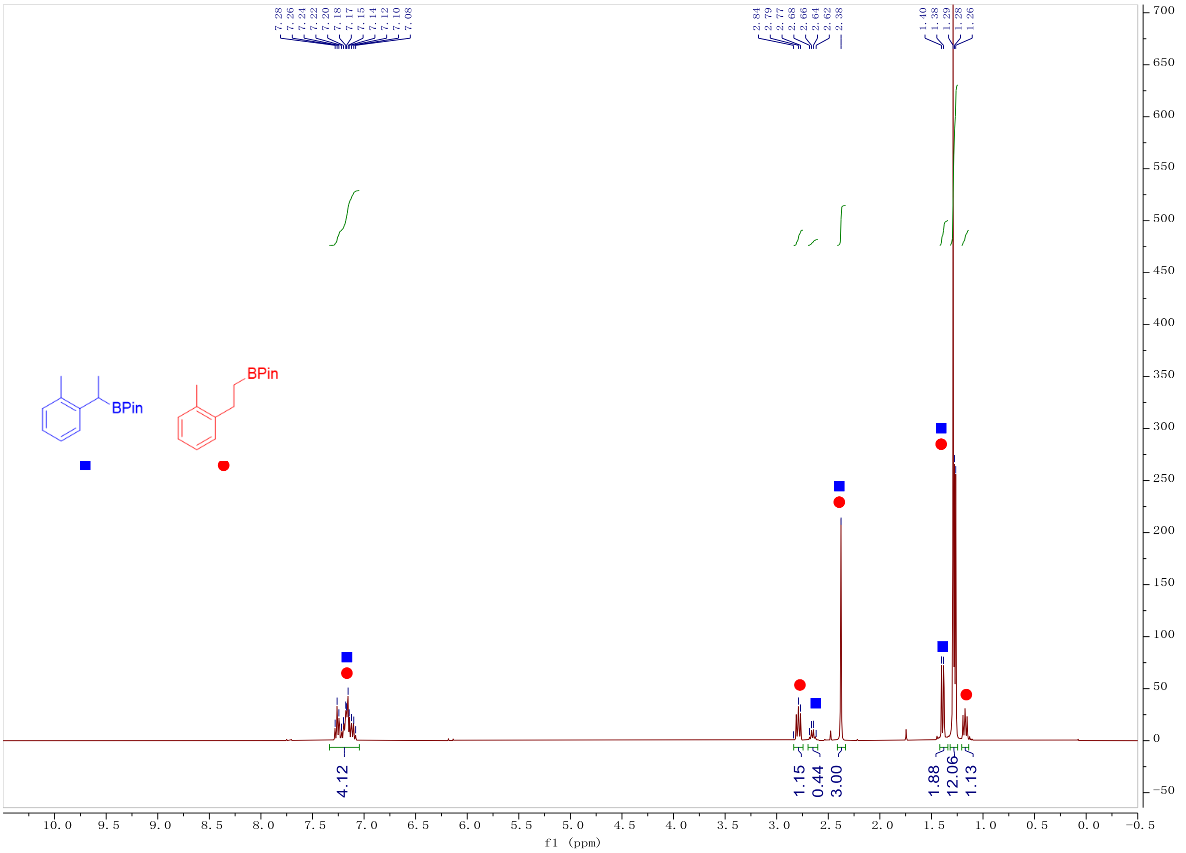
7.28 7.26 7.24 7.22 7.20 7.18 7.17 7.15 7.14 7.12 7.10 7.08
2.84 2.79 2.77 2.68 2.66 2.64 2.62 2.38
1.40 1.38 1.29 1.28 1.26
BPin
BPin
4.12
1.15
0.44
3.00
1.88
12.06
1.13
f1 (ppm)

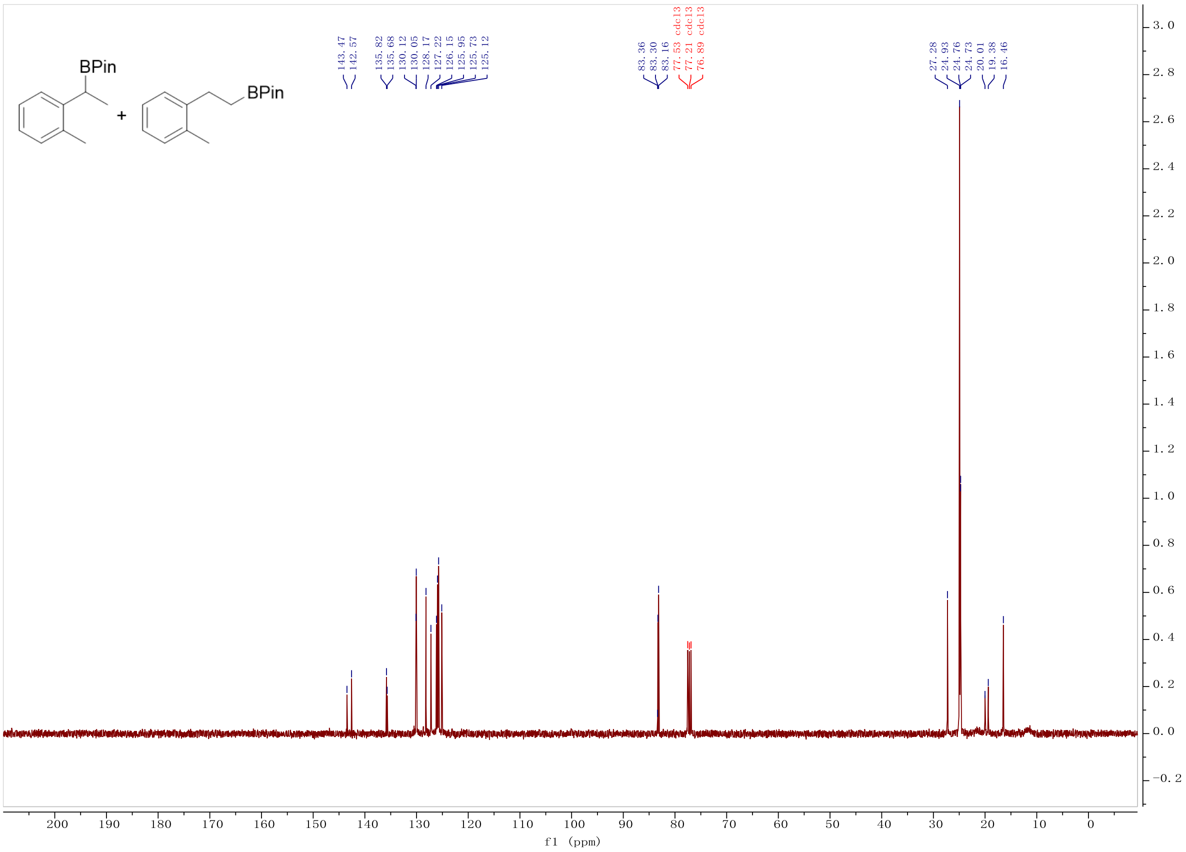
BPin
+
BPin
143.47 142.57 135.82 135.68 130.12 130.05 128.17 127.22 126.15 125.95 125.73 125.12
83.36 83.30 83.16 77.53 cdcl3 77.21 cdcl3 76.89 cdcl3
27.28 24.93 24.76 24.73 20.01 19.38 16.46
f1 (ppm)

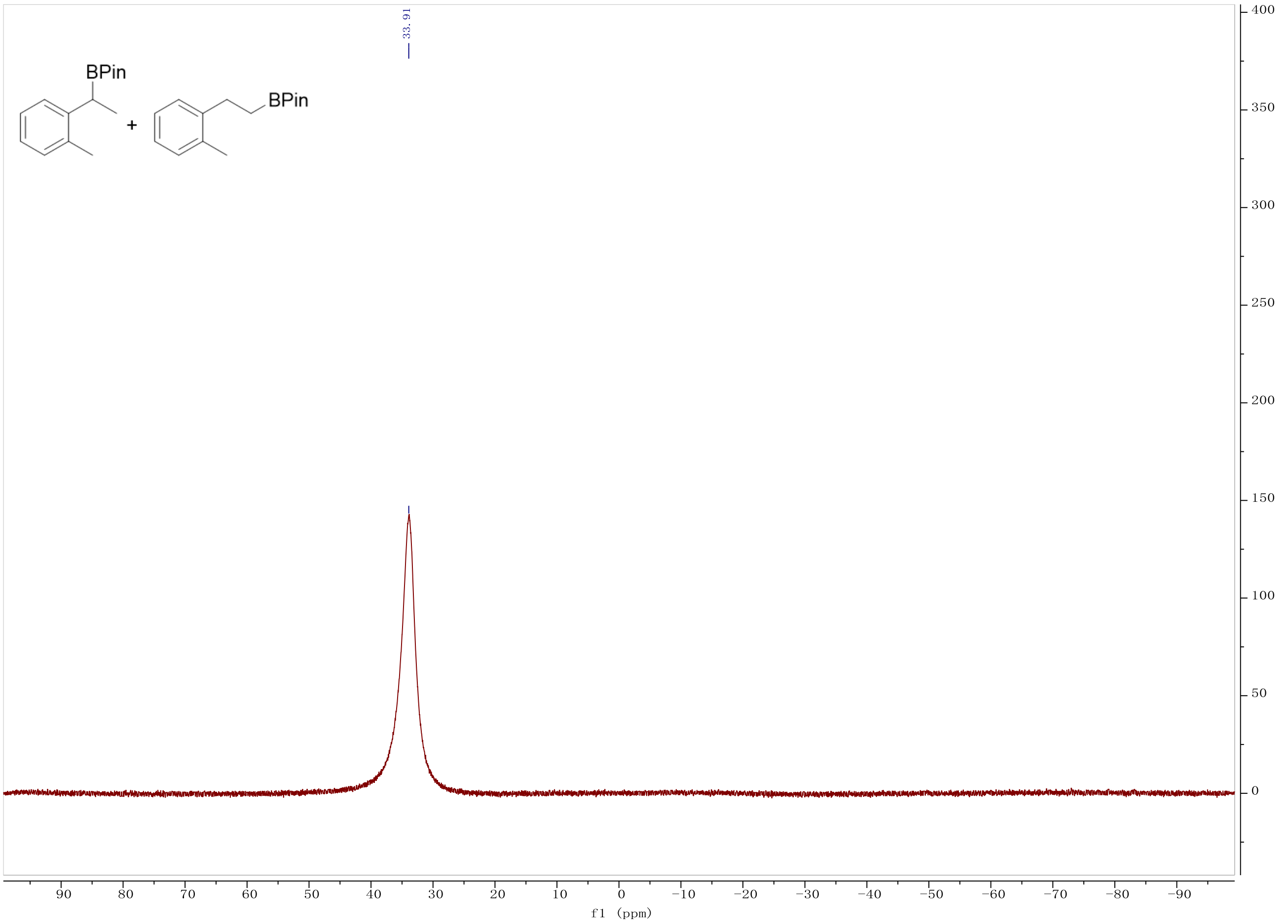

Supplementary Figure 13. ^{1}**H**, ^{13}**C**, ^{11}**B NMR** of the mixture of compound **2e & 3e**

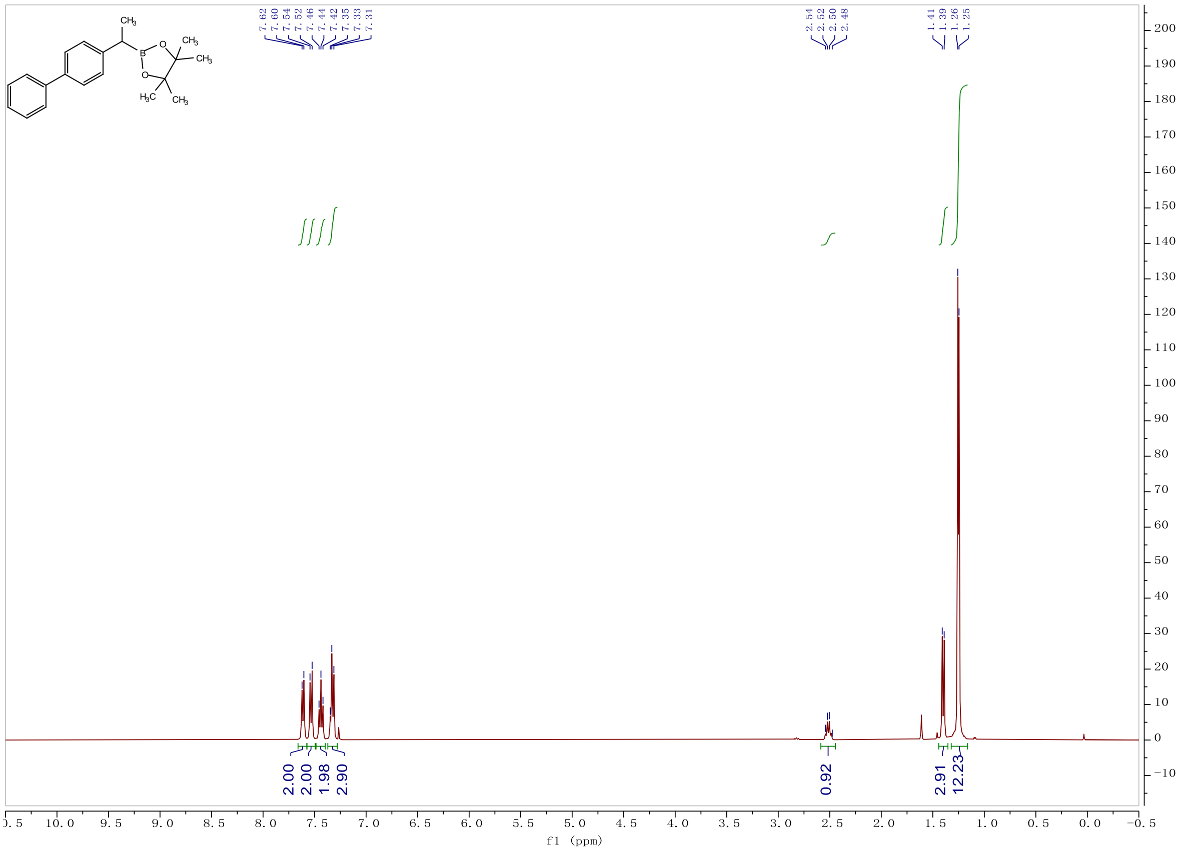

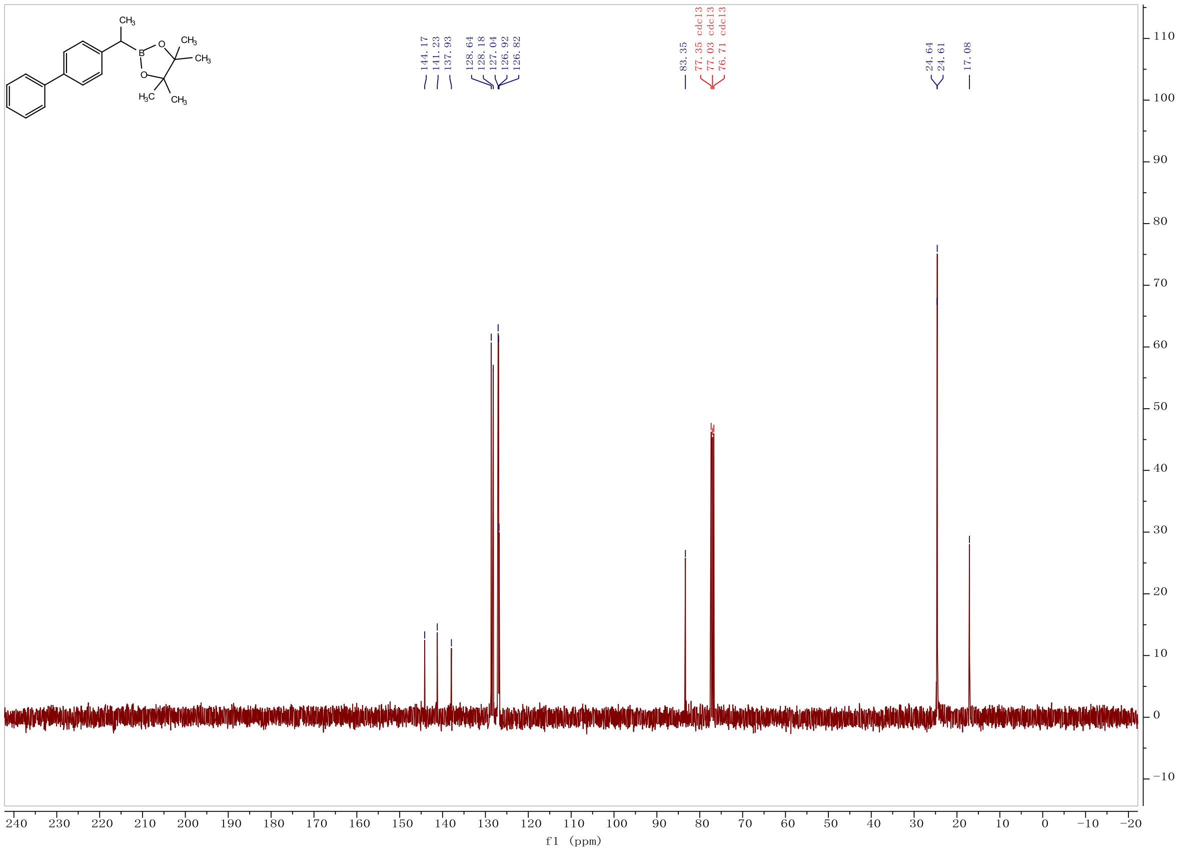

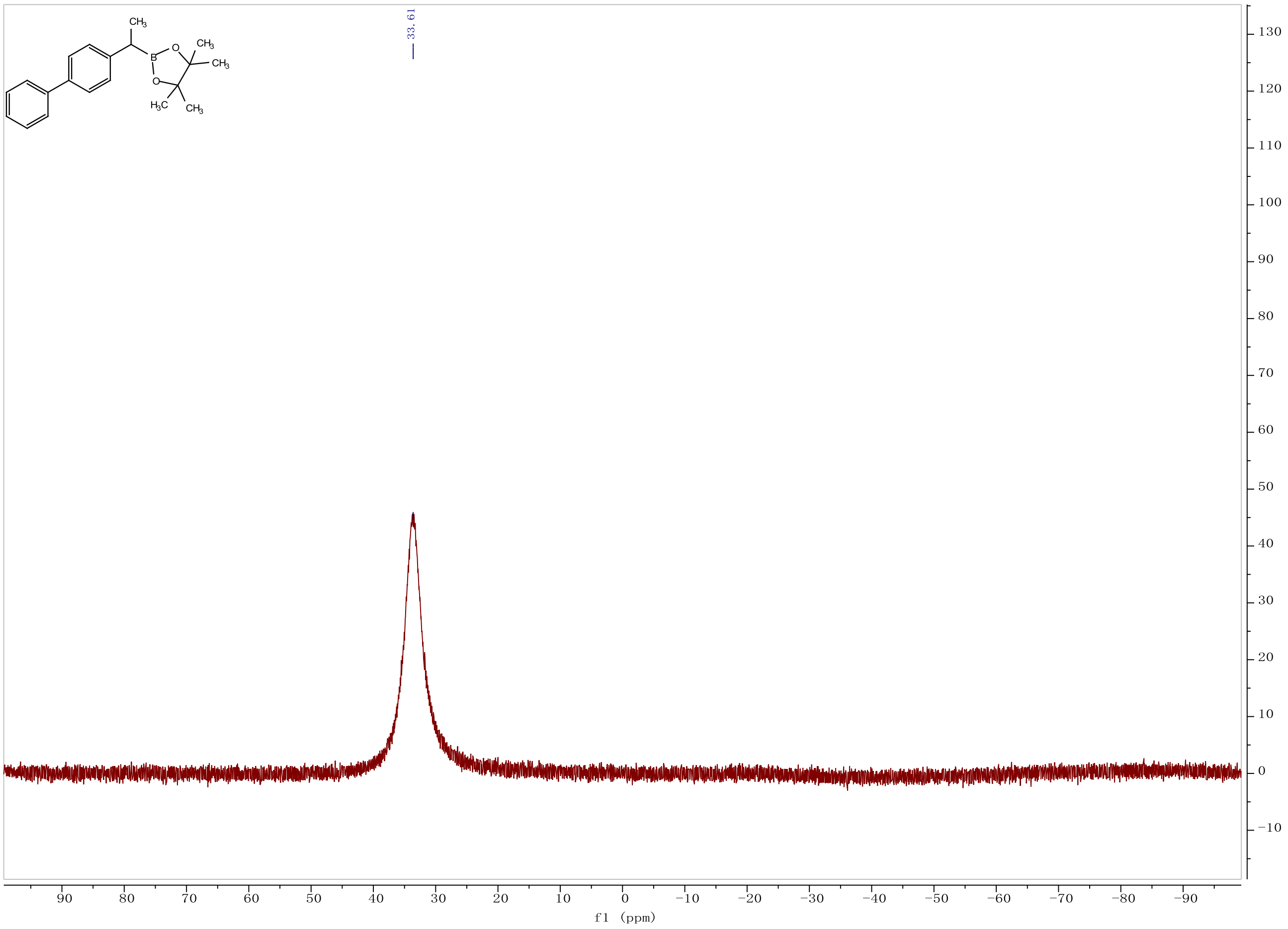

Supplementary Figure 14. ^{1}**H**, ^{13}**C**, ^{11}**B NMR** of compound **2f**.

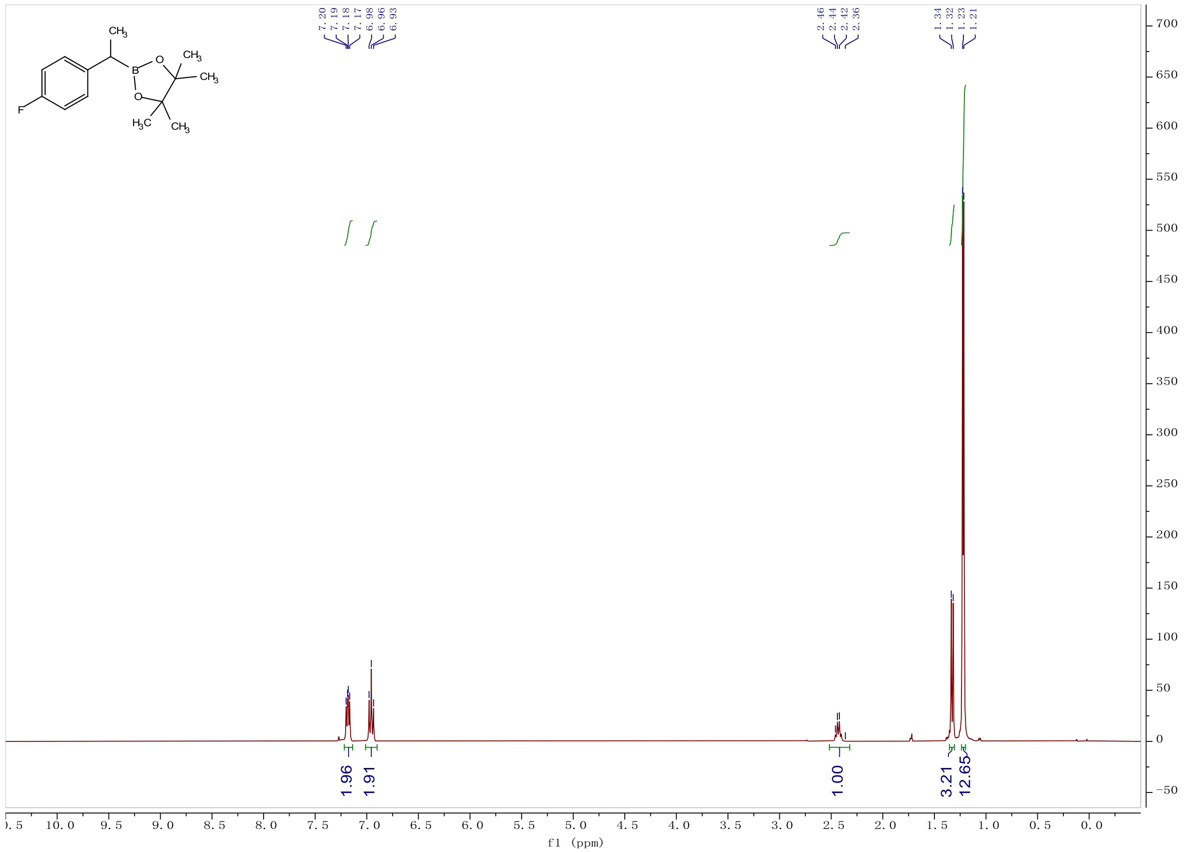
7.20
7.19
7.18
7.17
6.98
6.96
6.93
2.46
2.44
2.42
2.36
1.34
1.32
1.23
1.21
1.96
1.91
1.00
3.21
12.65
f1 (ppm)

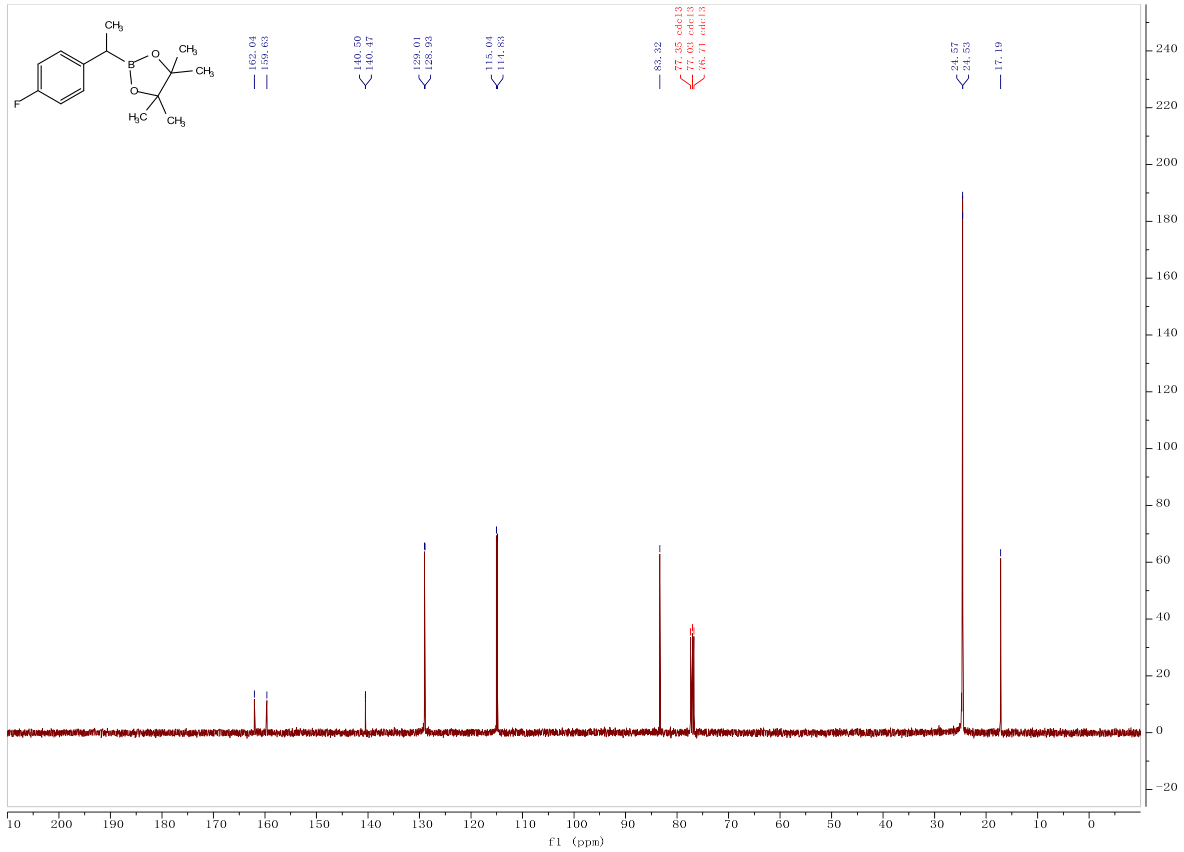
162.04
159.63
140.50
140.47
129.01
128.93
115.04
114.83
83.32
77.35 cdcl3
77.03 cdcl3
76.71 cdcl3
24.57
24.53
17.19
f1 (ppm)

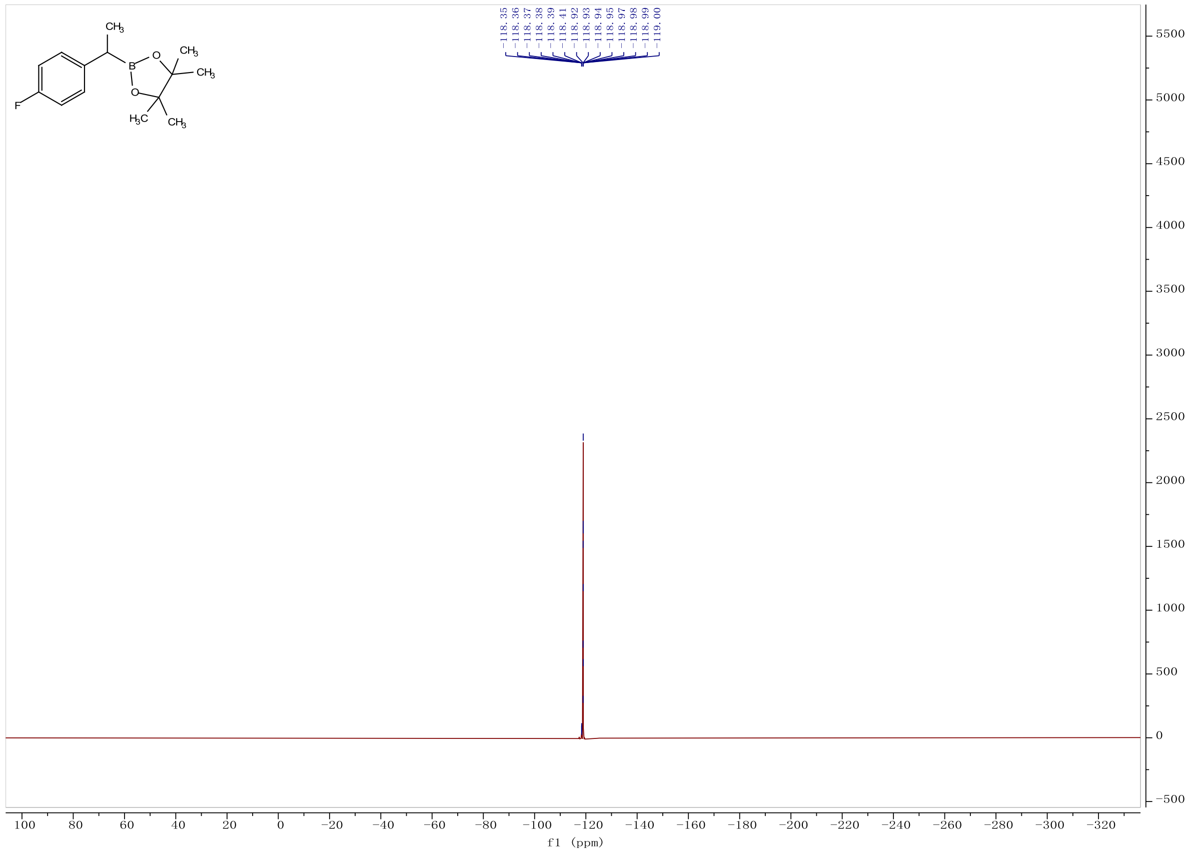

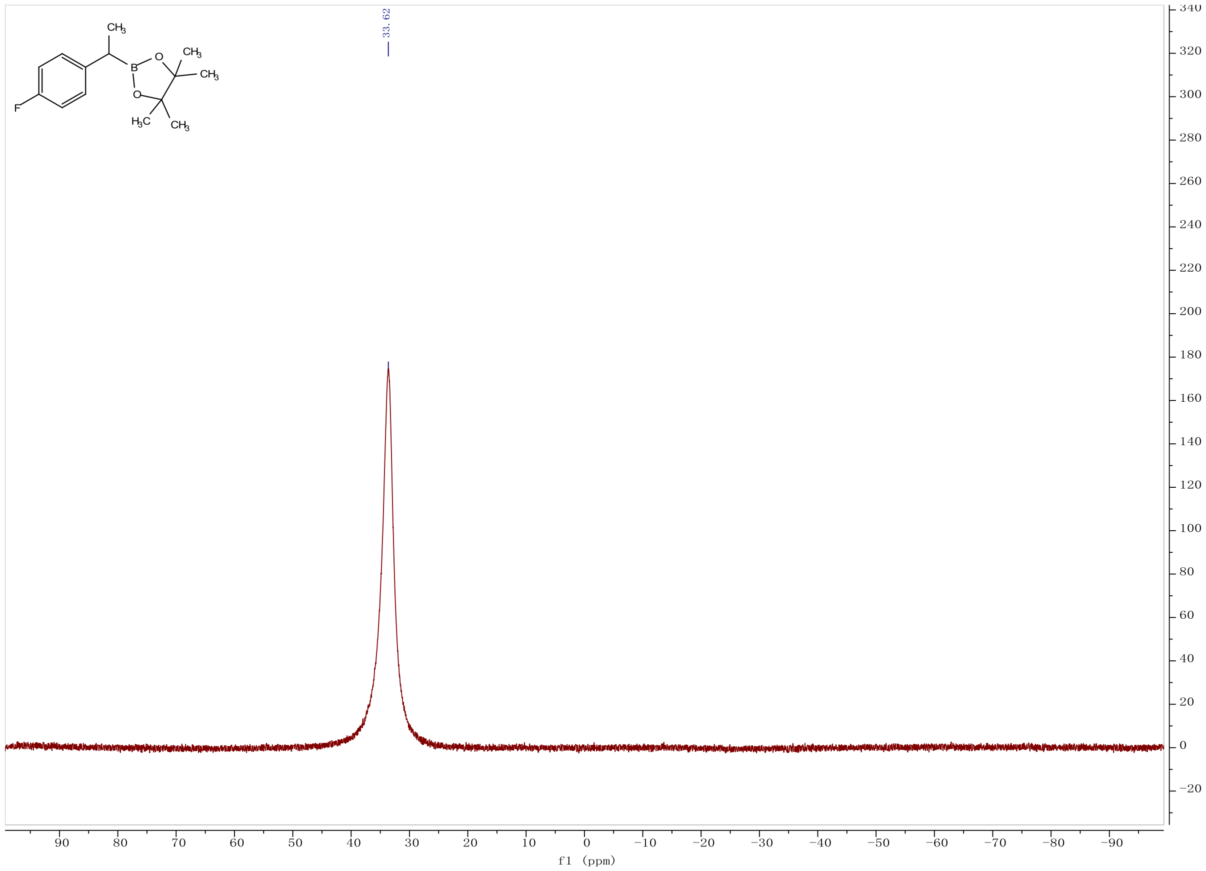

Supplementary Figure 15. ^{1}H, ^{13}C, ^{19}F, ^{11}B NMR of compound **2g**.

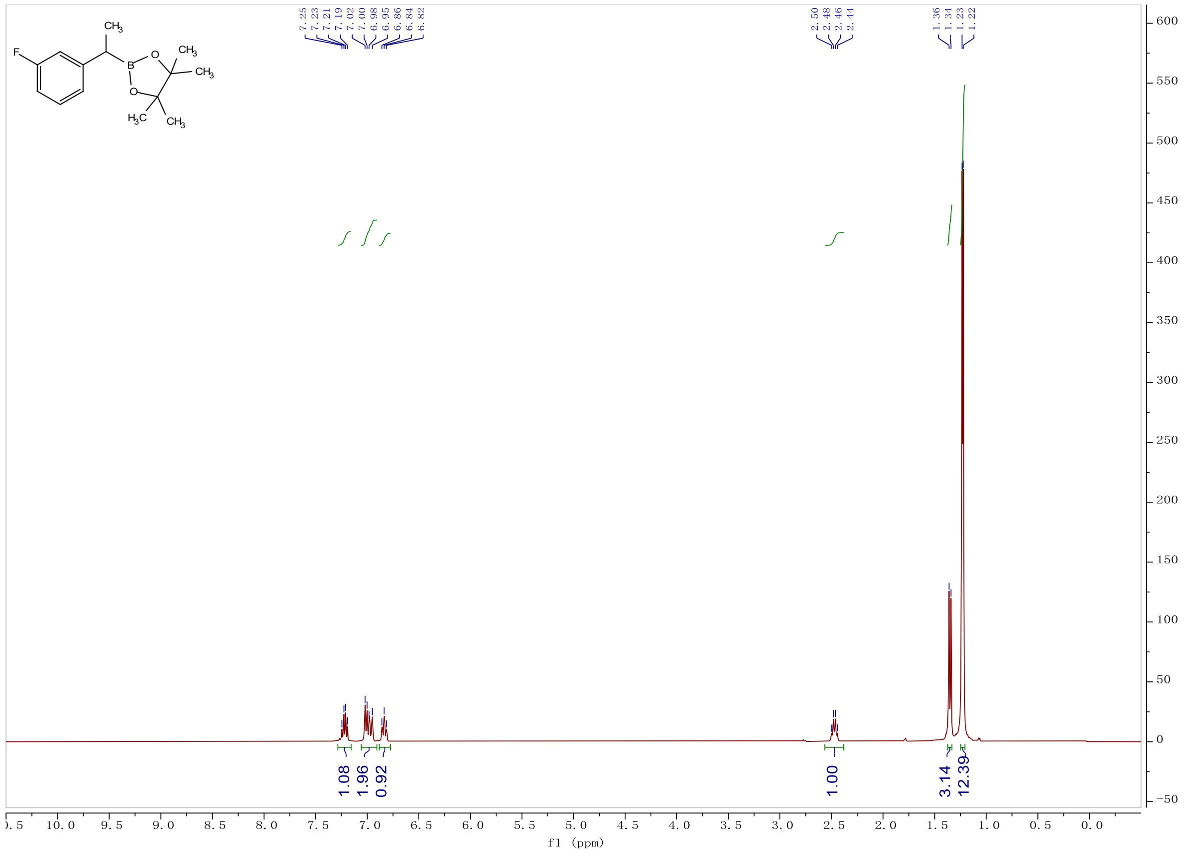

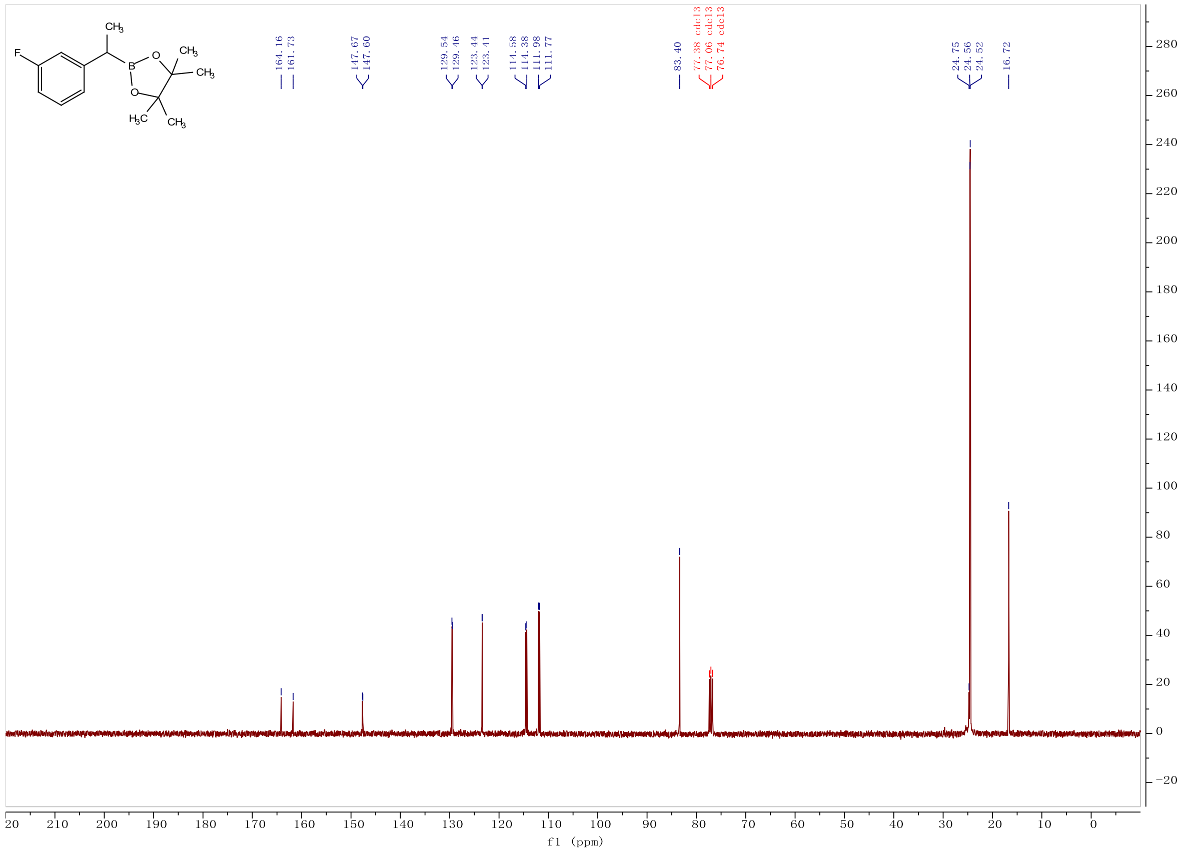

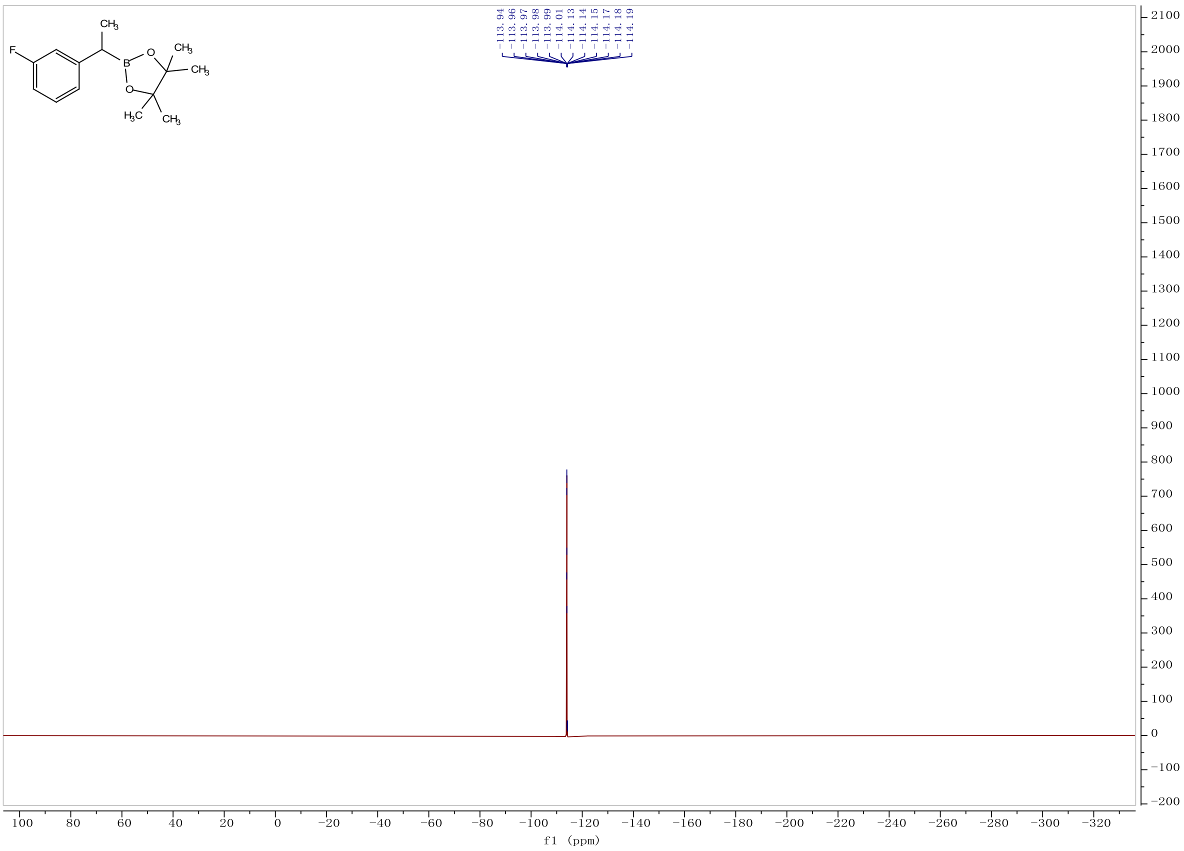

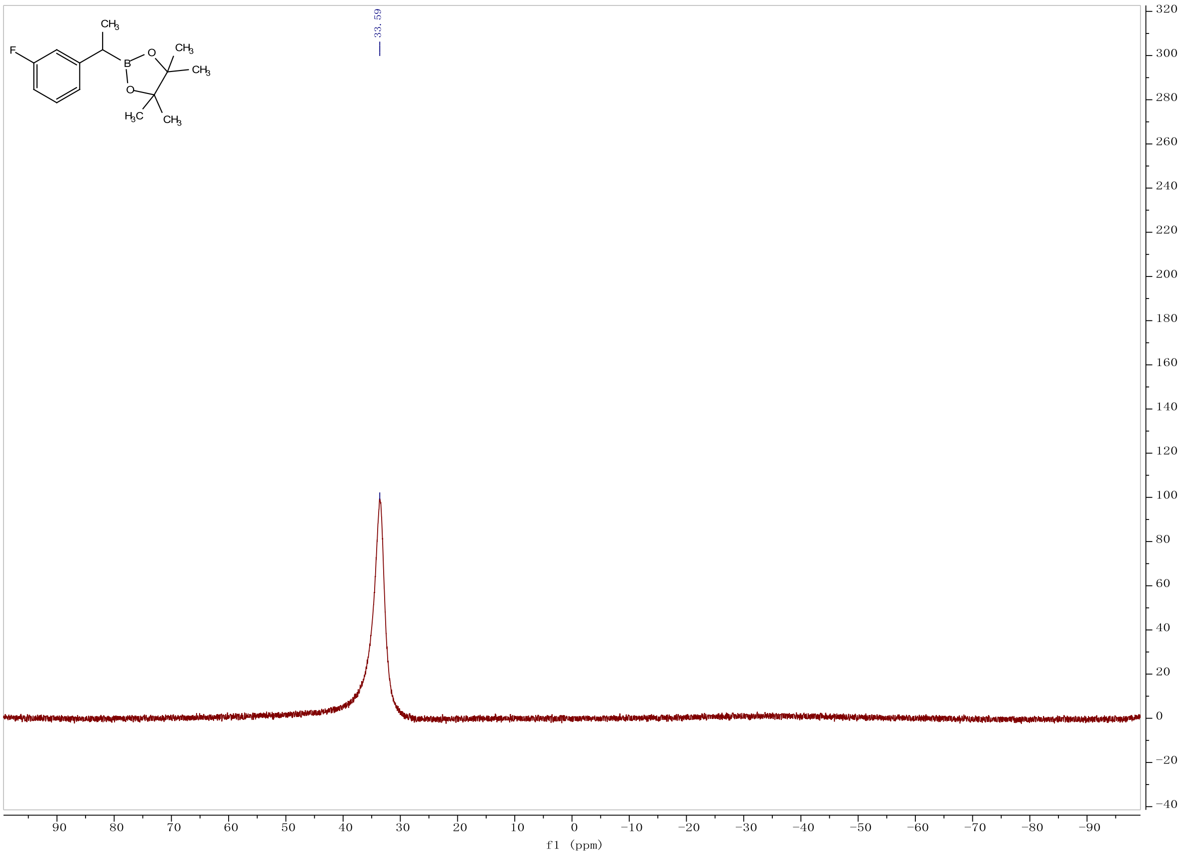

Supplementary Figure 16. ^{1}**H**, ^{13}**C,** ^{19}**F,** ^{11}**B NMR** of compound **2h**.

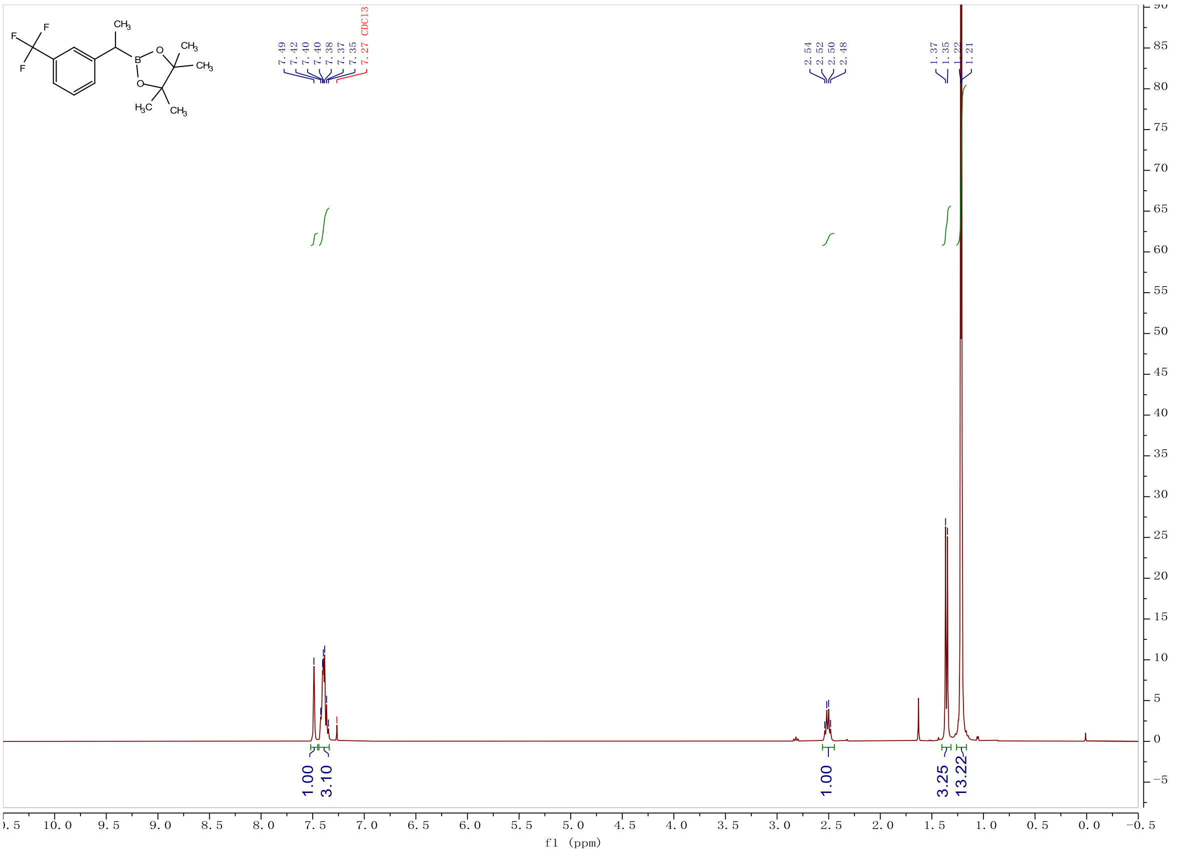
7.49
7.42
7.40
7.40
7.38
7.37
7.35
7.27 CDCl3
2.54
2.52
2.50
2.48
1.37
1.35
1.22
1.21
1.00
3.10
1.00
3.25
13.22
f1 (ppm)

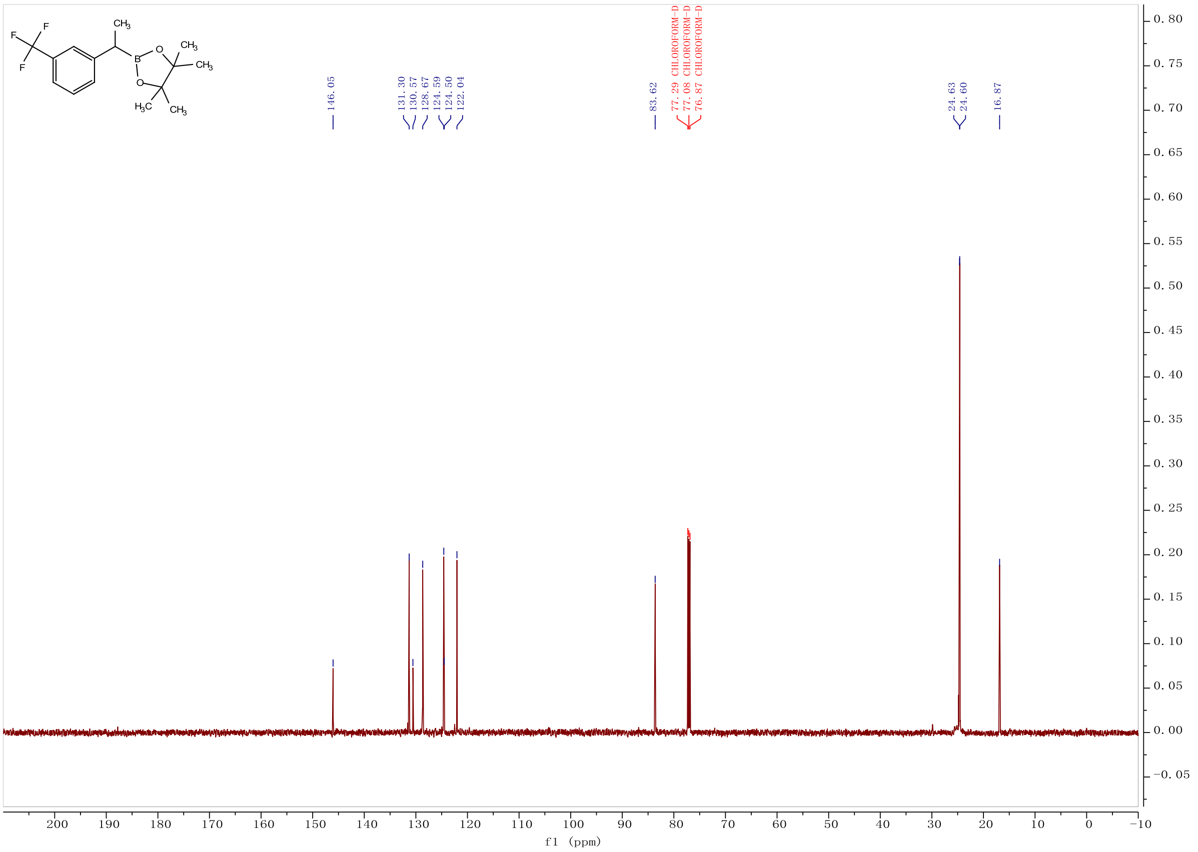
146.05
131.30
130.57
128.67
124.59
124.50
122.04
83.62
77.29 CHLOROFORM-D
77.08 CHLOROFORM-D
76.87 CHLOROFORM-D
24.63
24.60
16.87
f1 (ppm)

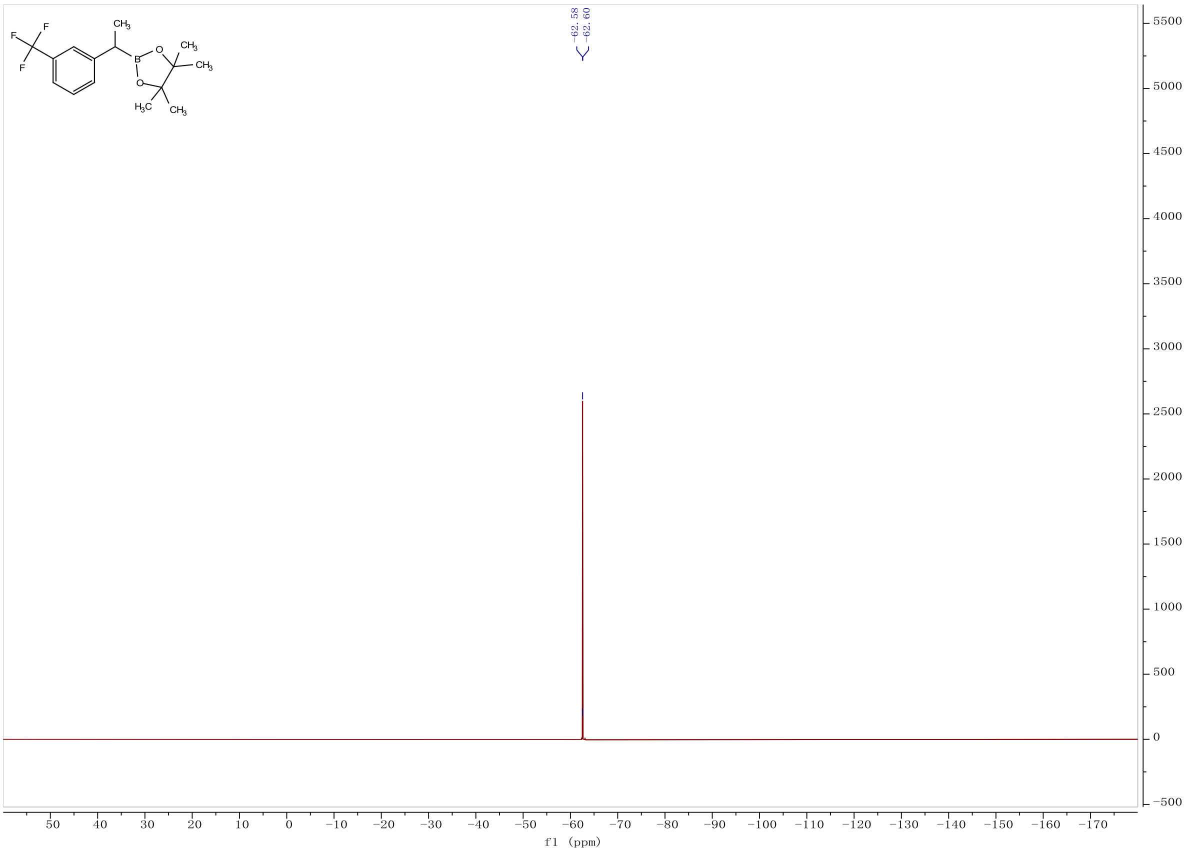

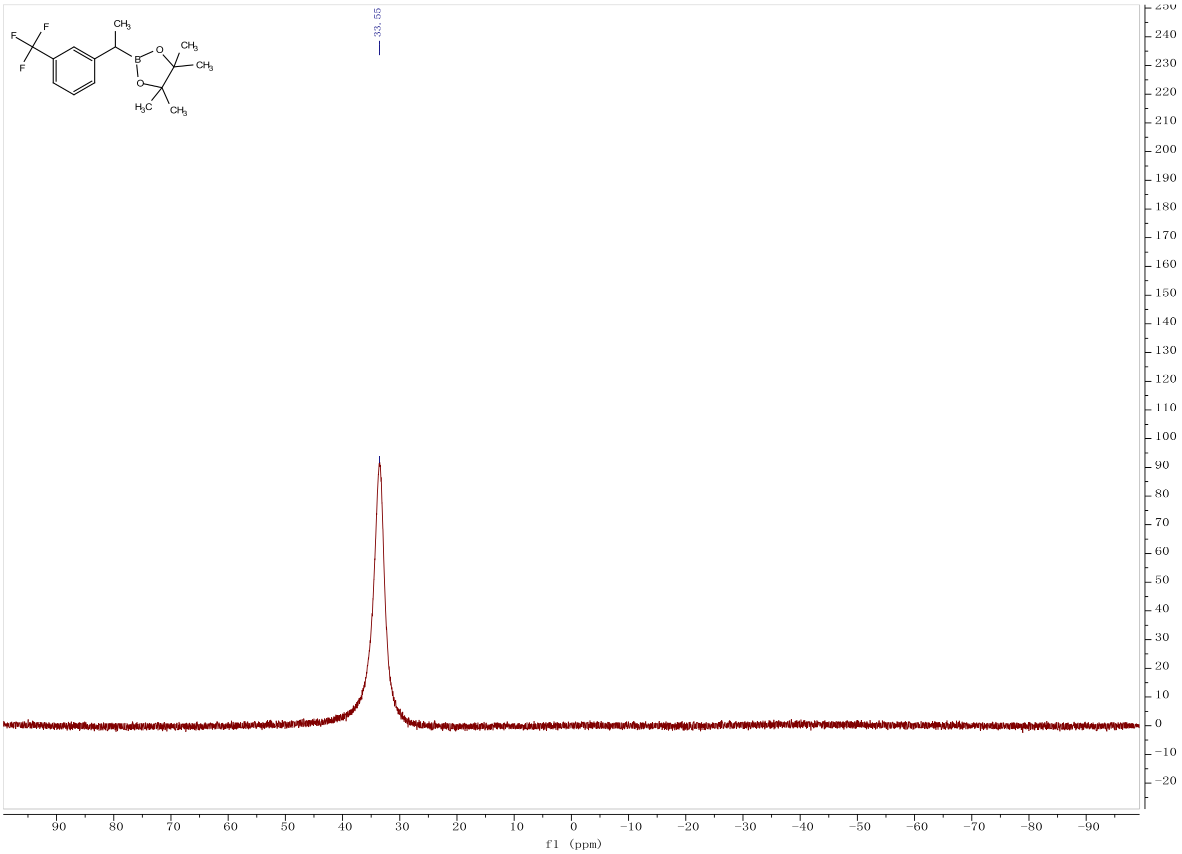

Supplementary Figure 17. ^{1}H, **^{13}C, ^{19}F, ^{11}B NMR** of Compound **2i**.

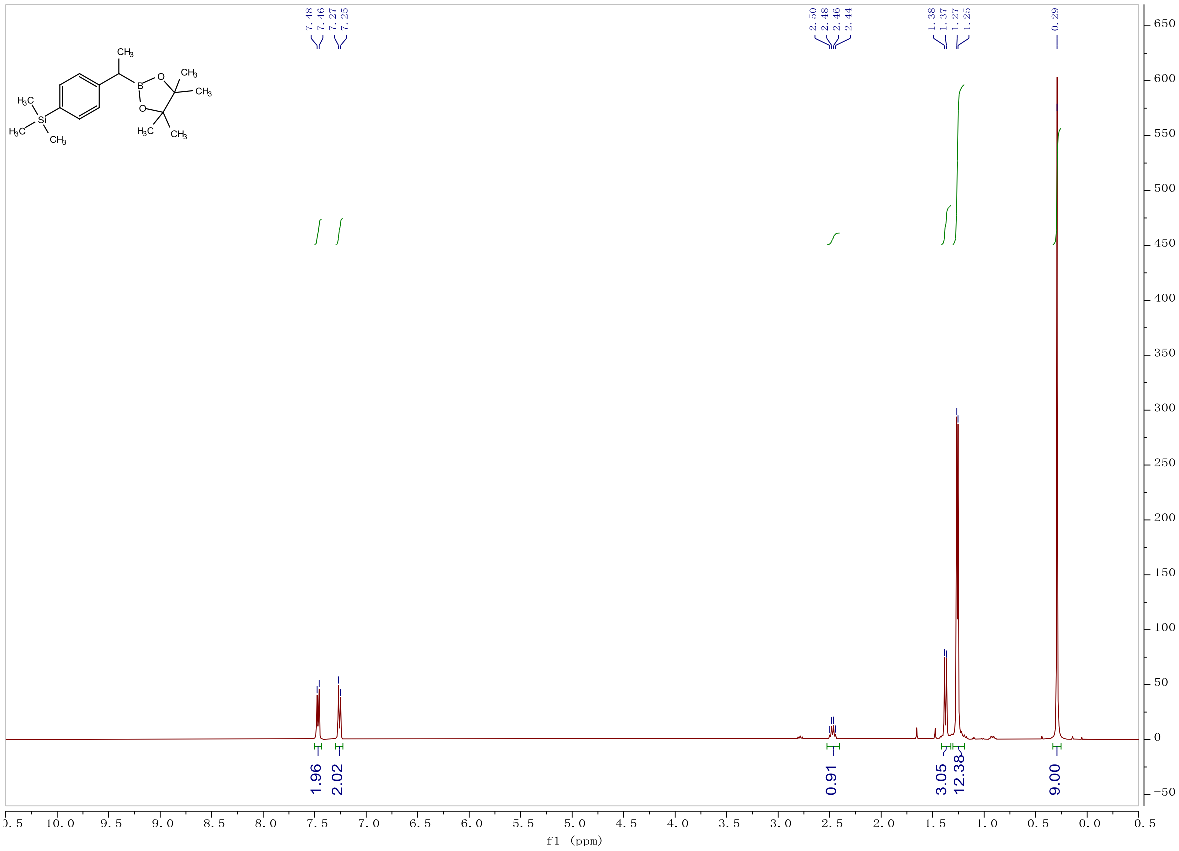

7.48
7.46
7.27
7.25
2.50
2.48
2.46
2.44
1.38
1.37
1.27
1.25
0.29
1.96
2.02
0.91
3.05
12.38
9.00
f1 (ppm)

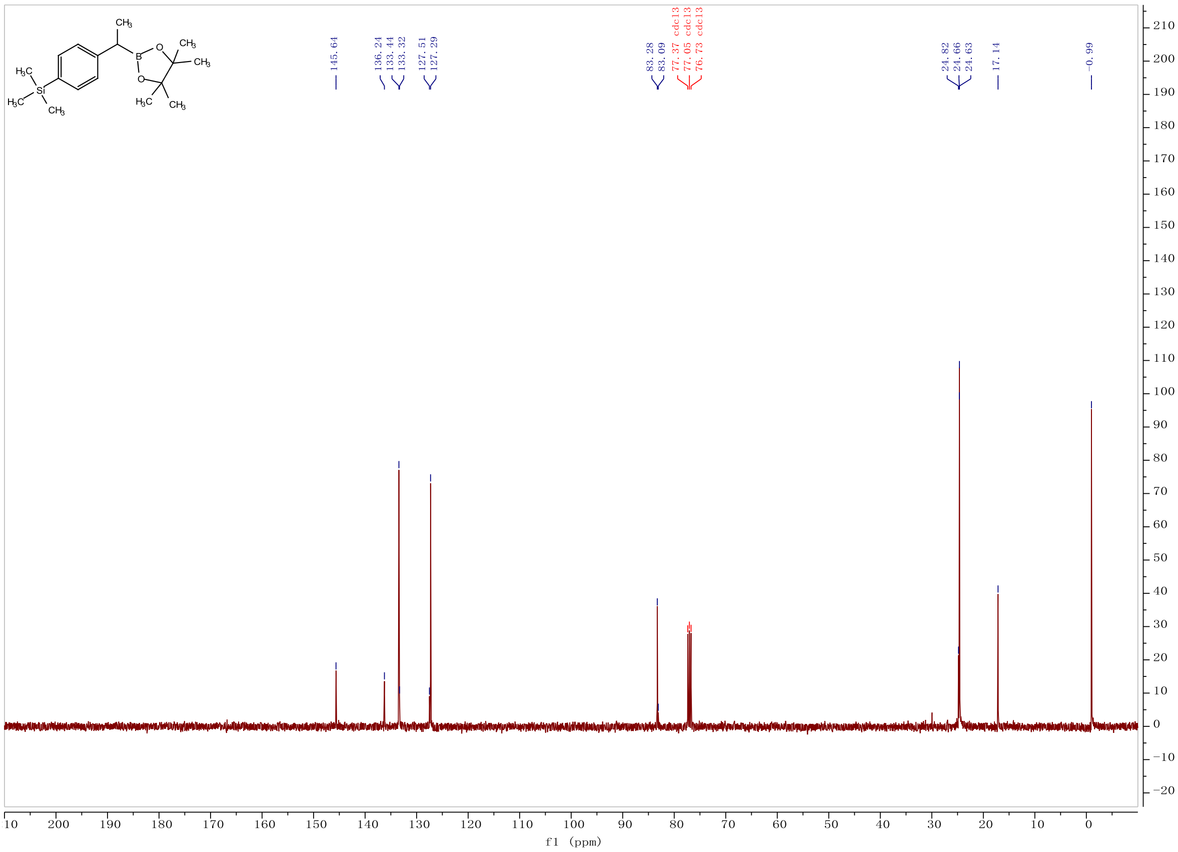

145.64
136.24
133.44
133.32
127.51
127.29
83.28
83.09
77.37 cdcl3
77.05 cdcl3
76.73 cdcl3
24.82
24.66
24.63
17.14
-0.99
f1 (ppm)

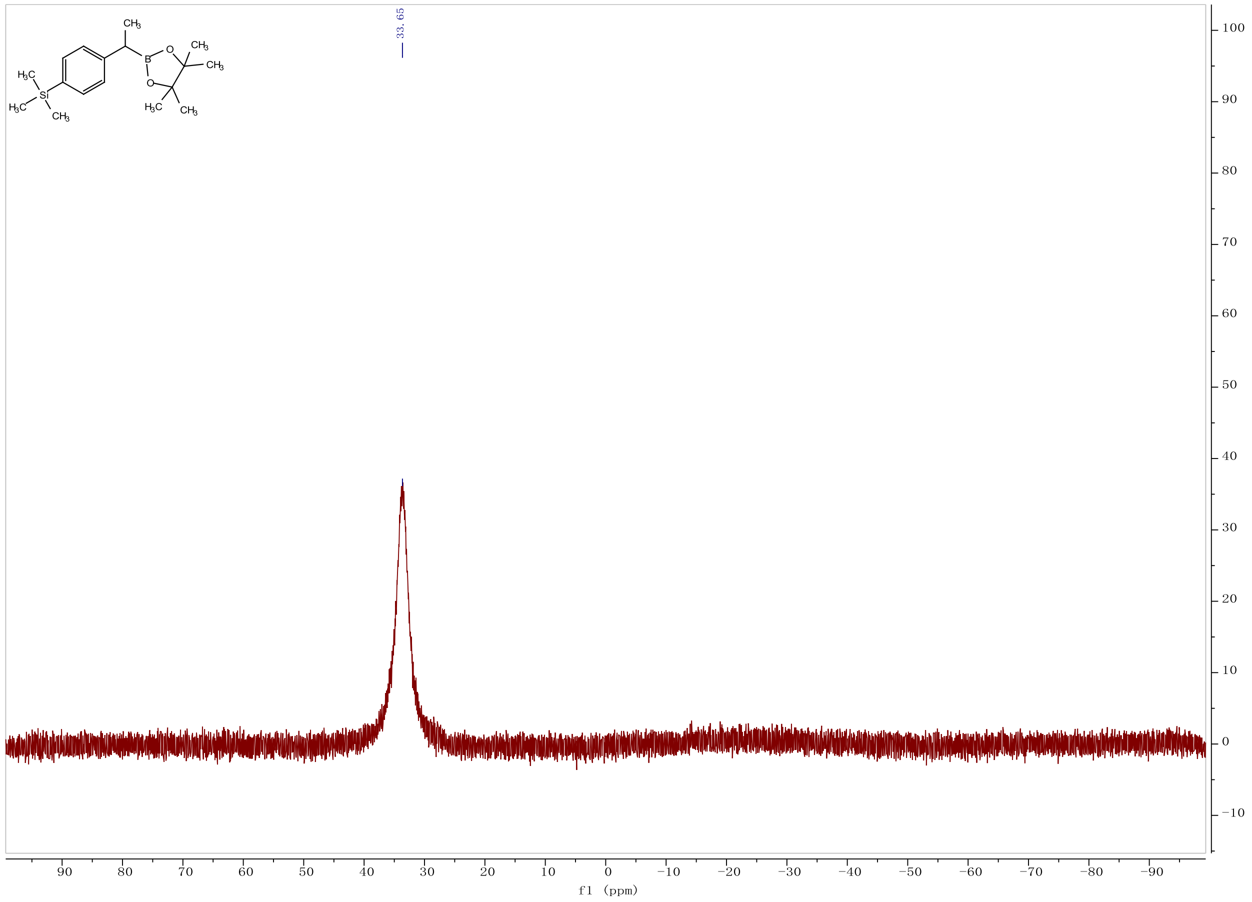

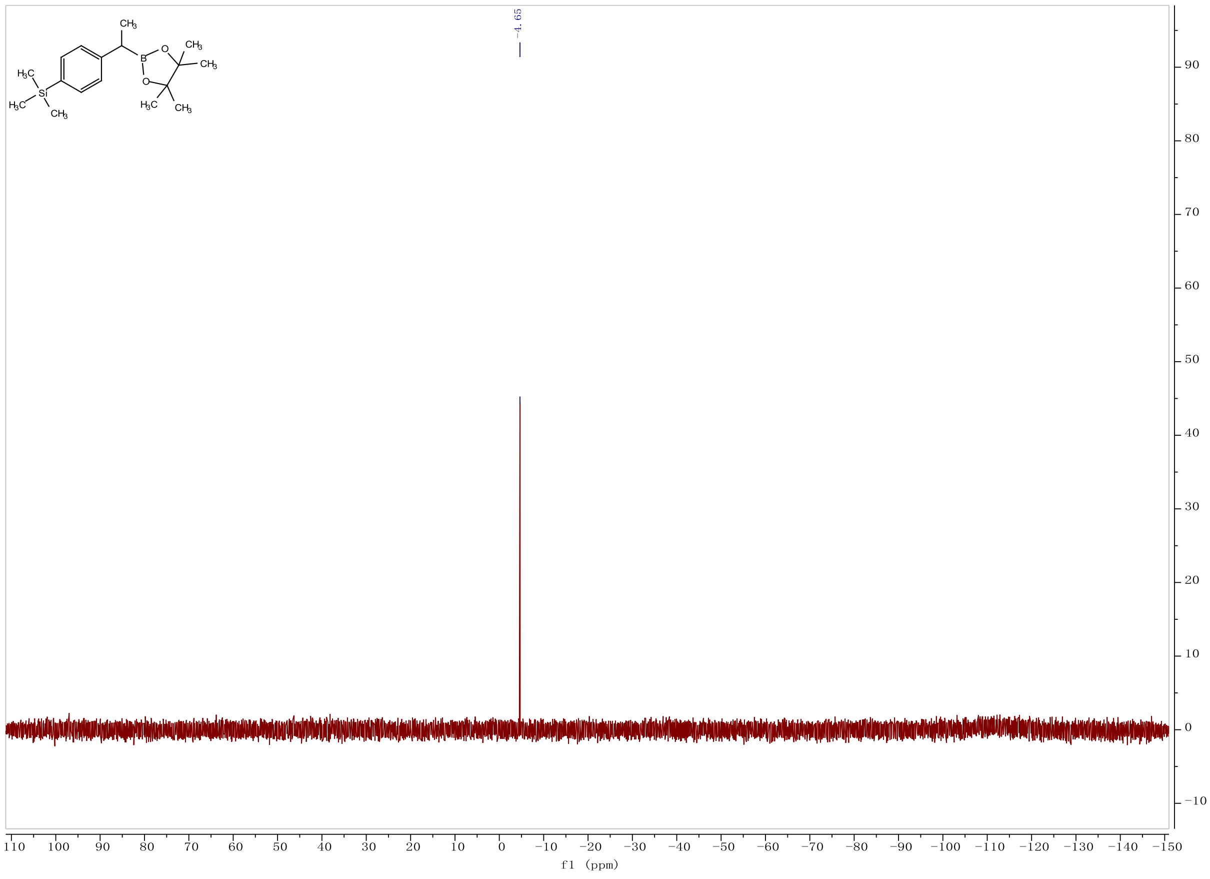

Supplementary Figure 18. ^{1}H, ^{13}C, ^{11}B , ^{29}Si NMR of compound **2j**.

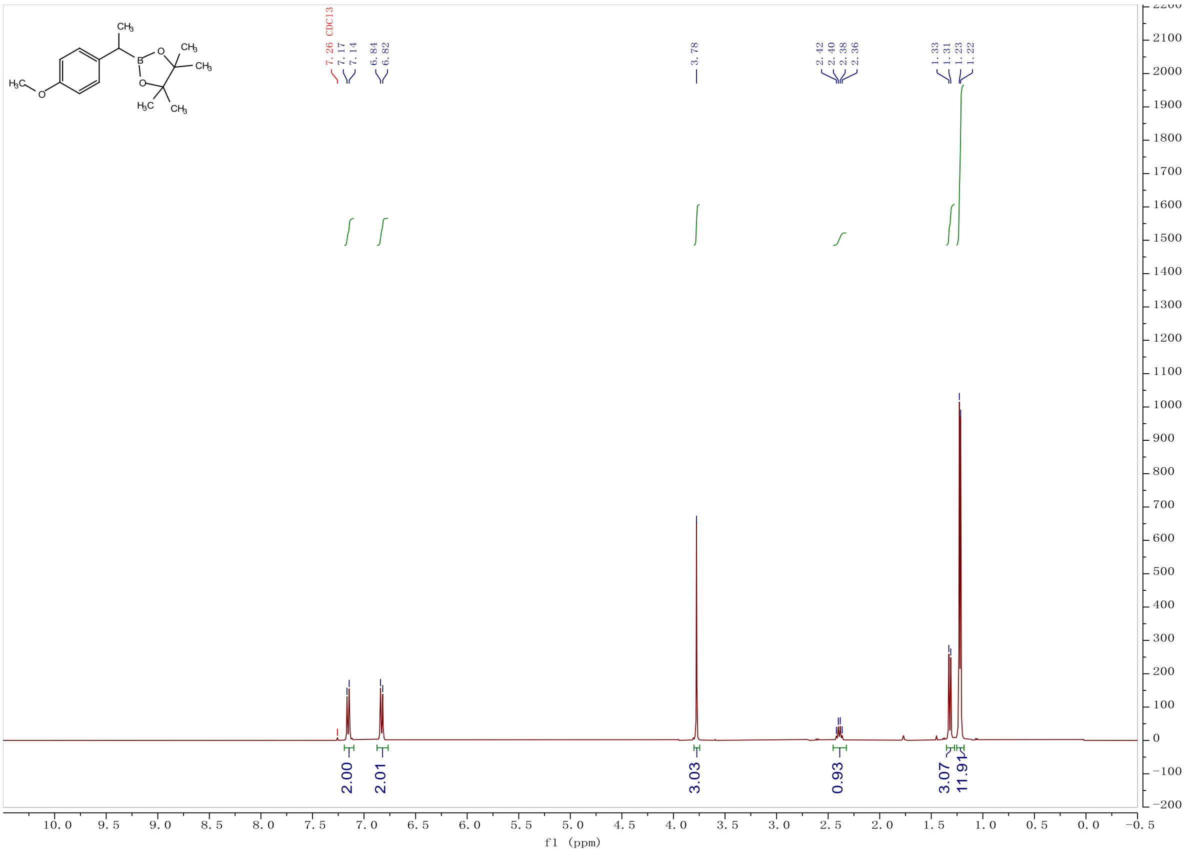
7.26 CDCl3
7.17
7.14
6.84
6.82
3.78
2.42
2.40
2.38
2.36
1.33
1.31
1.23
1.22
2.00
2.01
3.03
0.93
3.07
11.91
f1 (ppm)

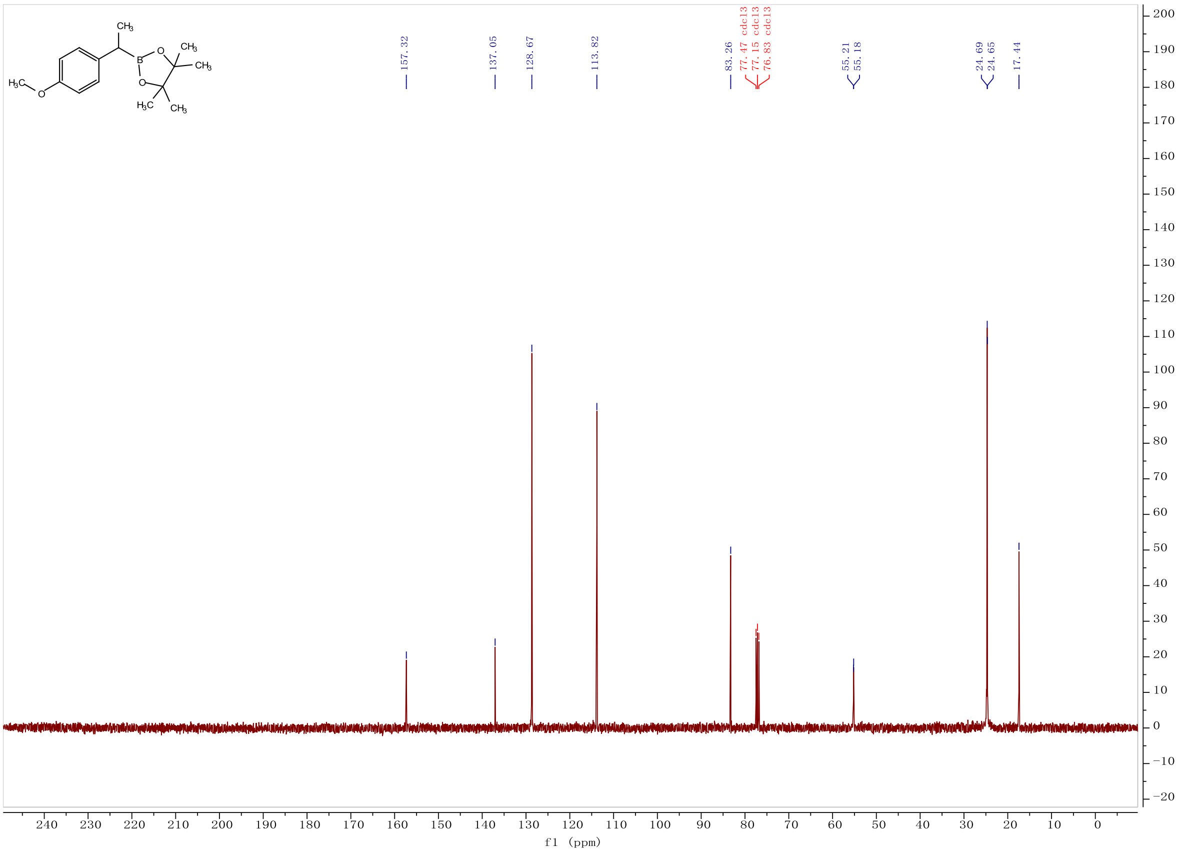
157.32
137.05
128.67
113.82
83.26
77.47 cdcl3
77.15 cdcl3
76.83 cdcl3
55.21
55.18
24.69
24.65
17.44
f1 (ppm)

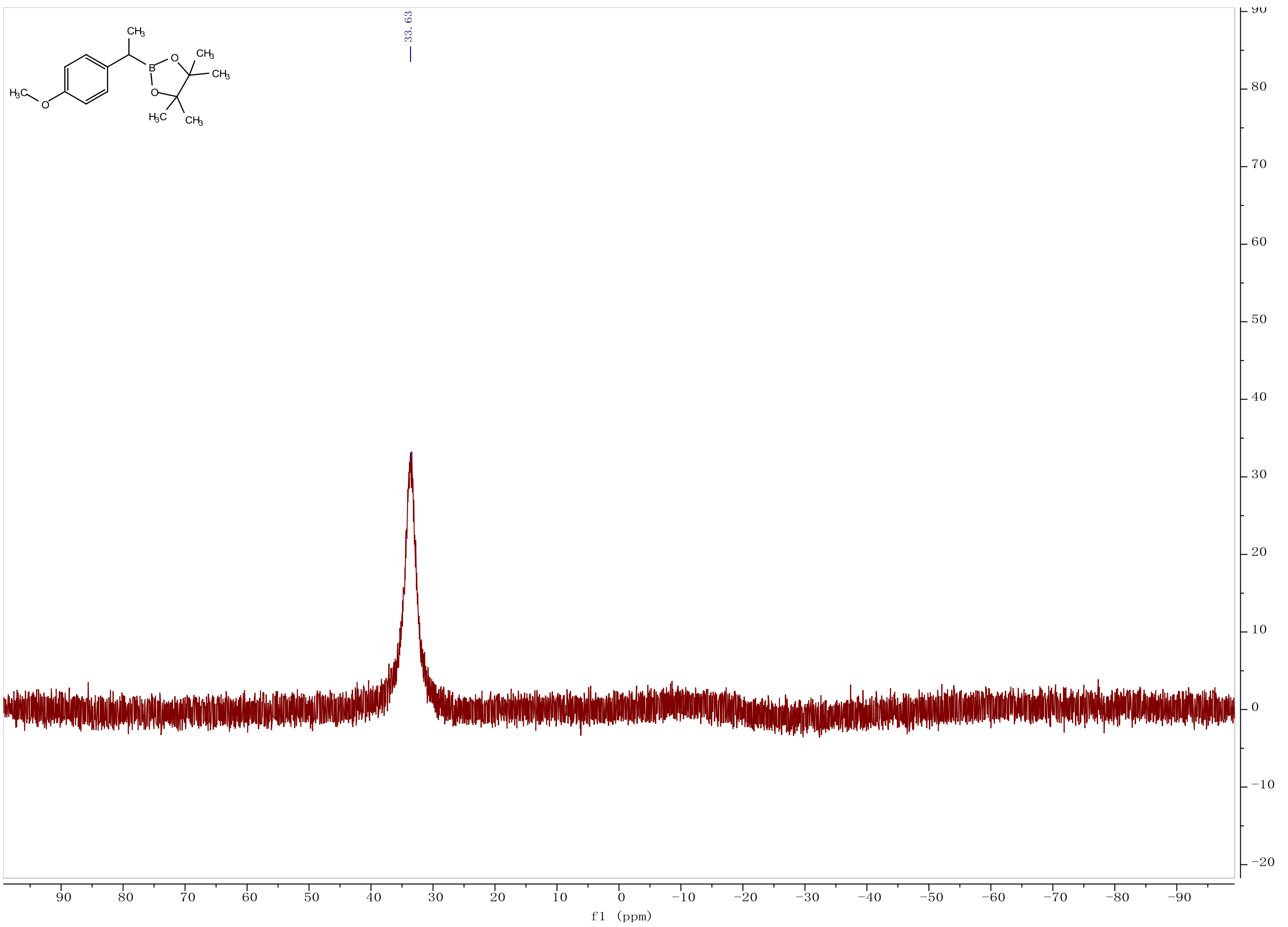

Supplementary Figure 19. ^{1}**H,** ^{13}**C,** ^{11}**B NMR** of compound **2k**.

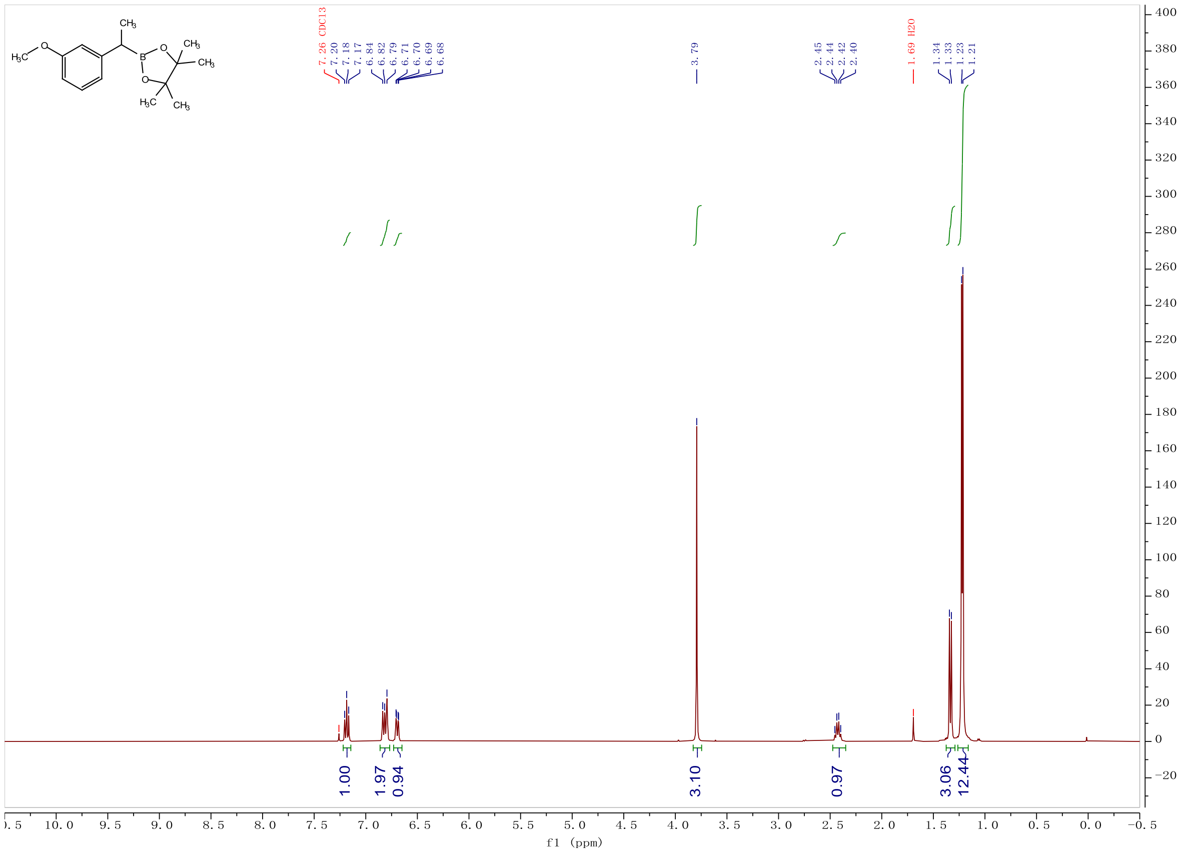

7.26 CDCl3
7.20
7.18
7.17
6.84
6.82
6.79
6.71
6.70
6.69
6.68
3.79
2.45
2.44
2.42
2.40
1.69 H2O
1.34
1.33
1.23
1.21
1.00
1.97
0.94
3.10
0.97
3.06
12.44
f1 (ppm)

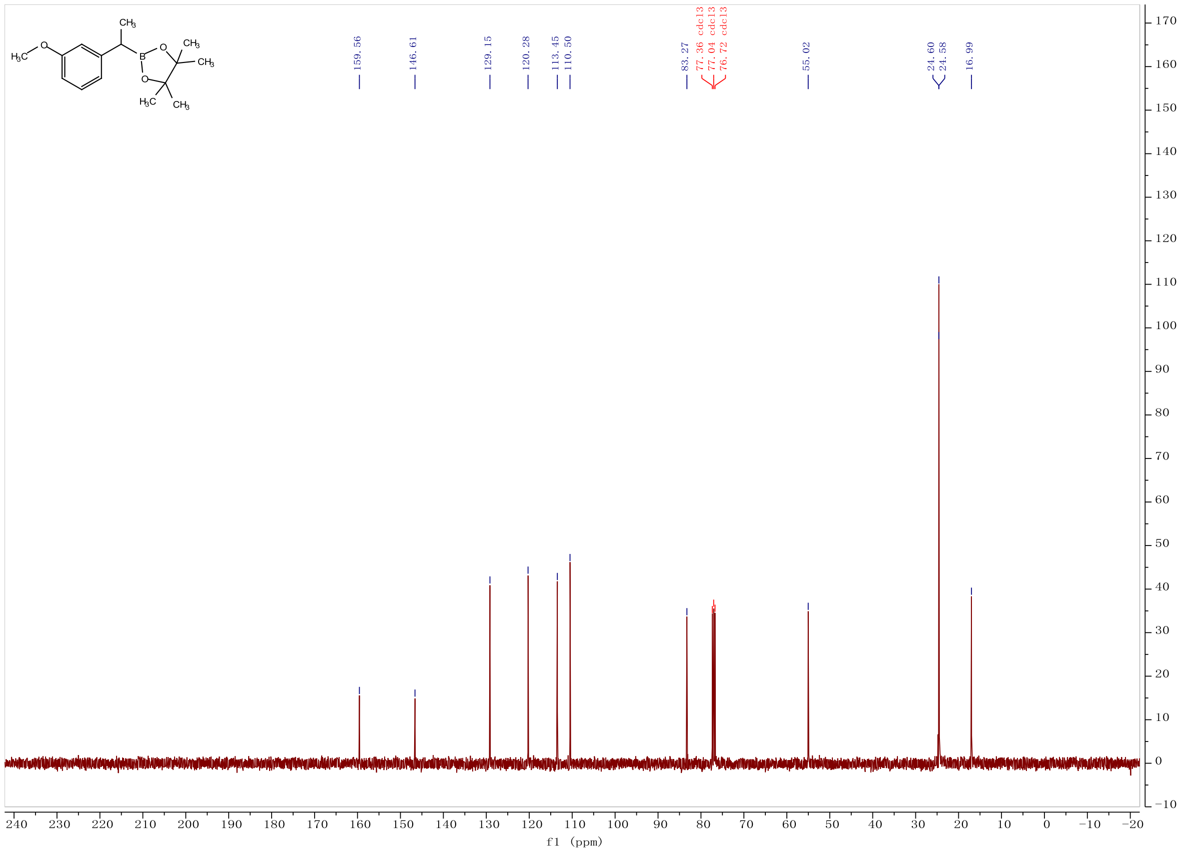

159.56
146.61
129.15
120.28
113.45
110.50
83.27
77.36 cdcl3
77.04 cdcl3
76.72 cdcl3
55.02
24.60
24.58
16.99
f1 (ppm)

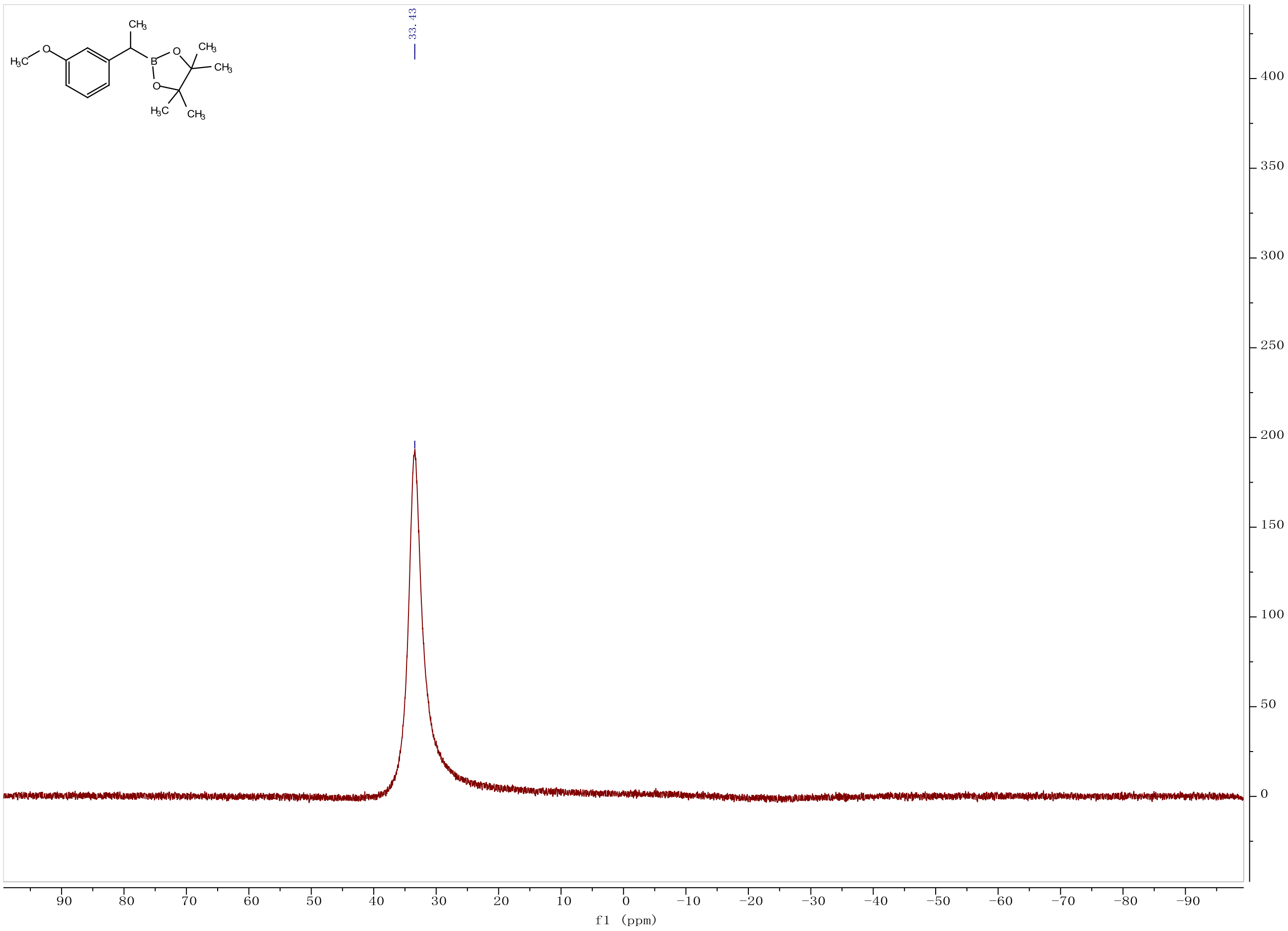

Supplementary Figure 20. **^{1}H**, **^{13}C, ^{11}B NMR** of compound **2l**.

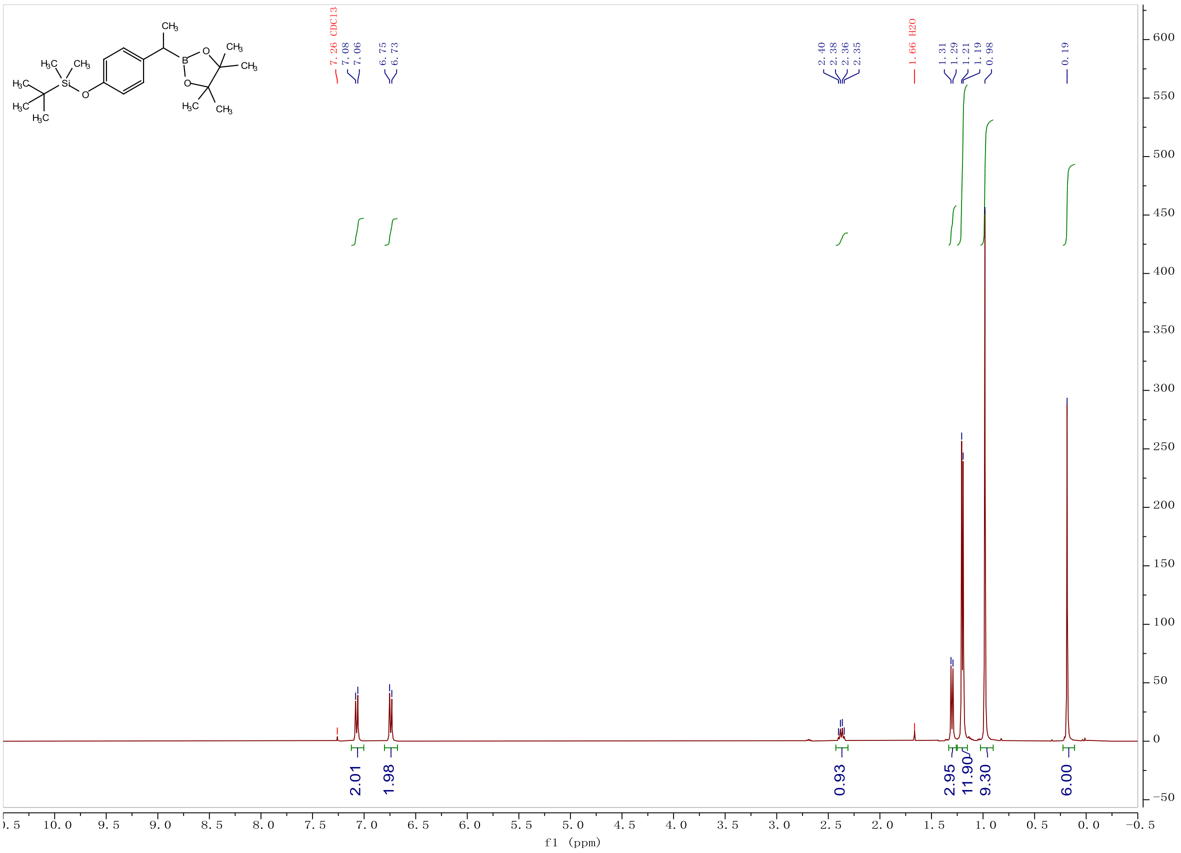
7.26 CDCl3
7.08
7.06
6.75
6.73
2.40
2.38
2.36
2.35
1.66 H2O
1.31
1.29
1.21
1.19
0.98
0.19
2.01
1.98
0.93
2.95
11.90
9.30
6.00
f1 (ppm)

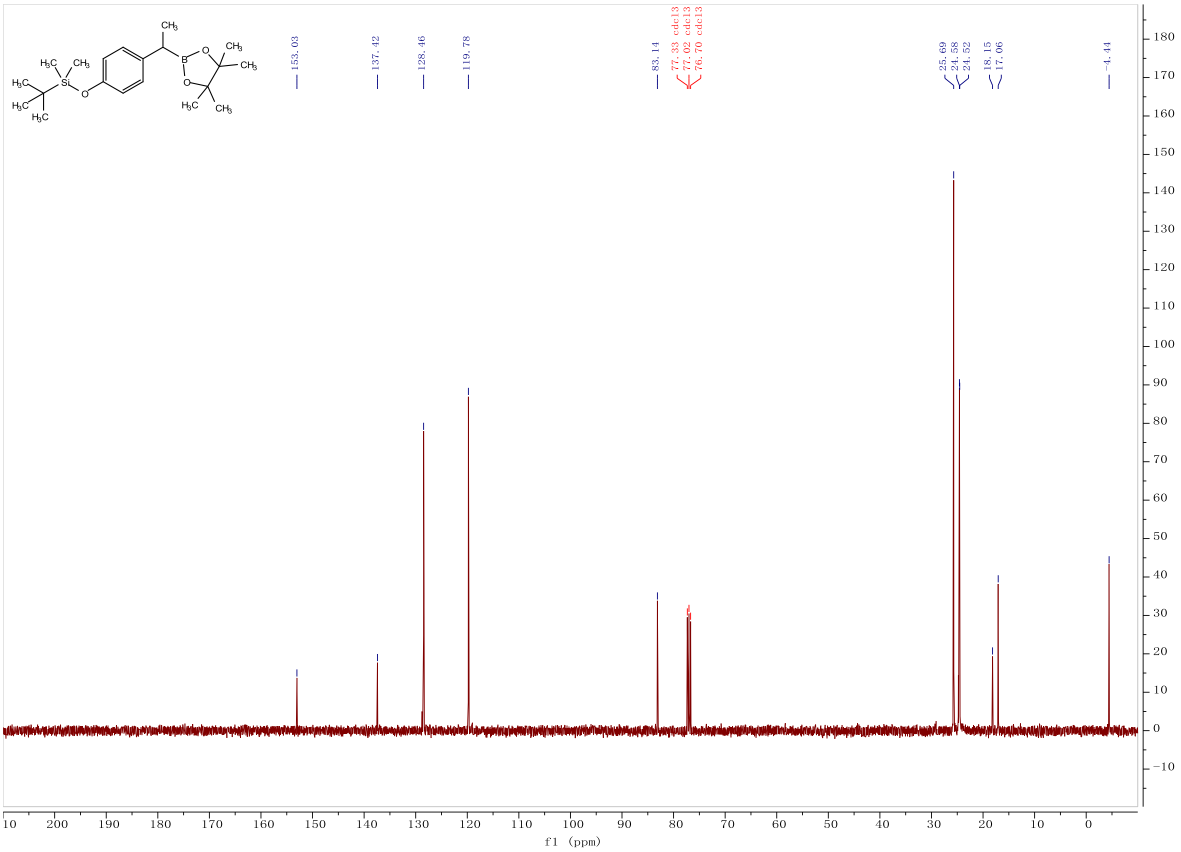
153.03
137.42
128.46
119.78
83.14
77.33 cdcl3
77.02 cdcl3
76.70 cdcl3
25.69
24.58
24.52
18.15
17.06
-4.44
f1 (ppm)

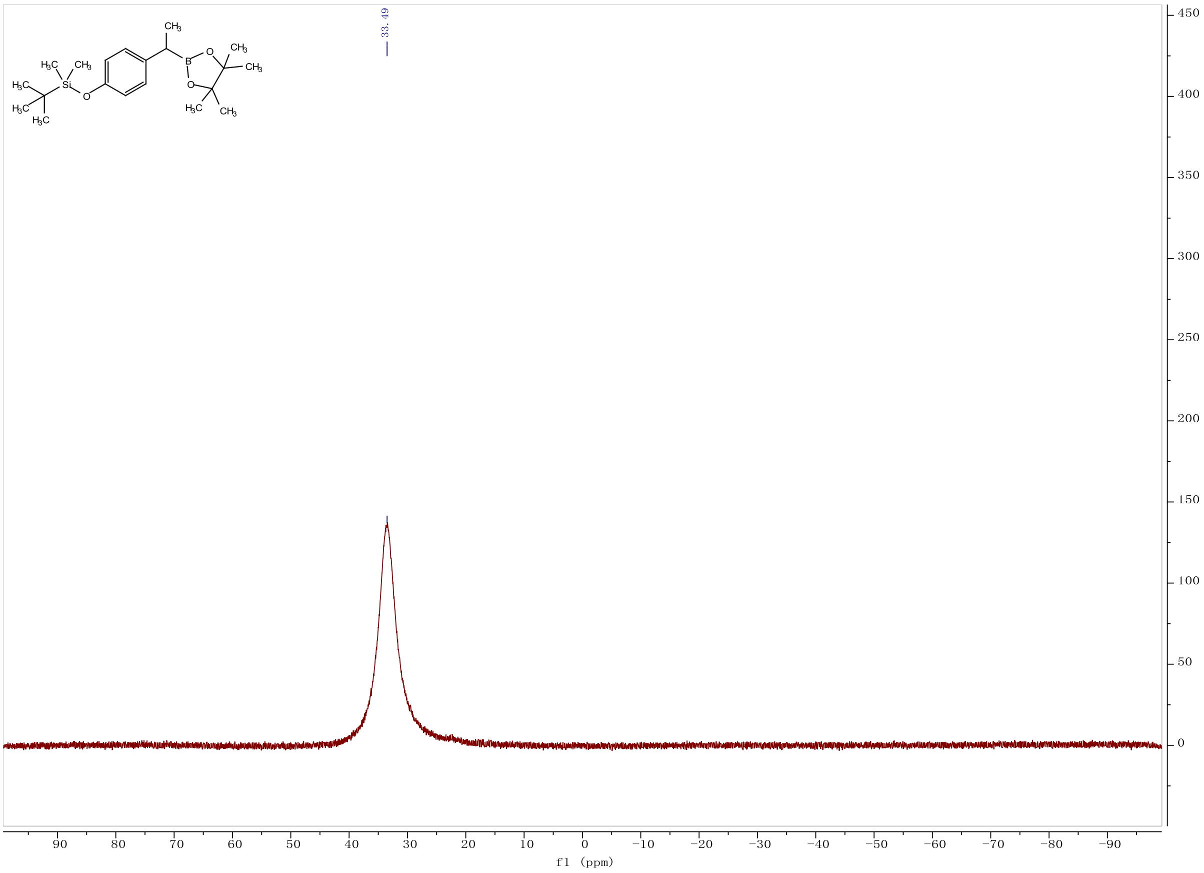

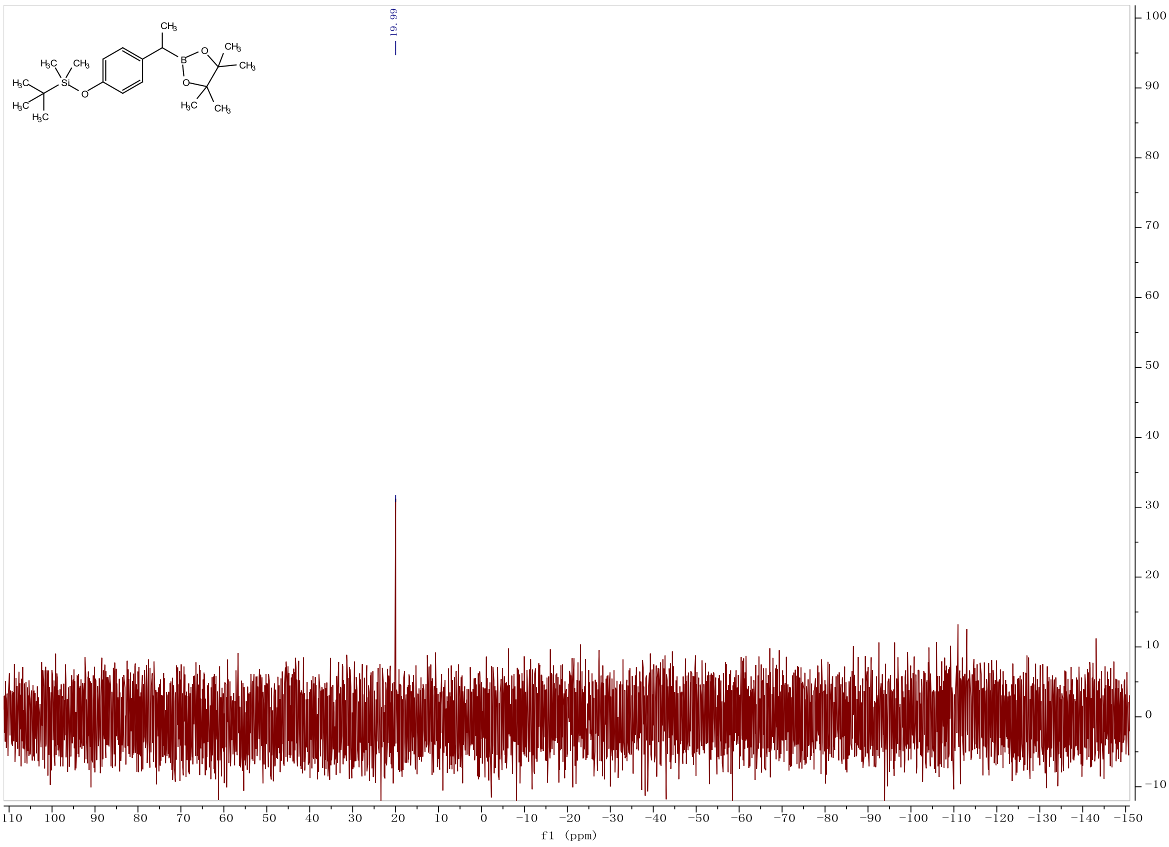

Supplementary Figure 21. **^{1}H**, **^{13}C, ^{11}B, ^{29}Si NMR** of compound **2m**.

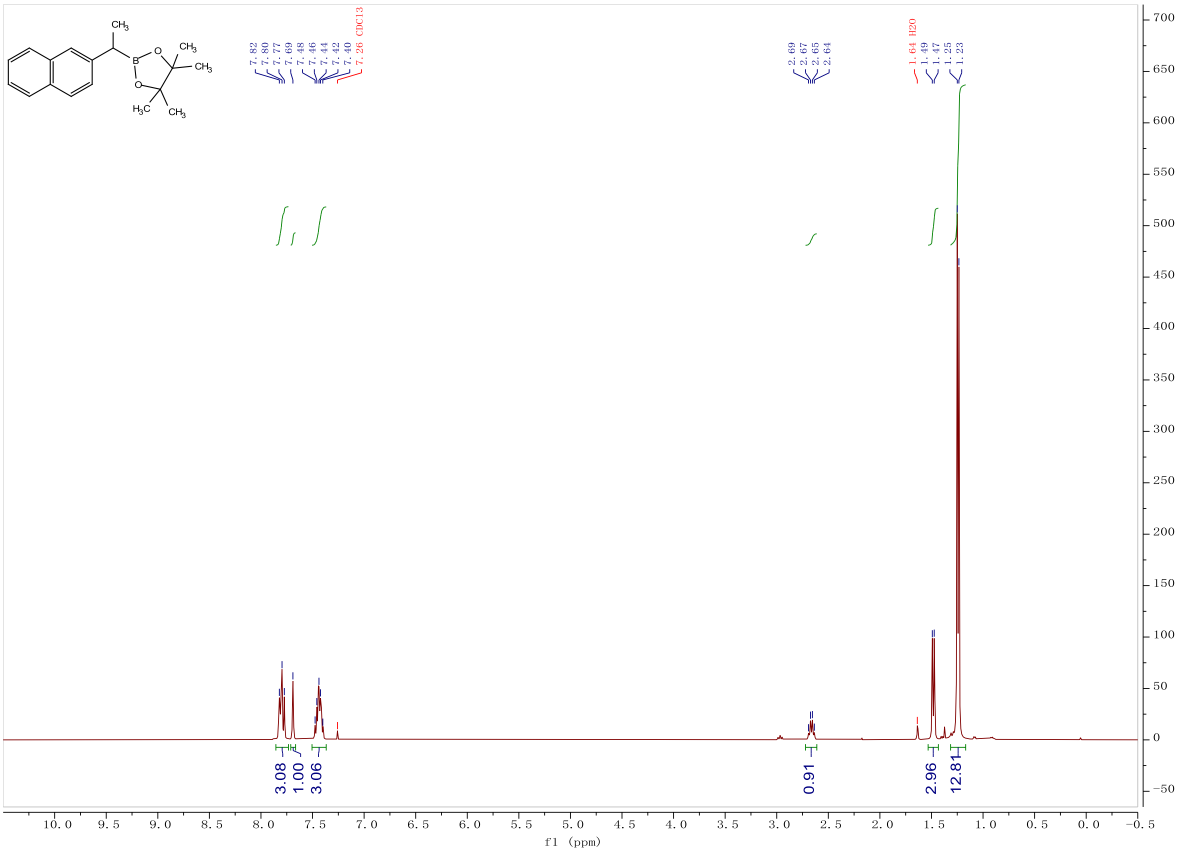
CH3
B
O
O
CH3
CH3
H3C
CH3
7.82
7.80
7.77
7.69
7.48
7.46
7.44
7.42
7.40
7.26 CDCl3
2.69
2.67
2.65
2.64
1.64 H2O
1.49
1.47
1.25
1.23
3.08
1.00
3.06
0.91
2.96
12.81
f1 (ppm)

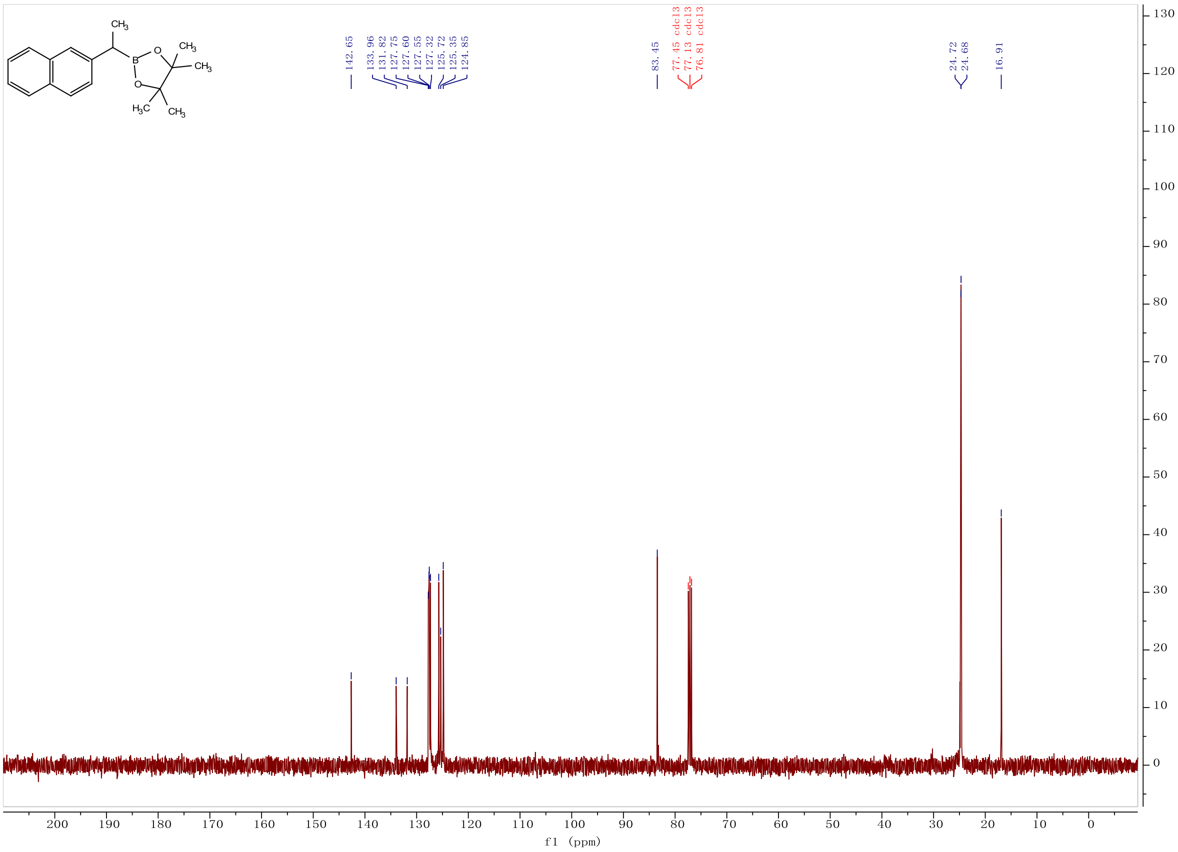
CH3
B
O
O
CH3
CH3
H3C
CH3
142.65
133.96
131.82
127.75
127.60
127.55
127.32
125.72
125.35
124.85
83.45
77.45 cdcl3
77.13 cdcl3
76.81 cdcl3
24.72
24.68
16.91
f1 (ppm)

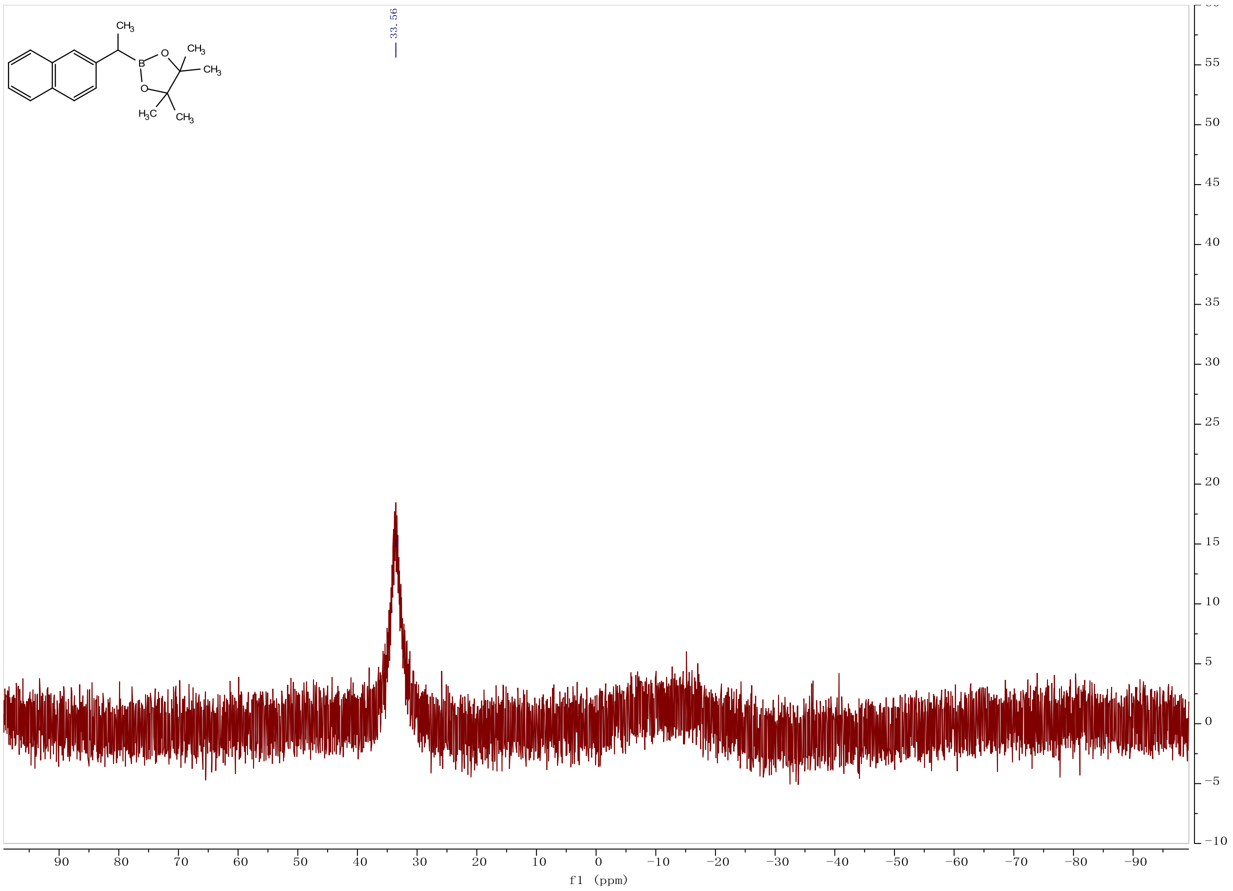

Supplementary Figure 22. ^{1}**H**, ^{13}**C,** ^{11}**B NMR** of compound **2n**.

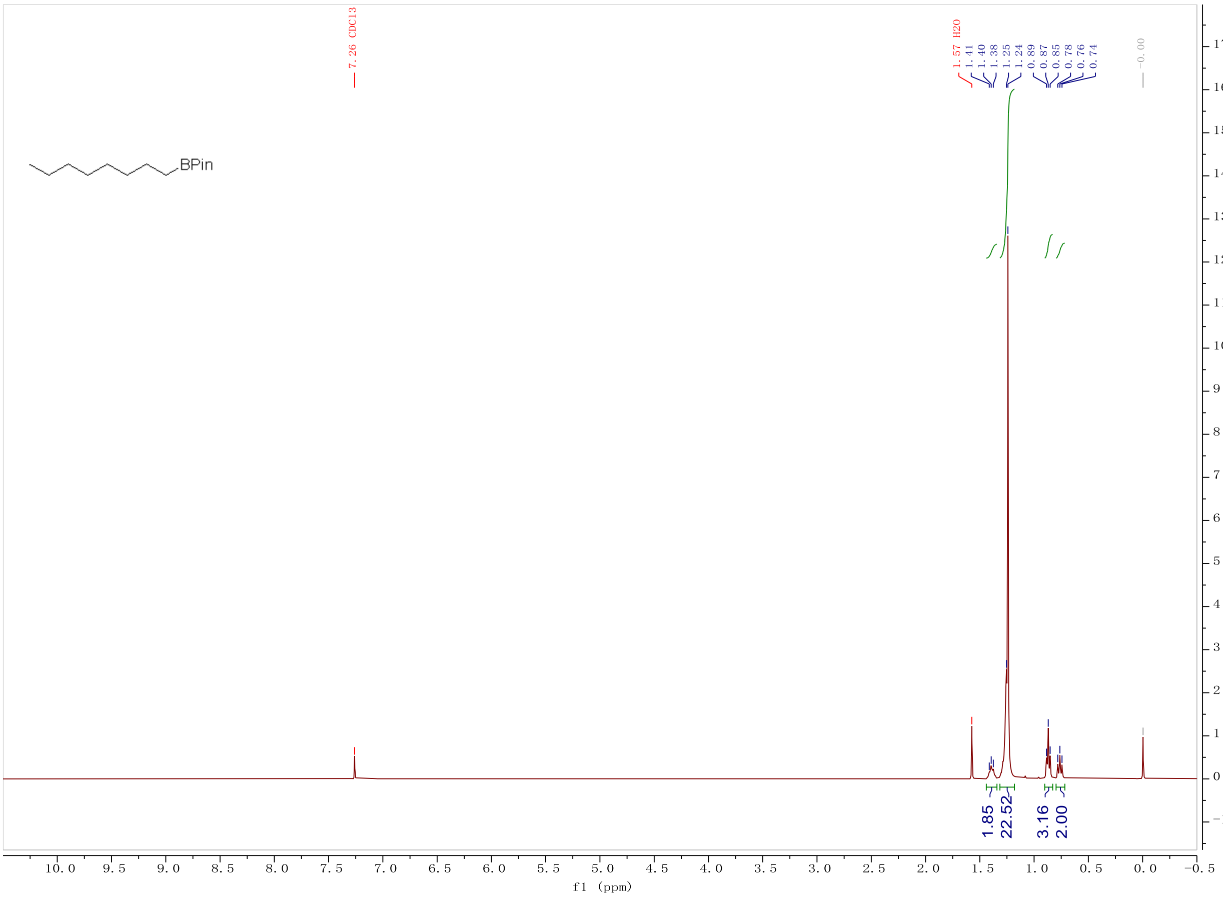

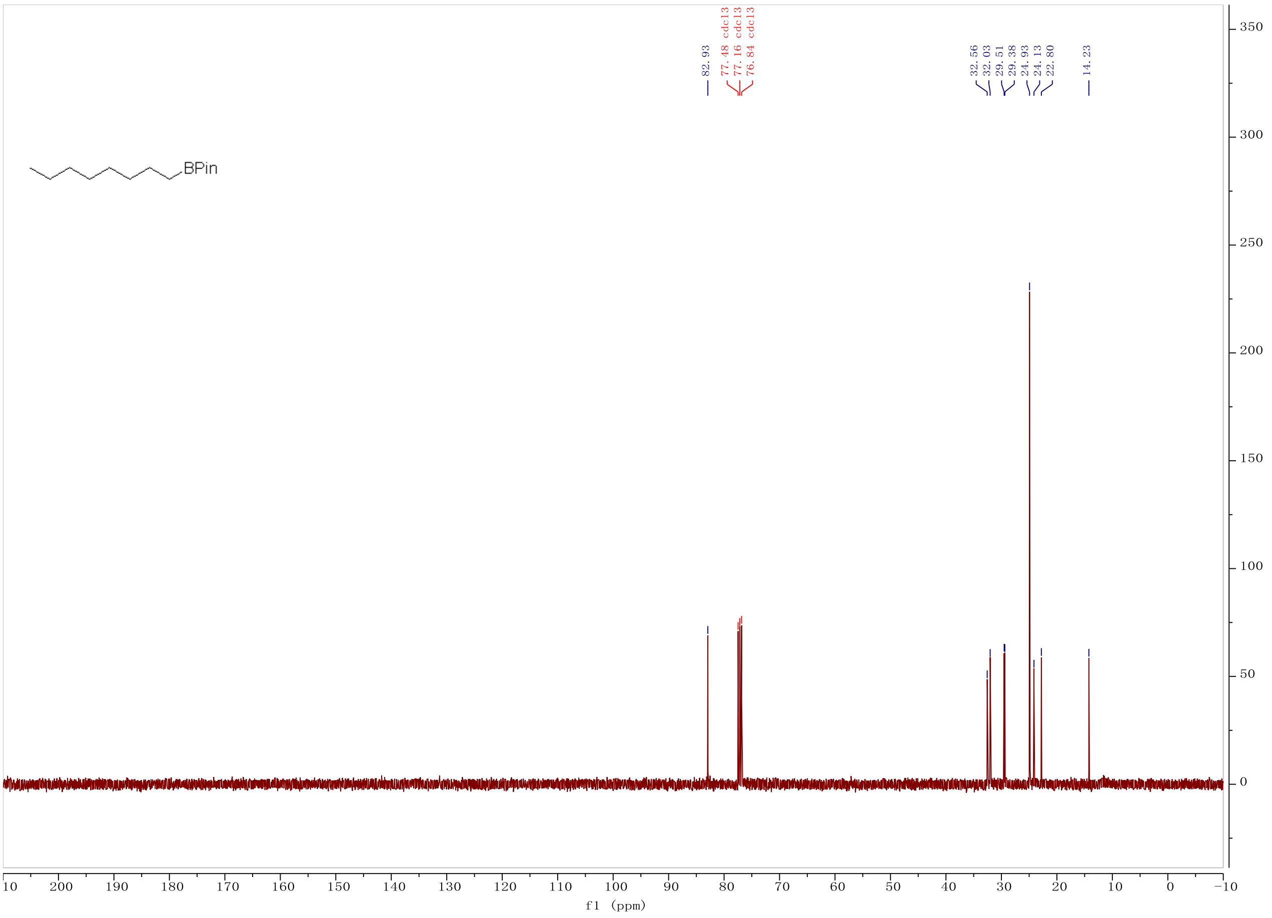

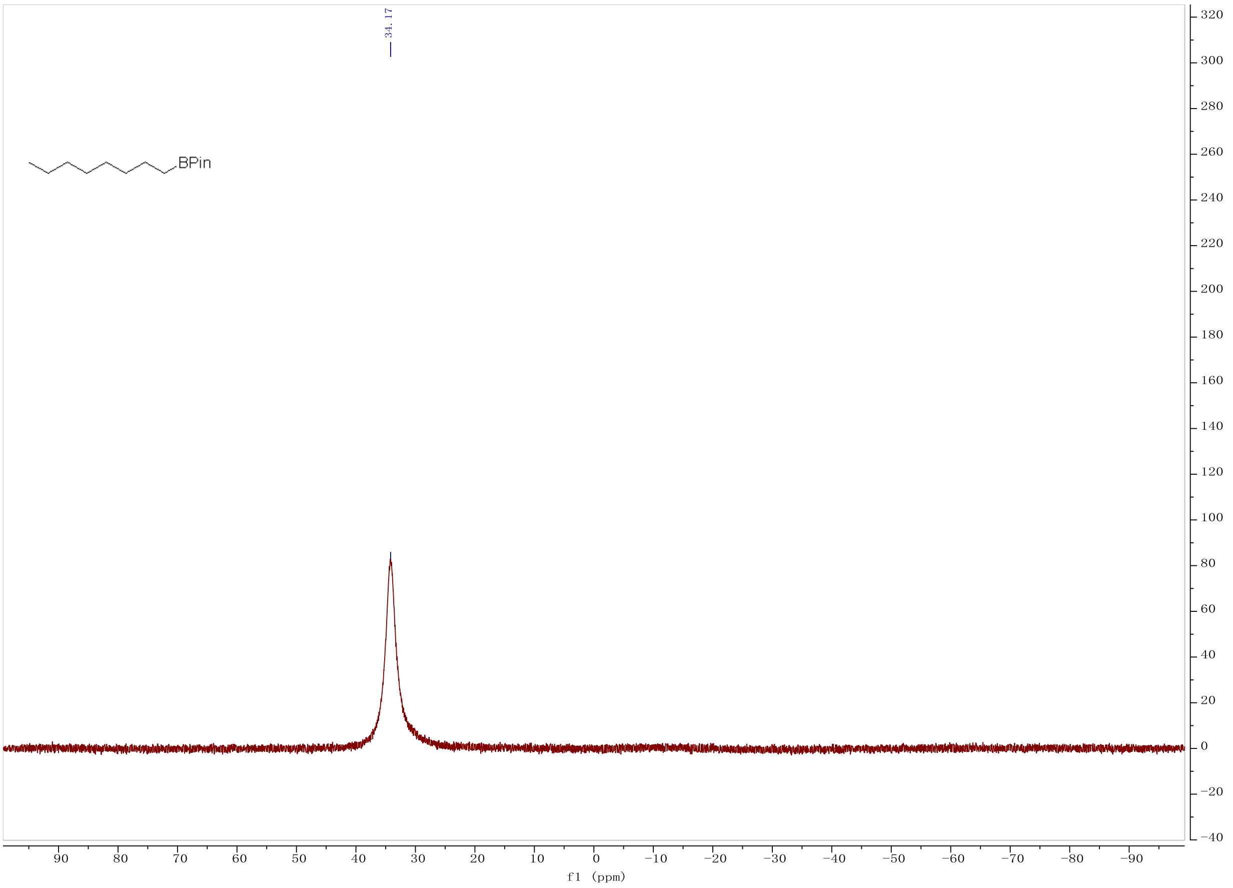

Supplementary Figure 23. ^{1}**H,** ^{13}**C,** ^{11}**B NMR** of compound **3o**

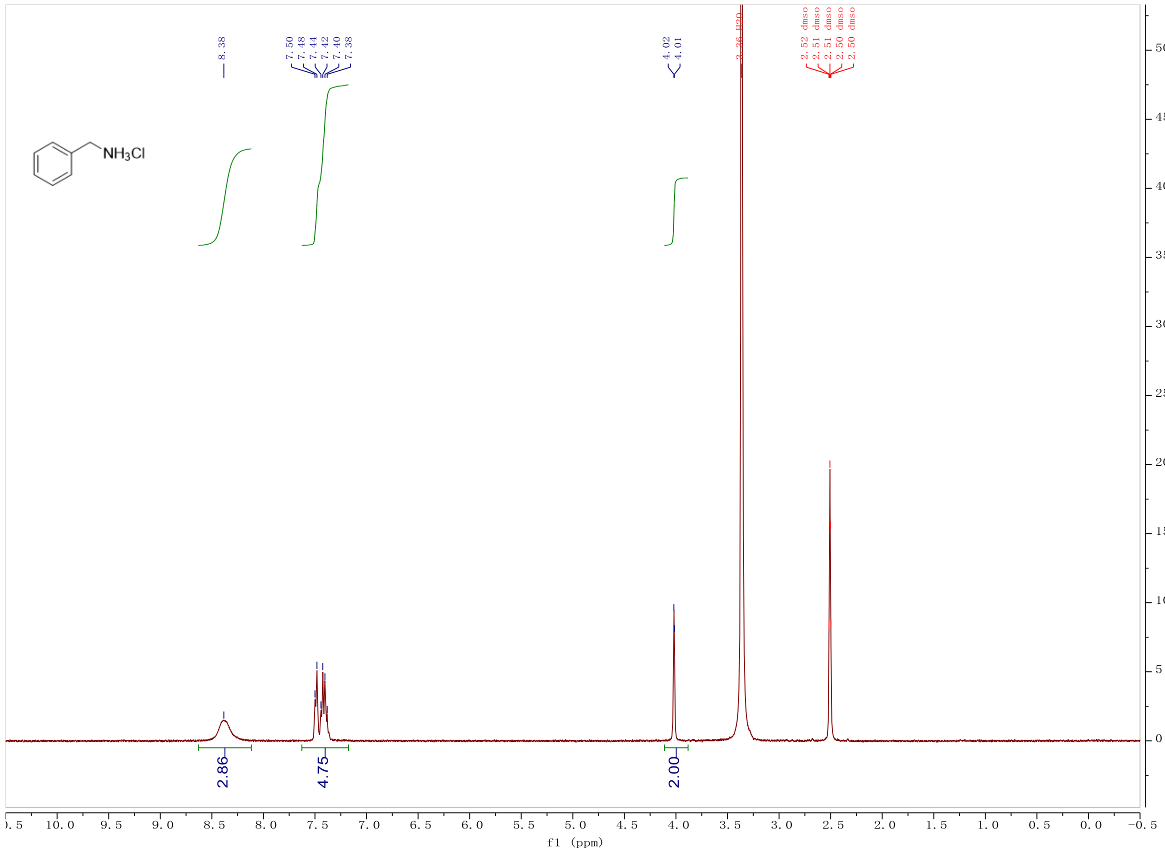

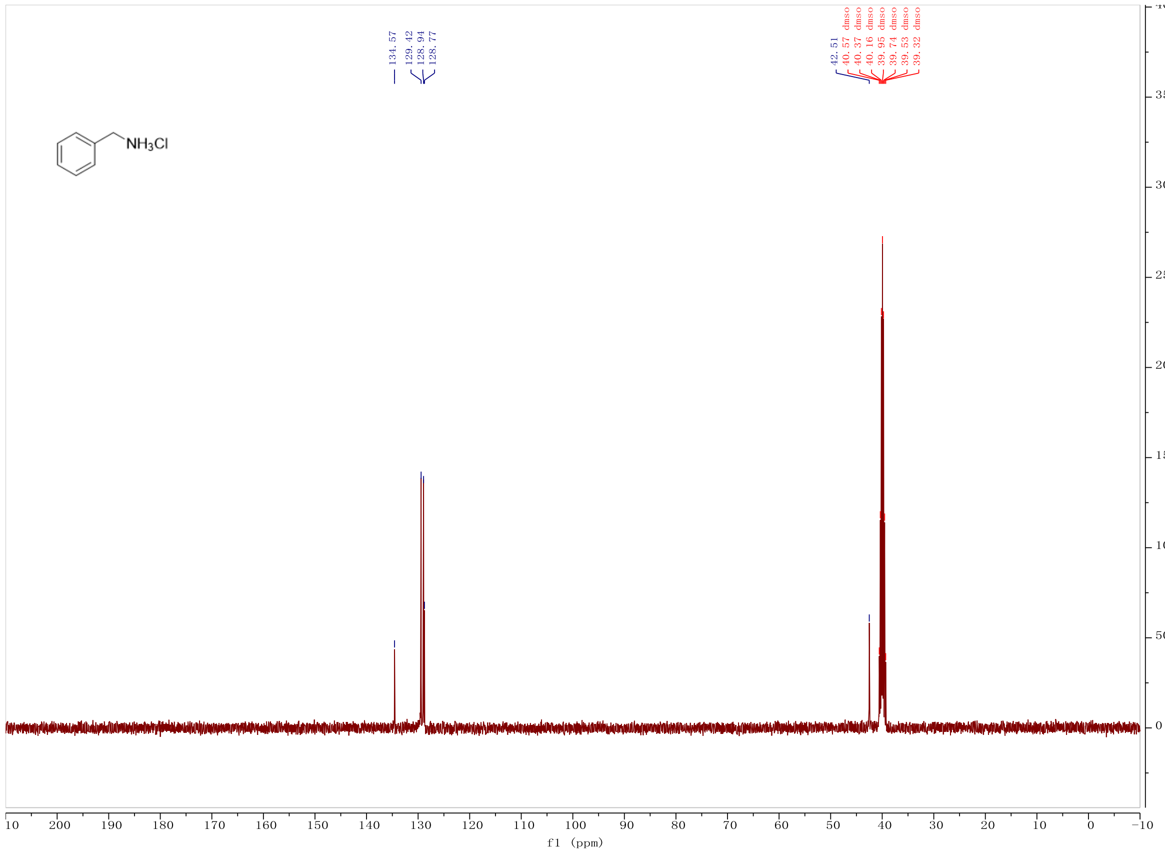

Supplementary Figure 24. ^{1}**H NMR** (400 MHz, DMSO-d_6) and ^{13}**C NMR** (101 MHz, DMSO-d_6) of compound **7a**.

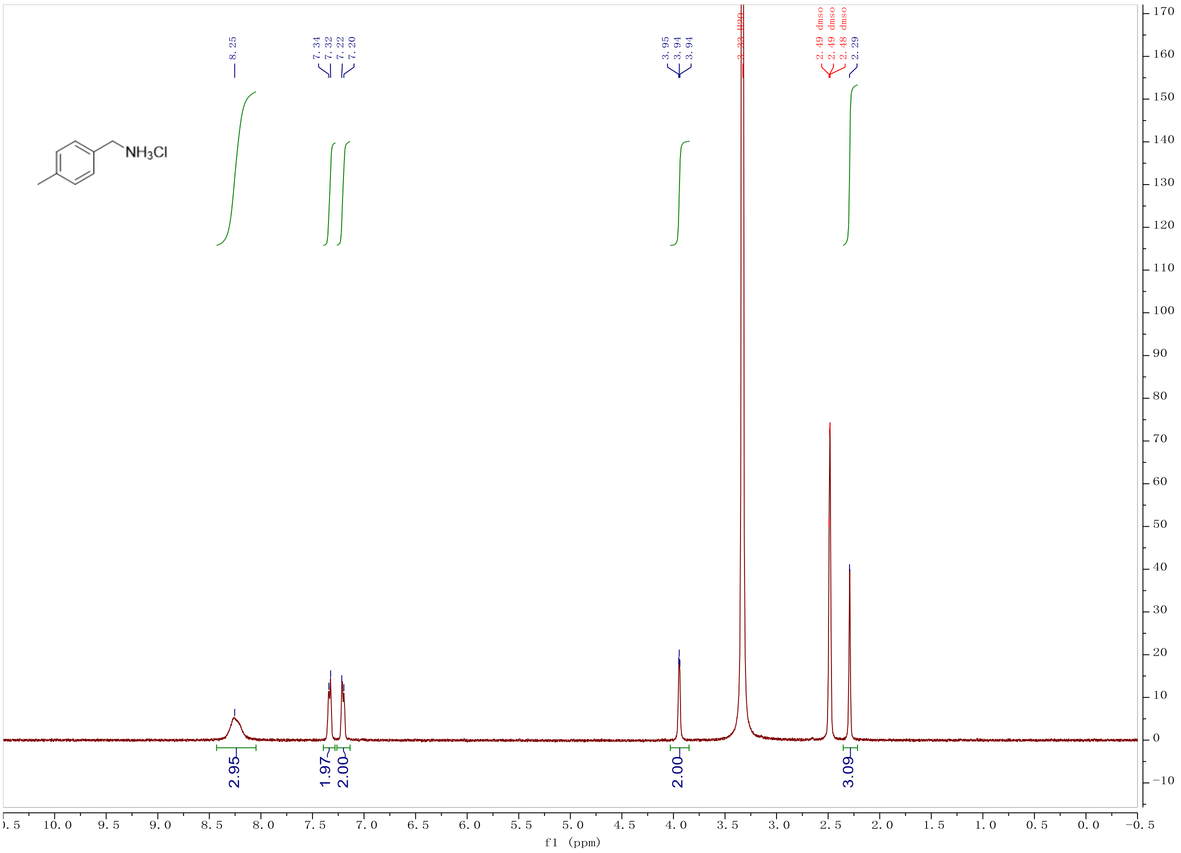

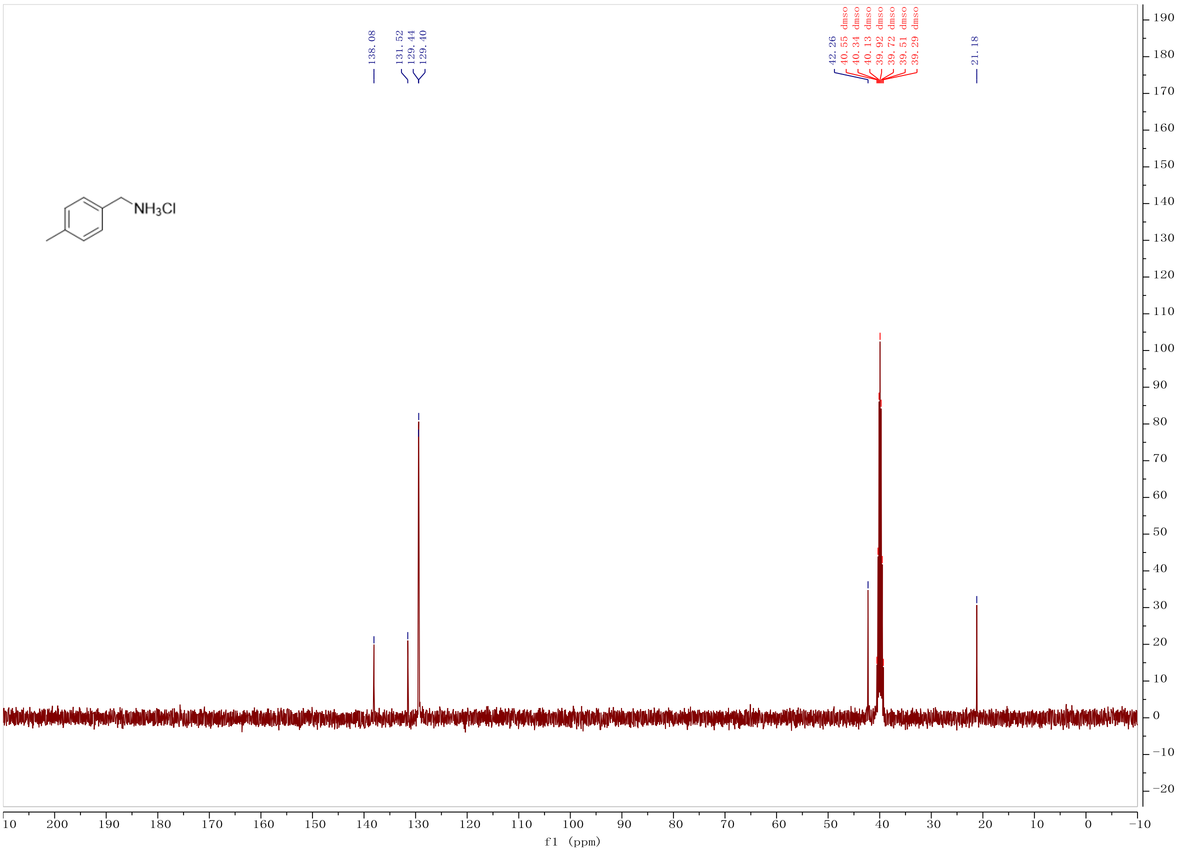

Supplementary Figure 25. ^{1}**H NMR** (400 MHz, DMSO-d_6) and ^{13}**C NMR** (101 MHz, DMSO-d_6) of compound **7b**.

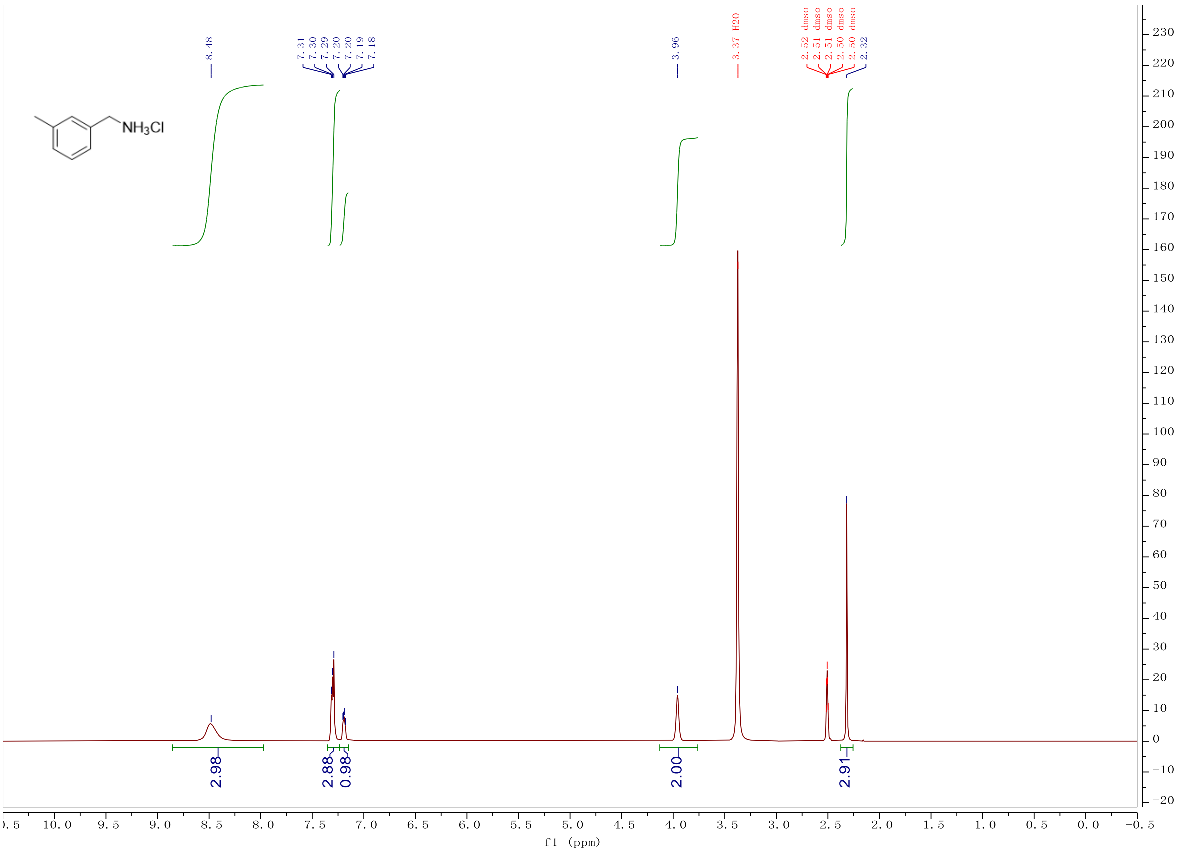

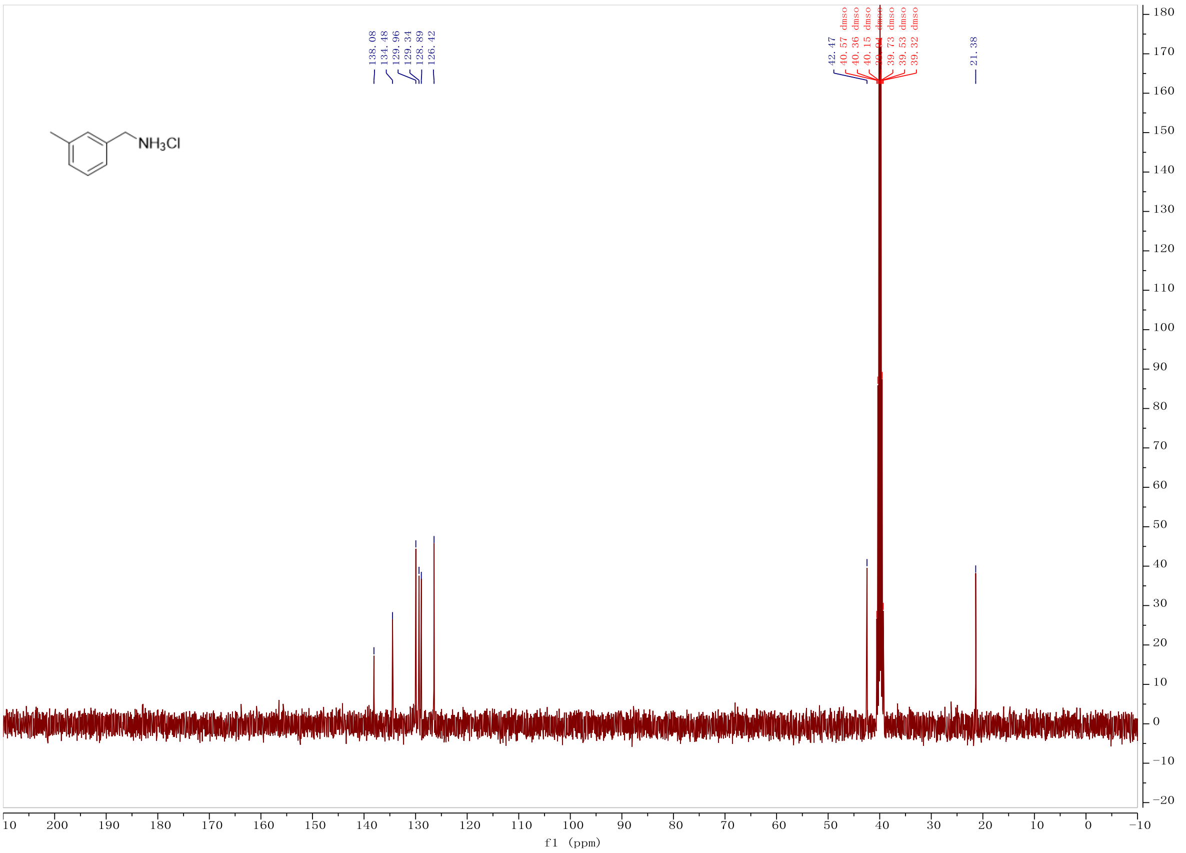

Supplementary Figure 26. ^{1}**H NMR** (400 MHz, DMSO-d_6) and ^{13}**C NMR** (101 MHz, DMSO-d_6) of compound **7c**.

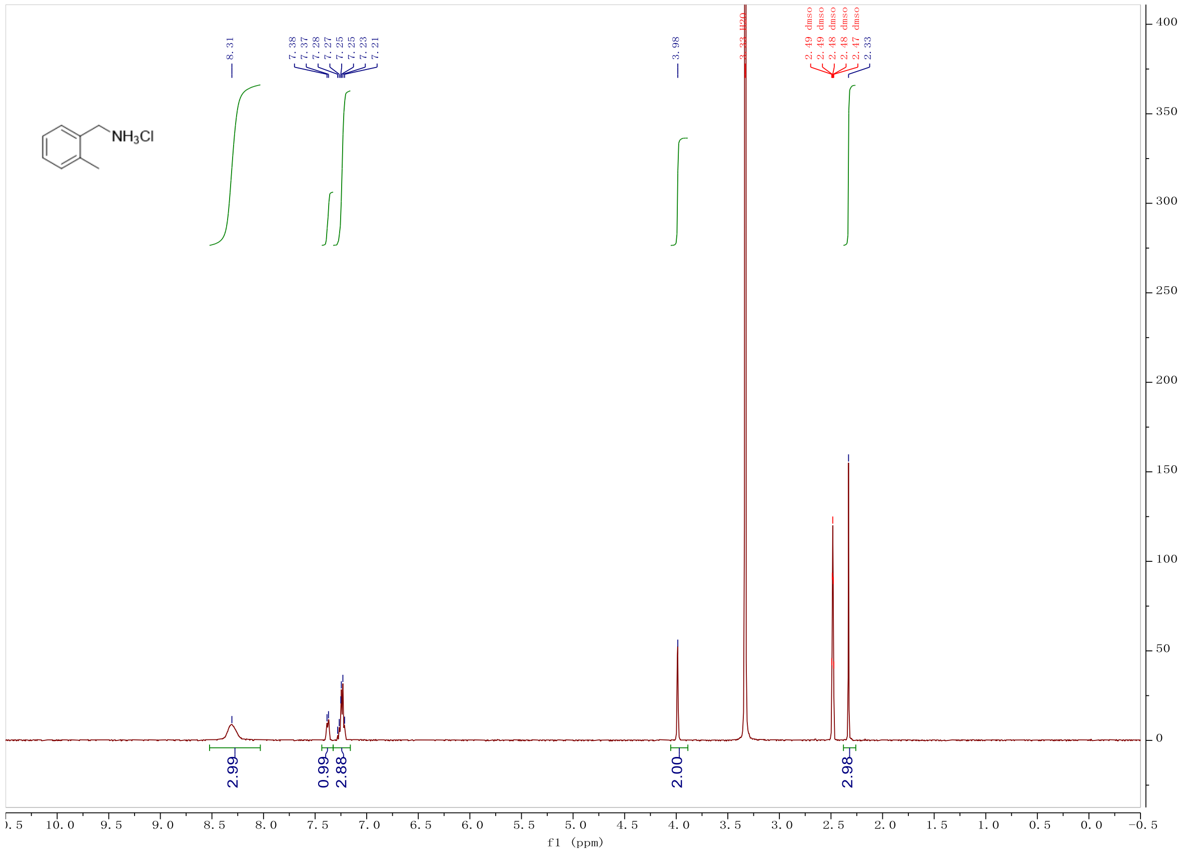

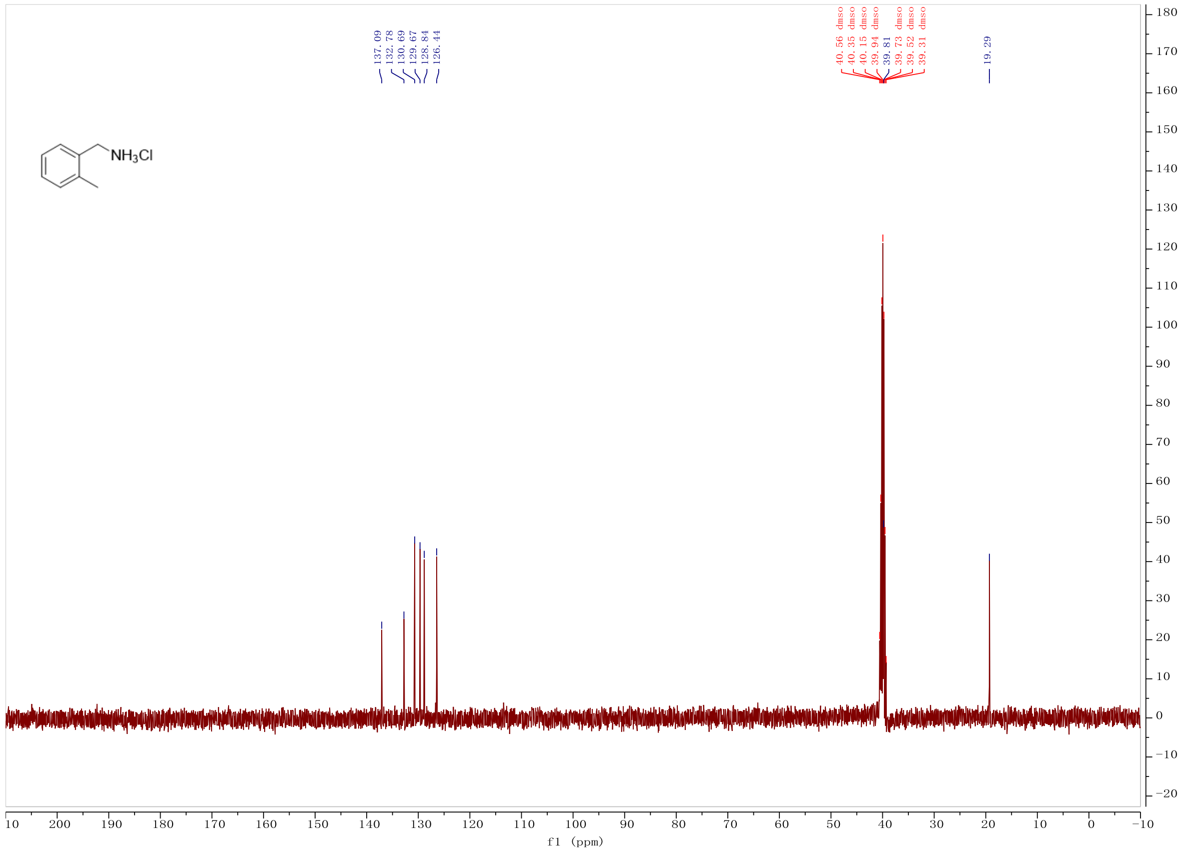

Supplementary Figure 27. **^{1}H NMR** (400 MHz, DMSO-d_6) and **^{13}C NMR** (101 MHz, DMSO-d_6) of compound **7d**.

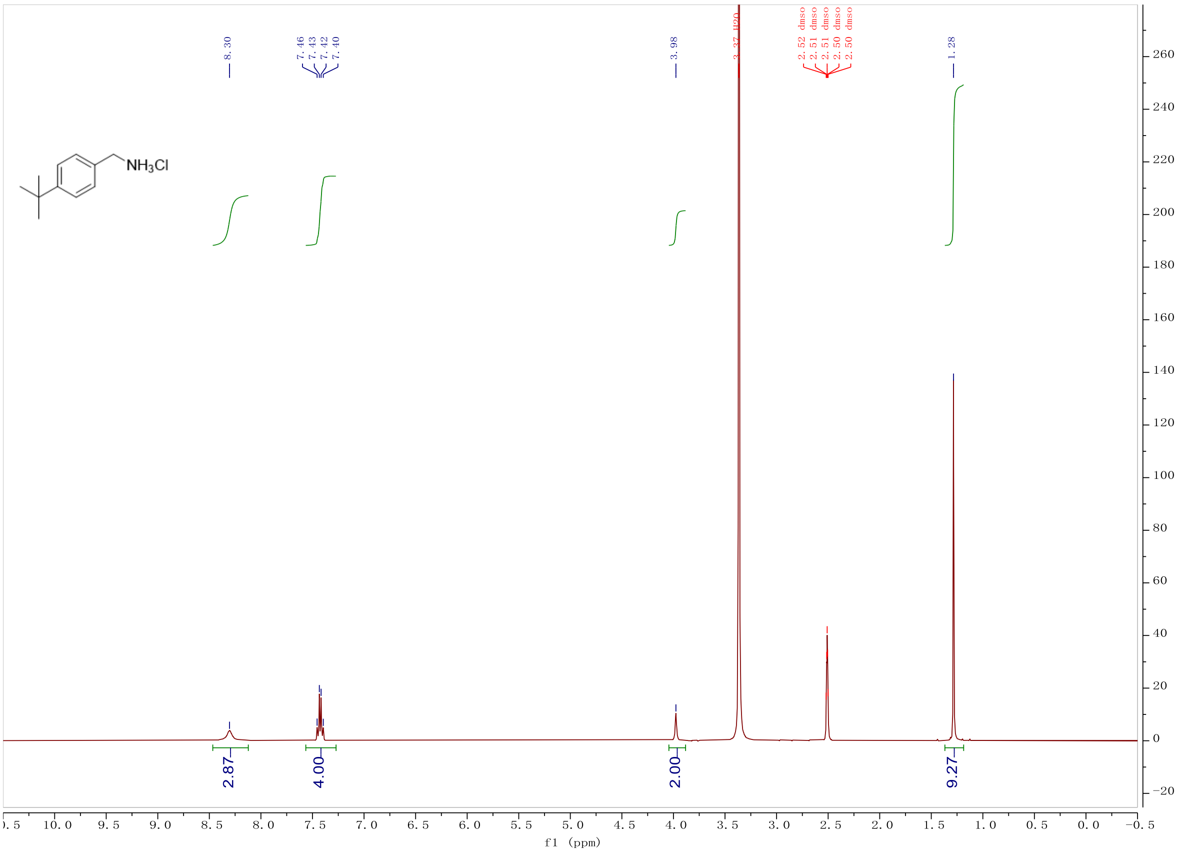

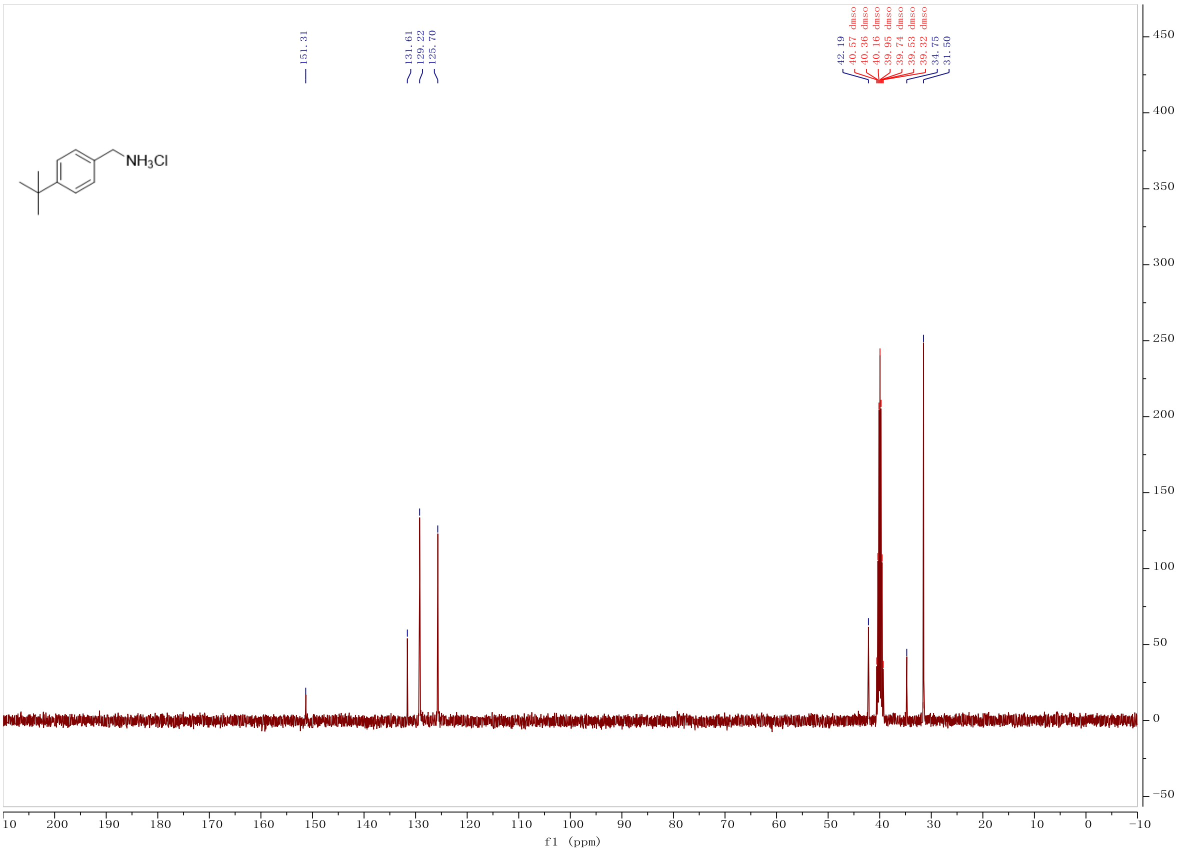

Supplementary Figure 28. ^{1}**H NMR** (400 MHz, DMSO-*d*$_6$) and ^{13}**C NMR** (101 MHz, DMSO-*d*$_6$) of compound **7e.**

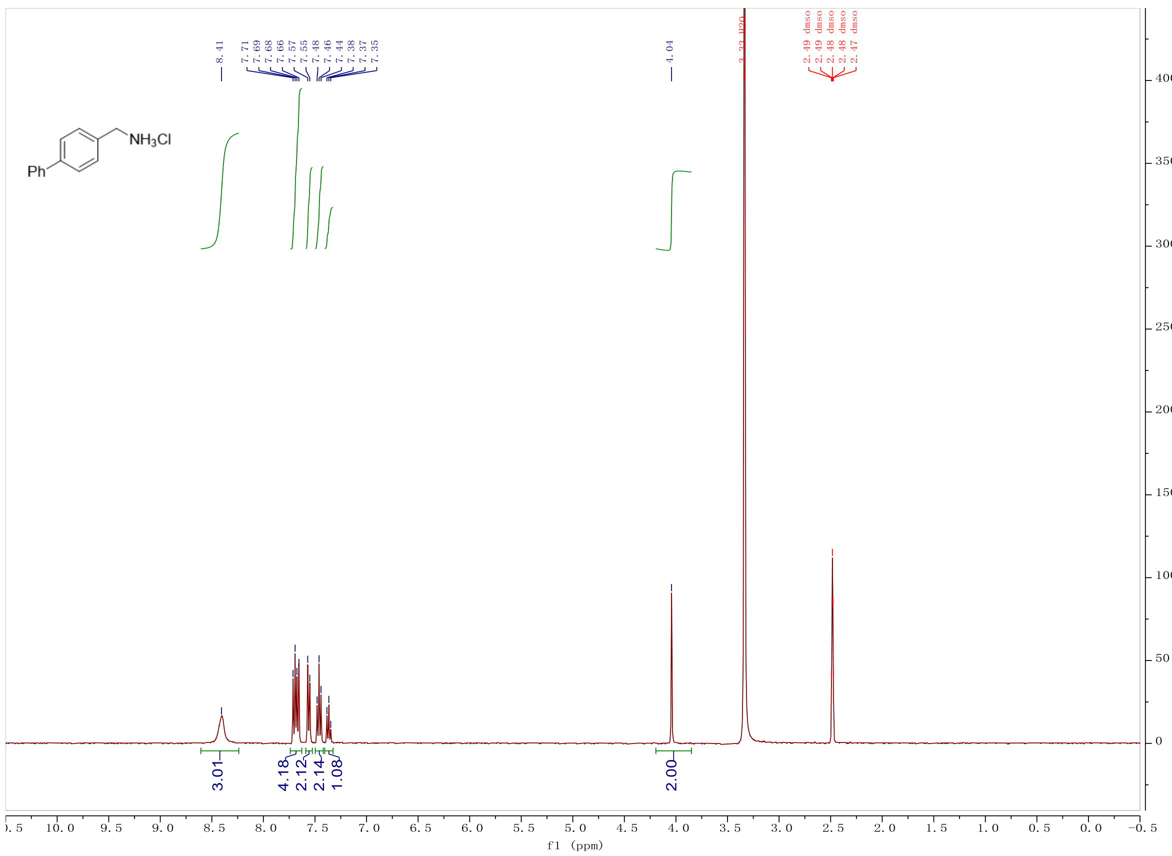

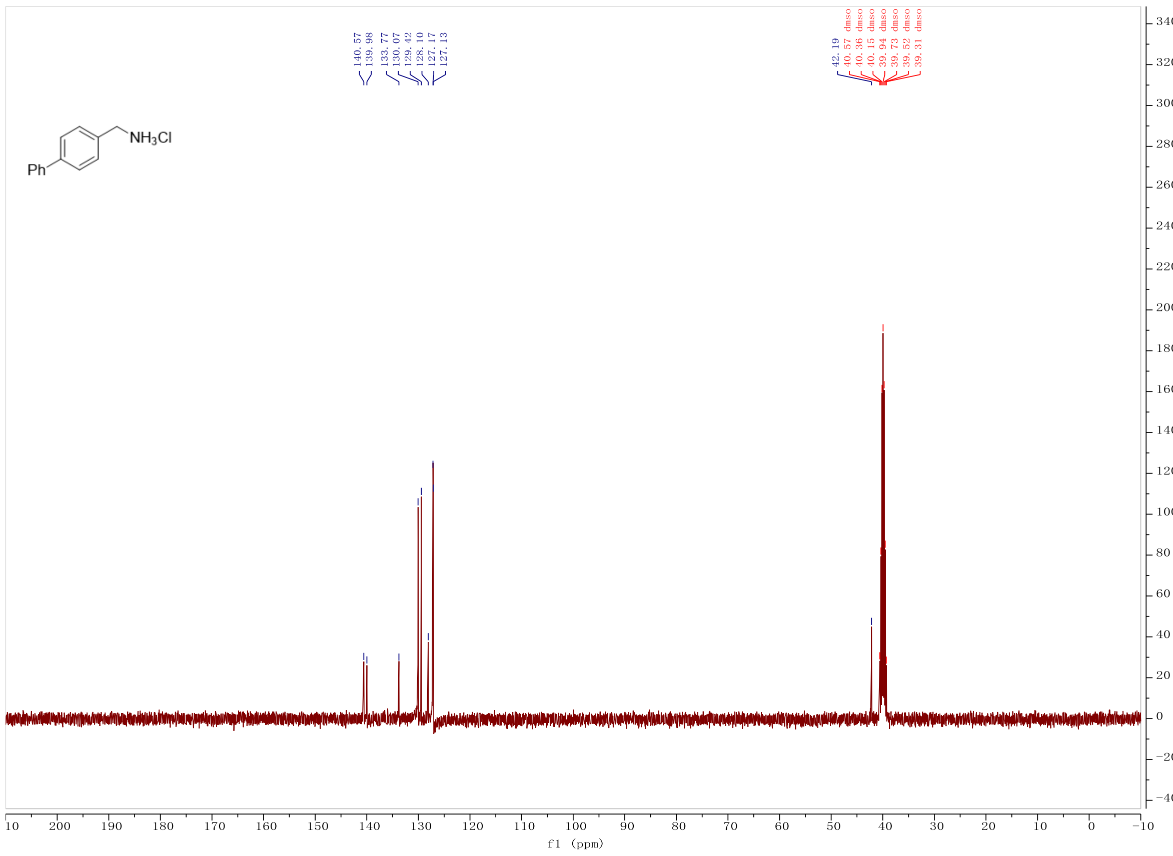

Supplementary Figure 29. ^{1}**H NMR** (400 MHz, DMSO-d_6) and ^{13}**C NMR** (101 MHz, DMSO-d_6) of compound **7f**.

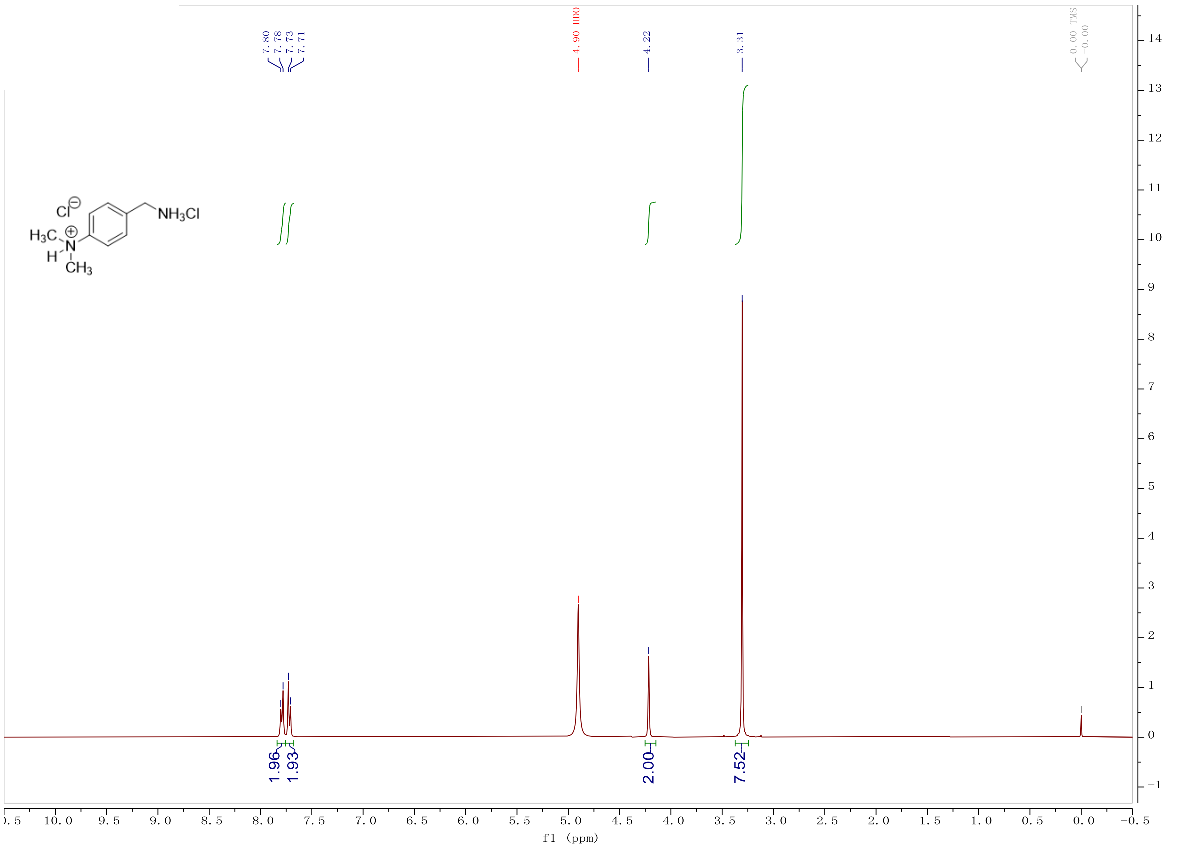

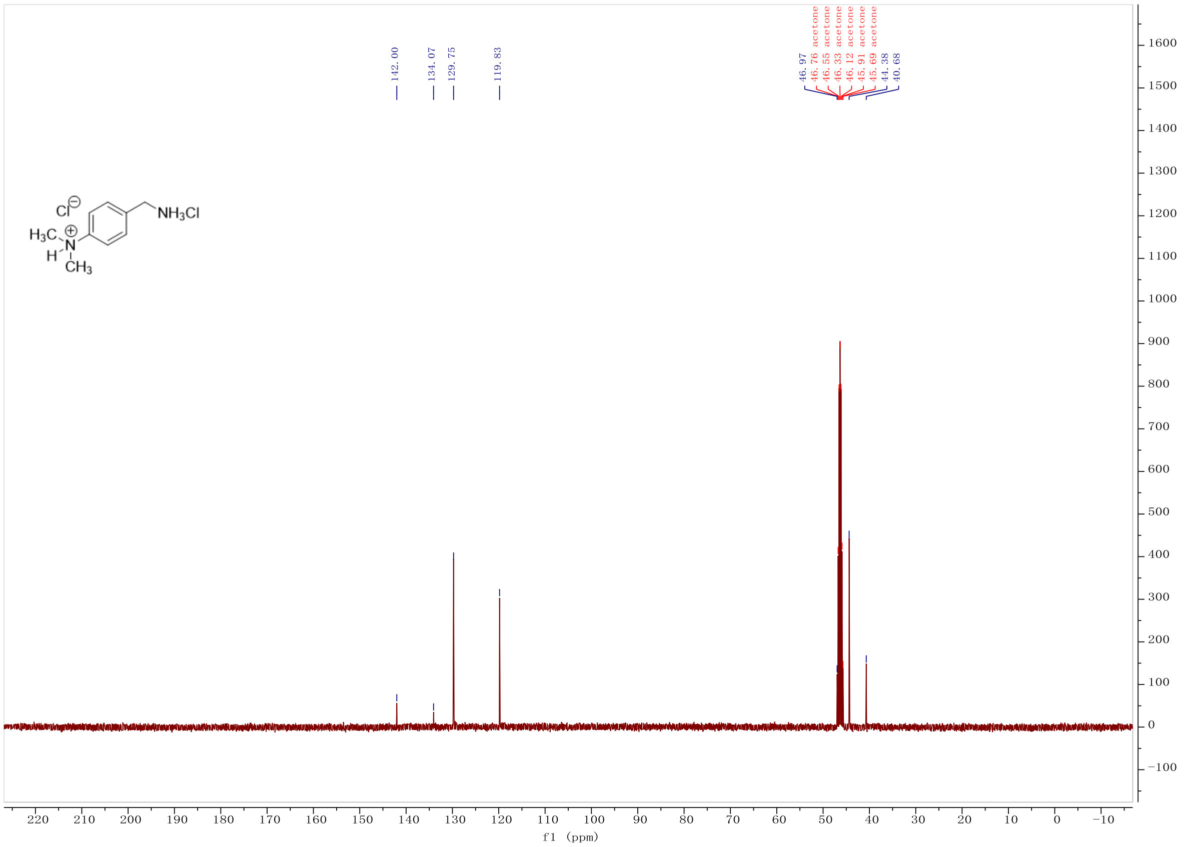

Supplementary Figure 30. ^{1}**H NMR** (400 MHz, CD_3OD) and ^{13}**C NMR** (101 MHz, CD_3OD) of compound **7g**.

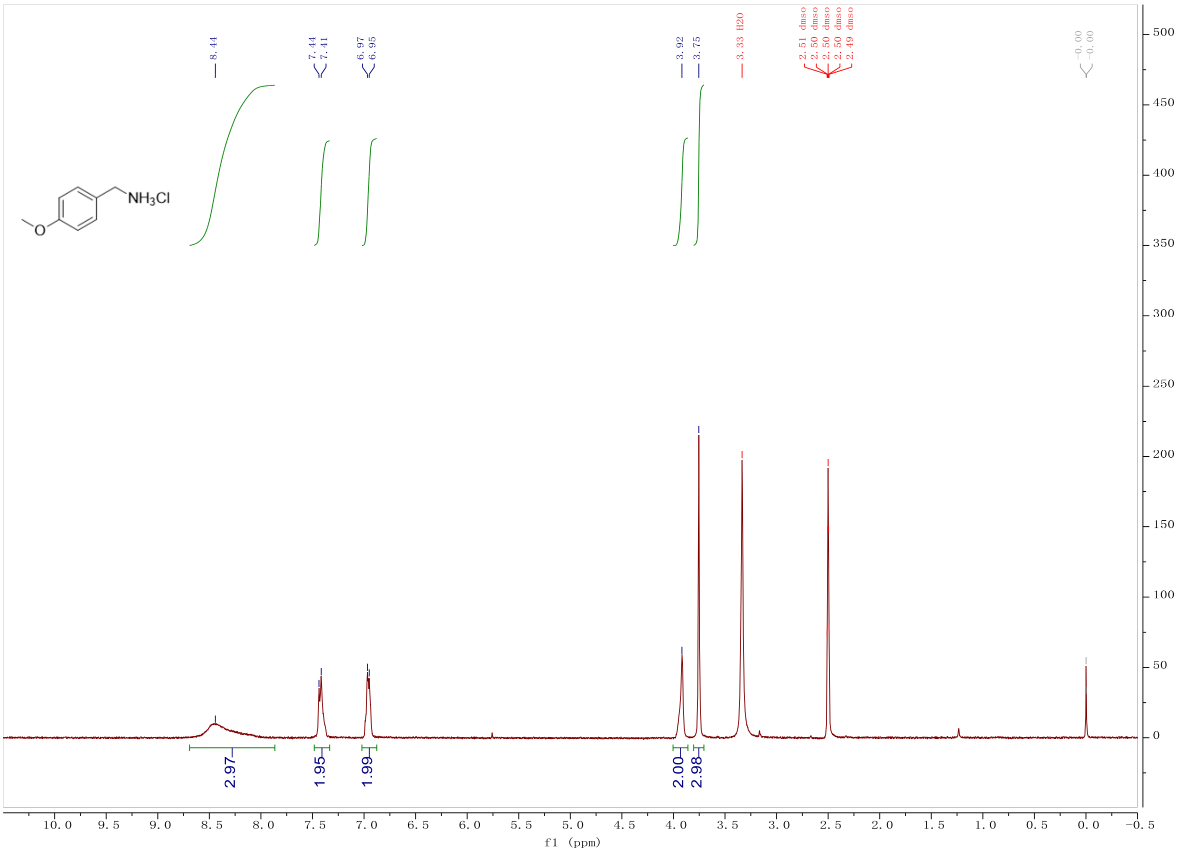

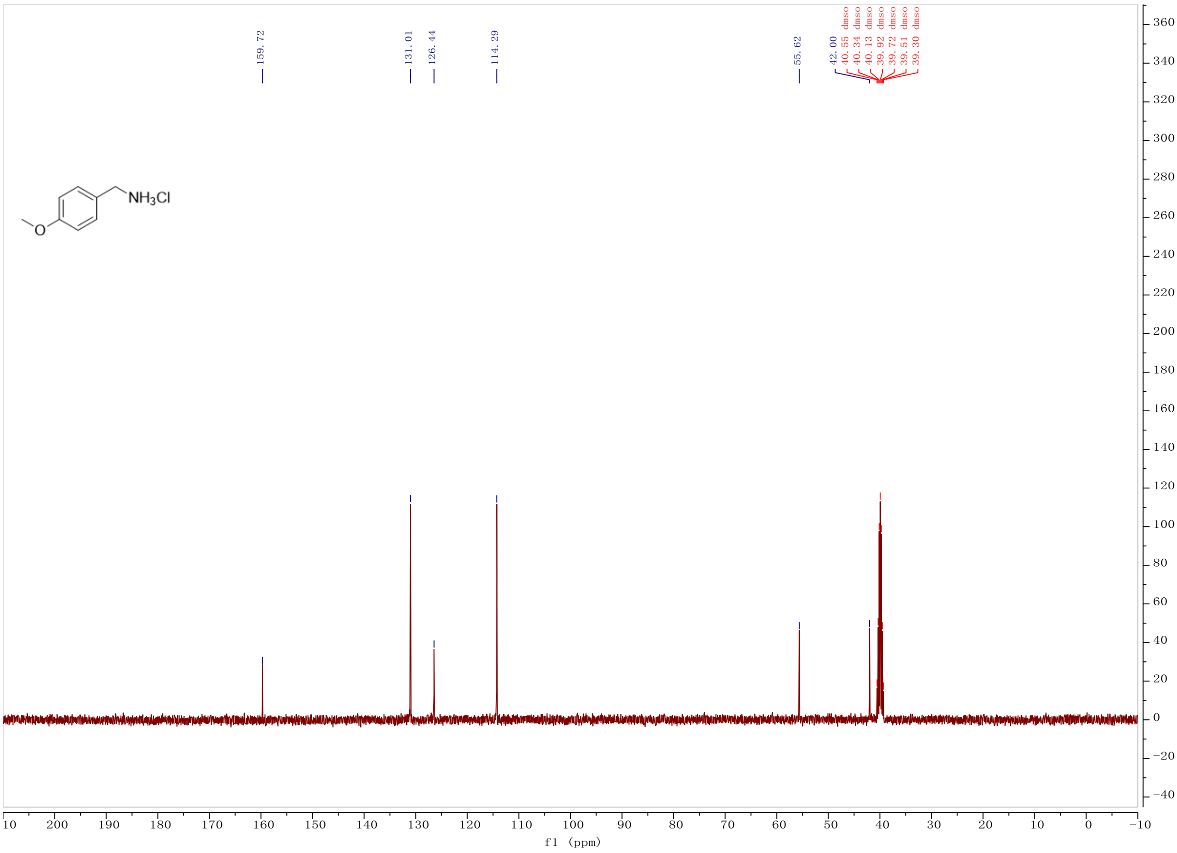

Supplementary Figure 31. ^{1}**H NMR** (400 MHz, DMSO-d_6) and ^{13}**C NMR** (101 MHz, DMSO-d_6) of compound **7h**.

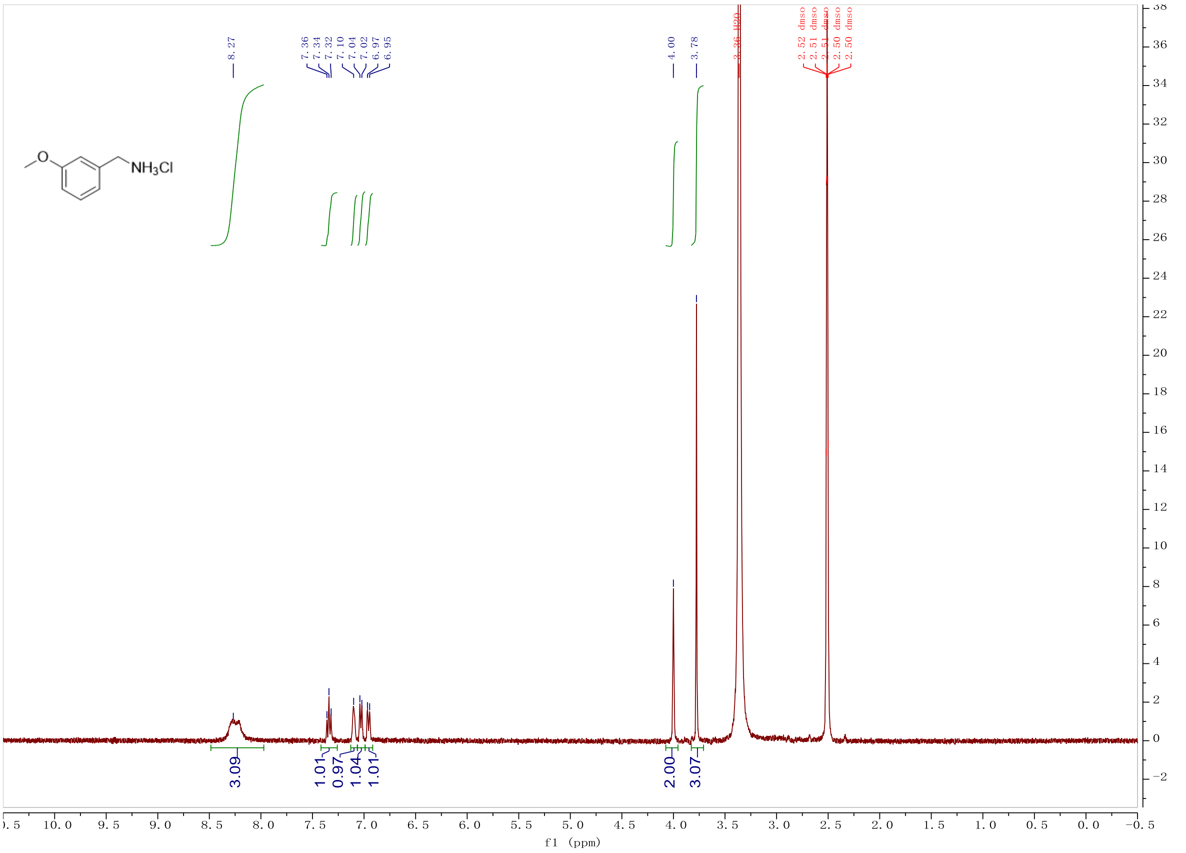

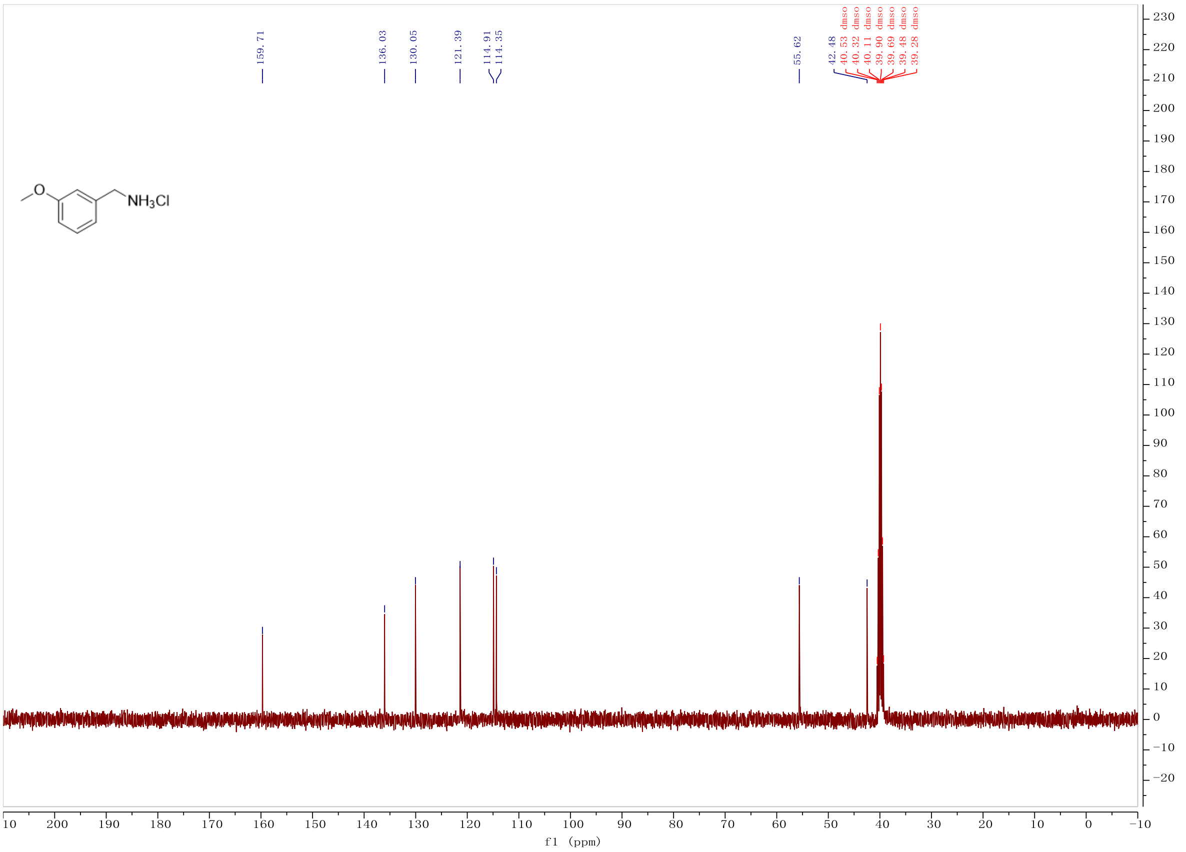

Supplementary Figure 32. **^{1}H NMR** (400 MHz, DMSO-d_6) and **^{13}C NMR** (101 MHz, DMSO-d_6) of compound **7i**.

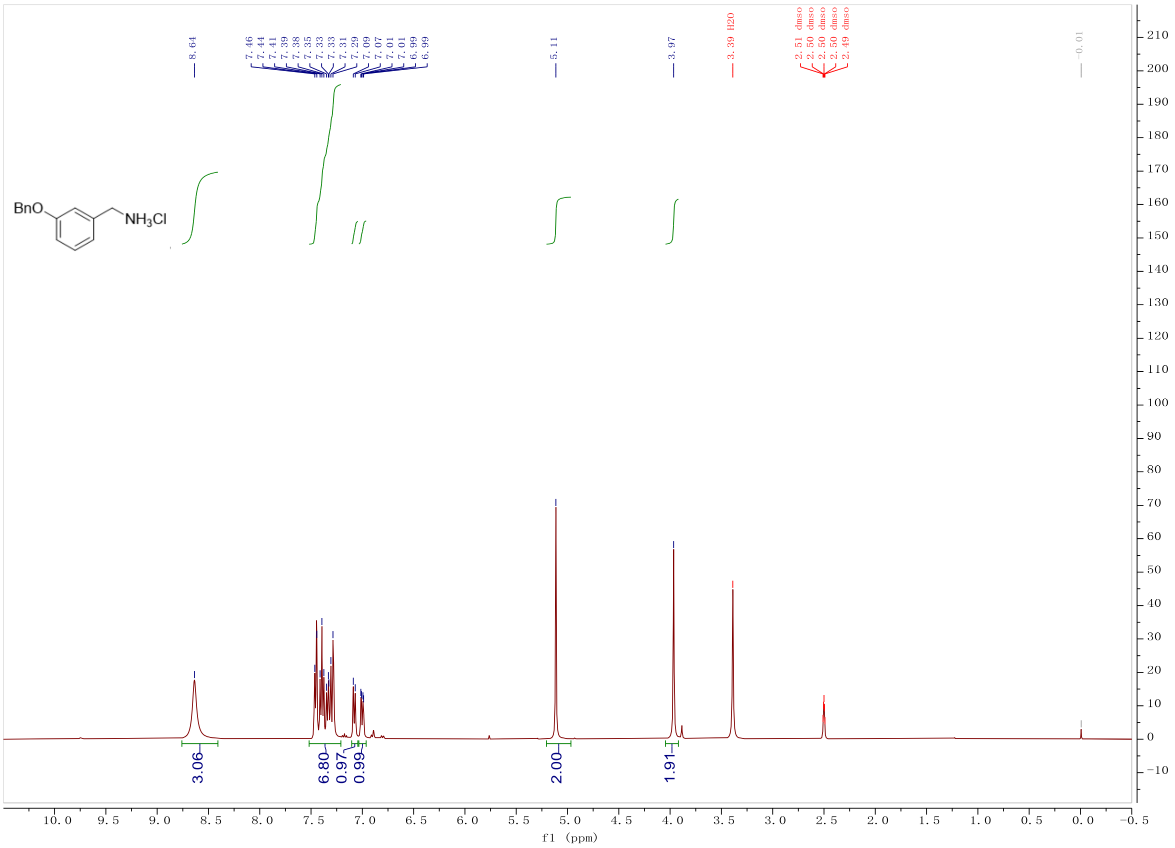

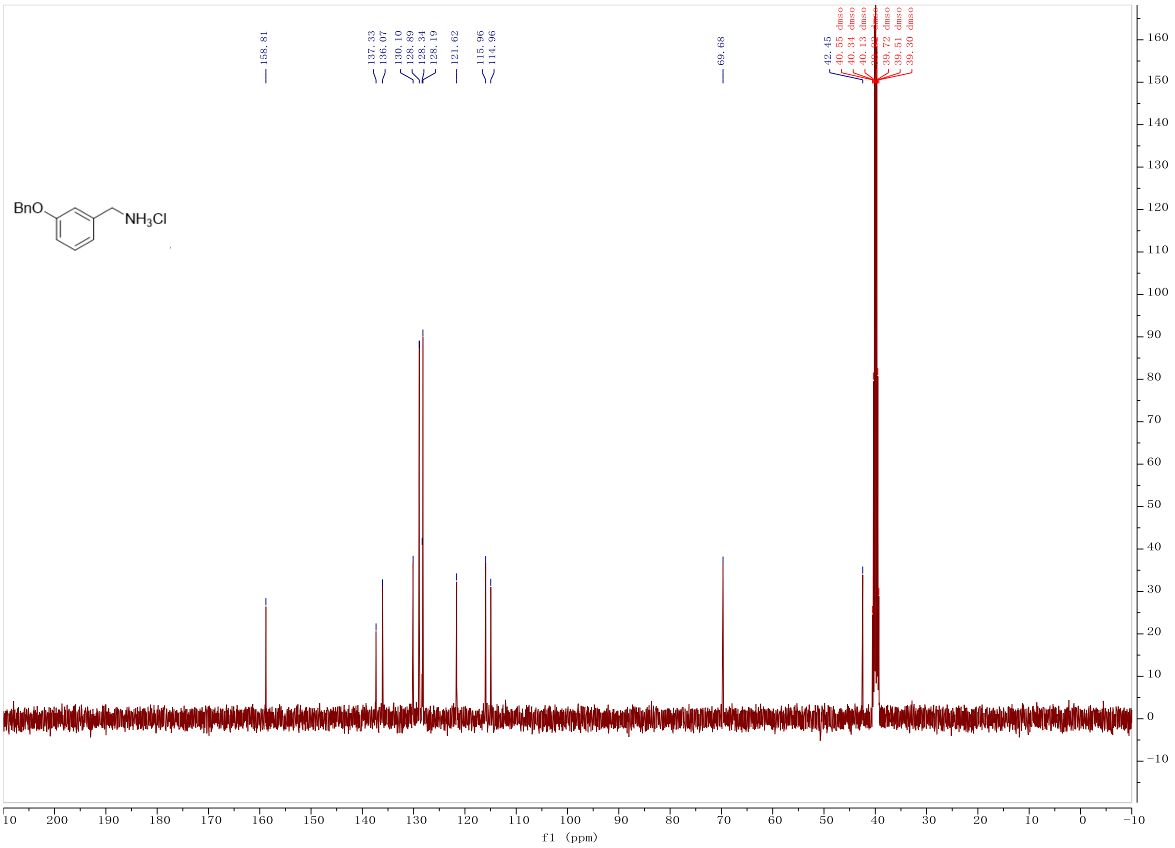

Supplementary Figure 33. **^{1}H NMR** (400 MHz, DMSO-d_6) and **^{13}C NMR** (101 MHz, DMSO-d_6) of compound **7j**.

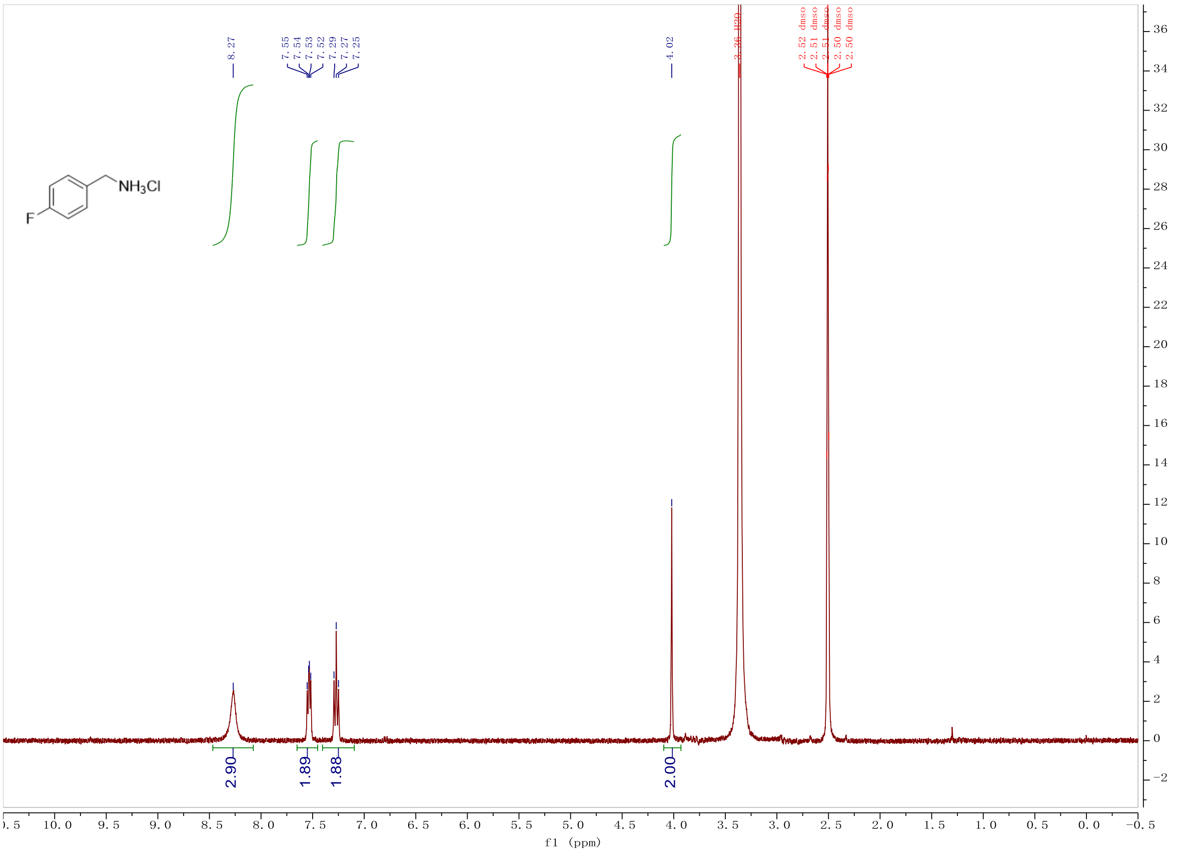

NH3Cl
F
8.27
7.55
7.54
7.53
7.52
7.29
7.27
7.25
4.02
2.52 dmso
2.51 dmso
2.51 dmso
2.50 dmso
2.50 dmso
2.90
1.89
1.88
2.00
f1 (ppm)

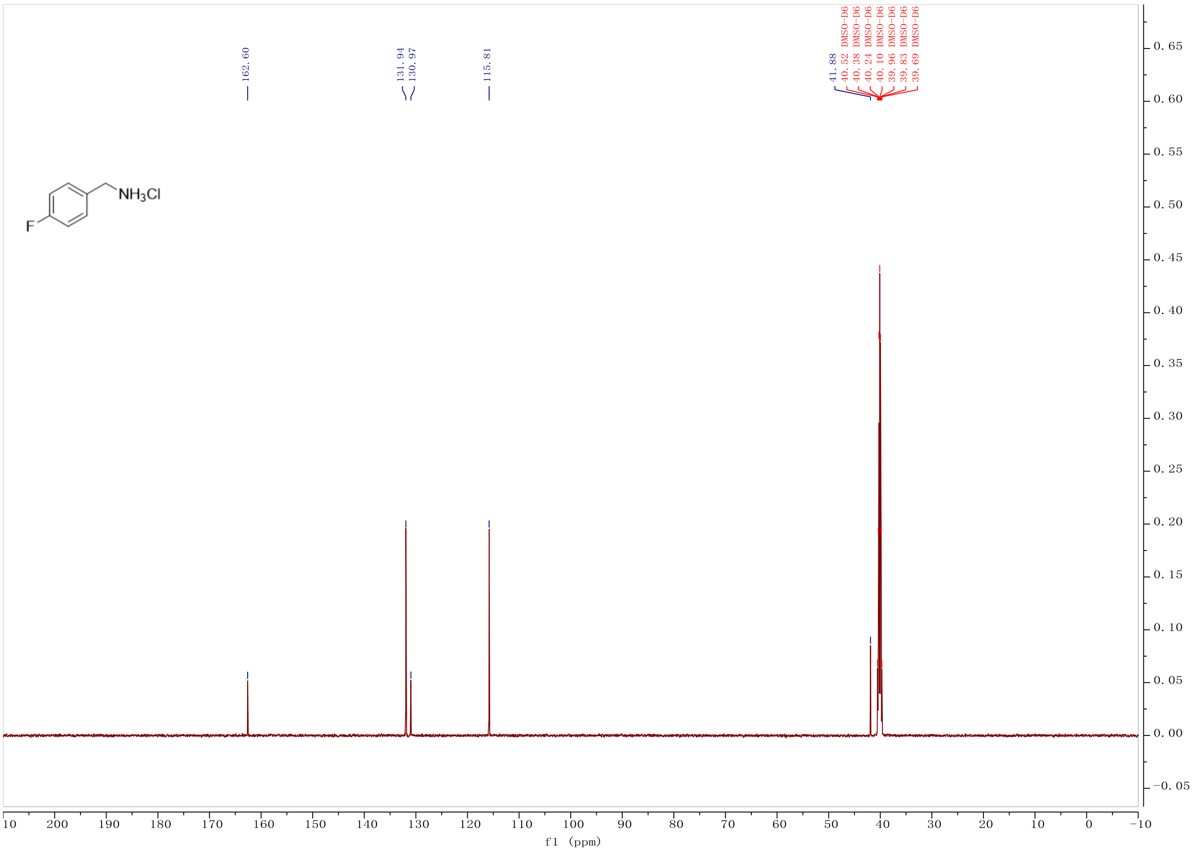

NH3Cl
F
162.60
131.94
130.97
115.81
41.88
40.52 DMSO-D6
40.38 DMSO-D6
40.24 DMSO-D6
40.10 DMSO-D6
39.96 DMSO-D6
39.83 DMSO-D6
39.69 DMSO-D6
f1 (ppm)

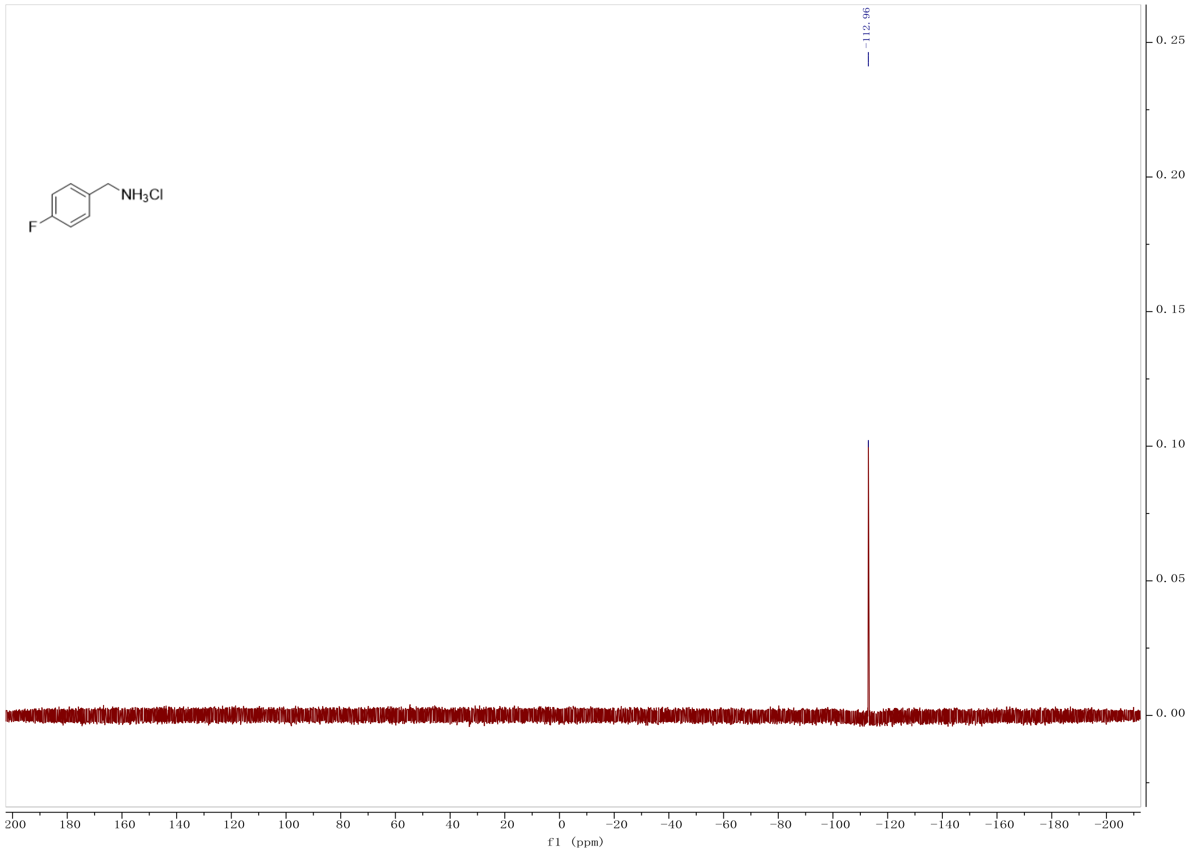

Supplementary Figure 34. ^{1}**H NMR** (400 MHz, DMSO-*d*$_6$), ^{13}**C {**^{19}**F} NMR** (151 MHz, DMSO-*d*$_6$), and ^{19}**F NMR** (376 MHz, DMSO-d$_6$) of compound **7k**.

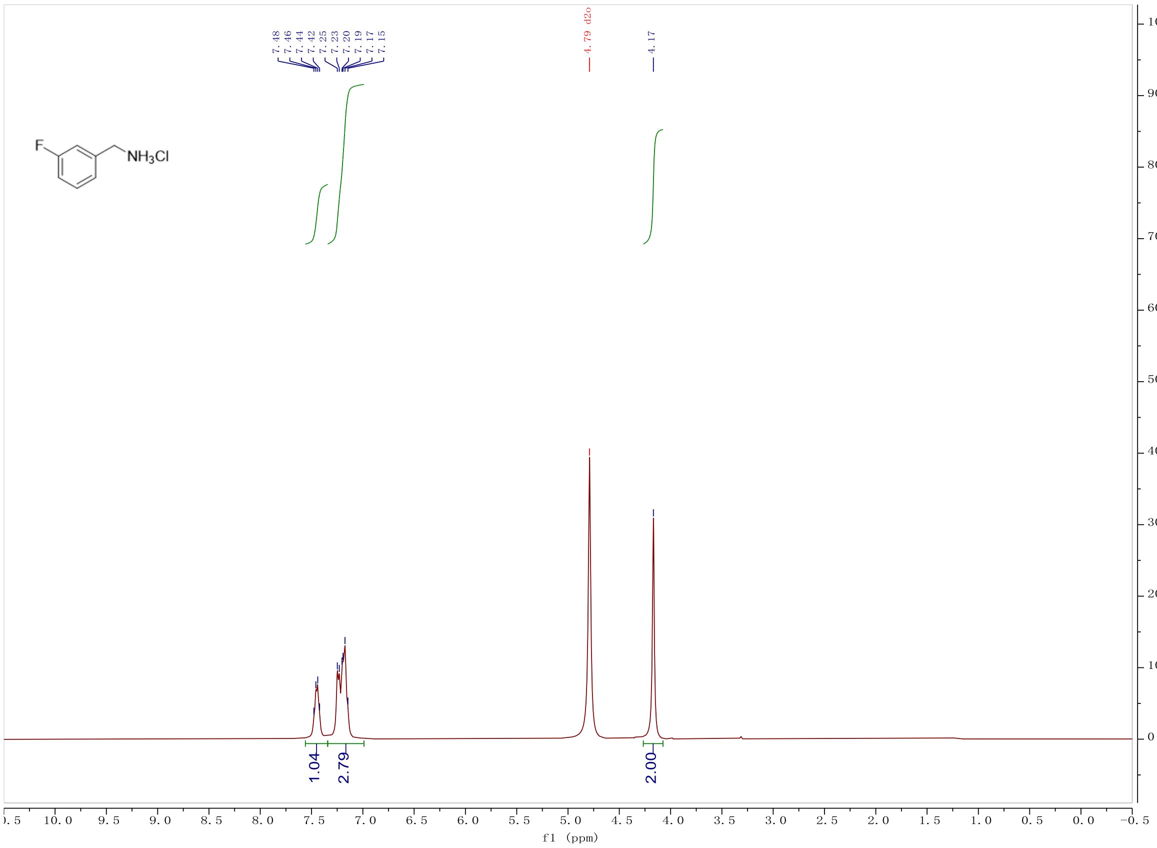
7.48
7.46
7.44
7.42
7.25
7.23
7.20
7.19
7.17
7.15
4.79 d2o
4.17
F
NH3Cl
1.04
2.79
2.00
f1 (ppm)

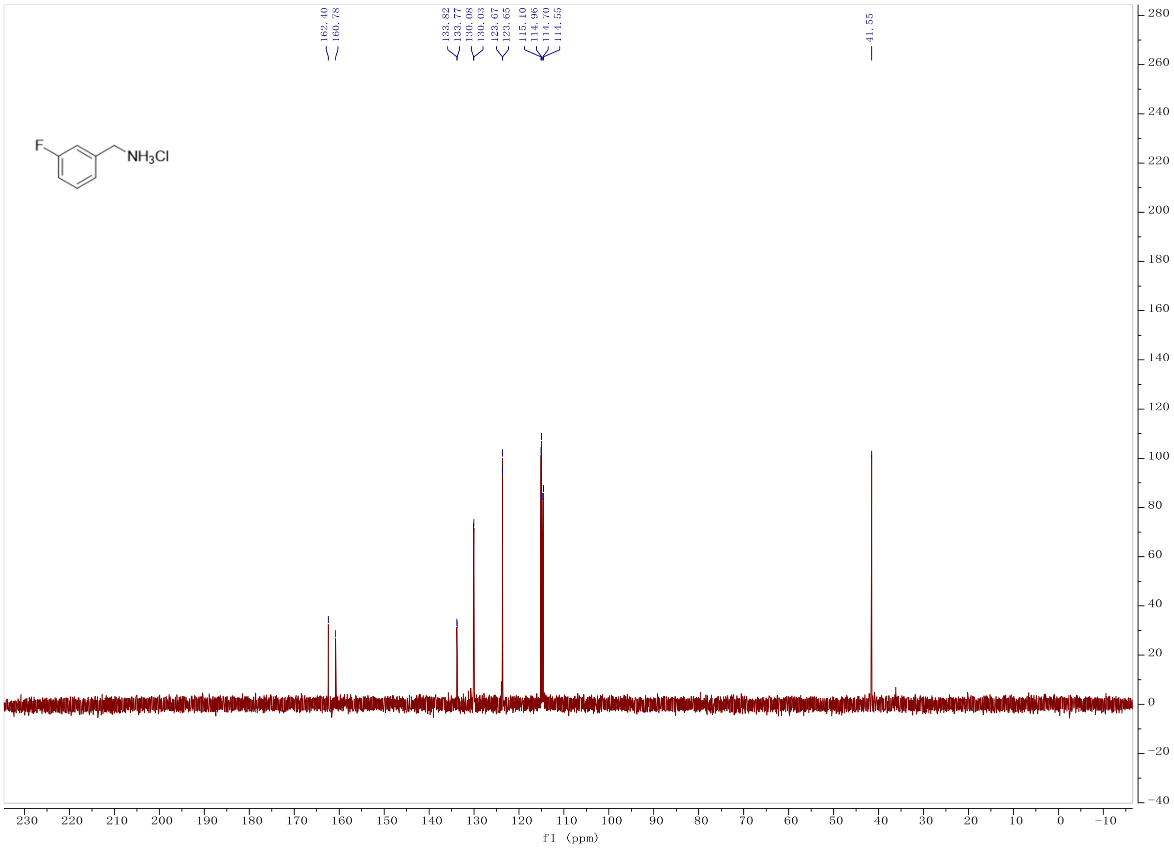
162.40
160.78
133.82
133.77
130.08
130.03
123.67
123.65
115.10
114.96
114.70
114.55
41.55
F
NH3Cl
f1 (ppm)

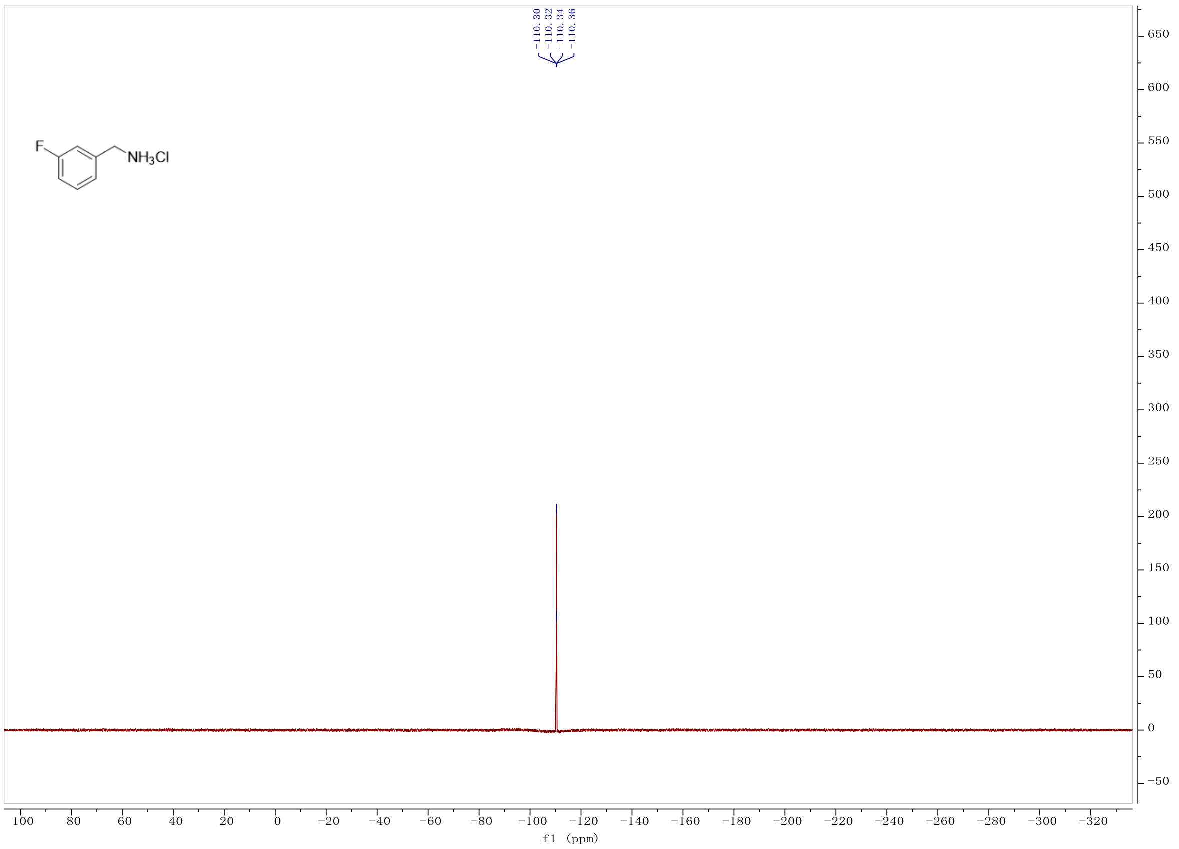

Supplementary Figure 35. **^{1}H NMR** (400 MHz, D_2O), **^{13}C NMR** (151 MHz, D_2O) and **^{19}F NMR** (376 MHz, D_2O) of compound **7l**.

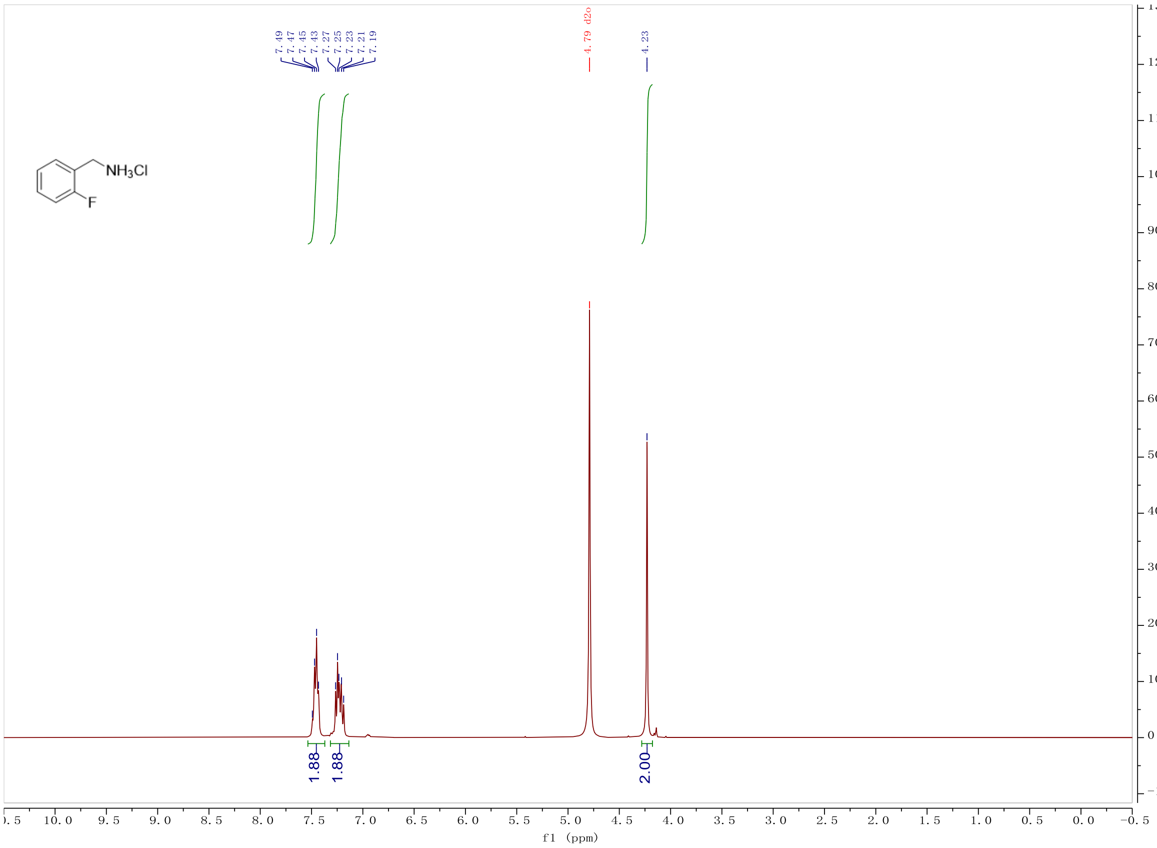

7.49
7.47
7.45
7.43
7.27
7.25
7.23
7.21
7.19
4.79 d2o
4.23
NH3Cl
F
1.88
1.88
2.00
f1 (ppm)

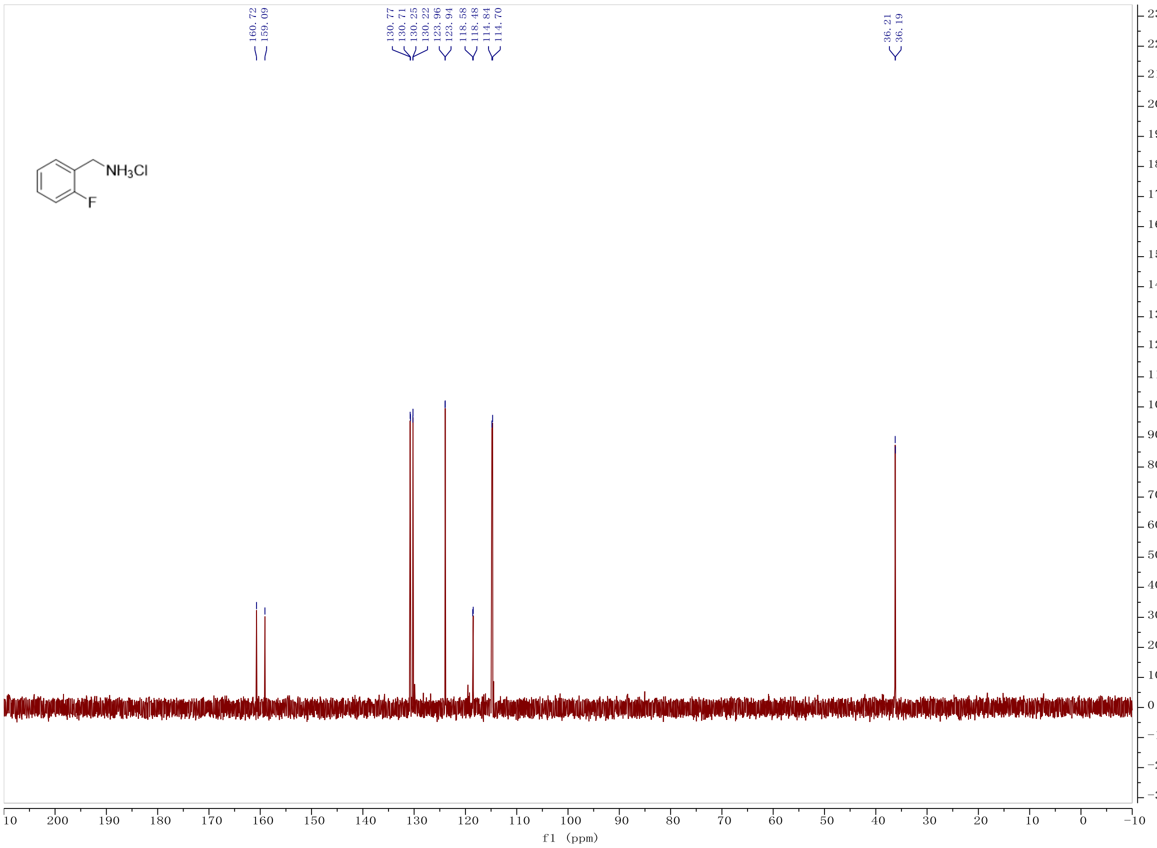

160.72
159.09
130.77
130.71
130.25
130.22
123.96
123.94
118.58
118.48
114.84
114.70
36.21
36.19
NH3Cl
F
f1 (ppm)

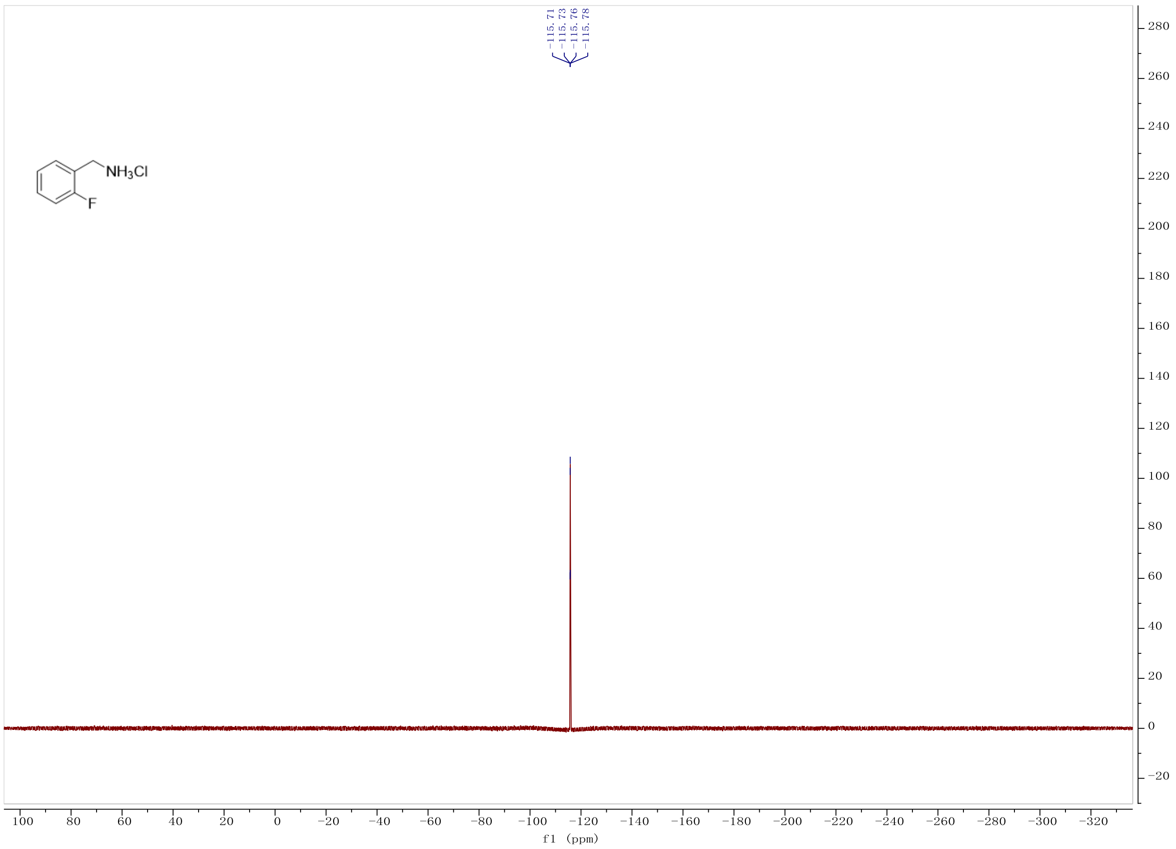

Supplementary Figure 36. **^{1}H NMR** (400 MHz, D_2O), **^{13}C NMR** (151 MHz, D_2O) and **^{19}F NMR** (376 MHz, D_2O) of compound **7m**.

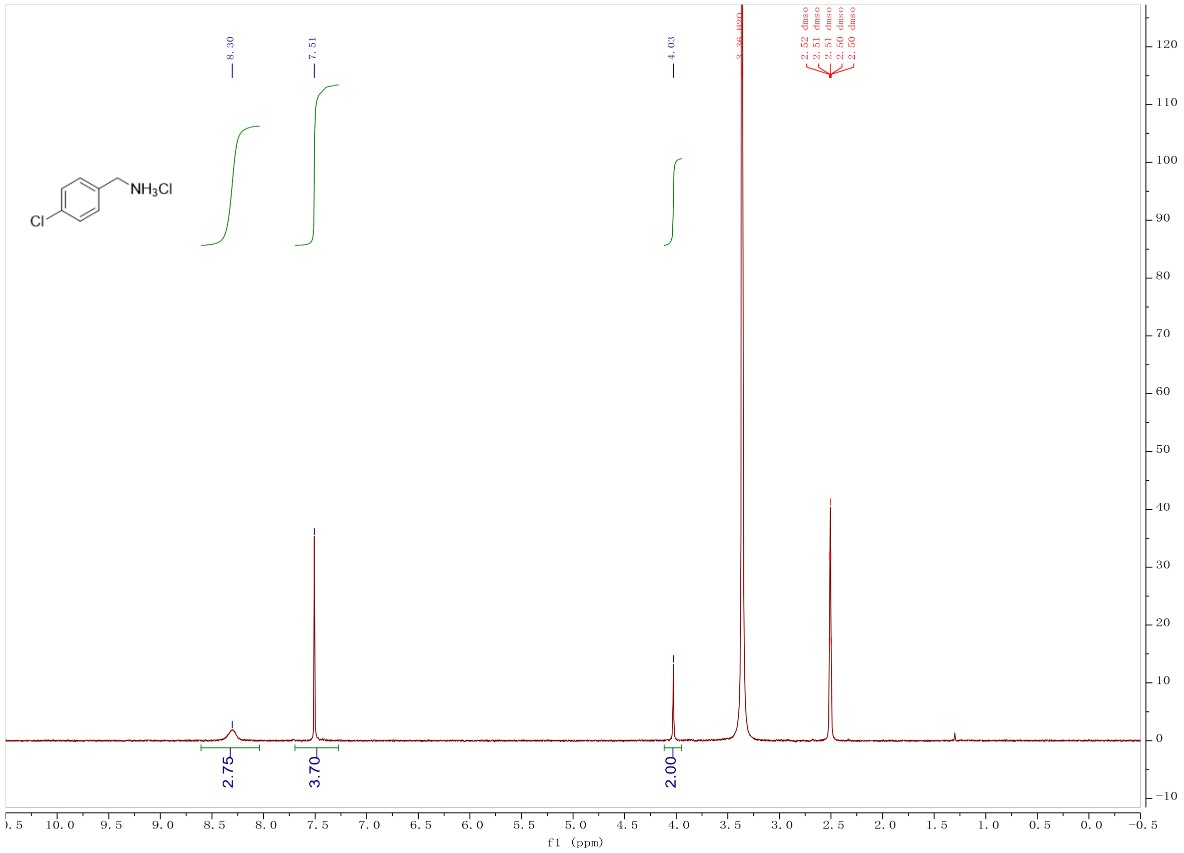

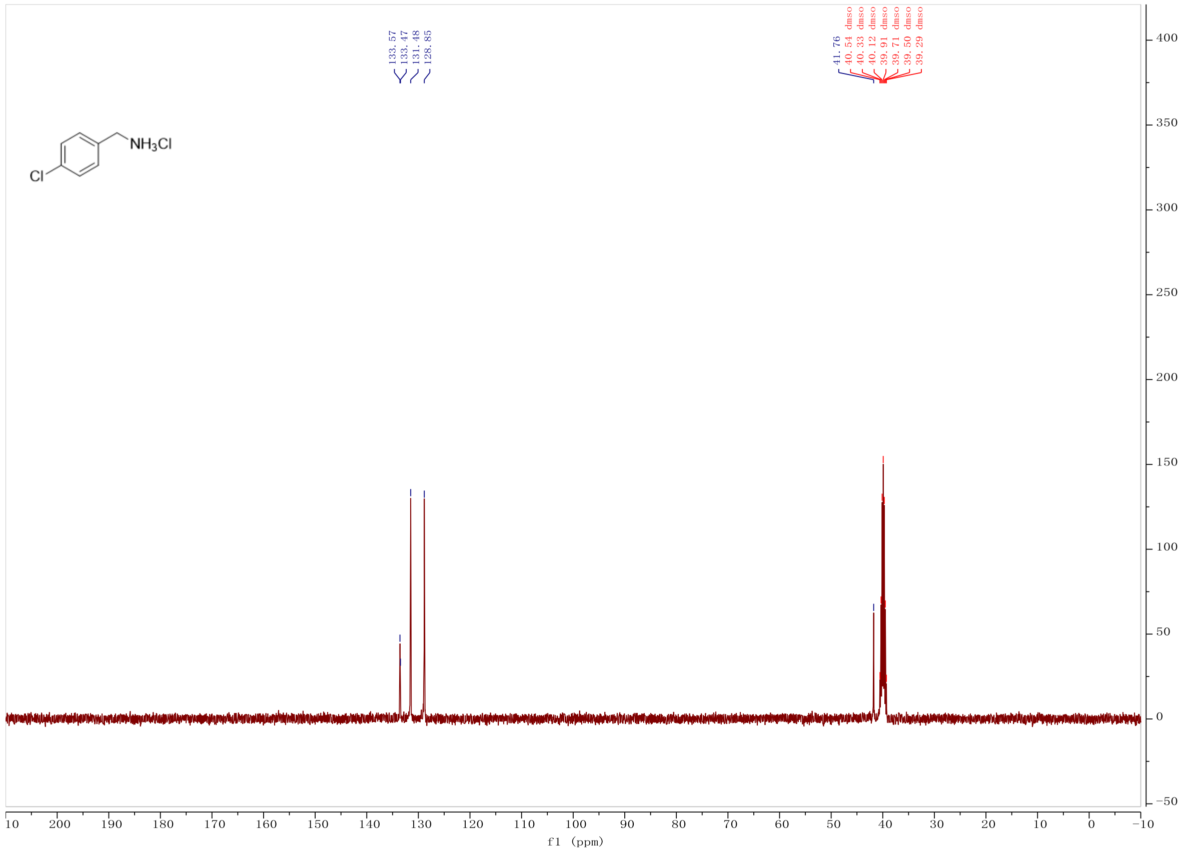

Supplementary Figure 37. ^{1}**H NMR** (400 MHz, DMSO-d_6) and ^{13}**C NMR** (101 MHz, DMSO-d_6) of compound **7n**.

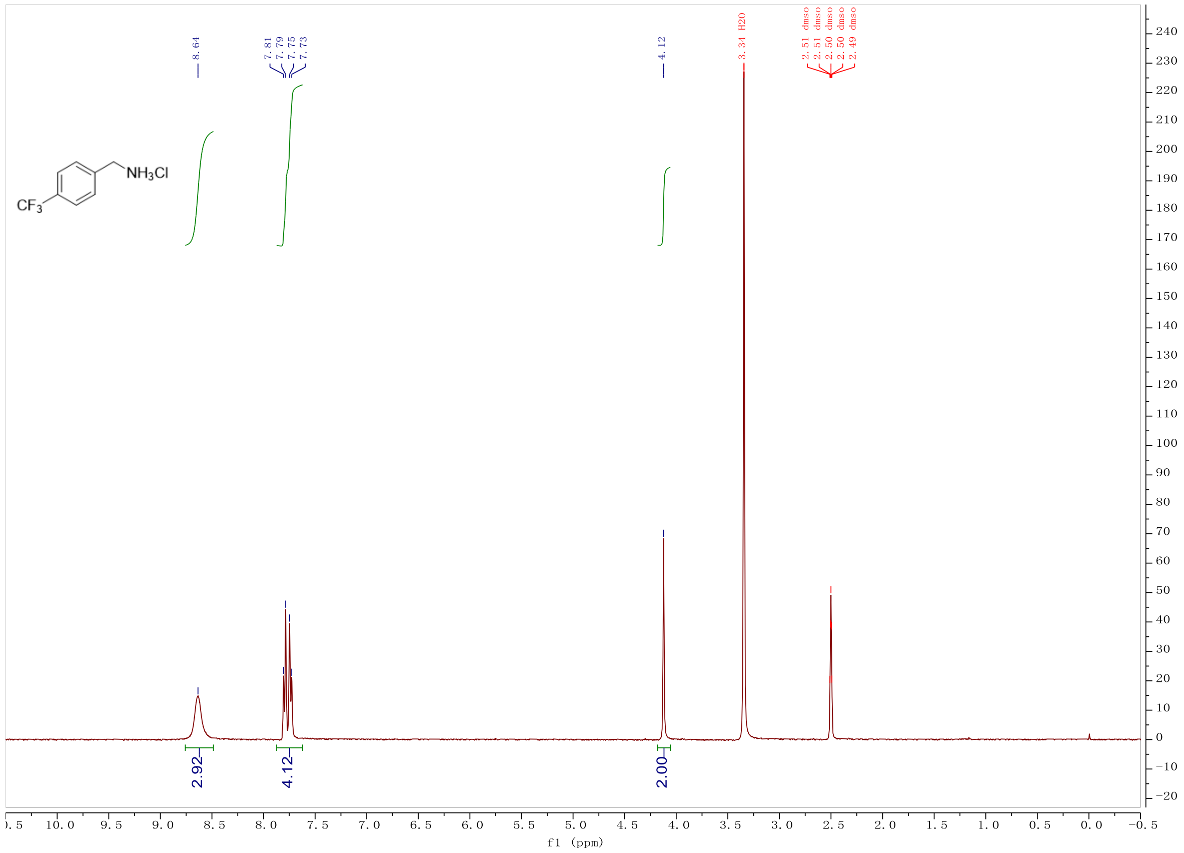
8.64
7.81
7.79
7.75
7.73
4.12
3.34 H2O
2.51 dmso
2.51 dmso
2.50 dmso
2.50 dmso
2.49 dmso
NH3Cl
CF3
2.92
4.12
2.00
f1 (ppm)

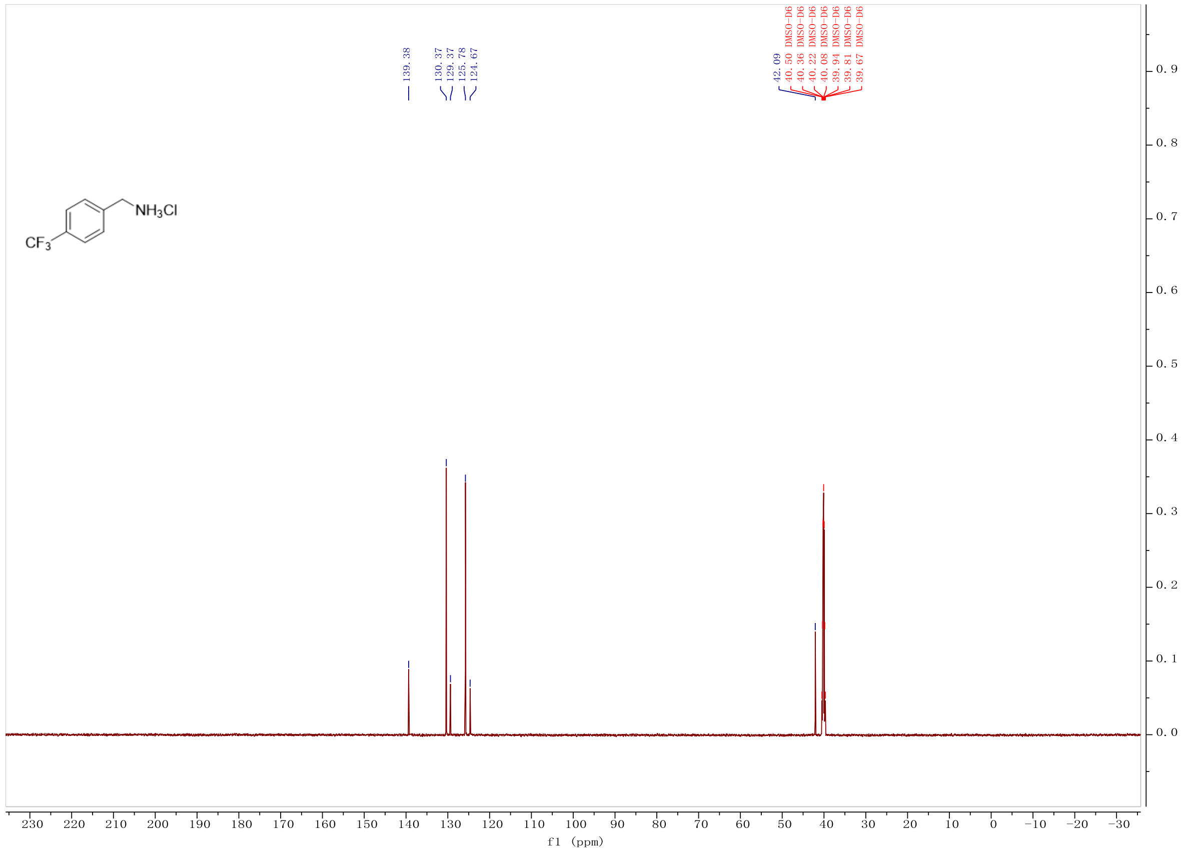
139.38
130.37
129.37
125.78
124.67
42.09
40.50 DMSO-D6
40.36 DMSO-D6
40.22 DMSO-D6
40.08 DMSO-D6
39.94 DMSO-D6
39.81 DMSO-D6
39.67 DMSO-D6
NH3Cl
CF3
f1 (ppm)

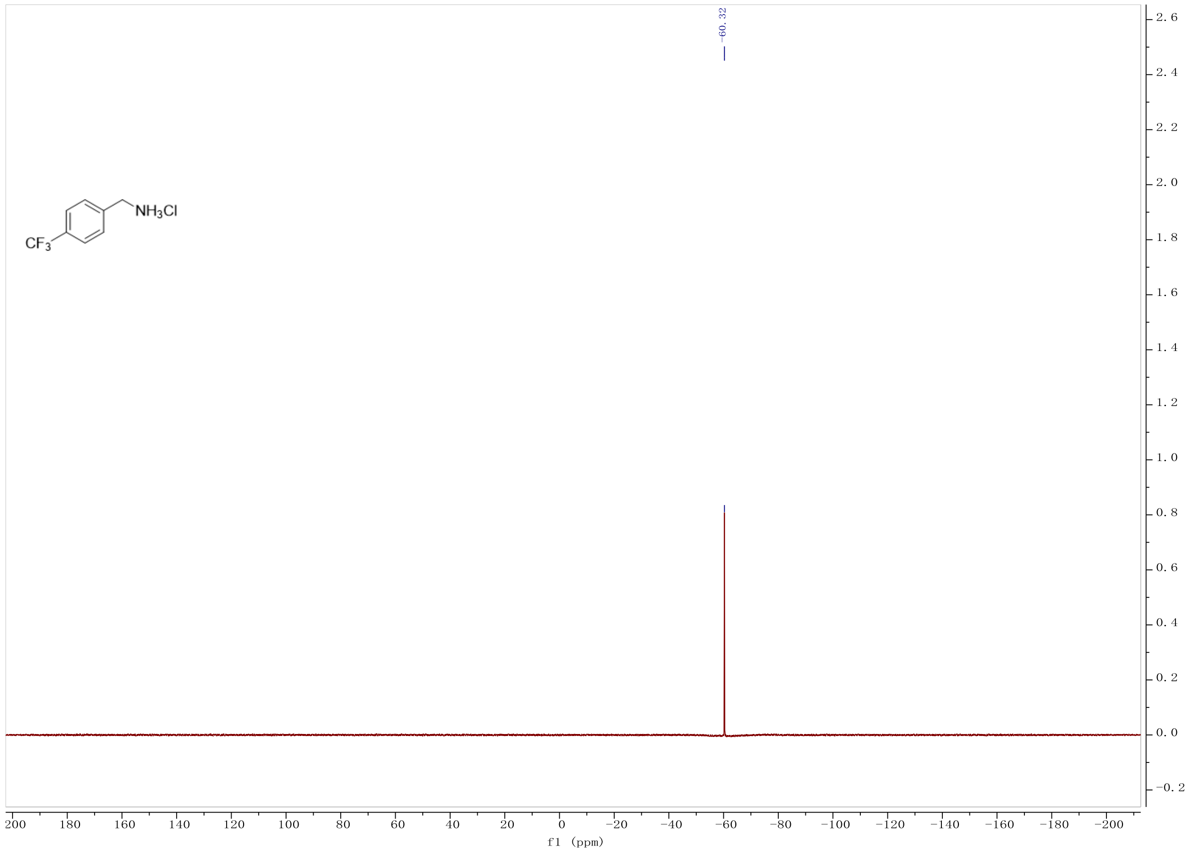

Supplementary Figure 38. **^{1}H NMR** (400 MHz, DMSO-d_6), **^{13}C {^{19}F} NMR** (151 MHz, DMSO-d_6), and **^{19}F NMR** (376 MHz, DMSO-d_6) of compound **7o**.

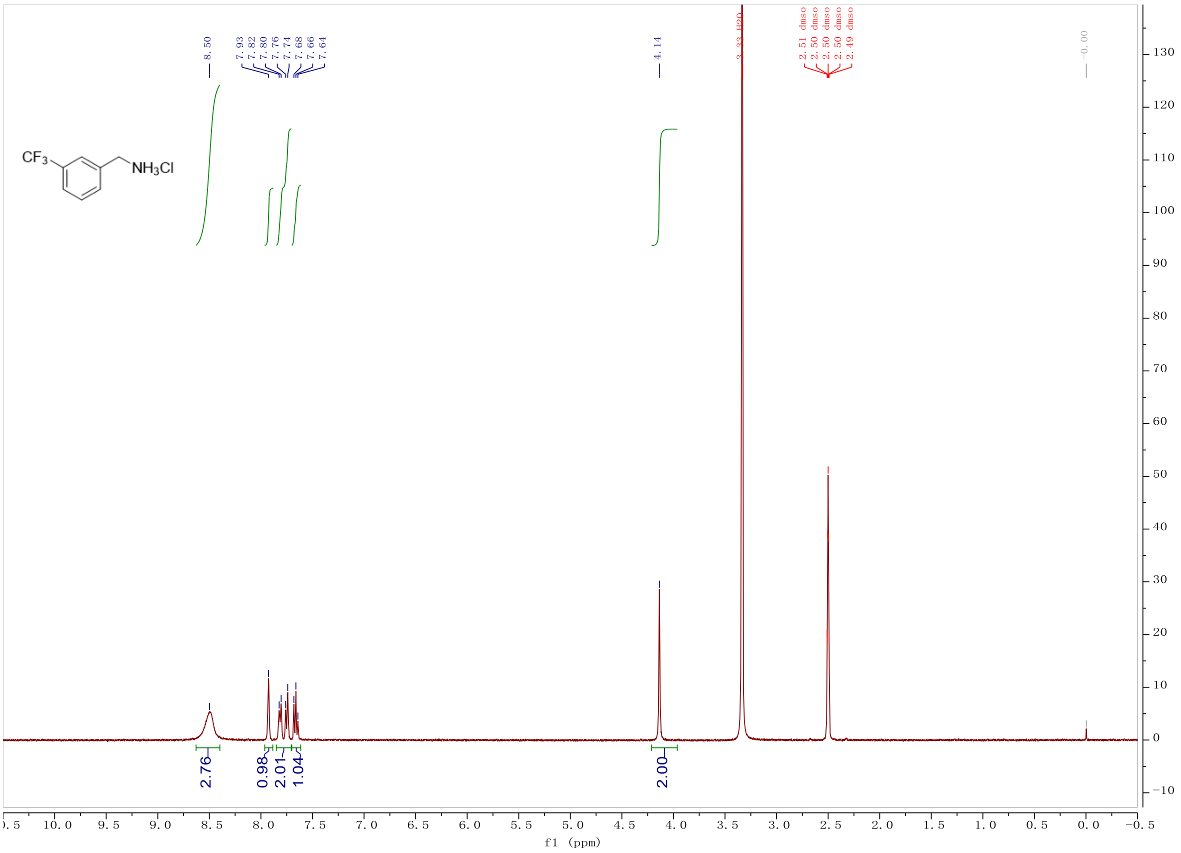

8.50
7.93
7.82
7.80
7.76
7.74
7.68
7.66
7.64
4.14
3.33 H2O
2.51 dmso
2.50 dmso
2.50 dmso
2.50 dmso
2.49 dmso
-0.00
CF3
NH3Cl
2.76
0.98
2.01
1.04
2.00
f1 (ppm)

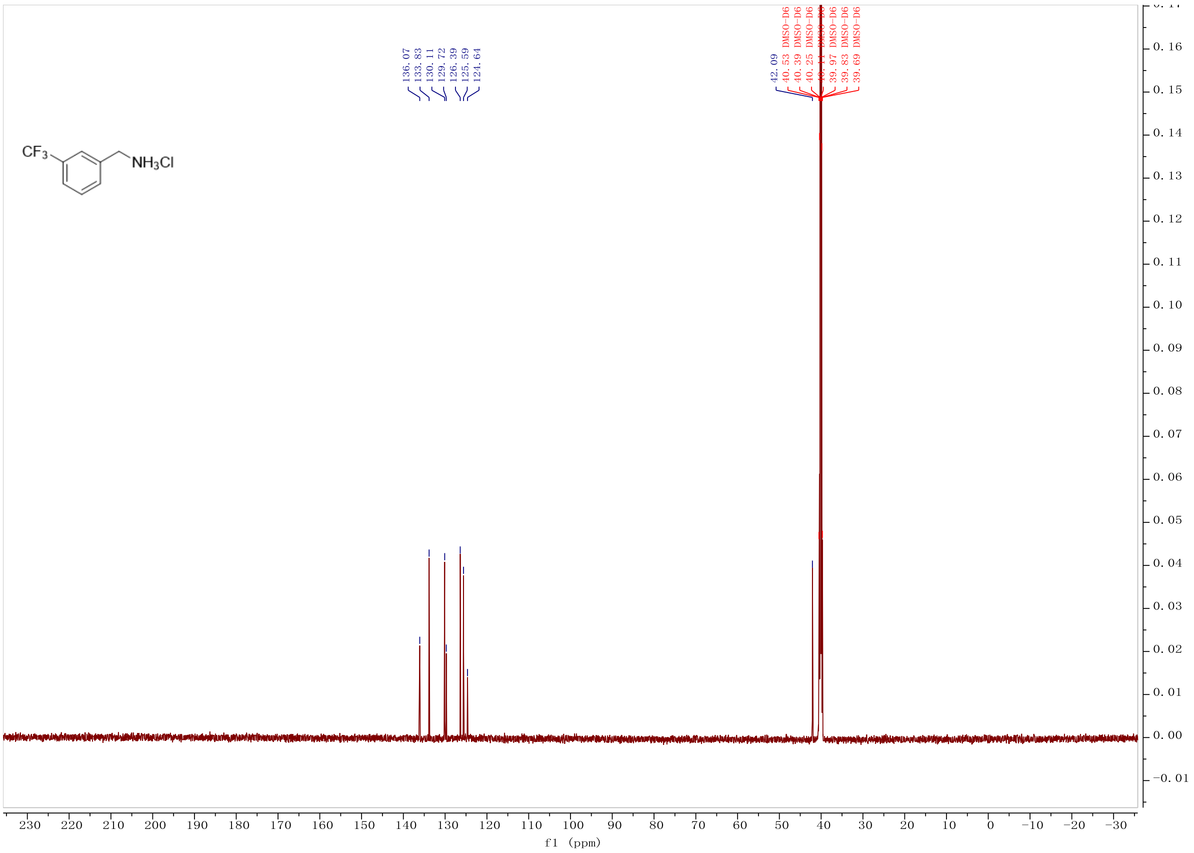

136.07
133.83
130.11
129.72
126.39
125.59
124.64
42.09
40.53 DMSO-D6
40.39 DMSO-D6
40.25 DMSO-D6
39.97 DMSO-D6
39.83 DMSO-D6
39.69 DMSO-D6
CF3
NH3Cl
f1 (ppm)

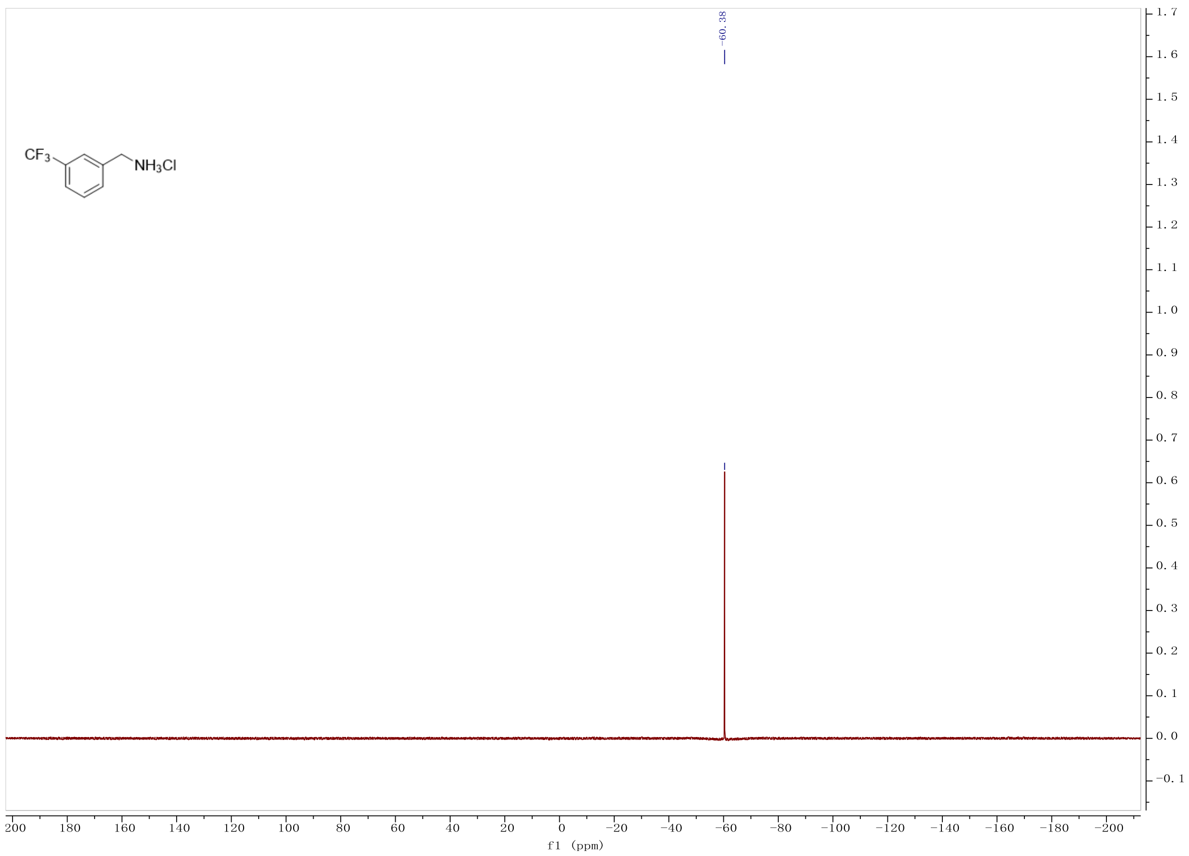

Supplementary Figure 39. **^{1}H NMR** (400 MHz, DMSO-*d*$_6$), **^{13}C {^{19}F} NMR** (151 MHz, DMSO-*d*$_6$), and **^{19}F NMR** (376 MHz, DMSO-d$_6$) of compound **7p**.

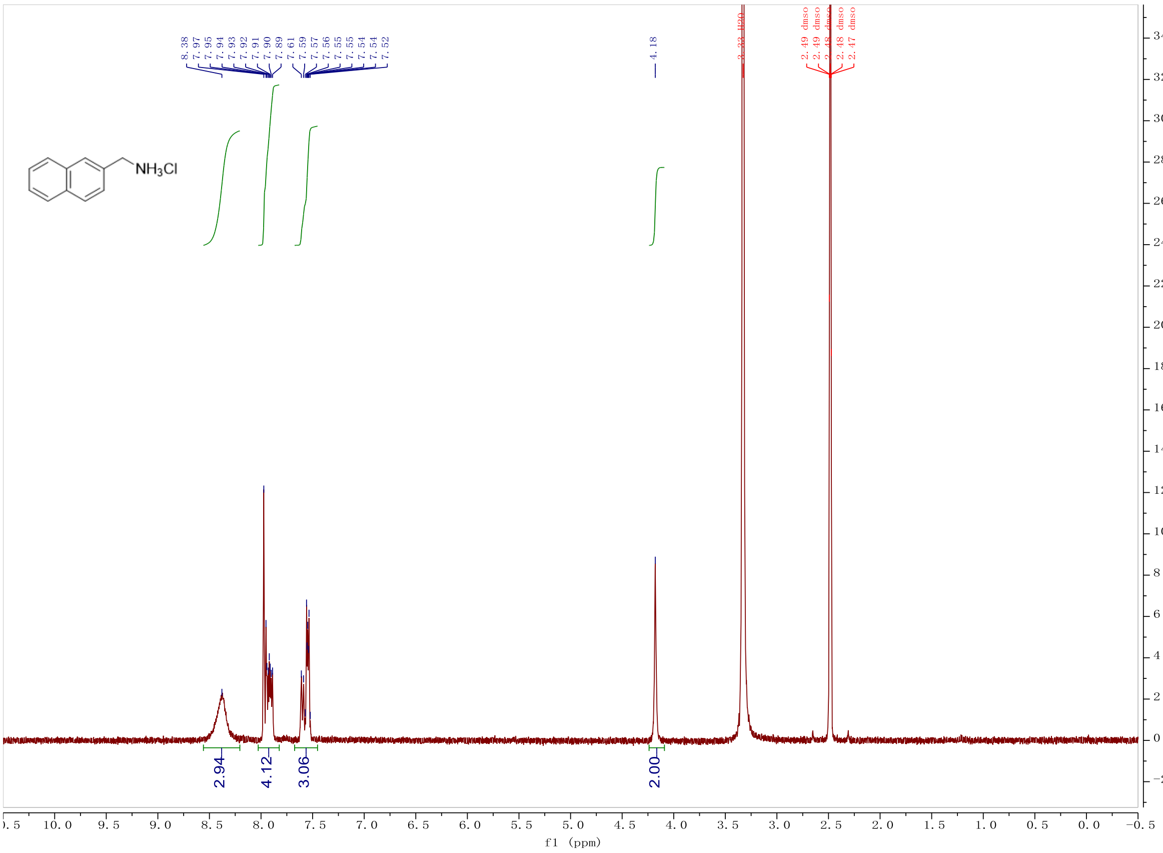

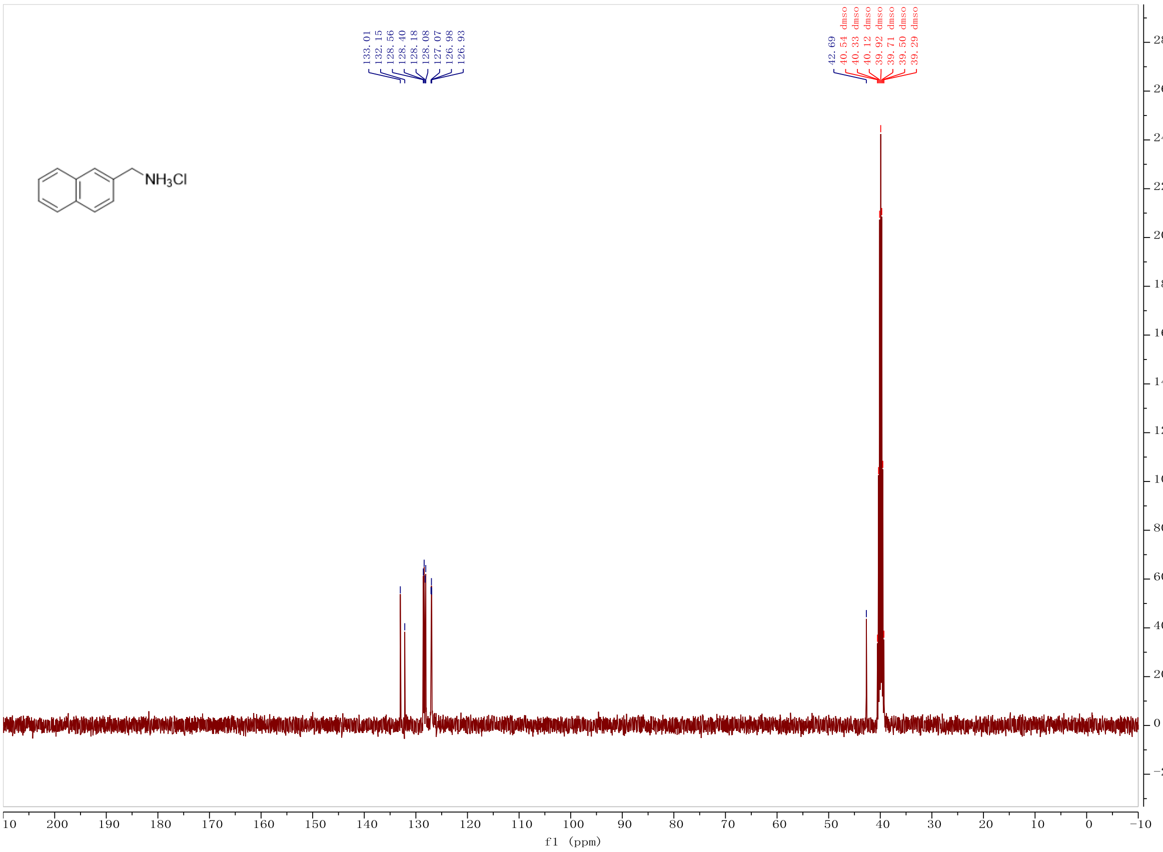

Supplementary Figure 40. ^{1}**H NMR** (400 MHz, DMSO-d_6) and ^{13}**C NMR** (101 MHz, DMSO-d_6) of compound **7q**.

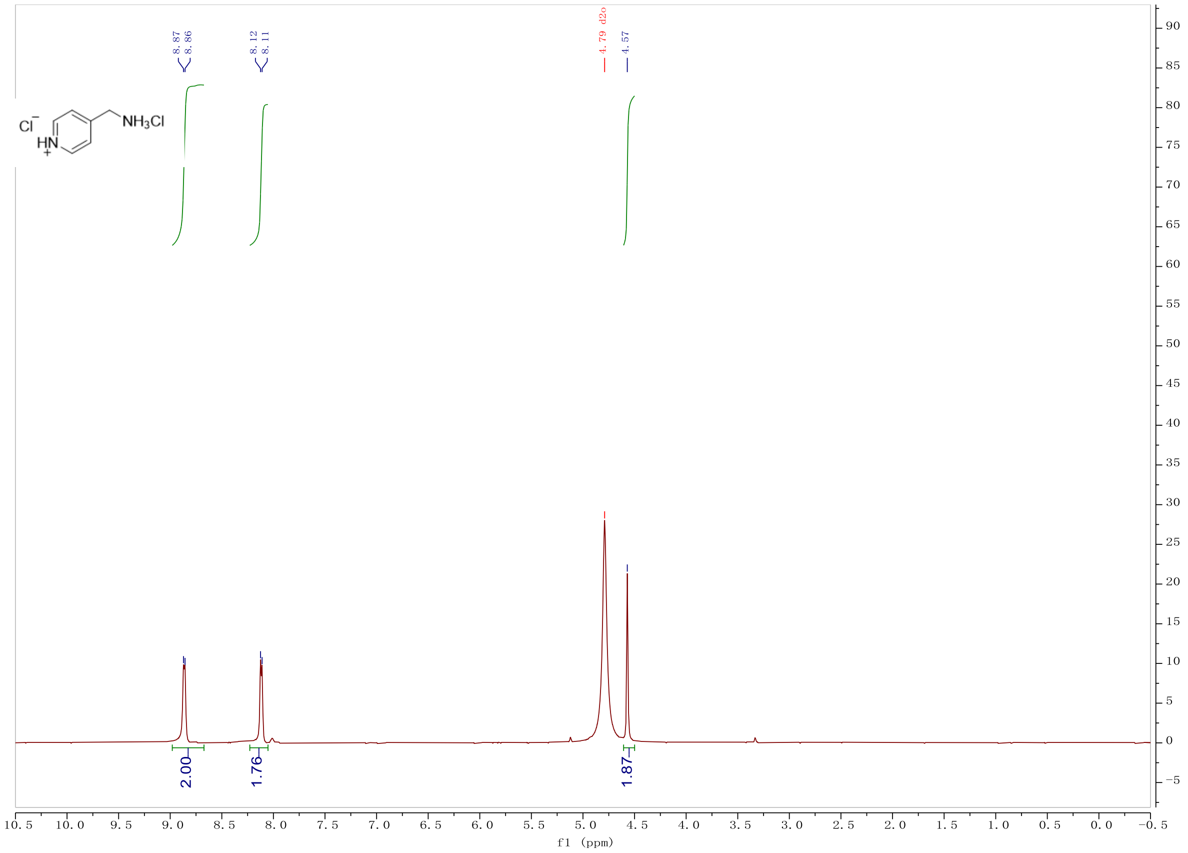

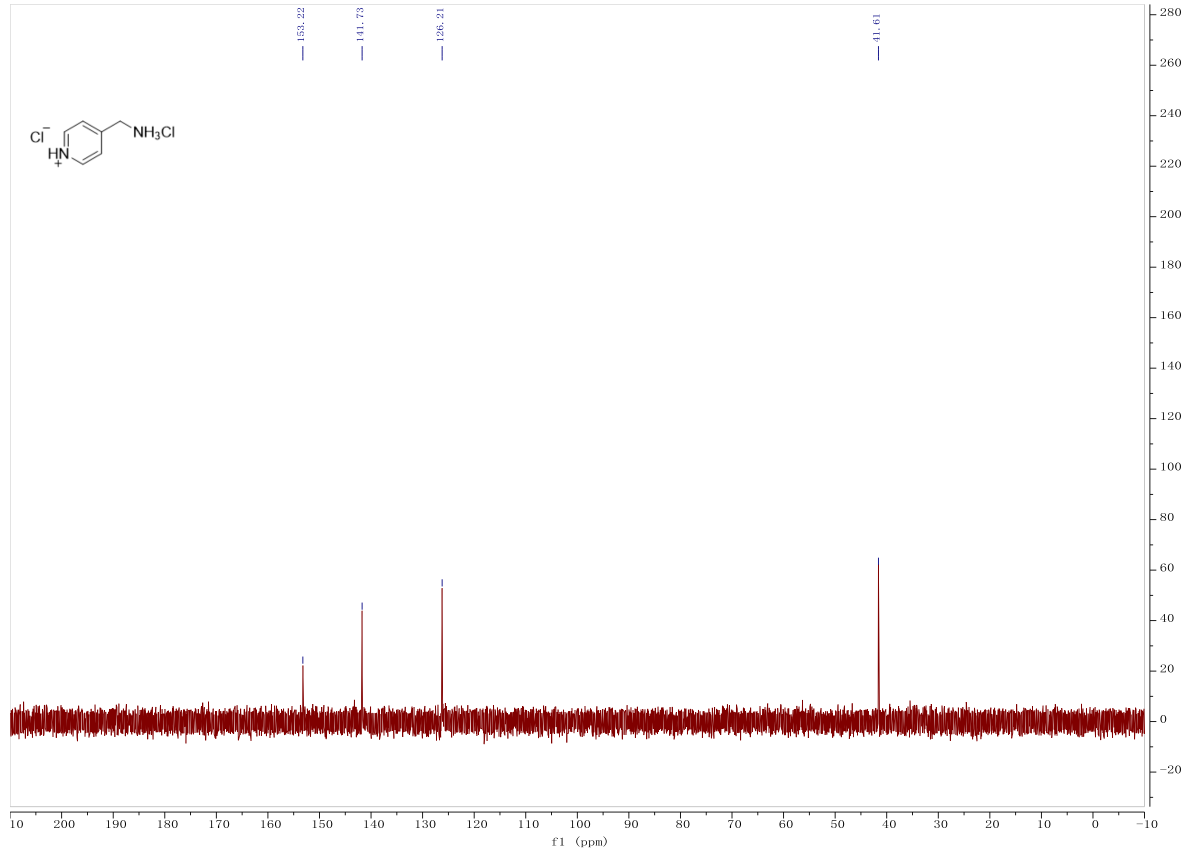

Supplementary Figure 41. **^{1}H NMR** (400 MHz, D_2O) and **^{13}C NMR** (101 MHz, D_2O) of compound **7r**.

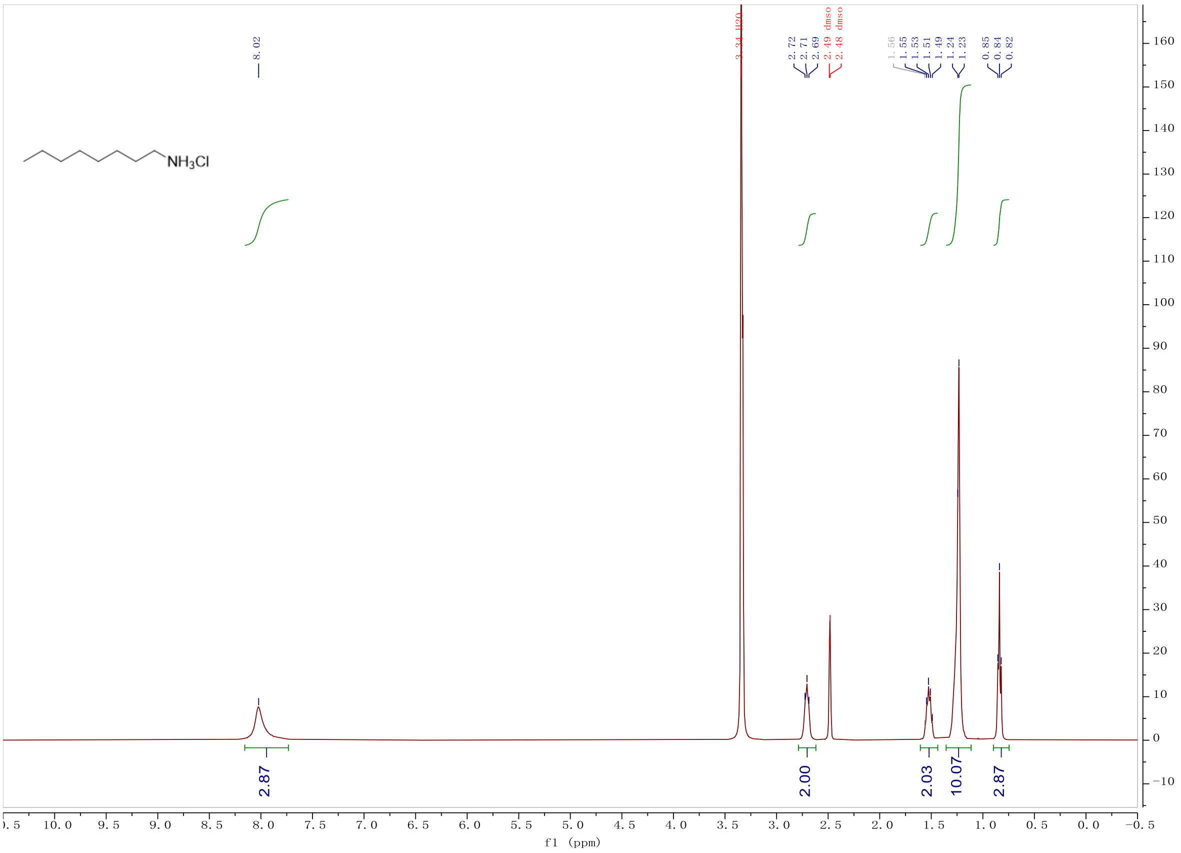

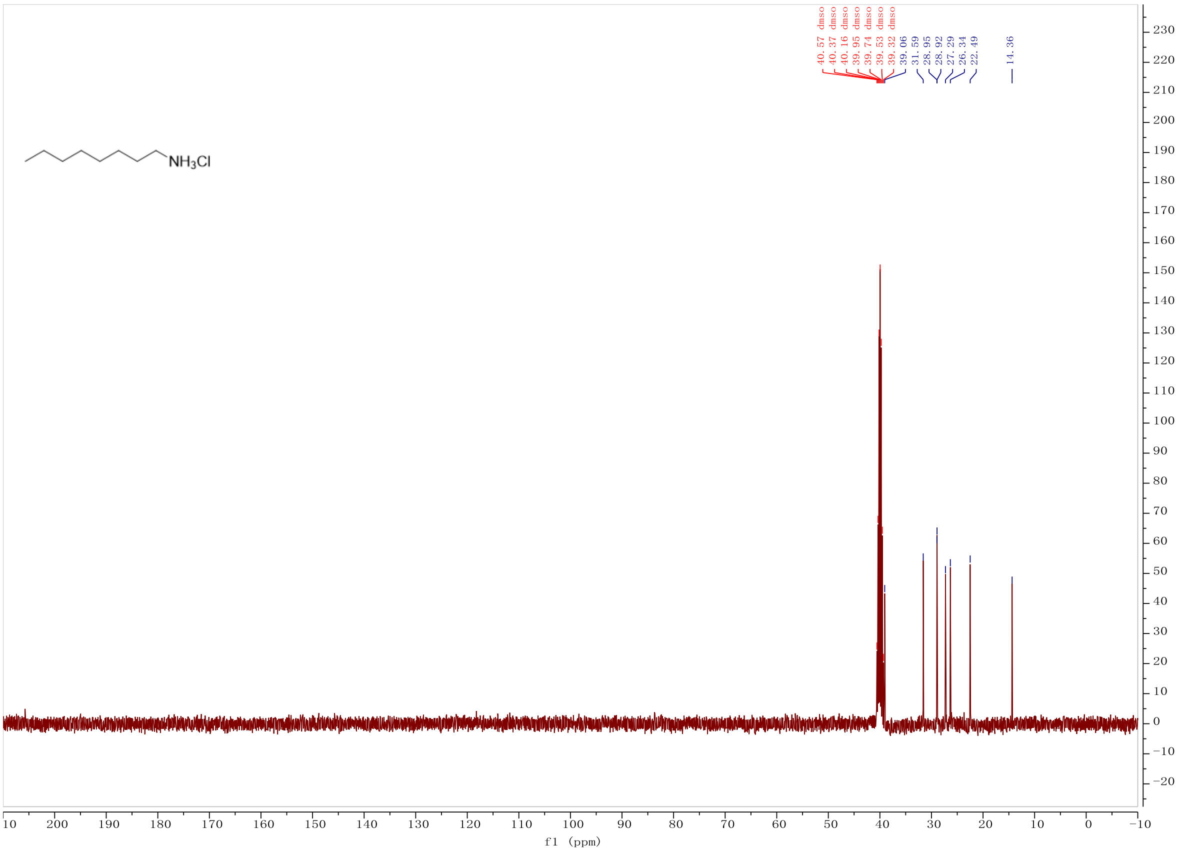

Supplementary Figure 42. **^{1}H NMR** (400 MHz, DMSO-d_6) and **^{13}C NMR** (101 MHz, DMSO-d_6) of compound **7s**.

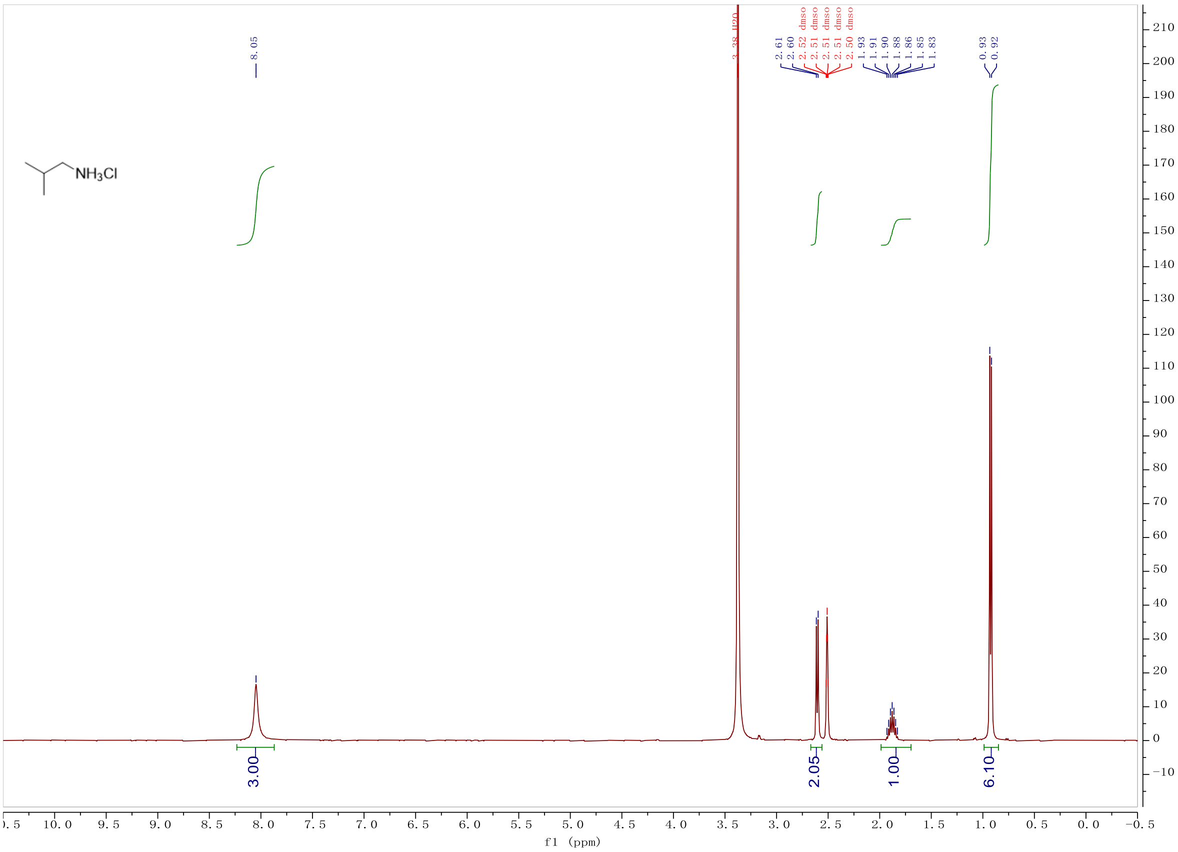

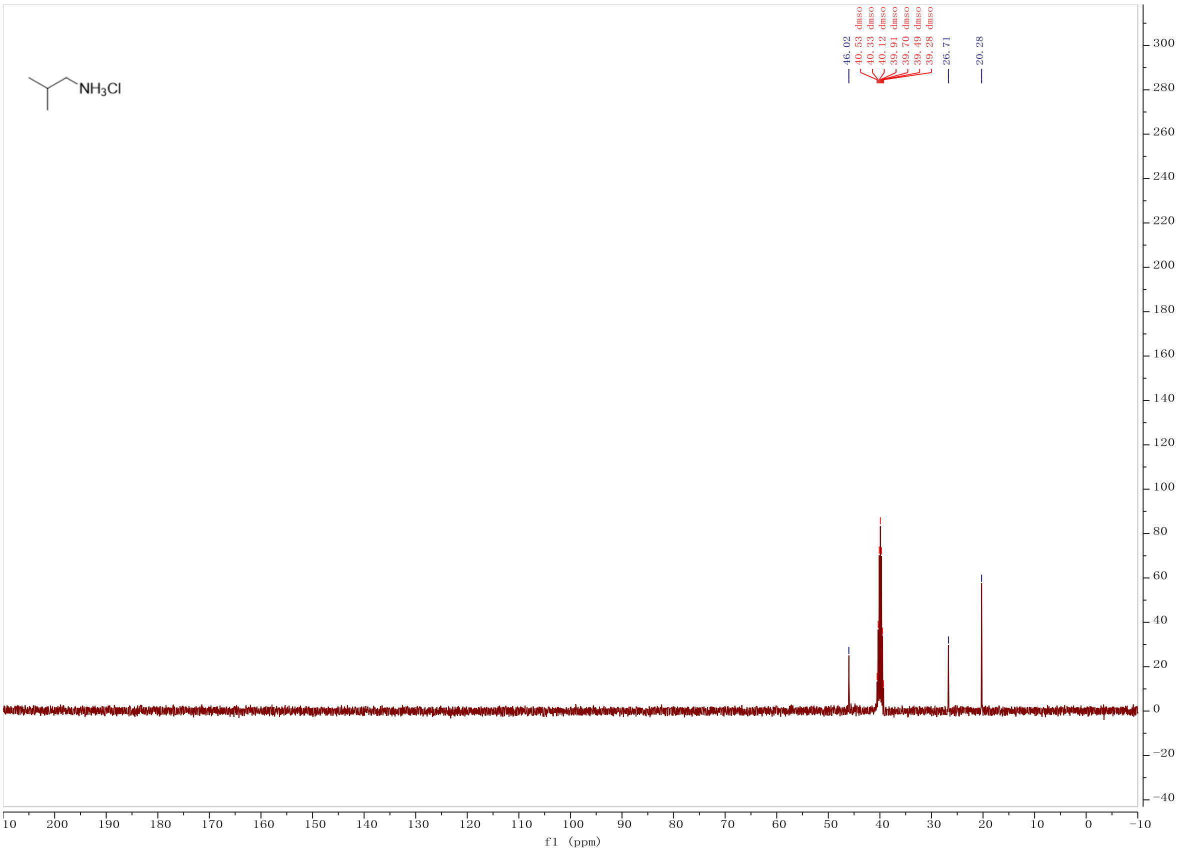

Supplementary Figure 43. **^{1}H NMR** (400 MHz, DMSO-d_6) and **^{13}C NMR** (101 MHz, DMSO-d_6) of compound **7t**.

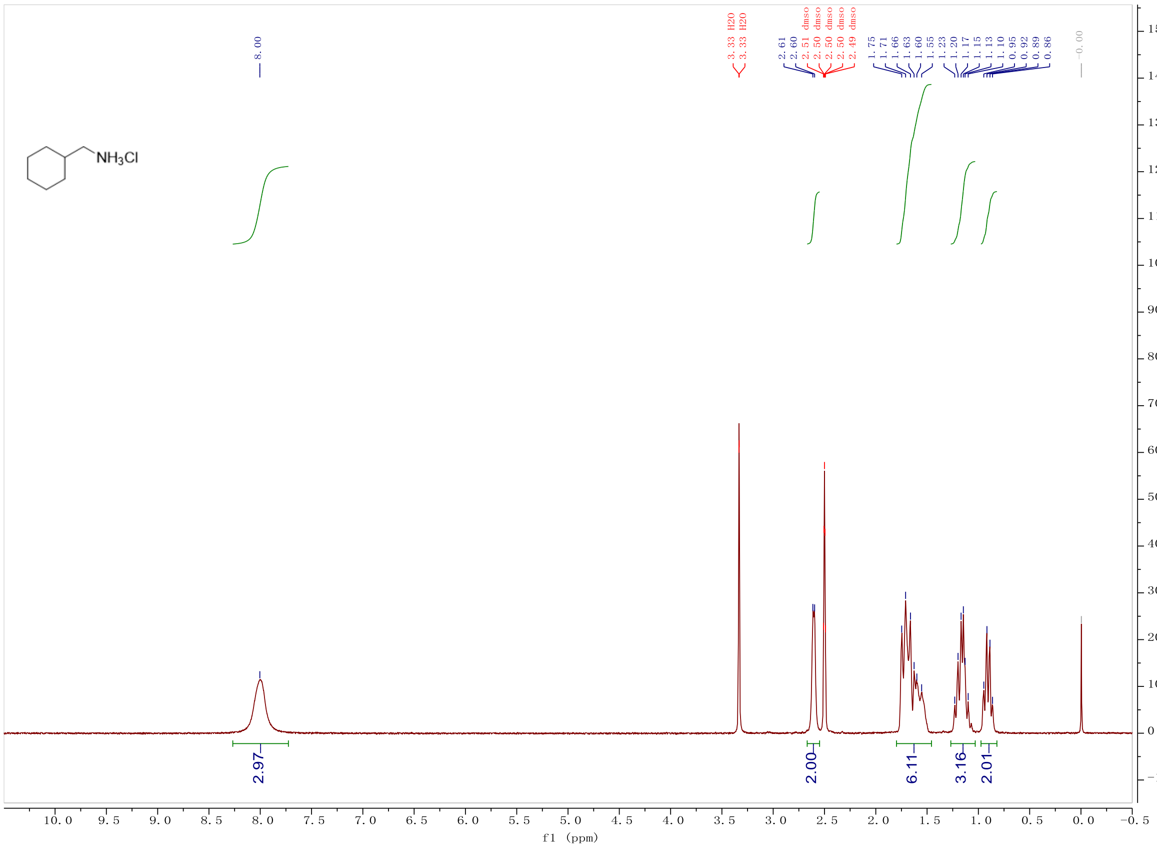

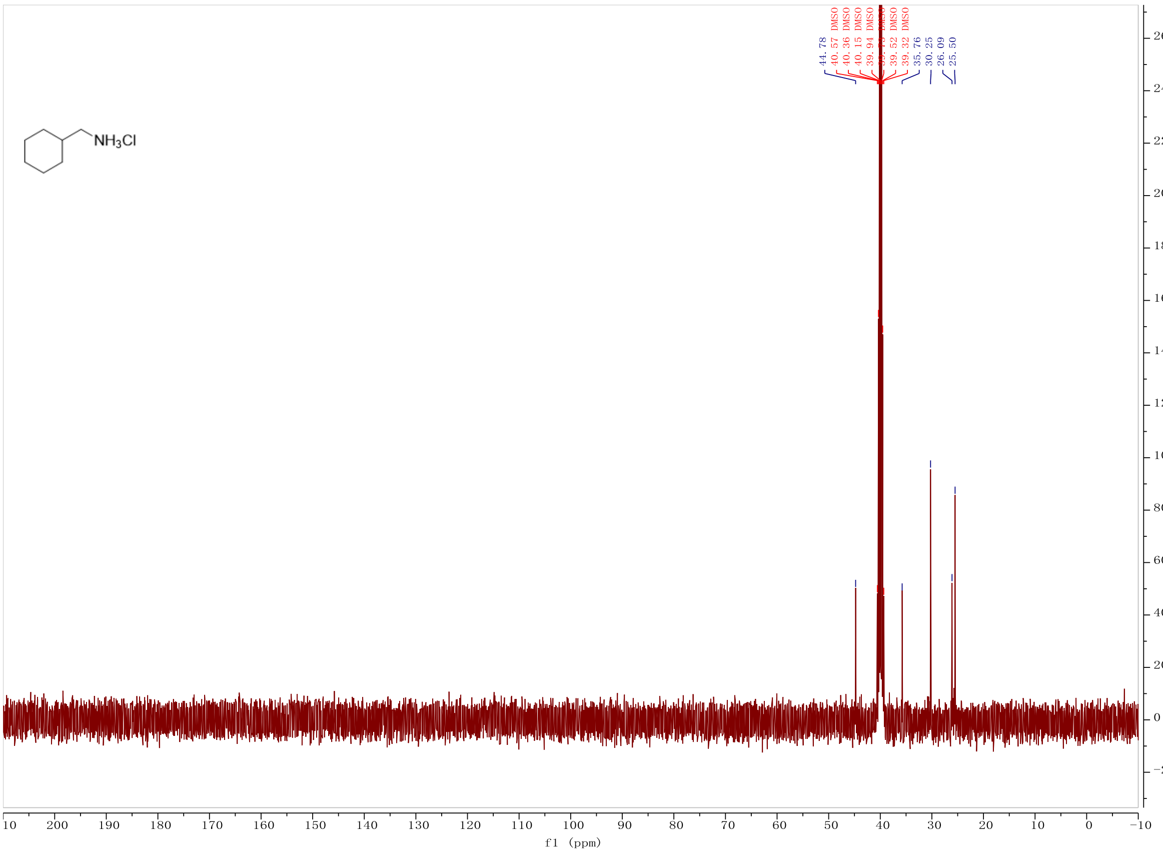

Supplementary Figure 44. **¹H NMR** (400 MHz, DMSO-d_6) and **¹³C NMR** (101 MHz, DMSO-d_6) of compound **7u**.

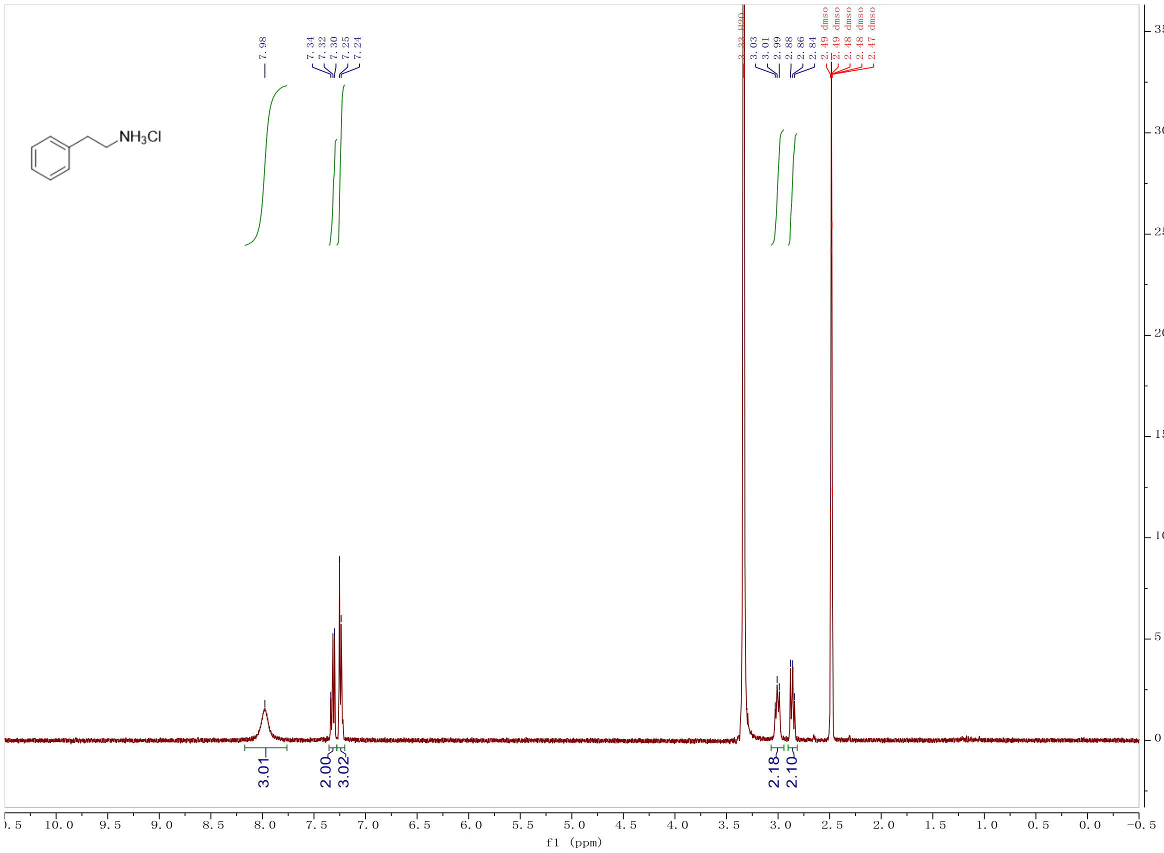

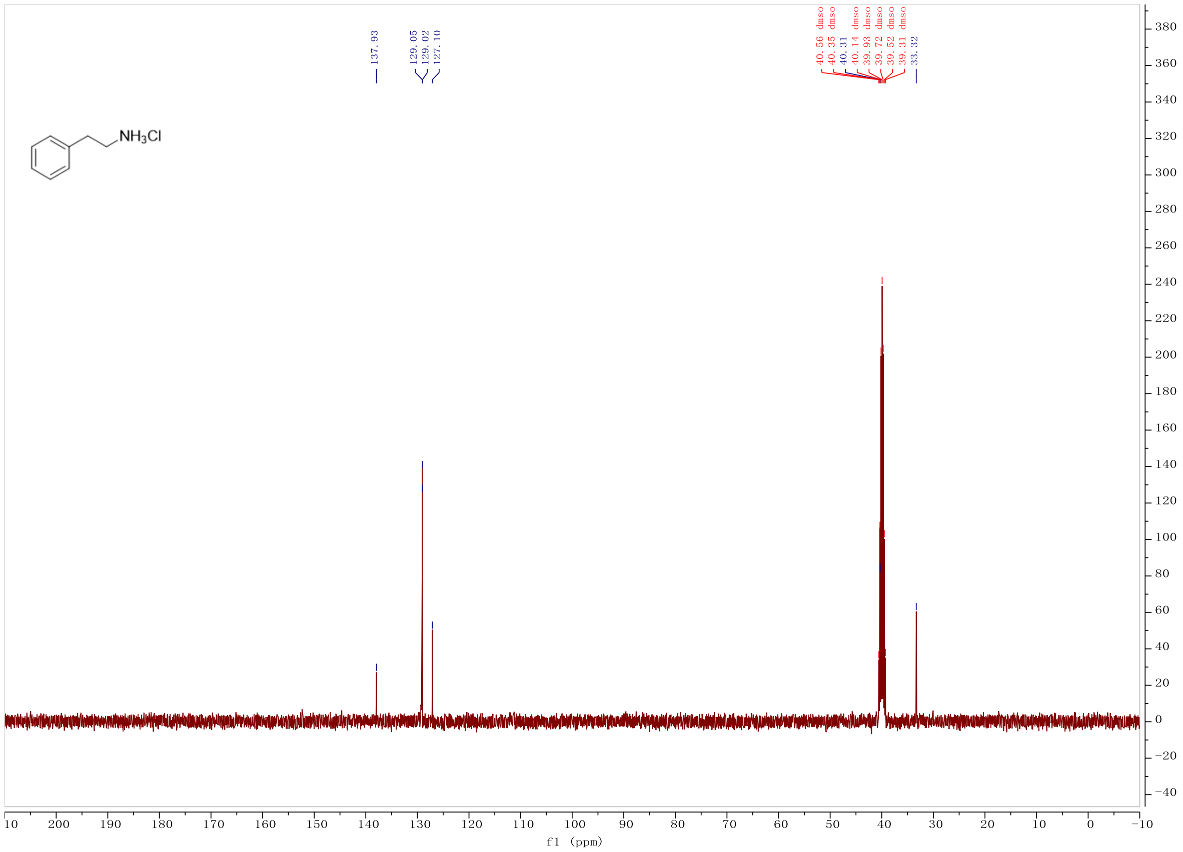

Supplementary Figure 45. **^{1}H NMR** (400 MHz, DMSO-d_6) and **^{13}C NMR** (101 MHz, DMSO-d_6) of compound **7v**.

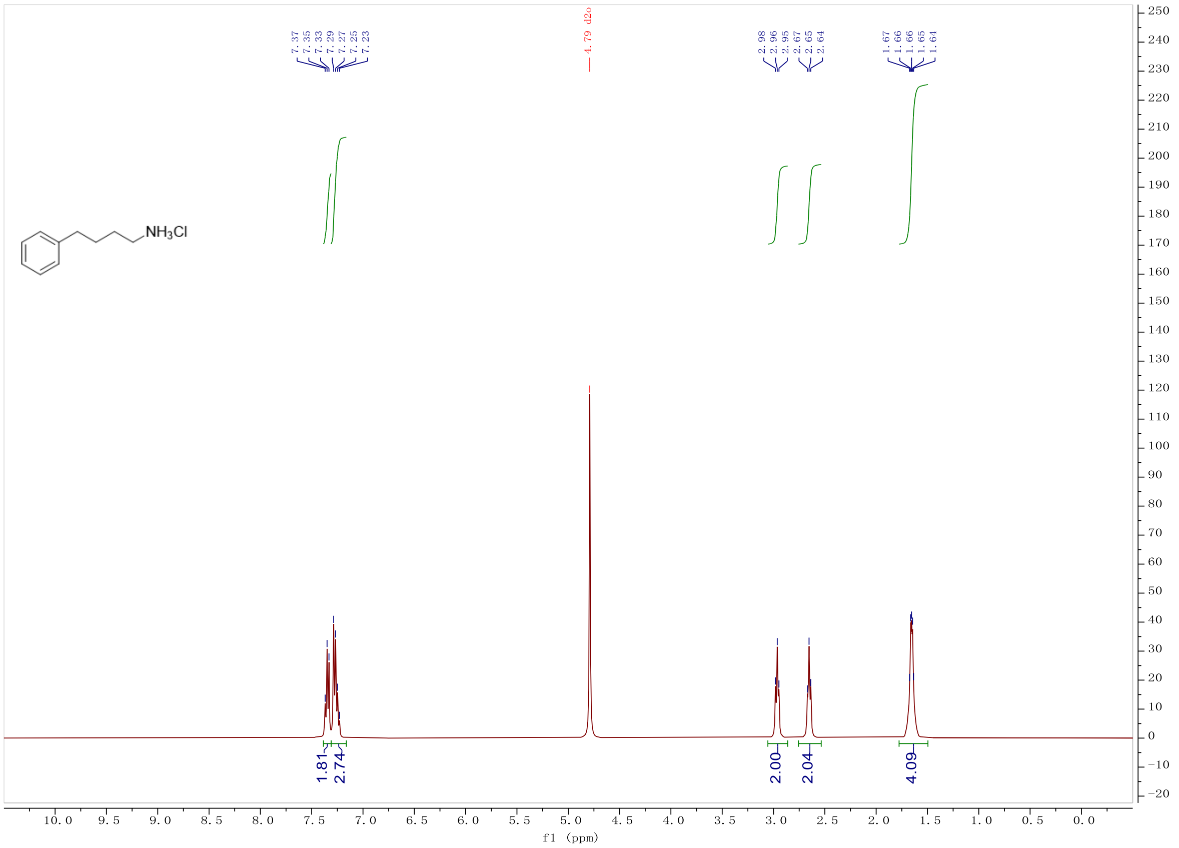

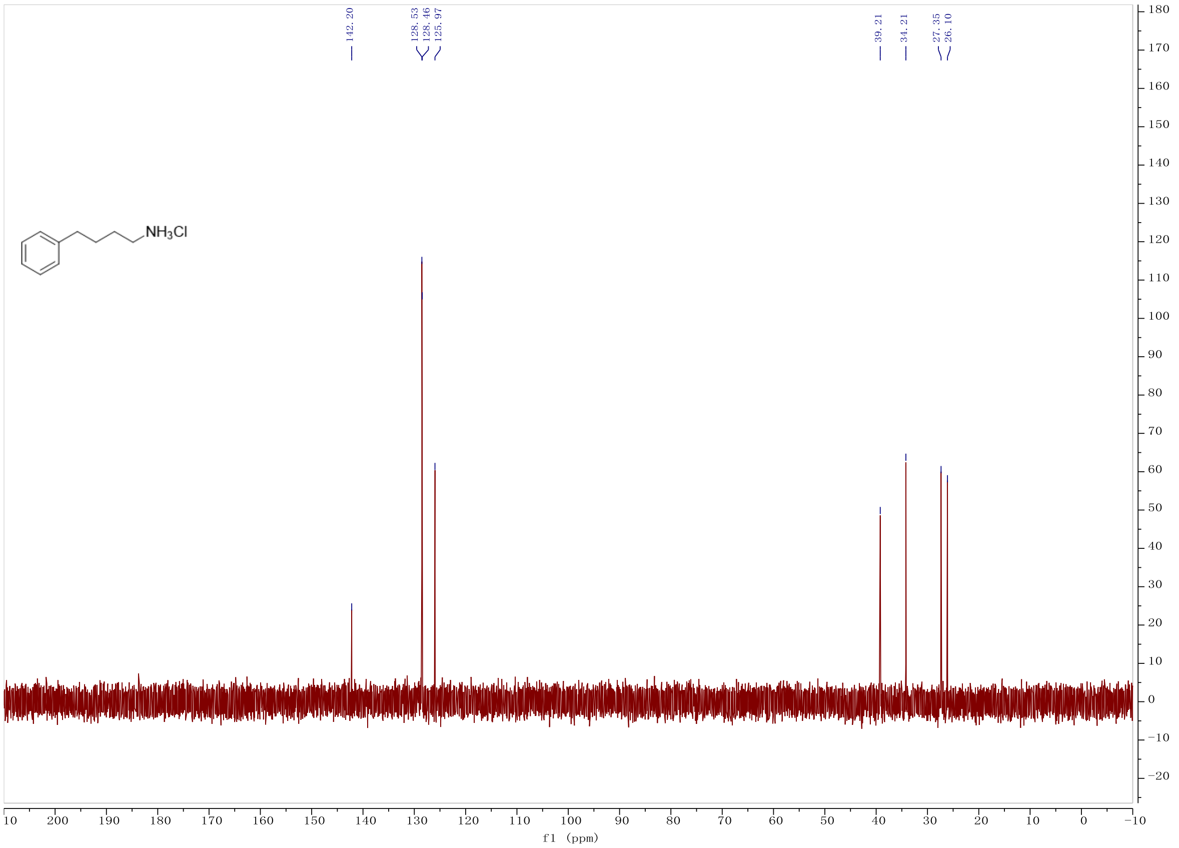

Supplementary Figure 46. ^{1}H NMR (400 MHz, D_2O) and **^{13}C NMR** (101 MHz, D_2O) of compound **7w**.

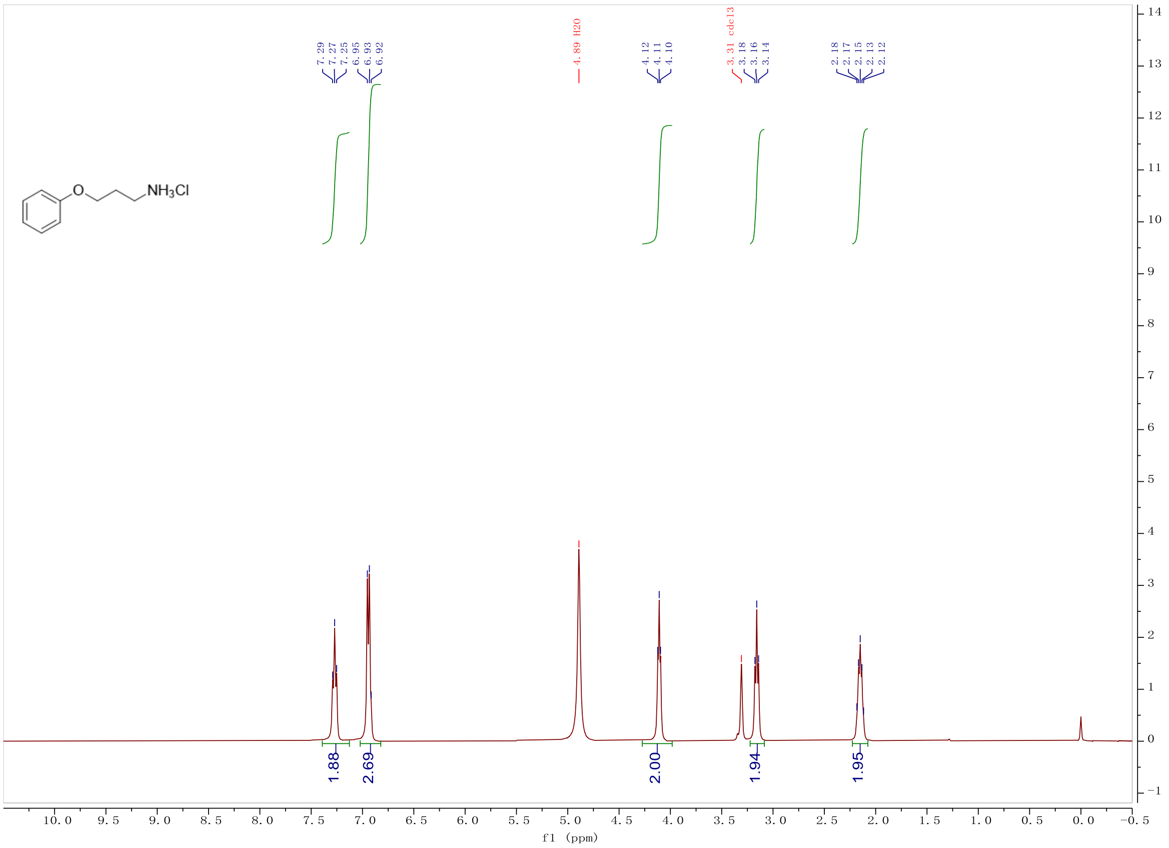

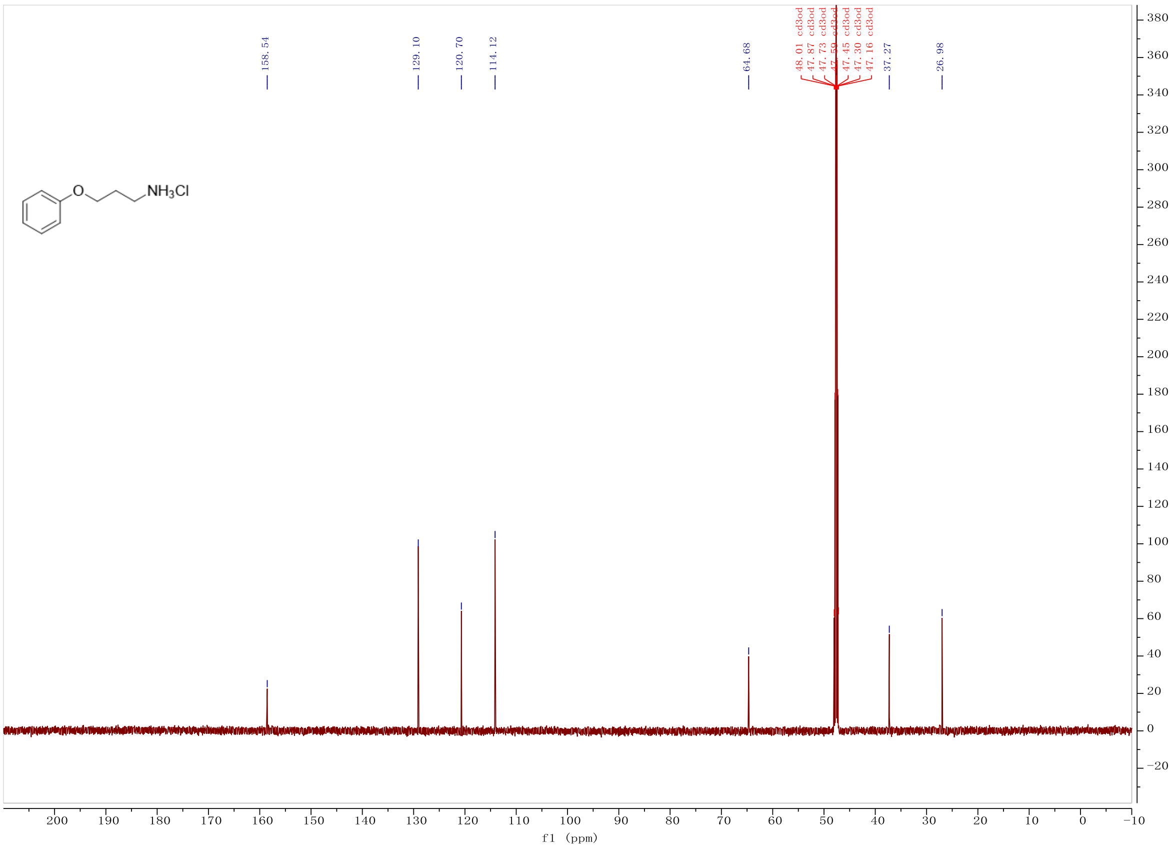

Supplementary Figure 47. **^{1}H NMR** (400 MHz, CD_3OD) and **^{13}C NMR** (151 MHz, CD_3OD) of compound **7x**.

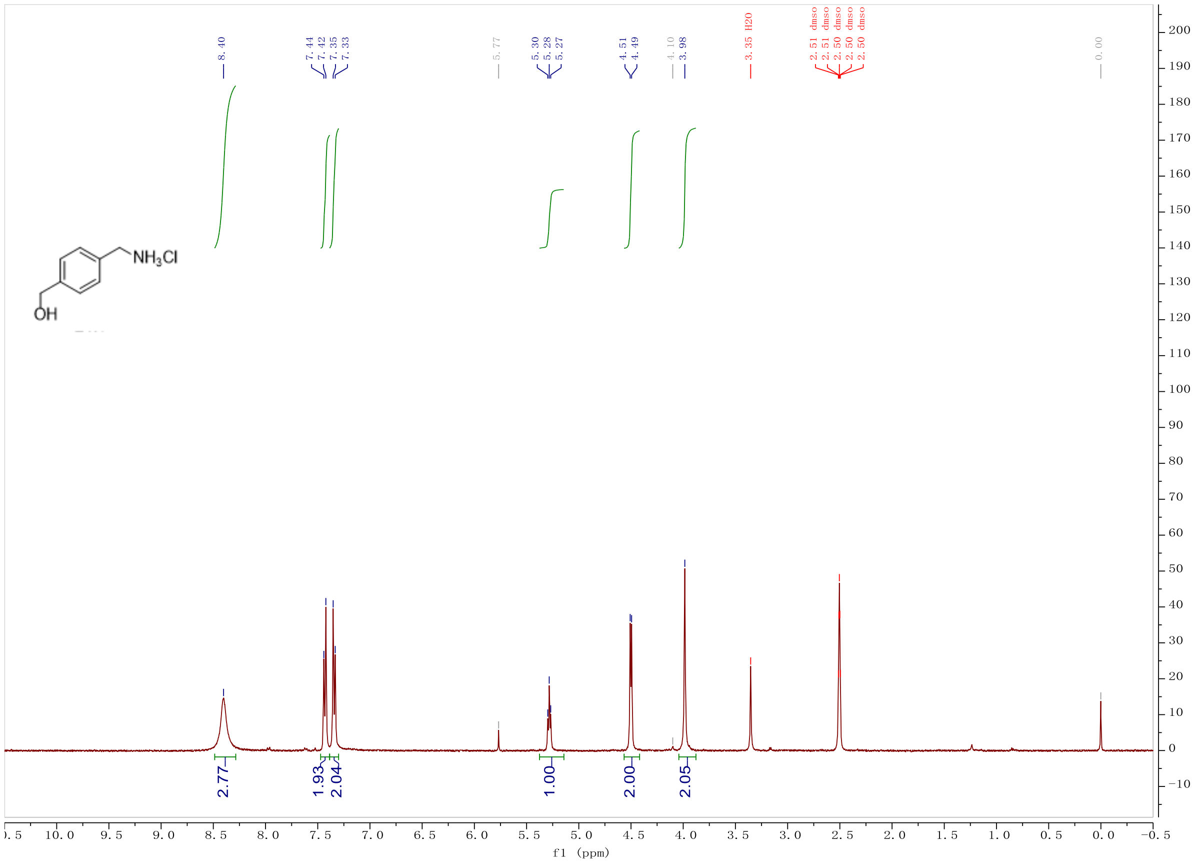

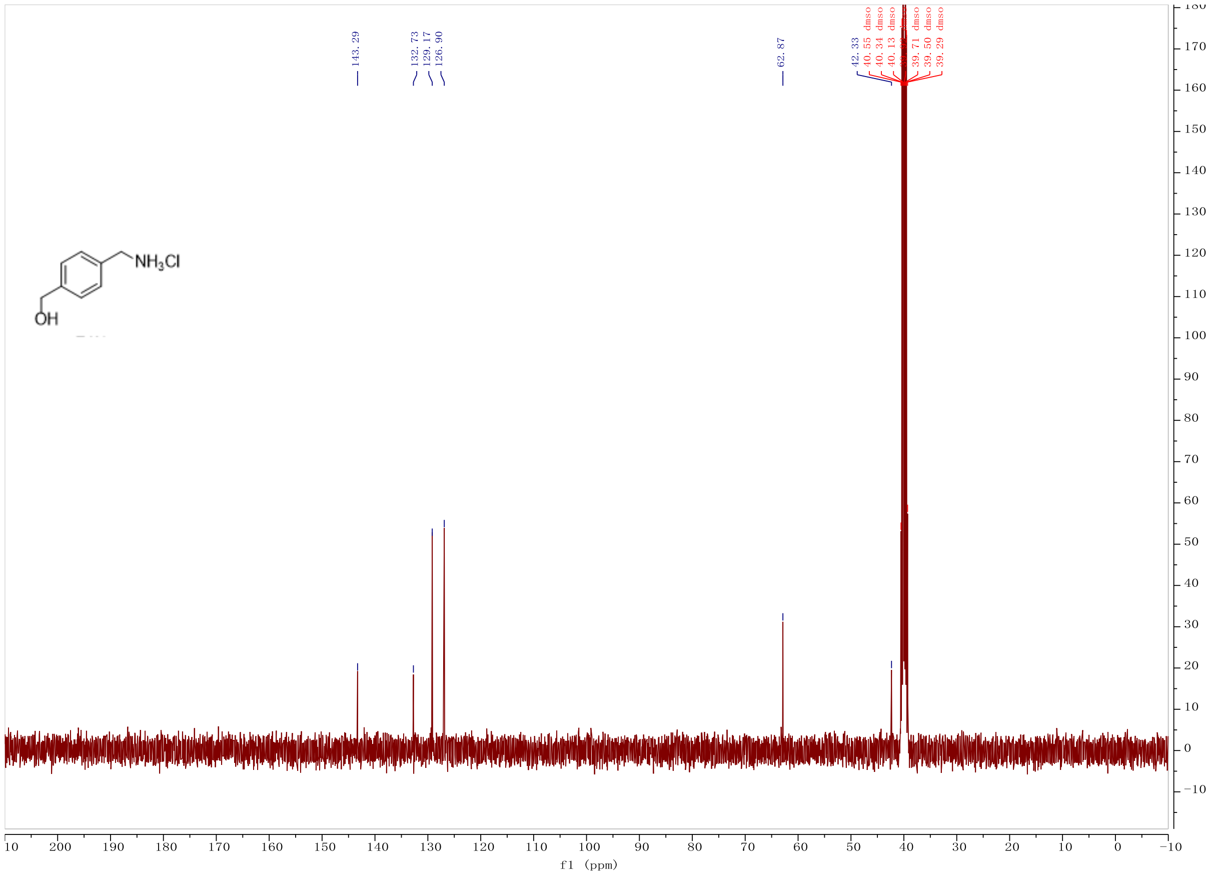

Supplementary Figure 48. **^{1}H NMR** (400 MHz, DMSO-d_6) and **^{13}C NMR** (101 MHz, DMSO-d_6) of compound **7y**.

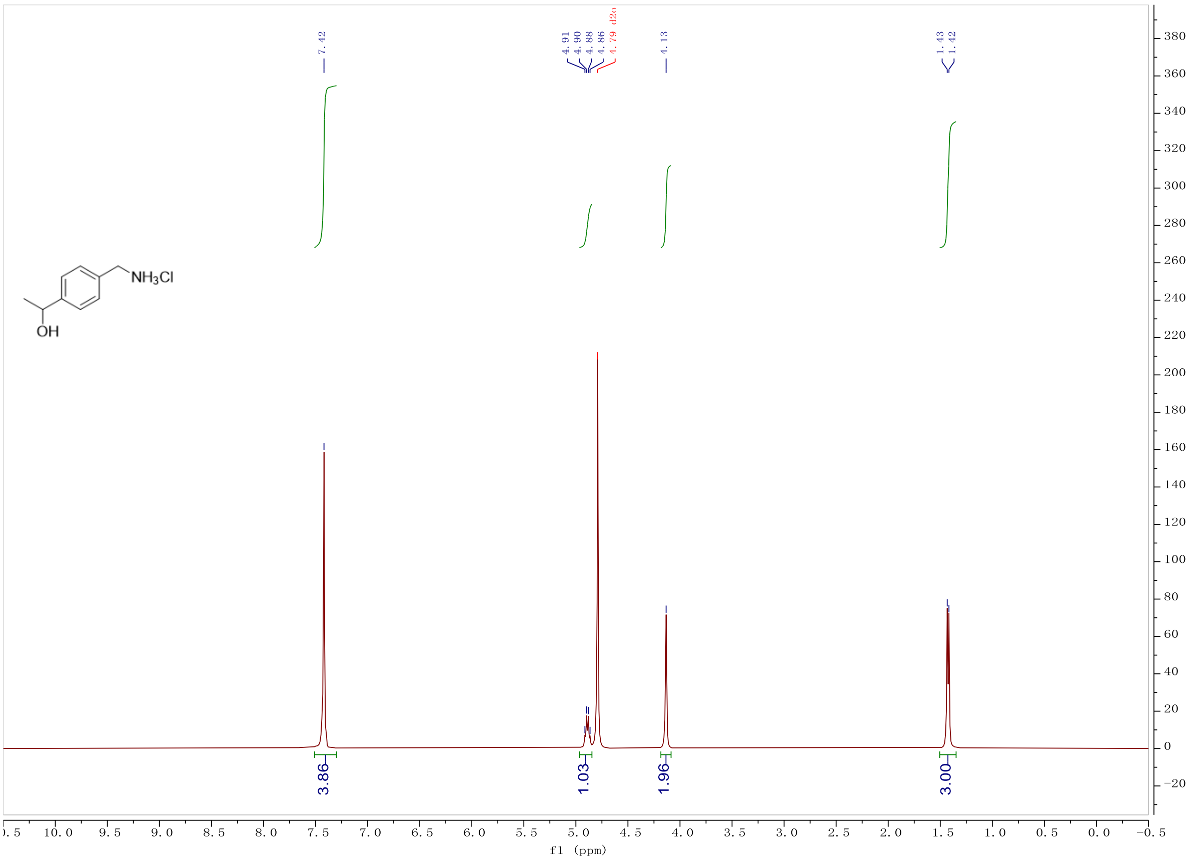

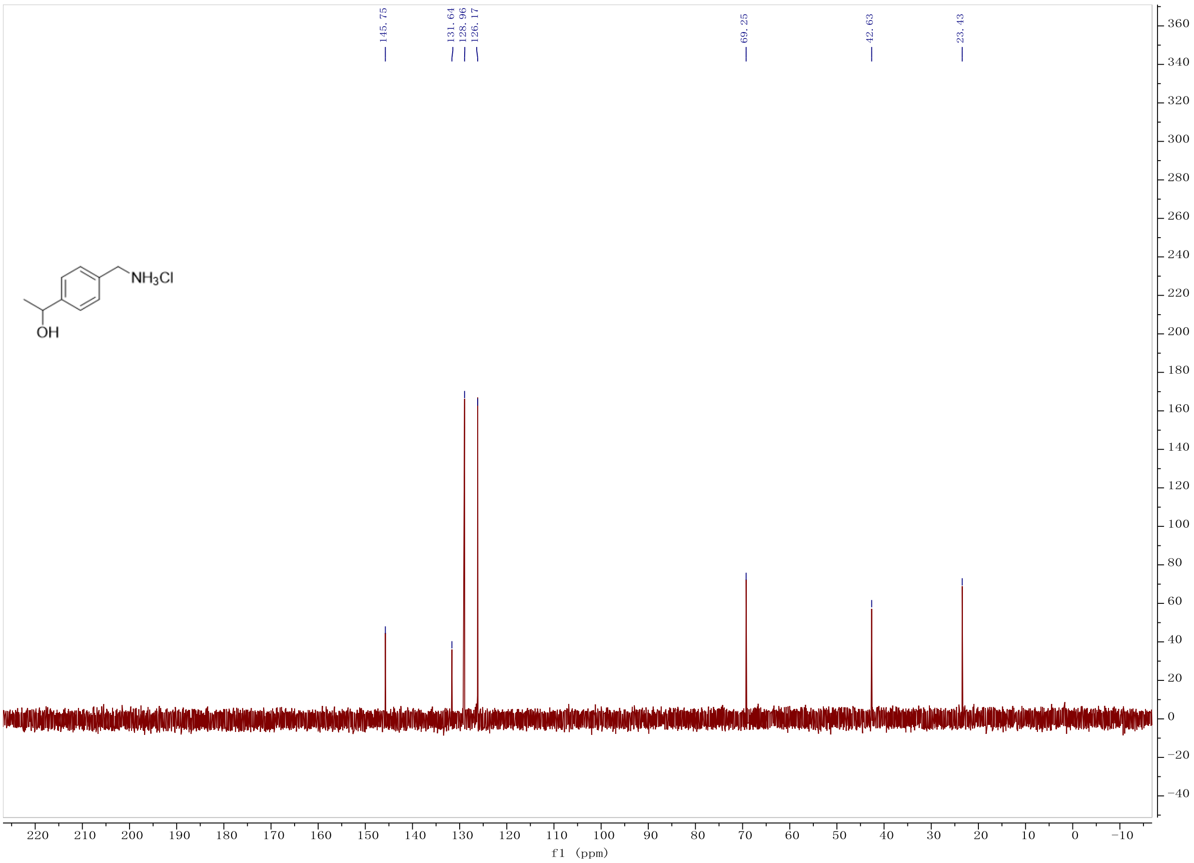

Supplementary Figure 49. **^{1}H NMR** (400 MHz, D_2O) and **^{13}C NMR** (101 MHz, D_2O) of compound **7z**.

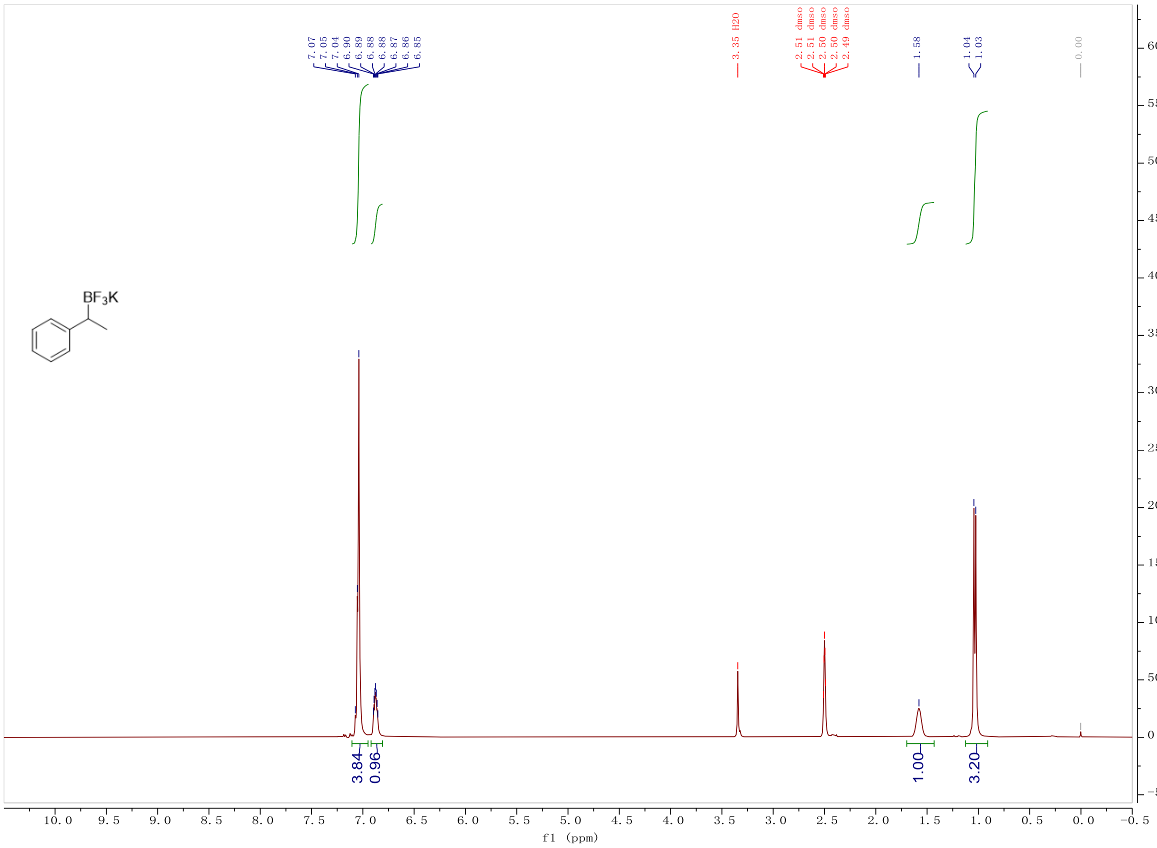
BF3K
7.07
7.05
7.04
6.90
6.89
6.88
6.88
6.87
6.86
6.85
3.35 H2O
2.51 dmso
2.51 dmso
2.50 dmso
2.50 dmso
2.49 dmso
1.58
1.04
1.03
0.00
3.84
0.96
1.00
3.20
f1 (ppm)

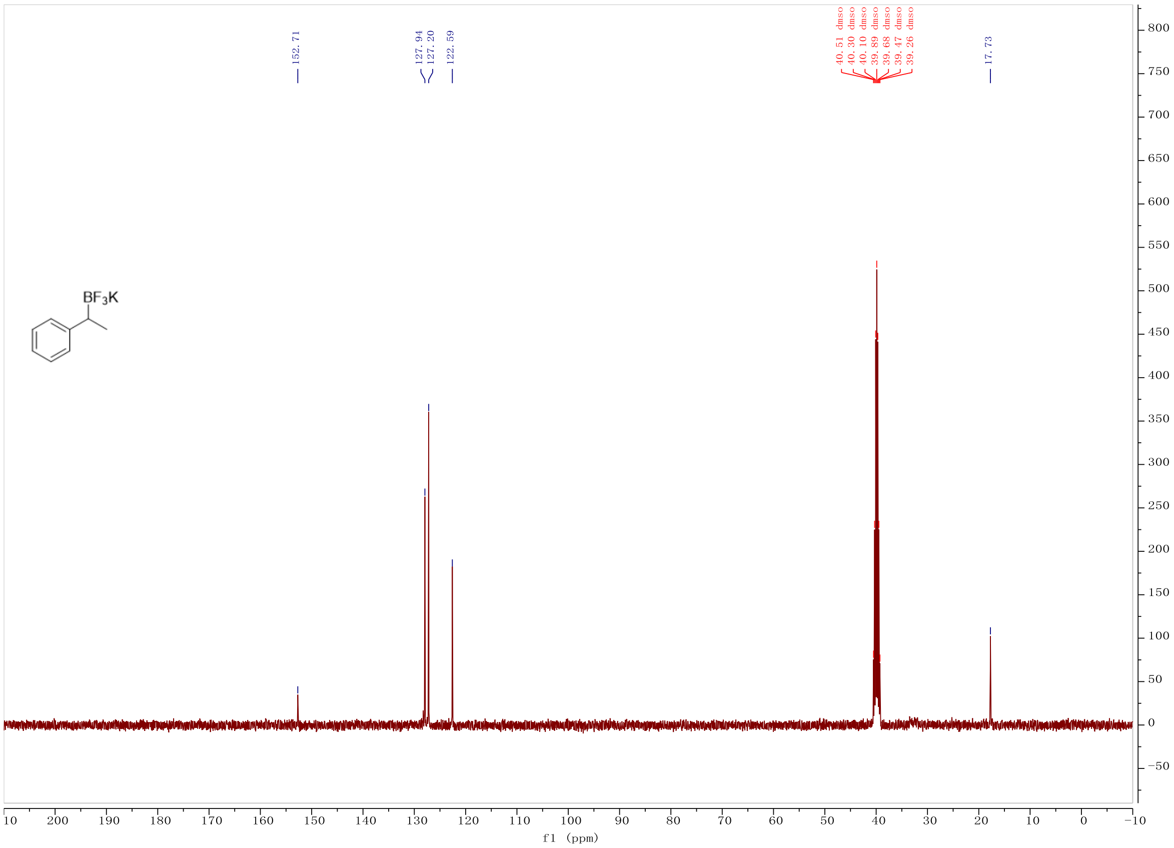
BF3K
152.71
127.94
127.20
122.59
40.51 dmso
40.30 dmso
40.10 dmso
39.89 dmso
39.68 dmso
39.47 dmso
39.26 dmso
17.73
f1 (ppm)

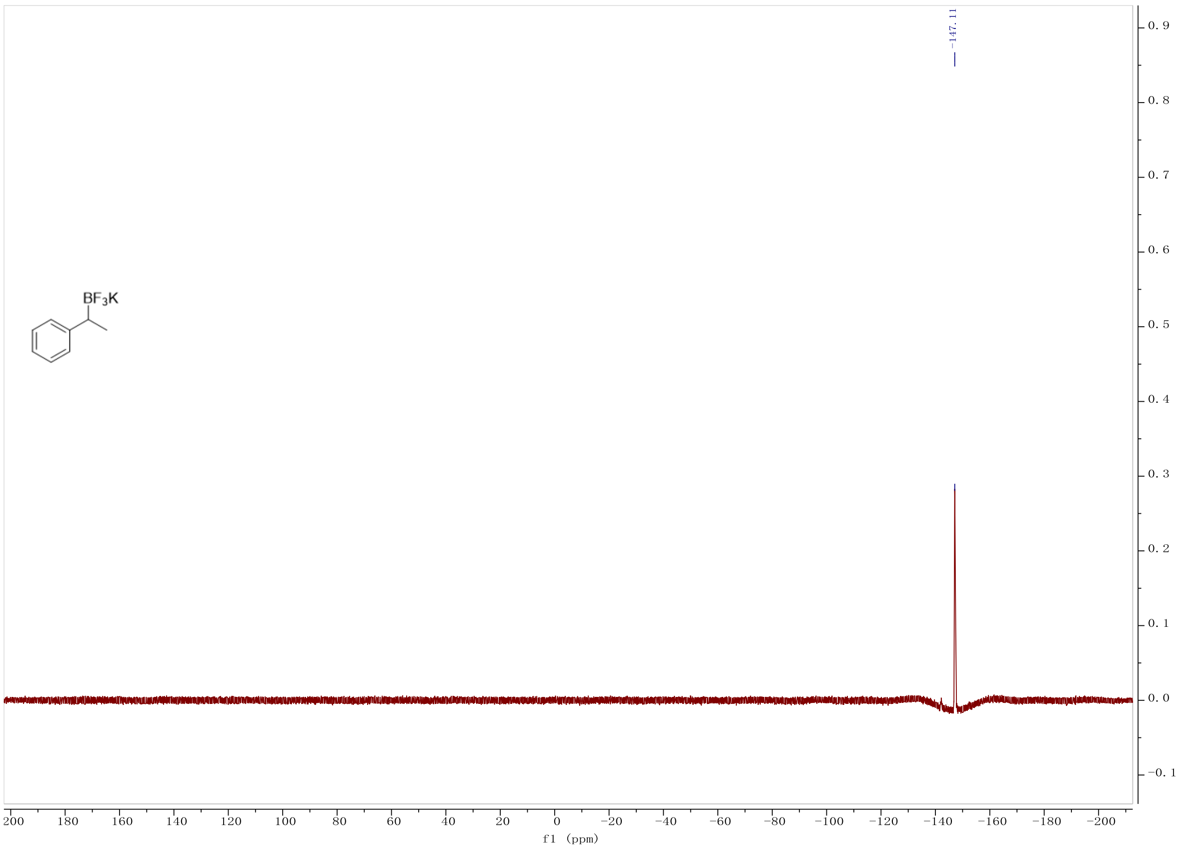

Supplementary Figure 50. **^{1}H NMR** (400 MHz, DMSO-d_6), **^{13}C NMR** (101 MHz, DMSO-d_6), and **^{19}F NMR** (376 MHz, DMSO-d_6) of compound **2a-I**.

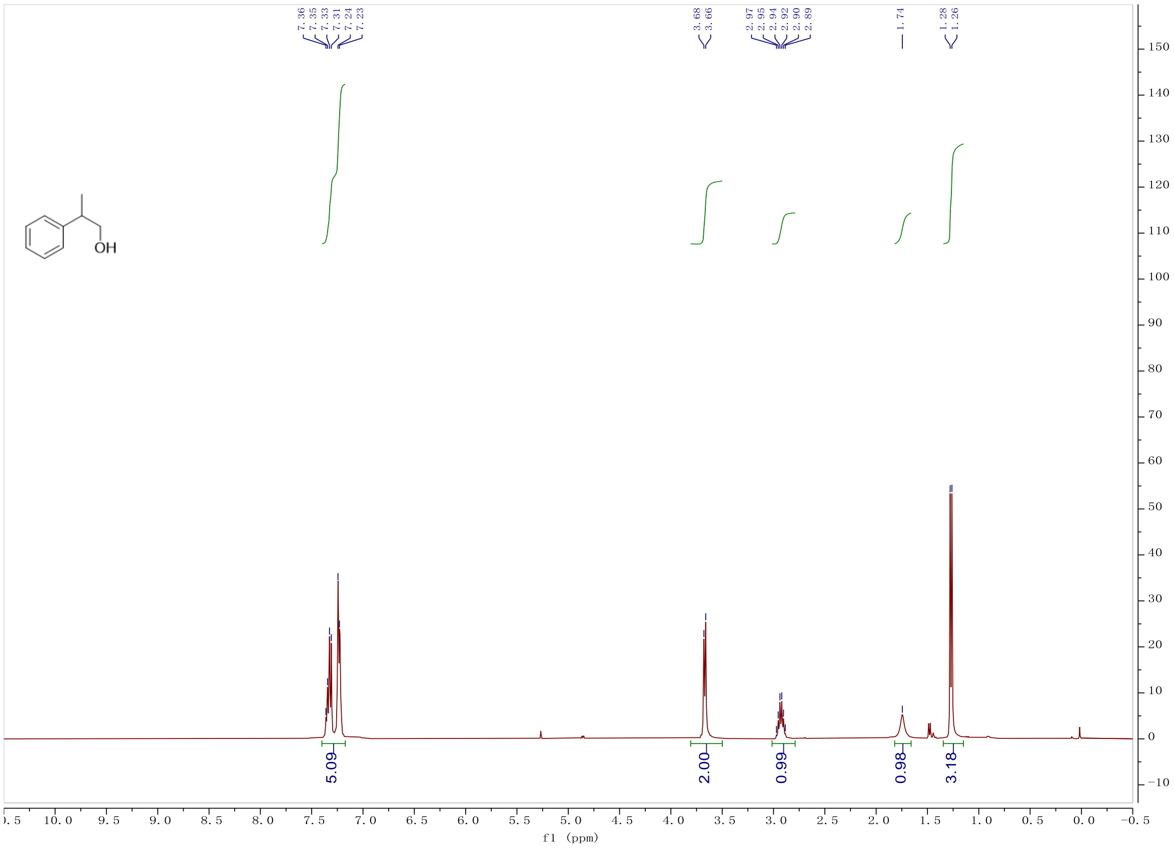

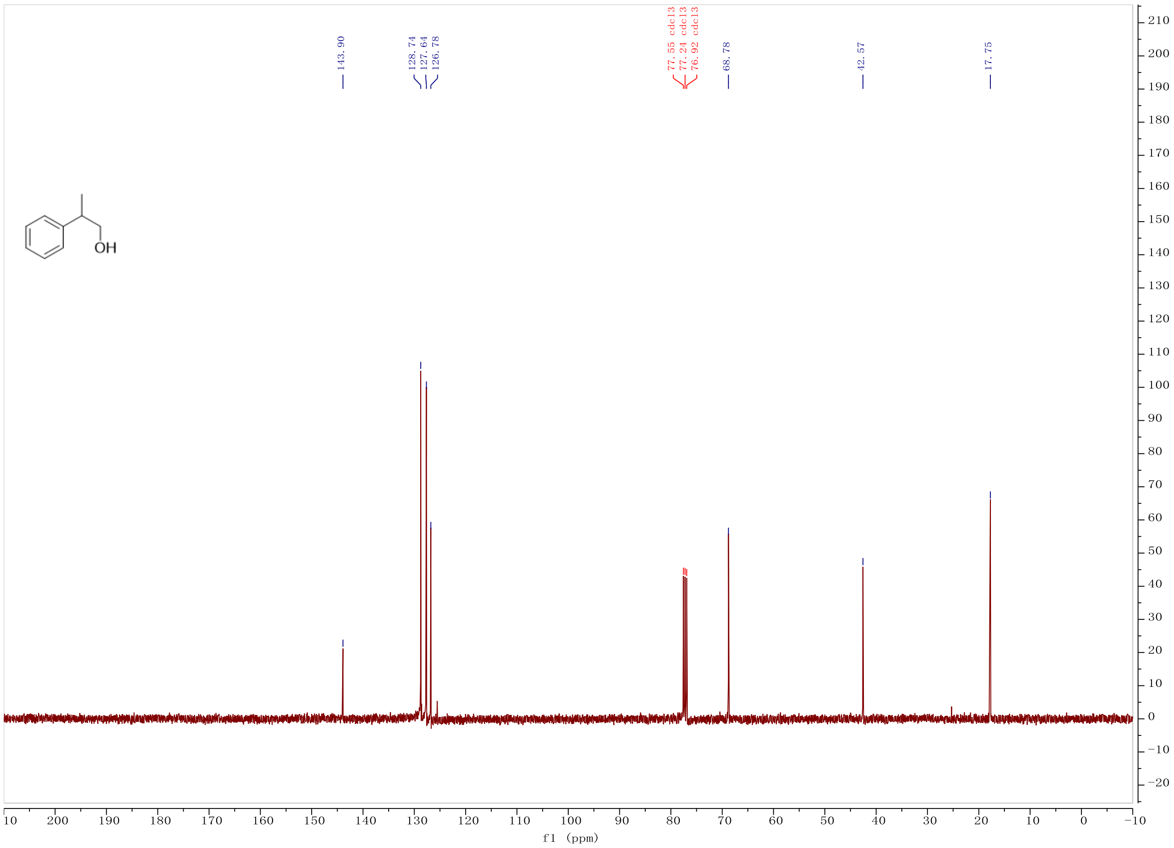

Supplementary Figure 51. ^{1}**H NMR** (400 MHz, $CDCl_3$) and ^{13}**C NMR** (101 MHz, $CDCl_3$) of compound **2a-II**.

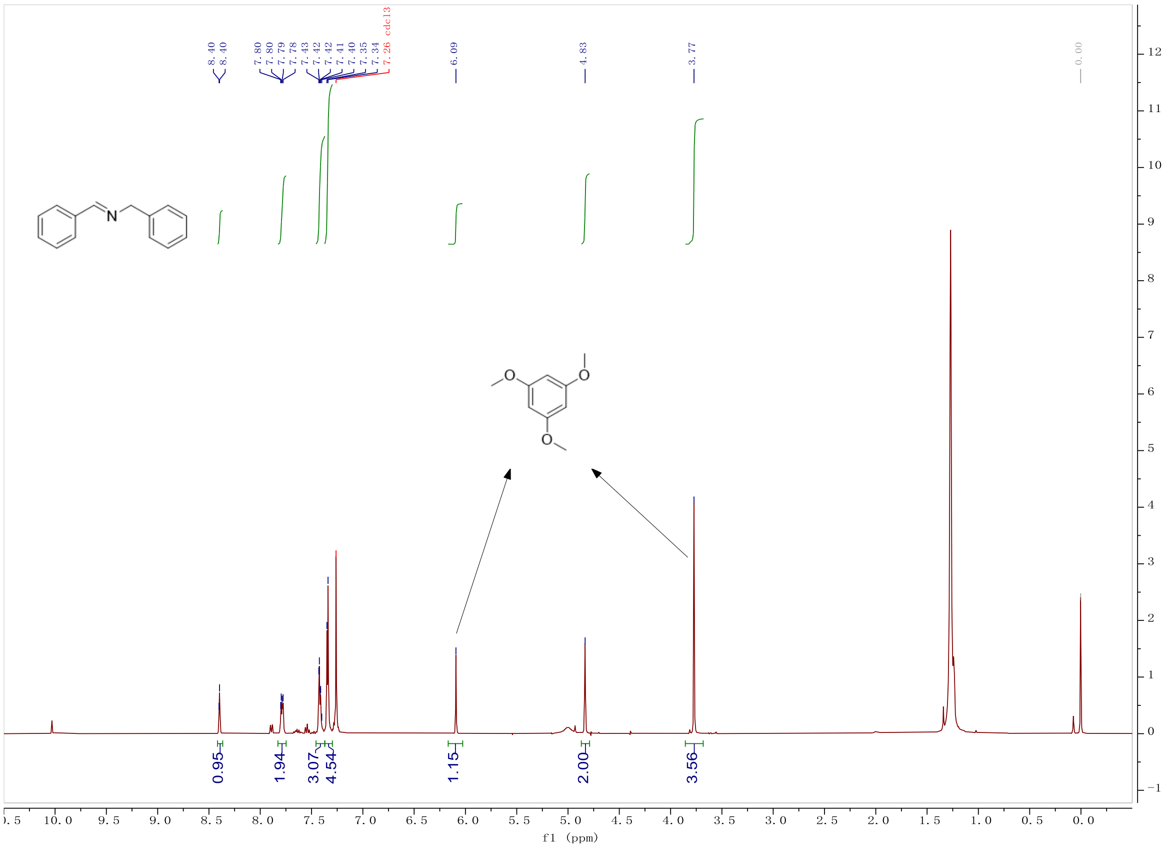

Supplementary Figure 52. **^{1}H NMR** (400 MHz, $CDCl_3$) of compound **6a-I**.

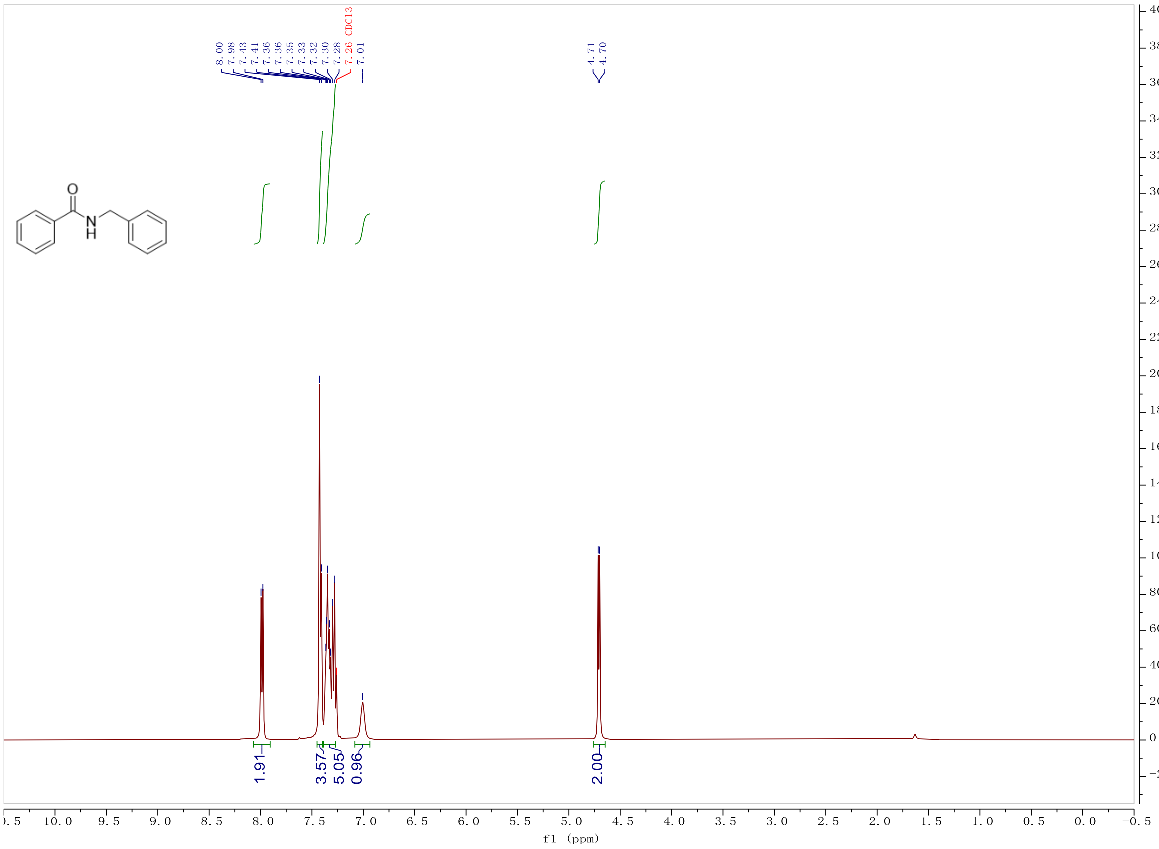

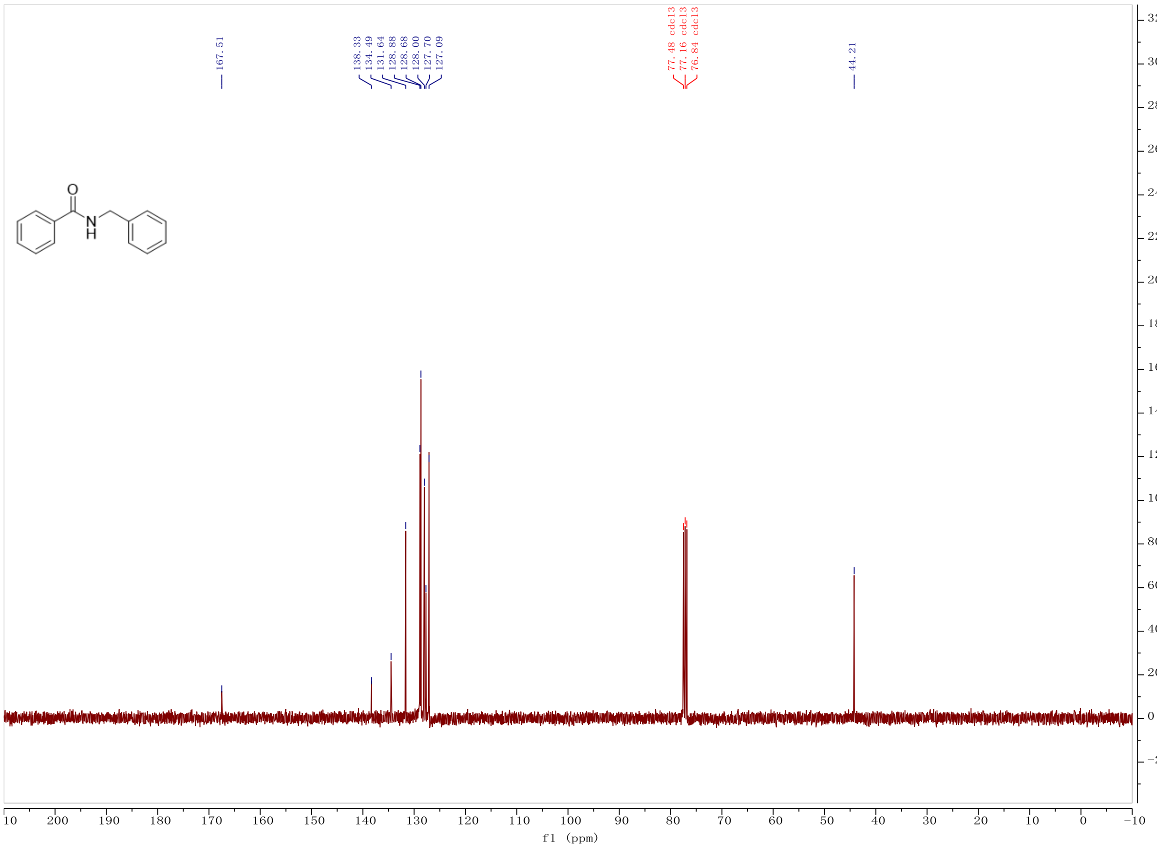

Supplementary Figure 53. **^{1}H NMR** (400 MHz, $CDCl_3$) and **^{13}C NMR** (101 MHz, $CDCl_3$) of compound **6a-II**.

9. Supplementary References

1.Boussard, C., Klimkait, T., Mahmood, N., Pritchard, M. & Gilbert, I. H. Design, synthesis and evaluation of potential inhibitors of HIV gp120-CD4 interactions. *Bioorg. Med. Chem. Lett.* **14**, 2673-2676 (2004).

2. Peng, J., Docherty, J. H., Dominey, A. P. & Thomas, S. P. Cobalt-catalysed Markovnikov selective hydroboration of vinylarenes. *Chem. Commun.* **53**, 4726-4729 (2017).

3. Tamang, S. R. *et al.* Cobalt-Catalyzed Hydroboration of Alkenes, Aldehydes, and Ketones. *Org. Lett.* **20**, 6695-6700, (2018).

4. Chen, X., Cheng, Z. & Lu, Z. Iron-Catalyzed, Markovnikov-Selective Hydroboration of Styrenes. *Org. Lett.* **19**, 969-971 (2017).

5. Verma, P. K., A, S. S. & Geetharani, K. Markovnikov-Selective Co(I)-Catalyzed Hydroboration of Vinylarenes and Carbonyl Compounds. *Org. Lett.* **20**, 7840-7845 (2018).

6. Sanagawa, A. & Nagashima, H. Hydrosilane Reduction of Nitriles to Primary Amines by Cobalt-Isocyanide Catalysts. *Org. Lett.* **21**, 287-291 (2019).

7. Bornschein, C., Werkmeister, S., Junge, K. & Beller, M. TBAF-catalyzed hydrosilylation for the reduction of aromatic nitriles. *New J. Chem.* **37**, 2061-2065 (2013).

8. Leischner, T., Spannenberg, A., Junge, K. & Beller, M. Synthesis of Molybdenum Pincer Complexes and Their Application in the Catalytic Hydrogenation of Nitriles. *ChemCatChem* **12**, 4543-4549 (2020).

9. Guan, Q. *et al.* Direct cycle between co-product and reactant: an approach to improve the atom economy and its application in the synthesis and protection of primary amines. *Green Chem.* **18**, 5794-5799 (2016).

10. Oestreich, M. & Wübbolt, S. Exhaustive Chemoselective Reduction of Nitriles by Catalytic Hydrosilylation Involving Cooperative Si–H Bond Activation. *Synlett* **28**, 2411-2414 (2017).

11. Gandhamsetty, N., Jeong, J., Park, J., Park, S. & Chang, S. Boron-Catalyzed Silylative Reduction of Nitriles in Accessing Primary Amines and Imines. *J. Org. Chem.* **80**, 7281-7287 (2015).

12. Cao, L., Hettiarachchi, G., Briken, V. & Isaacs, L. Cucurbit[7]uril containers for targeted delivery of oxaliplatin to cancer cells. *Angew. Chem. Int. Ed.* **52**, 12033-12037 (2013).

13. Wang, A., Chang, Z., Liu, Y. & Huang, P. Mild N-deacylation of secondary amides by alkylation with organocerium reagents. *Chin. Chem. Lett.* **26**, 1055-1058 (2015).

14. Hidalgo Ramos, P., Saisaha, P., Elemans, J. A. A. W., Rowan, A. E. & Nolte, R. J. M. Conformational Analysis and Binding Properties of a Cavity Containing Porphyrin Catalyst Provided with Urea Functions. *Eur. J. Org. Chem.* **2016**, 4487-4495 (2016).

15. Paul, B., Chakrabarti, K. & Kundu, S. Optimum bifunctionality in a 2-(2-pyridyl-2-ol)-1,10-phenanthroline based ruthenium complex for transfer hydrogenation of ketones and nitriles: impact of the number of 2-hydroxypyridine fragments. *Dalton Trans* **45**, 11162-11171 (2016).

16. Cazorla, C., Métay, E. & Lemaire, M. Oxidative nucleophilic substitution: transformation of alkylboronic derivatives. *Tetrahedron* **67**, 8615-8621 (2011).

17. Ren, L. & Crudden, C. M. Homologations of boronate esters: the first observation of sequential insertions. *Chem. Commun.* 721-722 (2000).

18. Gudun, K. A., Slamova, A., Hayrapetyan, D. & Khalimon, A. Y. Efficient Co-Catalyzed Double Hydroboration of Nitriles: Application to One-Pot Conversion of Nitriles to Aldimines. *Chem. Eur. J.* **26**, 4963-4968 (2020).

19. Ghosh, C. *et al.* Efficient Cobalt Catalyst for Ambient-Temperature Nitrile Dihydroboration, the Elucidation of a

Chelate-Assisted Borylation Mechanism, and a New Synthetic Route to Amides. *J. Am. Chem. Soc.* **141**, 15327-15337 (2019).

20. Buslov, I., Song, F. & Hu, X. An Easily Accessed Nickel Nanoparticle Catalyst for Alkene Hydrosilylation with Tertiary Silanes. *Angew. Chem. Int. Ed.* **55**, 12295-12299 (2016).

21. Tamang, S. R. & Findlater, M. Cobalt catalysed reduction of CO_2 via hydroboration. *Dalton Trans.* **47**, 8199-8203 (2018).

22. Kuciński, K. & Hreczycho, G. Lithium triethylborohydride as catalyst for solvent-free hydroboration of aldehydes and ketones. *Green Chem.* **21**, 1912-1915 (2019).
